# Whole genome sequencing of one complex pedigree illustrates challenges with genomic medicine

**DOI:** 10.1186/s12920-017-0246-5

**Published:** 2017-02-23

**Authors:** Han Fang, Yiyang Wu, Hui Yang, Margaret Yoon, Laura T. Jiménez-Barrón, David Mittelman, Reid Robison, Kai Wang, Gholson J. Lyon

**Affiliations:** 1 Stanley Institute for Cognitive Genomics, One Bungtown Road, Cold Spring Harbor Laboratory, Cold Spring Harbor, NY USA; 2 Stony Brook University, 100 Nicolls Rd, Stony Brook, NY USA; 3 Simons Center for Quantitative Biology, One Bungtown Road, Cold Spring Harbor Laboratory, Cold Spring Harbor, NY USA; 4 Centro de Ciencias Genomicas, Universidad Nacional Autonoma de Mexico, Cuernavaca, Morelos, MX Mexico; 5 Tute, Genomics Inc., 150 S 100 W, Provo, UT USA; 6 Utah Foundation for Biomedical Research, Salt Lake City, UT USA; 7 Zilkha Neurogenetic Institute, University of Southern California, Los Angeles, CA USA; 8 Neuroscience Graduate Program, University of Southern California, Los Angeles, CA USA; 9 Department of Psychiatry, University of Southern California, Los Angeles, CA USA; 10 Division of Bioinformatics, Department of Preventive Medicine, University of Southern California, Los Angeles, CA USA; 11 Present Address: Department of Biomedical Informatics and Institute for Genomic Medicine, Columbia University Medical Center, New York, 10032 NY USA

**Keywords:** Whole genome sequencing, Precision medicine, Human phenotype ontology, Phenolyzer, Variant calling, Prader–Willi Syndrome, Dysautonomia, Hemochromatosis

## Abstract

**Background:**

Human Phenotype Ontology (HPO) has risen as a useful tool for precision medicine by providing a standardized vocabulary of phenotypic abnormalities to describe presentations of human pathologies; however, there have been relatively few reports combining whole genome sequencing (WGS) and HPO, especially in the context of structural variants.

**Methods:**

We illustrate an integrative analysis of WGS and HPO using an extended pedigree, which involves Prader–Willi Syndrome (PWS), hereditary hemochromatosis (HH), and dysautonomia-like symptoms. A comprehensive WGS pipeline was used to ensure reliable detection of genomic variants. Beyond variant filtering, we pursued phenotypic prioritization of candidate genes using Phenolyzer.

**Results:**

Regarding PWS, WGS confirmed a 5.5 Mb *de novo* deletion of the parental allele at 15q11.2 to 15q13.1. Phenolyzer successfully returned the diagnosis of PWS, and pinpointed clinically relevant genes in the deletion. Further, Phenolyzer revealed how each of the genes is linked with the phenotypes represented by HPO terms. For HH, WGS identified a known disease variant (p.C282Y) in *HFE* of an affected female. Analysis of HPO terms alone fails to provide a correct diagnosis, but Phenolyzer successfully revealed the phenotype-genotype relationship using a disease-centric approach. Finally, Phenolyzer also revealed the complexity behind dysautonomia-like symptoms, and seven variants that might be associated with the phenotypes were identified by manual filtering based on a dominant inheritance model.

**Conclusions:**

The integration of WGS and HPO can inform comprehensive molecular diagnosis for patients, eliminate false positives and reveal novel insights into undiagnosed diseases. Due to extreme heterogeneity and insufficient knowledge of human diseases, it is also important that phenotypic and genomic data are standardized and shared simultaneously.

**Electronic supplementary material:**

The online version of this article (doi:10.1186/s12920-017-0246-5) contains supplementary material, which is available to authorized users.

## Background

Many genetic tests have been commonly performed on individuals that have phenotypes overlapping with known diseases, especially for cancer and rare diseases [1–4]. Physicians have also been routinely prescribing prenatal genetic tests and newborn screenings in clinics [5–7]. However, there is a degree of uncertainty inherent in most genetic testings regarding the development, age of onset, and severity of disease [8]. In addition, current genetic testing has not yet established predictive or even diagnostic value for common complex diseases [9]. Some groups have begun to leverage the power of next-generation sequencing (NGS) to help diagnose rare diseases [10–13]. Many studies have used whole exome sequencing (WES) to facilitate the molecular diagnosis of individuals with diseases that appear to have a single large-effect size mutation contributing substantially to the development of the disease, referred to by many as “Mendelian disorders” [14, 15]. Of course, such disorders also have an extraordinary phenotypic variability and spectrum brought about by genetic background, environmental differences and stochastic developmental variation (SDV) [16–20].

Despite much success using NGS-based techniques to identify mutations, there are still practical issues for the analytic validity for exome- or genome-wide NGS-based techiques, particularly in clinical settings [21, 22]. The clinical utility of genomic medicine is also uncertain, prompting some to suggest the need for better standards and benchmarking [23, 24]. However, the genetic architecture behind human disease is heterogeneous, and there are many reports of regulatory variants in the non-coding genome and splicing variants in the intronic regions that have a large-effect size on particular phenotypes [25–30]. In hypothesis-driven research studies, one might gain higher statistical power with a larger sample size by using cheaper NGS assays like WES or gene panels. But whole genome sequencing (WGS) has a unique strength in its ability to cover a broader spectrum of variants; small insertions and deletions (INDELs), structual variants (SVs), and copy number variants (CNVs). This becomes extremely valuable in studies where disease associated variants might not be necessarily SNVs [31–33]. In particular, from a study design perspective, WGS results in a more uniform coverage and better detection of INDELs, and is free of exome capture deficiency issues [34]. Of course, cost and technical considerations are still practical issues for WGS, but this will eventually become the optimal assay to address the extreme heterogeneity of different genetic architectures for different diseases.

Human Phenotype Ontology (HPO) has risen as a useful techique for precision medicine by providing a standardized vocabulary bank of phenotypic abnormalities to describe presentations of human pathologies [35–37]. Some showed that phenotypic matching can help interpret CNV findings based on integrated cross-species phenotypic information [38]. The potential clinical usage of HPO derives from a wealth of medical literature and databases such as Online Mendelian Inheritance in Man (OMIM) [35]. Computational tools like Exomizer and PhenIX were developed to aid disease associated variant prioritization from exome sequencing data [39–41], and this has been recently extended with the development of Genomiser for WGS data [42]. Another tool is Phenolyzer [43], which uses prior biological knowledge and phenotype information to implicate genes involved in diseases. Phenolyzer reveals the hidden connection of genotypes and phenotypes by examing gene-gene, gene-disease and disease-phenotype interactions [43]. Based on standarized phenotypic reports, Phenolyzer can be used to further prioritize WGS findings for disease associated variants.

We report here a comprehensive analysis of an extended pedigree, including genomics filtering on WGS data and phenotypic prioritization of candidate genes using Phenolyzer. The pedigree involves probands with Prader–Willi Syndrome (PWS) [44, 45], Hereditary Hemochromatosis (HH), dysautonomia-like symptoms, Tourette Syndrome (TS) [46] and other illnesses. We specifically chose this family for whole genome sequencing due to the phenotypic complexity in the family, including at least one genetic syndrome with a known genetic etiology, which on some level serves as a positive control among a range of diseases of unknown (or controversial) genetic architecture. Nine members of the family underwent WGS, enabling a wide scope of variant calling from SNVs to large copy number events. Notably, this is the first report of Illumina HiSeq WGS experiement on a PWS individual carrying the paternally-inherited deletion. The use of WGS enables the reconstruction of the recombination event in this imprinting hotspot, which provides a better understanding of the PWS disease mechanism. This report emphasizes the effectiveness of Phenolyzer, which can be used to integrate and share WGS and HPO data. Neither technique is yet perfect for clinical diagnosis, but combining the two can help eliminate false positives and reveal novel insights into human diseases.

## Methods

### Clinical phenotyping of individuals participating in this study

The family was interviewed by the corresponding author, GJL, a board-certified child, adolescent and adult psychiatrist. Medical records were obtained and reviewed, in conjunction with further interviews with the family. The interviews were videotaped and later reviewed to facilitate further diagnostic efforts. Various clinical diagnostic testings were performed on K10031-10133, including tilt table test, brain MRI, ultrasound of the kidneys and chest X-ray. In addition, her cholesterol level, thyroid profile, urine vanillylmandelic acid (VMA), catecholamines panel (urine-free), basic metabolic panel (BMP), and epinephrine and norepinephrine levels were also screened. Other clinical tests included electrocardiogram (EKG), polysomnographic report, and echocardiogram. For K10031-10232, the following diagnostic evaluations were performed: multiple sleep latency test (MSLT) [47], autism diagnostic observation system (ADOS) - module 2 [48], the Childhood Autism Rating Scale (CARS) [49], Behavior Assessment System for Children (BASC) [50], Intelligence Quotient (IQ), and Abnormal Involuntary Movement Scale (AIMS) [51].

### Generation of WGS and microarray data

Blood and saliva samples were collected from nine individuals (K10031-10143, 10144, 10145, 10235, 10133, 10138, 10231, 10232, 10233) from the extended pedigree described in the results. Two CLIA-certified WGS tests (K10031-10133 and K10031-10138) were performed at Illumina, San Diego. The other seven WGS runs were performed at the sequencing center at Cold Spring Harbor Laboratory (CSHL). All libraries were constructed with PCR amplification, and sequenced on one Illumina HiSeq2000 with an average paired-end read length of 100 bp. Since the DNA extracted from saliva samples contains a certain proportion of bacterial DNA, these samples were sequenced on additional lanes to achieve an average coverage of 40X after removing unmapped reads (Additional file 1: Table S1). Microarray data for the same samples were generated with the Illumina Omni 2.5 microarray at the Center for Applied Genomics Core of the Children’s Hospital of Philidephia (CHOP). Illumina Genome Studio was used to extract the SNV calls and log R ratio (LRR) and B allele frequency (BAF) from the microarray data. The general analysis work-flow is shown in Additional file 1: Fig. S1.

### Alignment and variant calling of WGS data

All of the unmapped raw reads were excluded to remove the sequence reads coming from the bacterial DNA (step 2 of Additional file 1: Fig. S1). The remaining reads were aligned to human reference genome (build hg19) with BWA-mem (v0.7-6a) [52]. In parallel, reads were also aligned with NovoAlign (v3.00.04) to reduce false negatives resulting from alignment artifacts. All of the alignments were sorted with SAMtools (v0.1.18) and PCR duplicates marked with Picard (v1.91) [53]. For the BWA-MEM bam files, INDELs were realigned with the GATK IndelRealigner (v2.6-4) and base quality scores were recalibrated [54]. For variant calling with FreeBayes, the alignment files were not processed with INDEL-realignment and base quality recalibration as these additional steps are not required by FreeBayes. Qualimap (v2.0) was used to perform QC analysis on the alignment files [55].

In order not to miss potentially disease-contributory variants, more than one pipeline were used to detect SNVs, INDELs, SVs, and CNVs [56, 57]. All variants are included in the downstream analysis and orthogonal validations were performed to confirm the variants of interest (step 3 to step 5 of Additional file 1: Fig. S1). First, SNVs and INDELs were jointly called from nine genomes with GATK HapolotypeCaller (v3.1-1) from the BWA-MEM alignment following best practices [58]. Second, a default parameter setting was used to call variants using FreeBayes from the NovoAlign alignment [59]. Third, Scalpel (v0.1.1) was used with the BWA-MEM bam files to identify INDELs in the exonic regions with sizes up to 100 bp [60]. Each exon was expanded by 20 bp upstream and 20 bp downstream to reveal possible INDELs harboring splicing sites. Following the benchmarking results as recently reported [34], Scalpel INDEL calls were filtered out if they have an alternative allele coverage less than five and a Chi-Square socre greater than 10.8. Fourth, RepeatSeq (v0.8.2) was utilized to detect variants near short tandem repeats regions in the genome using default settings [61]. Fifth, Lumpy (v 0.2.6) and CNVnator were both used to call SVs with sizes >100 bp [62, 63]. Among Lumpy calls, events supported by >50 reads or less than four reads were excluded because regions of either too low or high coverage are more likely to contain biases in sequencing or alignment. Sixth, ERDS (v1.1) was used to call CNVs from the BWA-mem bam files with default settings [64]. Among ERDS calls with a confidence score >300, duplications with sizes < 200 Kb and deletion calls with sizes <10 Kb were excluded from downstream analysis. CNVnator (v0.3) was used to identify smaller CNVs that are present in the WGS data using the parameters -his 100, −stat 100, −partition 100, −call 100 [63]. Sixth, to achieve high confidence CNV calls, PennCNV (2011Jun16 version) was used to call CNVs from the microarray data [65]. Each CNV was supported by at least 10 markers, excluding CNVs with an inter-marker distance of >50 Kb. SVs and CNVs that overlapped with segmental duplication regions by 50% were also filtered out with BEDtools [66].

### Genomic filtering and annotations of the variants

To annotate the variants of interest, GEMINI (v0.11.0), ANNOVAR (2013Aug23 version) were used (step 6 of Additional file 1: Fig. S1) [67, 68]. The circos plot of K10031-10232’s genome was generated using circlize in R [69]. The population allele frequencies (AF) were loaded with GEMINI from the 1000G database (http://www.1000genomes.org/) and Exome Aggregation Consortium (ExAC) database (http://exac.broadinstitute.org/) [70]. GEMINI also served to import the CADD C-scores, loss-of-function variants defined by LOFTEE, and the reported pathogenicity information from the ClinVar database [71, 72]. There were several steps in filtering variants with respect to the segregation pattern, population frequency, allele deleteriousness prediction, and ClinVar annotation. First, variants were partitioned by the following disease inheritance models: autosomal dominant, autosomal recessive, *de novo*, compound heterzygous, and X-linked dominant. Second, autosomal or X-linked dominant and *de novo* variants were excluded if they had an AAF >0.01 in either ExAC or 1000G database while the cut-off was increased to 0.05 for autosomal recessive and compound heterzygous variants. Third, only the variants that met the following criteria were considered in the downstream analysis: 1) called by at least one pipeline and validated with a second pipeline, 2) had an adjusted p-value lower than 0.05 reported by pVAAST [73], 3) defined as medium or high impact by GEMINI, or defined as loss-of-function by LOFTEE, 4) with a CADD c-score greater than 15. Fourth, we also searched for variants that were considered as pathogenic, probably-pathogenic, mixed, or drug-response in the ClinVar database. Lastly, the VCF files were also uploaded to the Omicia Opal platform and the Tute Genomics platform for online annotation, filtering, and pharmacogenomic analysis. The Tute Genomics variant interpretation report for each individual can be found in Additional file 2.

### Phenotypic prioritization of candidate genes using Phenolyzer

Clinical features of K10031-10232, K10031-10133, and K10031-10145 were mapped to HPO terms using the Phenomizer clinical diagnostics tool [74]. Complete Phenomizer diagnosis forms are available in supplemental files. Phenolyzer was used for phenotypic prioritization of the genomic variants in above three probands. For each proband, we first performed a genomic filtering of the WGS data, compiling a list of candidate genes and genomic intervals of SVs and CNVs. Then we uploaded the filtered list and their HPO terms to Phenolyzer for phenotypic prioritization. HPO terms were generated for K10031-10232, K10031-10145, K10031-10133 (see Table 1, Table 2, Additional file 1: Supplemental Table S5, S6, and S10).

**Table 1:** Main Clinical Presentation of Proband K10031-10232

Clinical manifestations	HPO#
*Development and growth*	
Delayed speech and language development	0000750
Growth hormone deficiency	0000824
Poor fine motor coordination	0007010
Mild intellectual disability	0001256
*Facial features*	
Almond-shaped eyes	0007874
Downslanted palpebral fissures	0000494
Narrow forehead	0000341
*Other physical features*	
Cryptorchidism	0000028
Excessive daytime sleepiness	0002189
Obstructive sleep apnea syndrome	0002870
Scoliosis	0002650
*Behavior features*	
Aggressive behavior	0000718
Anxiety	0000739
Depression	0000716
Impaired ability to form peer relationships	0000728
Impaired social reciprocity	0012760
Inflexible adherence to routines or rituals	0000732
Irritability	0000737
Low frustration tolerance	0000744
Obsessive-compulsive disorder	0000722
Pain insensitivity	0007021
Polyphagia	0002591
Poor eye contact	0000817
Restrictive behavior	0000723
Short attention span	0000736

For a full version of the table, please refer to Additional file 1: Table S5

**Table 2:** Main Clinical Presentation of Proband K10031-10133

Clinical manifestations	HPO#
*Cardiovascular*	
Bradycardia	0001662
Patent foramen ovale	0001655
Syncope	0001279
Tachycardia	0001649
*Eyes*	
Diplopia	0000651
Peripheral vision	NF
*Gastrointestinal*	
Gastroparesis	0002578
Nausea	0002018
*Gynecologic & genitourinary*	
Urinary retention	0000016
Urinary incontinence	0000020
*Musculoskeletal*	
Arthralgia	0002829
Joint stiffness	0001387
*Neurological*	
Apraxia	0002186
Arthritis	0001369
Auditory hallucinations	0008765
Convulsions	NF
Dizziness	NF
Dysarthria	0001260
Fatigue	0012378
Frequent falls	0002359
Heat intolerance	0002046
Migraine	0002076
Seizure	0001250
Tremor (Postural/Resting)	0002174/0002322
Visual hallucinations	0002367
*Respiratory*	
Asthma	0002099
*Psychiatric*	
Anxiety	0000739
Depression	0000716

*Abbreviation*: *NF*, Not found. For a full version of the table, please refer to Additional file 1: Table S6

To find out what HPO terms affect our results the most, we performed a ranking analysis with Phenolyzer. We used individual HPO term as input and compared the Phenolyzer scores of the CNV containing NDN and SNRPN. Ideally, the higher the score, the more important this HPO term is to this CNV. Further, to understand the impact of the number of HPO terms on the final result, we randomly downsampled to a smaller number (one to six) of HPO terms from the entire set of 21. Then we used each combination as an input for Phenolyzer analysis. We defined the confidence level of a result based on the Phenolyzer score of the correct CNV; ‘High confidence’ (> = 0.5), ‘Medium confidence’ (0.1 = < Phenolyzer score <0.5) and ‘Low confidence’ (<0.1). For each scenario (one to six HPO terms), we counted the number of times when the correct CNV was prioritized at high/medium/low confidence levels. Finally, we computed and summarized the percentage of each (Fig. 6).

## Results and discussion

### Clinical presentation (with HPO annotation) and family history

**Fig. 1:**
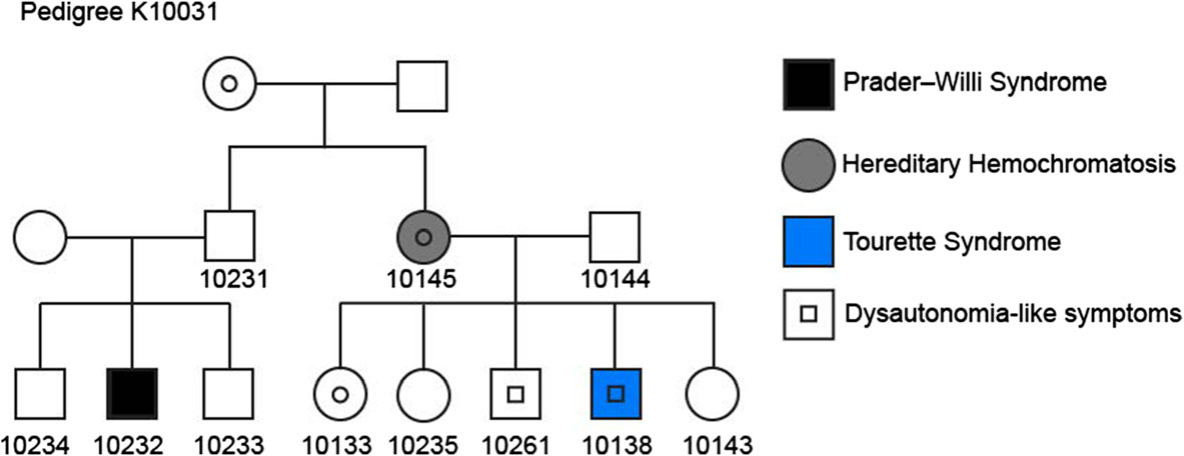
A pedigree spanning three generations with multiple diseases in this study. DNA was collected with informed consent from individuals marked with a number underneath. All samples underwent WGS except for K10031-10234 and K10031-10261

Here, we present the phenotypic characterization of a Utah pedigree K10031, consisting of 14 individuals from three generations (Fig. 1) with various medical conditions as mentioned above. The two probands we discuss in detail below come from two nuclear families in this extended pedigree.

### Proband K10031-10232

Proband K10031-10232 is a 25-year-old (25 y.o.) male. He is the son of a Caucasian farther (K10031-10231), and an Asian mother (did not participate in the study). He has two older male siblings, namely K10031-10233 and K10031-10234. This proband was diagnosed with PWS at 11 months old, and has dysmorphic facial features including a narrow forehead, downslanted palpebral fissures and almond-shaped eyes. A description of a video recording (HDV_0073) illustrating his clinical manifestations can be found in the supplemental section, and the video can be provided on request to qualified investigators. Since the PWS diagnosis, his behavior has been assessed in great detail (Table 1, and Additional file 1: Supplemental Data), and the following diagnoses have been given: obsessive-compulsive disorder (OCD), depression, anxiety disorder, pervasive developmental disorder (PDD), hyperphagia, trichotillomania, and daytime hypersomnolence. He has an IQ ranging between 60 and 65, categorized as mild mental retardation. He also has diagnoses of mild dysarthria, obstructive sleep apnea syndrome (OSAS), and severe scoliosis. The latter has been corrected surgically. He has also undergone orchiopexy, tonsillectomy, and adenoidectomy. His physical exam is otherwise unremarkable. He has denied having significant psychotic symptoms, including auditory or visual hallucinations, delusions, ideas of grandiosity, or paranoid ideation.

In an effort to help standardize phenotype reporting, we used Human Phenotype Ontology (HPO) annotation [75]. See Table 1 and Additional file 1: Table S5 for a list of clinical phenotype features collected from this proband. The Phenomizer tool [74] ranked the diagnosis for Prader-Willi Syndrome as the highest priority diagnosis for this proband (see Additional file 3), supporting the fact that highly specific and annotated phenotype information can yield accurate diagnoses, at least for a characteristic syndrome like PWS. As presented below, the genomic analysis of proband K10031-10232 further confirmed deletions in the chromosome regions from 15q11.2 to 15q13.1, making PWS the most credible diagnoses for him at present.

### Proband K10031-10133

Proband K10031-10133 is a 26 y.o. female, born to a Caucasian mother (K10031-10145) and a Caucasian father (K10031-10144). She is the eldest child amongst her two sisters and two brothers. Prior to age 18, K10031-10133 had a fairly unremarkable medical history. Arthralgia and episodes of fatigue and dizziness started at around 18 years of age. At age 20, she started to have refractory syncopal events, which led to multiple body injuries. During the same period of time, she also developed postural orthostatic tachycardia syndrome (POTS), heart palpitations, gastroparesis, urinary incontinence, diplopia, and seizures. In addition, she reported experiencing auditory and visual hallucinations. She underwent dysautonomia evaluation and revealed a positive tilt table test. Other tests revealed unusual changes to her optic disks but without an elevated intraocular pressure, and nonspecific findings on her brain MRI, including a subtle focus of T2 signal abnormality involving the subcortical white matter of the right parietal lobe without associated enhancement. See Table 2 and Additional file 1: Table S6 for proband K10031-10133’s clinical phenotype list with HPO annotations, and Additional file 1: Supplemental Data for a full report of HPO analysis on her. Descriptions of video recordings (HDV_0079) of this proband illustrating her medical presentation and (HDV_0072) in which conditions in other family members are discussed are included in the supplemental videos section, and these videos can be provided on request to qualified investigators.

As for her family history (Additional file 1: Table S6), there are some noticeable symptoms that are shared by all her siblings and her mother, including dysautonomia-like symptoms such as dizziness and fainting, as well as tremors and asthma. In addition, anxiety, attention deficit, arthritis, dyslexia, gastroesophageal reflux, seizures and TS are other diagnoses found among her siblings. Her mother (K10031-10145), on the other hand, has HH and OCD traits. Her father has significant migraines, gastroesophageal reflux, hiatal hernia, and right sensorineural hearing loss. See detailed descriptions of her family members in Additional file 1: Supplemental Data. We are highlighting here that extensive characterization of families, including videotaping and the collection of collateral information from other relatives, yields a rich texture of findings that are not always easily captured in written medical records.

### Summary statistics of the WGS data

**Fig. 2:**
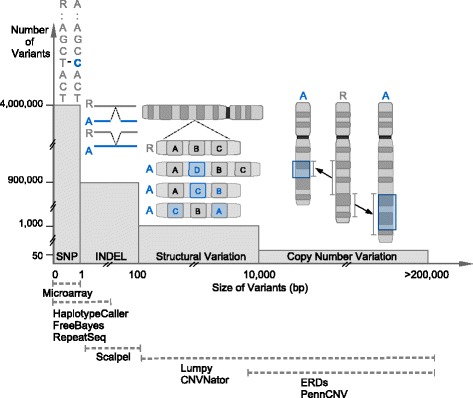
WGS can reveal a broad spectrum of variants with softwares that are specialized for different types of variants. This is a conceptual illustration of variations in the human genome. The Y-axis shows the approximate number of variants in that category while the X-axis shows the approximate size of those variants. The interval below shows that variants of different sizes and sequence compositions can be better detected by leveraging the strength of different callers. SNV: single-nucleotide variation, INDEL: insertions and deletion, SV: structural variant, CNV: copy number variant

We previously reported a large false negative rate with the Complete Genomics platform [56], so we chose to utilize the Illumina platform for WGS. Nine members of the family underwent WGS, enabling a wide scope of variant calling from a single SNV to large CNVs. To reduce false variant calls, more than one pipeline were used to detect SNVs, INDELs, large SVs, and CNVs, as we previously suggested [56] (Fig. 2). Summary statistics for the WGS data for each sample are reported in Additional file 1: Table S1, S2, and Fig. S2. The average number of reads per sample is 1,432,506,869. The number of mapped bases per sample is 124,410,724,287, with a mean coverage of the WGS data across the genome of about 40X (89% of the bases in the genome covered with at least 20X). The insert size of the libraries is about 338 and the GC content is approximately 40% across samples. With the WGS data, a mean number of 4,099,604 (SD = 47,076) SNVs, 896,253 (SD = 14327) INDELs, 1,284 (SD = 103) SVs, and 61 (SD = 4) CNVs are detected across nine samples (Additional file 1: Table S2). Within the coding regions, the average number of SNVs, INDELs, SVs, and CNVs detected are 22,406, 2,812, 511, 12, respectively. Kinship between individuals was inferred with KING to confirm family relationship between research participants in this study (Additional file 1: Table S3) [76].

### *WGS identified de novo CNV* deletions in 15q11.2 to 15q13.1 of proband K10031-10232

**Fig. 3:**
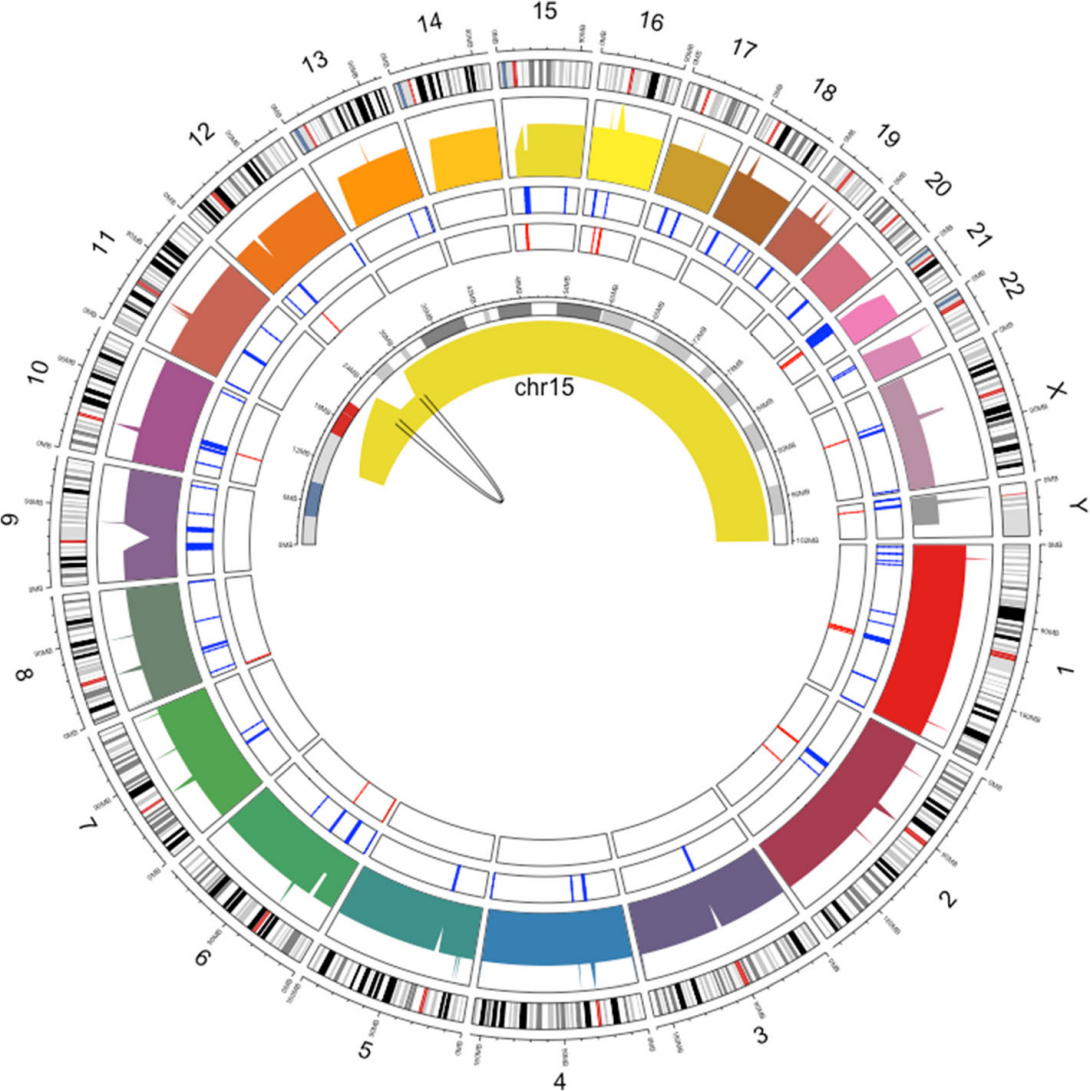
Circos plot of the PWS proband’s genome, highlighting chromosome 15. The outer circle is the cytoband of the human genome. The inner circle is the genome coverage of the PWS proband’s (K10031-10232) genome. The breakpoint of the 15q11.2-15q13 deletion region in chromosome 15 is illustrated in the center

**Fig. 4:**
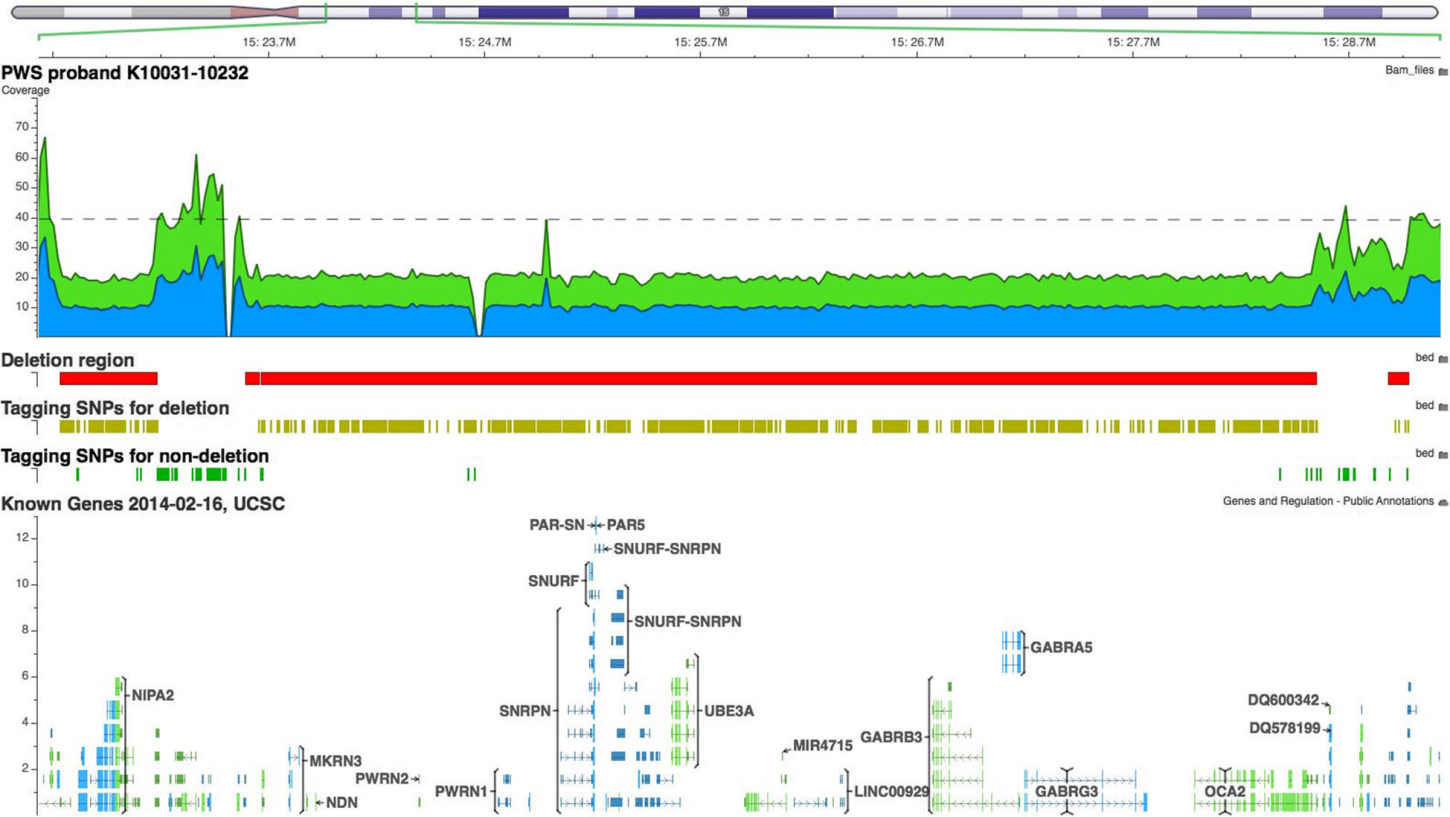
Screenshot of three heterozygous *de novo* deletions between the region 15q11.2 to 15q13 in proband K10031-10232. The deleted regions are denoted by the red boxes. The yellow tagging SNVs represent the SNVs that violate the Mendelian inheritance law. The non-deleted regions are denoted by the green tagging SNVs. Genome-wide average coverage (40X) is denoted by the grey dashed line. The breakpoints of these deletions (PWS Type I deletion) are chr15:22,749,401-23,198,800 (~449 Kb), chr15:23,608,601-28,566,000 (~4.96 Mb), and chr15:28,897,601-28,992,600 (95 Kb) (hg19). These deletions are not detected either in the proband’s father or the unaffected brother

ERDS and CNVnator both detected three *de novo* heterozygous deletions with a total size of about 5.5 Mb, in the chromosome regions from 15q11.2 to 15q13.1 of the proband with PWS (K10031-10232) (Fig. 3). The hg19 genomic coordinates of the breakpoints are chr15:22,749,401-23,198,800 (~449 Kb), chr15:23,608,601-28,566,000 (~4.96 Mb), and chr15:28,897,601-28,992,600 (~95 Kb). Notably, these deletions are relatively close to one another; the distances between each deletion are ~410 Kb and ~332 Kb, respectively. Within the regions containing the *de novo* deletions, the depth of coverage in the proband’s genome is 20X, about half of the genome-wide mean coverage (40X). Due to the lack of the proband’s mother’s sequencing data (as she refused to participate), analysis was performed to determine which allele (paternal or maternal) is deleted. This can be inferred through SNVs where the mendelian inheritance law is violated; meaning those instances in which the proband (K10031-10232) does not carry certain paternal or maternal SNVs that his brother (K10031-10233) does carry. In total, there are 2,987 SNVs where the proband’s father (K10031-10231) is a homozygote and the proband’s brother is a heterozygote. Out of the 2112 SNVs where the father is homozygous to the reference allele, the proband is homozygous to the alternative allele at 1944 loci (92%, Fig. 4). Among 875 SNVs where the father does not carry any reference allele, the proband carries only the reference allele at 861 SNVs (94%, Fig. 4). This indicates that the proband only carries the maternal alleles in those regions. These deletions were not detected in either the proband’s father or his brother using the WGS data (Additional file 1: Fig. S3). The Illumina microarray data further confirmed this discovery; the proband carries these deletions (Additional file 1: Fig. S4) while his father and his brothers (K10031-10233 and K10031-10234) do not carry any of these deletions in their genome (Additional file 1: Fig. S4-S6). Probe distributions of Log-R ratios and B allele frequencies are not uniform in the microarray because the density of SNV varies between genomic regions (Additional file 1: Fig. S4-S7). This highlights the higher resolution and completeness of WGS over microarray for precise molecular diagnosis of such diseases. Thus, we confirm that the proband carries the *de novo* PWS Type I deletion (spanning breakpoints BP1 and BP3) defined by previous publications [77, 78]. The complete list of genes that fall into the deletion regions are described in Additional file 1: Table S7.

### Phenolyzer discovered interaction between PWS deletions and disease subtypes

**Fig. 5:**
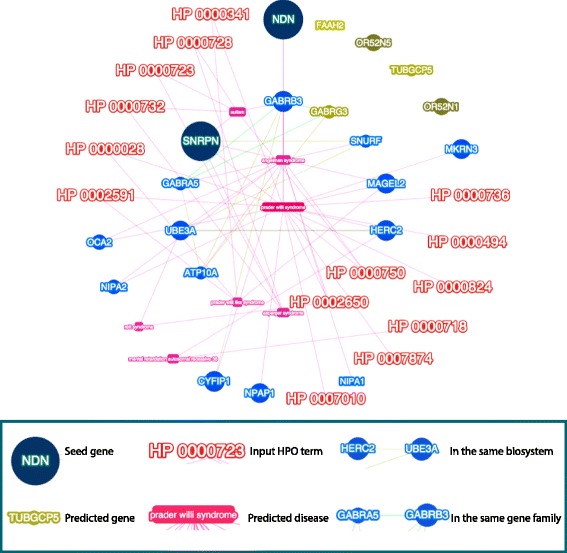
Phenolyzer networks analysis of WGS gene findings, HPO terms, and diseases types. Phenolyzer revealed the diagnosis of PWS and how genes in the deletion regions are linked towards the phenotypes represented by HPO terms. The most disease relevant genes are showed as seed genes, alongside with predicted genes in the deletion regions. Yellow lines indicate that the two node genes are within the same biosystem, while green lines indicate that the two genes are within the same gene family

Phenolyzer accurately revealed the diagnosis of PWS and how genes in the deletion regions are linked towards the phenotypes represented by HPO terms. The Phenolyzer network analysis of gene findings, HPO terms, and diseases types are shown in Fig. 5. The most disease relevant genes are showed as seed genes. Among all genes in the deletion regions, *SNRPN*, *NDN* and thirteen other genes are the most confident genes, which are maternally imprinted and commonly deleted in PWS [79, 80]. Further, yellow lines indicate that the two node genes are within the same biosystem while green lines indicate that the two genes are within the same gene family. Phenolyzer reports two clusters of genes that are in the same Biosystem. The first cluster includes *SNRPN*, *OR52N5*, *SNUPF*, *UBE3A*, *HERC2*. The second cluster consists of *GABRG3*, *GABRB3*, *GABRB5*, *ATP10A*, while the former three genes are also from the same gene family.

**Fig. 6:**
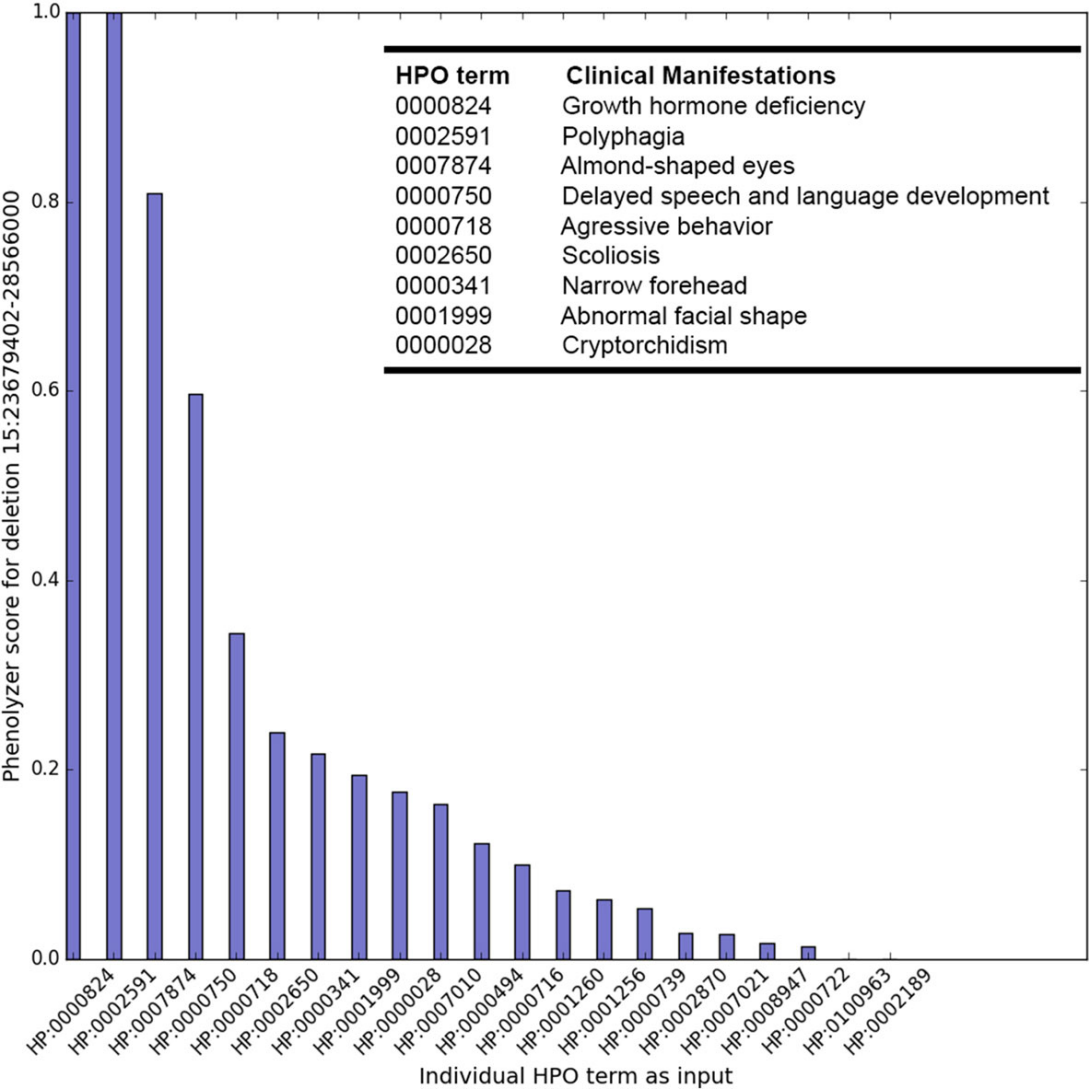
Phenolyzer scores represent the importance of each HPO term for PWS diagnosis. Phenolyzer scores are normalized to a range from 0 to 1. A higher score indicates that this HPO term has a higher impact on the diagnosis of the corresponding disease. The clinical manifestations of the top HPO terms are shown in the figure legend

Phenolyzer mapped nine HPO terms to PWS, including HP:0002591 (polyphagia), HP:0000824 (growth hormone deficiency), and HP:0007874 (almond shaped eyes). It is shown that the combination of the above three terms largely determine the diagnosis of PWS (Fig. 6). Phenolyzer score of the correct deletion corresponding to each HPO term is normalized to a range from 0 to 1. A higher score indicates that this HPO term has a higher impact on the diagnosis of the corresponding disease. Both ‘polyphagia’ and ‘growth hormone deficiency’ have a Phenolyzer score of 1.0 and ‘almond shaped eyes’ has a score of 0.8. Phenolyzer also reveals the molecular and phenotypic similarities between PWS and its related diseases. For example, Phenolyzer reports that Angelman Symdrome (AS) shares three HPO terms and eleven genes. Among those genes, *UBE3A* has been implicated in AS due to a loss of gene expression from maternal chromosome [81]. Three overlapping phenotypic features are also discovered, including HP:0002650 (Scoliosis), HP:0000750 (Delayed speech and language development), and HP:0001999 (Abnormal facial shape).

**Fig. 7:**
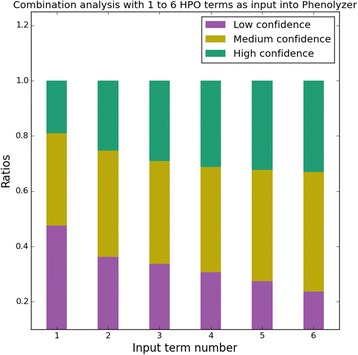
Combination analysis indicates using more HPO terms lead to a higher chance of the correct prioritization. ‘High confidence’, where the known deletion has a normalized Phenolyzer score no less than 0.5; ‘Medium confidence’, where the known deletion has a normalized Phenolyzer score between 0.1 and 0.5; ‘Low confidence’, where the known deletion has a normalized Phenolyzer score less than 0.1

Another interesting question is whether the number of input HPO terms used will impact the final result. To answer this, we conducted a series of combination analyses with one to six HPO terms out of the 21 candidate terms as input into Phenolyzer, respectively. We noticed that the more candidate terms we used, the more combinations we have. For example, the number of combinations for six out of 21 is 54,264. Thus, due to the large overhead of computation time, we did not go beyond six terms. From the result, the more HPO terms we used as input, the higher the chance the known CNV was prioritized as ‘High confidence’ (Fig. 7).

### Phenolyzer revealed the relationship between p.C282Y variant and HH in individual K10031-10145, which was missed by HPO analysis alone

**Fig. 8:**
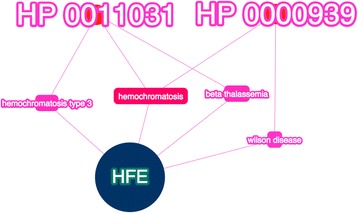
Phenolyzer networks analysis of both HPO and WGS data yielded the correct diagnosis for the individual with HH (K10031-10145). Phenolyzer successfully linked the gene *HFE* to two HPO terms (HP:0011031 Abnormality of iron homeostasis, HP:0000939 Osteoporosis), bridged by the predictive diagnosis, hemochromatosis. The most disease relevant gene *HFE*, is showed as a seed gene (*blue*)

The mother (K10031-10145) with HH is homozygous for the p.C282Y variant in HFE, which is consistent with her molecular genetic assay results. Analyzing the HPO data with Phenomizer alone failed to suggest the diagnosis of HH (Additional file 1: Supplemental Data). This is likely because HH has many clinical features overlaping with other diseases. Even the most specific HPO term, HP:0011031 (abnormality of iron homeostasis), still links to 14 diseases and 9 genes. However, we were able to recover this finding when we used Phenolyzer to compare the patient’s genomic and phenotypic profile. Phenolyzer pinpointed the only two out of eight input HPO terms which are indeed related to hemochromatosis, although these two terms are also reported with other diseases. As shown in Fig. 8, Phenolyzer successfully related the input HPO terms to hemochromatosis, as well as the input gene *HFE*. Because this was the most confident finding, the diagnosis of hemochromatosis was thus recommeneded by Phenolyzer.

Results from analyzing the WGS data showed that the mother’s brother (K10031-10231) is also homozygous for the p.C282Y variant in HFE. However, his clinical test result has not yet provided any evidence to support the diagnosis of HH, even though male p.C282Y homozygotes are considered more likely to develop iron-overload–related diseases due to the lack of the iron clearance events like menstruation and pregnancy in women [82]. This is in line with the fact that even family members can have variable expressivity of disease, including different onset ages, etc. This instance with the brother and the sister again highlights the point that the phenotypic expression of a given mutation in *HFE* may vary widely, influenced in part by unidentified modifier loci [83–89]. Some studies previously estimated that less than 1% of individuals in the U.S. carrying homozygous mutations present clearly with clinical diagnoses of hemochromatosis [90]. In contrast to studies that have searched for the “causal” gene, some have reported that genetic variations can instead have large effects on phenotypic variability, suggesting underlying genomic complexity from multiple interacting loci [91–94]. Understanding such diseases thus requires probabilistic thinking about the risk of developing the clinical manifestation, rather than deterministic genotype-phenotype “causation” [16, 95–97], and there will always be some level of stochasticity as well [20]. Further, alongside the primary research-focused analysis, the participating subjects and families also received the research findings (Additional file 1: Table S8). Of course, we cannot exclude the possibility that we might have missed some variants, including possibly non-coding variants, and we expect that the future phenotyping, sequencing, and collation of data from millions of people will reveal associations that are not currently known.

### Analysis of dysautonomia-like symptoms

None of the family members with dysautonomia-like symptoms carry any previously reported variants in *IKBKAP* that are implicated in the autosomal recessive transmission of FD, which is also called hereditary sensory and autonomic neuropathy type III (HSAN-III). The WGS data have effective sequence coverage (> average coverage 40X) for this gene, but no novel rare variants were identified. Notably, both the mother (K10031-10145) and the male proband (K10031-10138) carry heterozygous variants of p.H604Y and p.G613V in the protein product of *NTRK1*, which has been proven to contribute to HSAN-IV (congenital insensitivity to pain with anhidrosis). HSAN-IV is a disease closely resembling FD (HSAN III), and is characterized by a lack of pain sensation, anhidrosis, unexplained fever since childhood, and self-mutilating behavior [98, 99]. Both variants are located within the intracellular tyrosine kinase domain of the encoded protein, but neither sites are conserved. Both variants have also been reported before in healthy individuals, so they are considered to be polymorphisms in the population and seem to be in linkage disequilibrium [100–104]. The mother’s brother (K10031-10231, unaffected) also carries these two variants, so this provides further evidence that they are likely to be polymorphisms. Most importantly, neither variant is present in the proband K10031-10133, who reported the most severe dysautonomia-like symptoms.

Instead of the *NTRK1* variant, a manual filtering found seven other putative variants in *PLCG2*, *ATXN2*, *VWA8*, *LRRIQ1*, *MYO1H*, *OR1J4*, and *RFX4* which follow a dominant inheritance model (Additional file 1: Table S4). Variants in *PLCG2*, *ATXN2*, and *VWA8* were previously reported to be associated with certain disease phenotypes, including cold-induced urticaria, antibody deficiency, susceptibility to infection and autoimmunity, spinocerebellar ataxia type 2, celiac disease, and susceptibility to amyotrophic lateral sclerosis [98–100]. However, the variants we identified in this family are not the same variants in the literature, and all of these predicted diseases have only partially overlapping manifestations with dysautonomia-like symptoms. For the rest of the four genes mentioned above, *LRRIQ1*, *MYO1H*, *OR1J4*, and *RFX4,* there has been, to our knowledge, no reports published to date discussing any variants in these genes associated with human disease. Therefore, the functional impact of these variants remains unclear.

Lastly, Phenolyzer analysis was carried out using the phenotype of proband K10031-10133 as input. It successfully prioritized two variants we identified in the manual filtering analysis discussed above, *ATXN2* and *VWA8*, and further revealed the complexity of such diseases (Additional file 1: Fig. S8).

These results together suggest that the genetic inheritance of dysautonomia-like symptoms in this pedigree may not consist of only one high-effect size mutation, but rather could be polygenic and/or environmentally influenced. It is possible that multiple variants including those we mentioned above are acting together or in conjunction with modifiers in these individuals’ genomes to give rise to a spectrum of complex clinical manifestations.

## Conclusions

This research report provides insights into using WGS as a genetic test to investigate PWS and other phenotypes. In our study, three *de novo* deletions were discovered at single base pair resolution. WGS enables the reconstruction of the recombination event in this imprinting hotspot 15q11-13, which provides deeper insights into the mechanism of PWS. Notably, this is the first report of an Illumina HiSeq WGS experiment on an individual with PWS with the paternal allele deletion. In principle, the use of WGS, once standardized, could eventually simplify the molecular diagnosis procedure for PWS and and other genetic syndrome patients, as one would no longer need to through the several steps for the standard genetic testing for PWS [77, 105, 106]. Since AS and PWS share a similar cytogenetic anomaly in 15q11.2 to 15q13 [107, 108], WGS could potentially help reveal the sub-types of both syndromes, given that the breakpoints of the CNVs can be mapped at the nucleotide level and one could distinguish which allele (paternal or maternal) has been deleted. However, WGS alone would not be enough to detect either uniparental paternal disomy with heterodisomy or imprinting defects in this genomic region for non-deleted PWS individuals [77, 109].

However, WGS might not always pinpoint the exact disease relevant variants, due to the limition of cohort size and disease complexity. Phenotype and genotype matching across cohorts is needed for confirming causal relationships. HPO has emerged as a standardized way to compare phenotypes, although it can only marginally solve the phenotype issue and cannot be directly used for WGS analysis. Fortunately, the development of phenotype-analysis tools such as Phenolyzer makes it possible to bridge the gap between the two on top of rich prior information across multiple databases. During the selection process for a particular patient’s features, one is able to query a surplus of clinical and scientific knowledge about the diseases linked to the feature in question. In addition, integration of four types of gene-gene interaction databases in Phenolyzer makes it possible to find more candidate genes beyond the existing gene-disease knowledge and generate new biological hypotheses. While the common drawback of all the gene prediction tools is the balance between sensitivity and specificity, Phenolyzer uses a modified logistic regression model to address this problem, ensuring that well-established genes are recommended among a large set of predictions.

This report about integrating WGS and HPO data demonstrates the effectiveness of such an approach and shows its potential for clinical implementation. Neither technique on its own is ideal for clinical diagnosis, but fortunately they complement each other and thus help eliminate false positives and reveal novel insights into human diseases. The potential for HPO remains in the development of a more multi-dimensional depiction of subjects that takes into account the past and present human presentation, and will aid in efforts for early diagnoses and intervention. As the field of medical genetics advances, researchers will need to find an efficient way to capture phenotypic information that allows for the use of computational algorithms to search for phenotypic similarity between genomics studies [36]. For WGS, with ever-increasing sequencing capacity, a scalable and reliable informatic solution is key to analyzing millions of genomes simultaneously. To maximize this potential in clinical settings, data from WGS and HPO should be integrated and shared in a unified fashion.

## Additional files

Additional file 1: Supplemental Tables and Figures. This file includes supplemental figures S1-S8, tables S1-S10, pharmacogenomic analyses for individual K10031-10133, additional clinical information of individuals in the study, and video descriptions. (PDF 1491 kb)
Additional file 2: Tute Genomics Report. This file contains nine Tute Genomics reports of all participants included in the study. (PDF 720 kb)
Additional file 3: Phenomizer Diagnosis Report. This file contains three Phenomizer diagnosis reports of individual K10031-10232, K10031-10133, and K10031-10145. (PDF 429 kb)

## Abbreviations

bp: Base pair
CIPA: Congenital insensitivity to pain with anhidrosis
CNV: Copy number variation
FD: Familial dysautonomia
HH: Hereditary hemochromatosis
HPO: The human phenotype ontology
INDELs: Insertions and deletions
Kb: Kilo base pairs
Mb: Megabase pairs
NGS: Next-generation sequencing
OCD: Obsessive-compulsive disorder
PCR: Polymerase chain reaction
PWS: Prader–Willi syndrome
SNV: Single-nucleotide variation
SV: Structual variant
TS: Tourette syndrome
WES: Whole exome sequencing
WGS: Whole genome sequencing
