# Supplementary material for: Whole genome sequencing of one complex pedigree illustrates challenges with genomic medicine
Source: BMC Med Genomics. 2017 Feb 23;10:10. doi: 10.1186/s12920-017-0246-5 (PMC5322674; doi:10.1186/s12920-017-0246-5)
Supplement: Additional file 1: — Supplemental Tables and Figures. This file includes supplemental figures S1-S8, tables S1-S10, pharmacogenomic analyses for individual K10031-10133, additional clinical information of individuals in the study, and video descriptions. (PDF 1491 kb) [file 12920_2017_246_MOESM1_ESM.pdf]

Supplemental Note

Table S1 Summary statistics of the whole genome sequencing data generated in this study

Sample in K10031	Number of reads	Percentage mapped, paired	# of mapped bases	Mean coverage	Fraction covered >20X	Median insert size	Mean mapping quality	GC content
10133	1,762,018,294	99.21%, 99.21%	171,859,247,896	54.78X	90.16%	320	48.08	39.31%
10138	1,413,986,946	99.36%, 99.36%	138,460,912,489	44.14X	89.31%	323	48.17	39.73%
10143	1,627,071,352	77.51%, 77.51%	125,253,967,485	39.93X	89.66%	362	48.15	40.09%
10144	1,964,930,229	68.61%, 68.61%	133,744,891,260	42.63X	89.05%	361	48.21	40.08%
10145	1,213,529,960	98.51%, 98.51%	118,888,431,070	37.9X	89.3%	362	48.2	39.92%
10231	1,204,598,812	98.12%, 98.12%	117,678,296,174	37.51X	87.54%	331	48.19	40.06%
10232	1,206,286,495	98.35%, 98.35%	118,169,799,321	37.67X	87.58%	328	48.2	40.14%
10233	1,215,246,598	98.3%, 98.3%	118,899,663,206	37.9X	87.83%	328	48.2	40.34%
10235	1,614,404,561	77.26%, 77.26%	124,189,833,293	39.59X	89.7%	329	48.11	40.62%

Table S2 Summary statistics of the variants detected from each individual in this study

Sample in K10031	# of SNPs	# of exonic SNPs	# of INDELs	# of exonic INDELs	# of SVs	# of exonic SVs	# of CNVs	# of exonic CNVs
10133	4,091,673	22,207	900,734	2,791	1,438	587	64	11
10138	4,169,002	22,909	894,680	2,837	1,439	555	62	13
10143	4,177,966	23,127	890,519	2,807	1,274	501	61	11
10144	4,036,600	22,060	864,093	2,830	1,349	531	59	8
10145	4,070,931	22,288	900,906	2,816	1,229	495	63	14
10231	4,103,856	22,153	900,027	2,717	1,146	443	64	15
10232	4,060,508	21,902	892,087	2,838	1,209	469	66	13
10233	4,081,064	22,550	915,298	2,826	1,224	495	59	12
10235	4,104,834	22,456	907,935	2,843	1,248	525	54	12
**Average**	4,099,604	22,406	896,253	2,812	1,284	511	61	12

SD	47,076	401	14,327	39	103	44	4	2

Table S3 Kinship inference between each pair of the individuals using KING

FID1	FID2	Number_SNP	HetHet	IBS0	Kinship
K10031_10144	K10031_10145	7712483	0.155	0.061	0.0377
K10031_10133	K10031_10144	7720872	0.204	0.005	0.2459
K10031_10133	K10031_10145	7719024	0.201	0.0051	0.2478
K10031_10138	K10031_10144	7721400	0.205	0.0052	0.2478
K10031_10138	K10031_10145	7720609	0.2	0.0054	0.2458
K10031_10143	K10031_10144	7719412	0.207	0.0041	0.2527
K10031_10143	K10031_10145	7721142	0.206	0.0041	0.252
K10031_10235	K10031_10144	7715502	0.207	0.004	0.2523
K10031_10235	K10031_10145	7716964	0.201	0.0041	0.2488
K10031_10231	K10031_10145	7712110	0.224	0.0197	0.2398
K10031_10231	K10031_10232	7709621	0.202	0.0049	0.2321
K10031_10231	K10031_10233	7710754	0.203	0.0045	0.2336

A kinship score of 0.25 indicates that these two individuals are parent-child or full siblings.

Table S4 A list of variants (AAF<1% in ExAC and 1000G) found in all sequenced individuals with dysautonomia-like symptoms

Gene	Genomic coordinates	Change	Effect	CADD	Zygosity & AAF
ATXN2	chr12: 112037180	G>T	missense	7.4	Het, None
VWA8	chr13: 42361611	ATCAG>A	frameshift	-	Het, 0.01%
LRRIQ1	chr12: 85450243	G>A	missense	10.2	Het, 0.37%
MYO1H	chr12: 109862622	A>G	missense	19.5	Het, 0.002%
OR1J4	chr9: 125281999	C>T	missense	7.4	Het, 0.62%
PLCG2	chr16: 81927314	G>A	splice_region	10.3	Het, 0.1%
RFX4	chr12: 107155117	C>T	missense	16.67	Het, 0.68%

The coordinates are shown with respect to hg19. These variants are reported in all three sequenced individuals affected by dysautonomia-like symptoms (K10031-10133, 10138, and 10145), called by at least one pipeline, located within coding regions, are ranked high by

pVAAST, and have a CADD c-score greater than 15 or have a GEMINI predicted of at least MED. The Alternative Allele Frequency (AAF) is computed based on the general population in the ExAC database or 1000G for some intronic variants.

Table S5 Complete clinical presentation of proband K10031-10232

**General Information**	
Age (years)	25
Gender	Male
**Clinical Manifestations**	**HPO#**
Prenatal History	
Cesarean section	0011410
Gestational diabetes	0009800
Oligohydramnios	0001562
Premature birth (36 weeks gestation)	0001622
Development and Growth	
Delayed speech and language development	0000750
Dysarthria	0001260
Growth hormone deficiency	0000824
Poor fine motor coordination	0007010
Mild intellectual disability	0001256
Facial Features	
Abnormal facial shape	0001999
Almond-shaped eyes	0007874
Downslanted palpebral fissures	0000494
Narrow forehead	0000341
Other Physical Features	
Cryptorchidism	0000028
Excessive daytime sleepiness	0002189
Infantile muscular hypotonia	0008947
Obstructive sleep apnea syndrome	0002870
Scoliosis	0002650
Behavior Features	
Aggressive behavior	0000718
Anxiety	0000739
Depression	0000716
Flipping pages in books	NL
Hair-pulling	0012167
Hyperesthesia	0100963
Impaired ability to form peer relationships	0000728
Impaired social reciprocity	0012760
Inflexible adherence to routines or rituals	0000732
Irritability	0000737
Lack of insight	0000757
Lack of peer relationships	0002332
Low frustration tolerance	0000744
Nail-biting	0012170
Obsessive-compulsive disorder	0000722
Pain insensitivity	0007021
Polyphagia	0002591
Poor eye contact	0000817
Restrictive behavior	0000723
Short attention span	0000736
Skin-picking	0012166

Abbreviations: BMI= body mass index, NL= not listed.

Table S6 Complete clinical presentation of proband K10031-10133's family

Pedigree K10031		10133	10145	10235	10261	10138	10143
**General Information**							
Age (years)		26	45	24	22	20	18
Gender		F	F	F	M	M	F
**Clinical Manifestations**	**HPO#**						
Developmental/Growth							
Dyslexia	0010522	-	-	-	+	-	-
Cardiovascular							
Bradycardia	0001662	+	-	-	-	+	-
PFO	0001655	+	-	-	-	-	-
Syncope	0001279	+	+	-	+	+	-
Tachycardia	0001649	+	+	-	-	-	-
Eyes							
Astigmatism	0000483	+	-	-	-	-	-
Diplopia	0000651	+	-	-	-	-	-
Myopia	0000545	+	-	-	-	-	-
Optic disks changes	NF	+	-	-	-	-	-
Peripheral vision	NF	+	-	-	-	-	-
Prominence to the eyes	NF	+	+	+	+	+	+
Gastrointestinal							
Acid reflux	0002020	-	-	+	-	-	-
Anorexia	0002039	-	+	-	-	-	-
Gastroparesis	0002578	+	-	-	-	-	-
Nausea	0002018	+	-	-	-	-	-
Gynecologic & Genitourinary							
Irregular periods	NF	+	-	-	NA	NA	-
Urinary retention	0000016	+	-	-	-	-	-
Urinary incontinence	0000020	+	-	-	-	-	-
Hematologic/Lymphatic/Immunologic							
Adenopathy	Lymphoadenopathy*: 0002716	+	-	-	-	-	-
Musculoskeletal							
Arthralgia	0002829	+	-	-	-	-	-
Arthritis	0001369	-	-	-	-	-	+
Cyanotic lower extremities	0001063	+	-	-	-	-	-
Joint Stiffness	0001387	+	-	-	-	-	-
Muscle weakness	0001324	+	-	-	-	-	-
Osteoporosis	0000939	-	+	-	-	-	-
Neurological							
Apraxia	0002186	+	NK	NK	NK	NK	NK
Arthritis	0001369	+	-	-	+	-	+
Auditory hallucinations	0008765	+	-	-	-	-	-
Concussion	NF	+	-	-	-	-	-
Convulsions	NF	+	-	-	-	-	-
Dizziness	NF	+	+	-	+	-	+
Dysarthria	0001260	+	-	-	-	-	-
Fatigue	0012378	+	+	-	-	-	-
Frequent falls	0002359	+	-	-	-	+	-
Headache	0002315	+	NK	NK	NK	NK	NK
Heat intolerance	0002046	+	+	-	-	-	-
Ischemic stroke	0002140	+	-	-	-	-	-
Migraine	0002076	+	-	+	+	+	+

Numbness	Paresthesia*: 0003401	+	-	-	-	-	-
Seizure	0001250	+	-	-	+	-	-
Tremor	0001337	+	+	+	+	+	+
Visual hallucinations	0002367	+	-	-	-	-	-
Respiratory							
Asthma	0002099	+	+	+	+	+	+
Dyspnea	0002094	+	NK	NK	NK	NK	NK
Psychiatric							
ADHD	0007018	-	-	-	+	+	-
Anxiety	0000739	+	+	+	-	+	+
Depression	0000716	+	+	-	-	-	-
Dissociative identity disorder	NF	-	+	-	-	-	-
Obsessive-compulsive behavior	0000722	-	+	-	-	+	-
Tourette syndrome	ORPHANET:856	-	-	-	-	+	-

Abbreviations: NA= not applicable, NF= not found, NK= not known. Terms with star refer to the synonym of the disease or feature in question as categorized in the HPO Phenomizer tool.

Table S7 A list of genes that fall into the deletion regions

Gene category	Genes
**Protein coding genes**	*TUBGCP5, CYFIP1, NIPA2, NIPA1, MKRN3, MAGEL2, NDN, NPAP1, SNRPN, SNURF, SNURF-SNRPN, HBT8, C15ORF49, UBE3A, ATP10A, GABRB3, GABRB5, GABRG3, OCA2, HERC2*
**Pseudogenes**	*GOLGA8S, GLOGA8EP, GOLGA6L2, GOLGA8IP, WHAMMP3* (partially deleted, exon 1 to exon 2), *HERC2P2, AK124131, AX747189, AK124673, HERC2P9, WHAMMP2* (partially deleted, exon 1 to exon 5)
**microRNAs**	*MIR4508, MIR4715*
**piRNAs**	*DQ600342, DQ582939, DQ578838, DQ588973, DQ595055, DQ572979, DQ582073, DQ594309, DQ595648, DQ578199, DQ596685, DQ582448, DQ593032, DQ588687, DQ597560*
**snoRNAs**	*SNORD109B, SNORD116- (1, 2, 3, 4, 5, 8, 10, 11, 12, 13, 15, 16, 18, 19, 20, 22, 24, 25, 26, 28, 29), SNORD115- (1, 2, 3, 4, 5, 6, 7, 8, 9, 10, 11, 13, 14, 15, 16, 17, 22, 24, 25, 26, 27, 28, 30, 31, 32, 33, 35, 37, 38, 39, 40, 41, 44, 45, 47, 48)*
**Non-coding RNAs (ncRNA)**	*PWRN2, PWRN1, PWARSN, PWAR1, PWAR5, LINC00929, LOC100128714, LOC283685, LOC440243, LOC283683*
**tRNA**	*tRNA_Glu*
**Unassigned RNA**	*JB175342*

The break points are chr15:22,749,401-23,198,800, chr15:23,608,601-28,566,000, and chr15:28,897,601-28,992,600**.** The coordinates are shown with respect to hg19. All genes are heterozygous for the deletion. The coordinates for the non-deleted region is chr15: 23383801-23679100. The genes that fall in this non-deleted region are mostly pseudogenes, small RNAs, and long non-coding RNA (lncRNA) genes, including *WHAMMP3* (partially deleted, exon 1 to exon 5), *GOLGA8IP, DQ600342, DQ582939, DQ578838, DQ588973, DQ595055, DQ572979,*

JB175342, HERC2P2, DQ582073, LOC440243, DQ594309, DQ595648, GLOGA8EP, DQ600342, DQ578838, DQ572979, JB175342, LOC440243, DQ582073, DQ595648, GOLGA8S, and *DQ578199*.

Table S8 A list of variants with previous evidence in ClinVar found in the pedigree members

Gene	Genomic coordinates	Change & variant type	Zygosity & Carriers	AAF	Relevant diseases & Inheritance
HFE	chr6: 26093141	G>A missense	hom: 10145, 10231<br>het: 10232, 10233, 10133, 10235, 10138, 10143	0.007%	hereditary hemochromatosis -AR
BRIP1	chr17: 59937223	G>C missense	het: 10231, 10145, 10133, 10235, 10138	0.04%	breast cancer, early-onset - AR
MKKS	chr20: 10393439	G>T missense	het: 10133, 10145, 10143	0.9%	Mckusick-Kaufman syndrome - AR
PRSS1	chr7: 142458451	A>T missense	CH: 10231, 10232, 10145, 10143, 10133, 10235, 10138	47%	hereditary pancreatitis - CH

Abbreviations: AAF= Alternate Allele Frequency in ExAC database, hom= homozygous, het= heterozygous, AR= Autosomal recessive, CH= compound heterozygous. The coordinates are shown with respect to hg19. The mother with hereditary hemochromatosis is homozygous for the p.C282Y variant in HFE. The carriers are represented by the numbers shown in Fig 2.

Table S9 Recommended dosages for warfarin and simvastatin dosages based on the individual K10031-10133's SNV results from the WGS data in comparison to what she was actually prescribed in the absence of any genetic testing

Drug	Recommend dosages based on genotypes	Previous prescriptions	FDA recommendations	Genotypes
**Warfarin**	5.85 mg/day	5 mg/day	2 to 10 mg/day (consider genetic testing results)	*VKORC1*: A/G (rs9923231)<br>*CYP2C9*: *1/*1 (rs1799853, rs1057910)
**Simvastatin**	20 mg/day increased risk of myopathy with 40mg Simvastatin	20 mg/day	80 mg/day	*SLCO1B1*: T/C (rs4149056)

Pharmacogenomics analyses were performed based on guidelines and algorithms from the International Warfarin Pharmarcogenomics Consortium (IWPC) and the Clinical Pharmacogenomics Implementation Consortium (CPIC) in the PharmGKB database. People who are homozygous for major alleles at both sites in *CYP2C9* are designated as *1/*1.

Table S10 HPO presentation of proband K10031-10145

Clinical Manifestations	HPO Number
Hypotension	0002615
Abnormality of iron homeostasis	0011031
Osteoporosis	0000939
Mood swings	0000720
Anxiety	0000739
Syncope	0001279
Obsessive-compulsive behavior	0000722
Heat intolerance	0002046

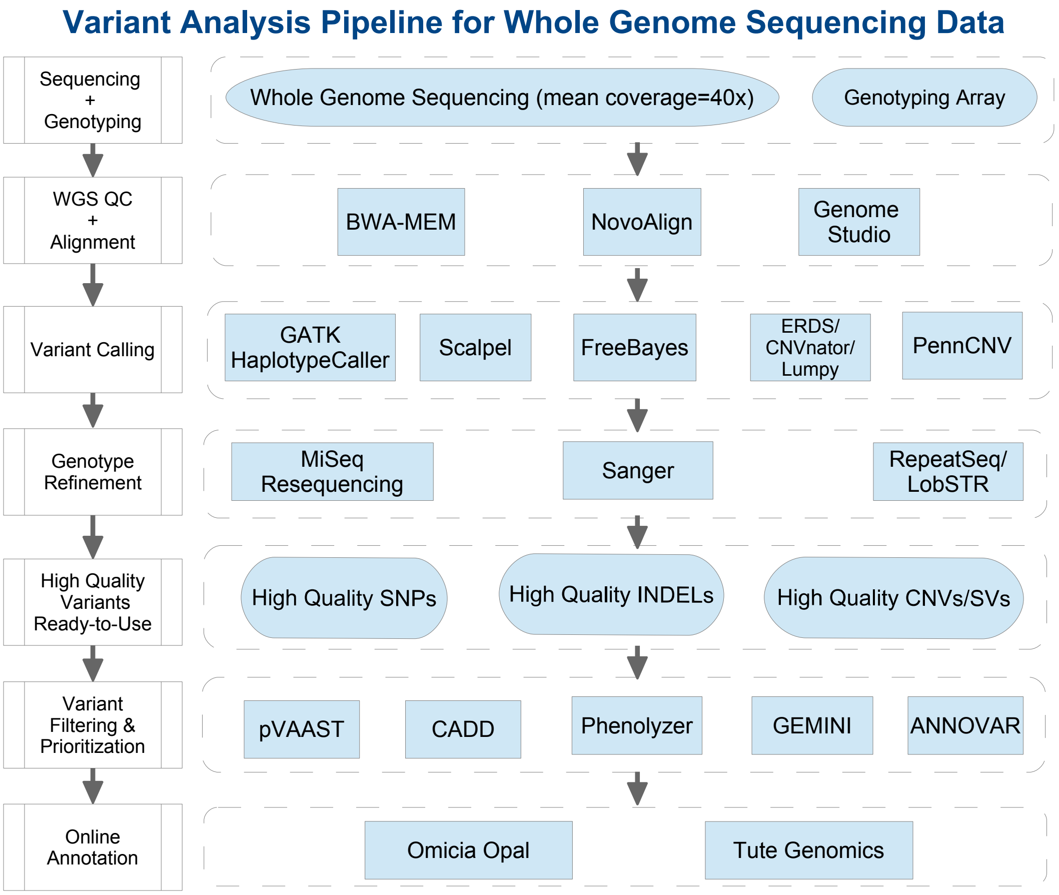

Fig. S1 Variant analysis pipeline for whole genome sequencing data and the microarray data. The left-hand side shows the major analysis work-flow while the right-hand side shows the details of each procedure.

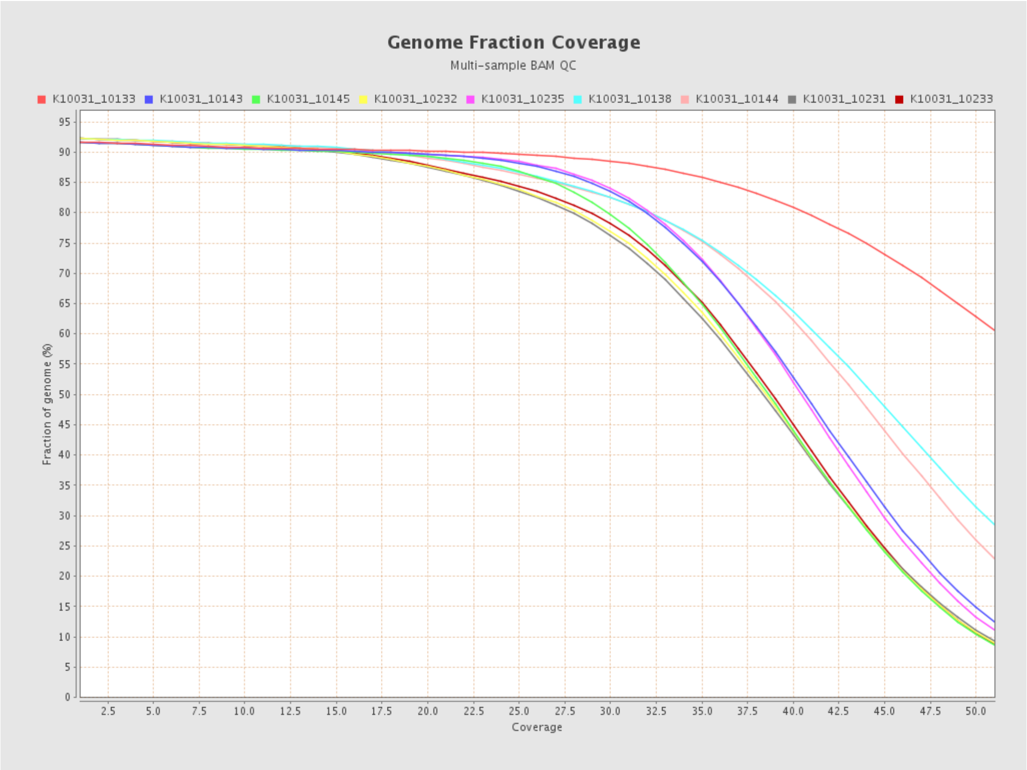

Fig. S2 Genome fraction coverage distributions of the sequencing data. Each curve represents one genome in this study. For each sample, more than 90% of the genome is covered with about 20 reads.

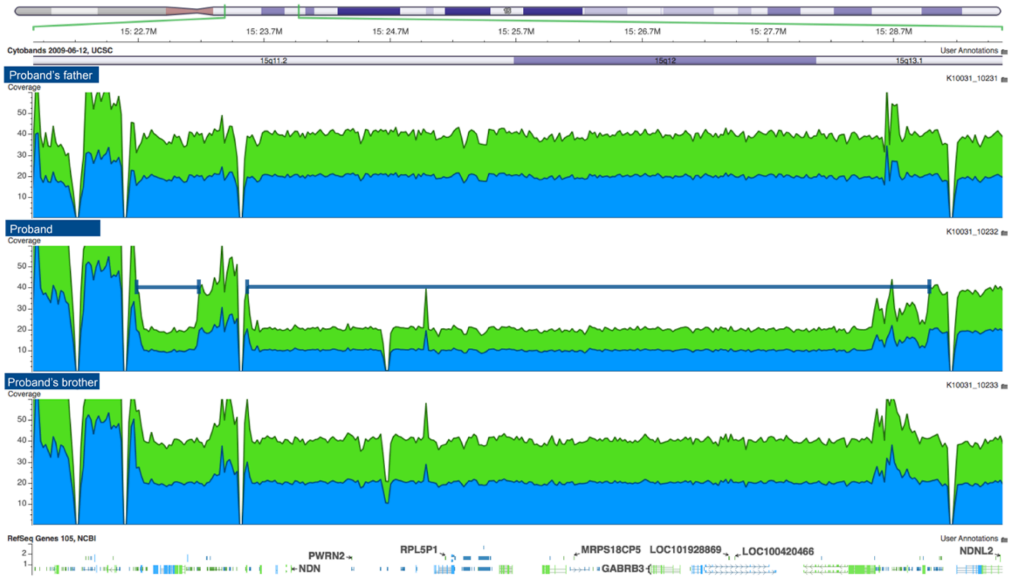

Fig. S3 Screenshot of the alignment in 15q11.2-15q13. These deletions are not detected either in the proband's father (K10031-10231) or the unaffected brother (K10031-10233). The deletion was confirmed with the Illumina Omni 2.5m microarray data.

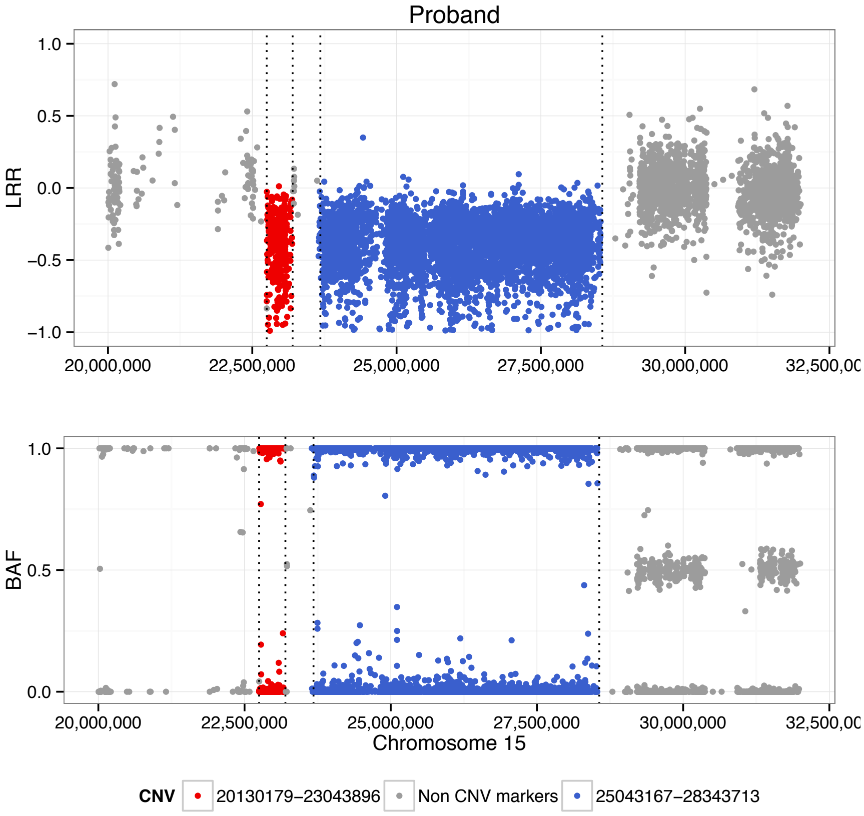

Fig. S4 Validation of the copy number variant in the proband with PWS (K10031-10232) using Illumina 2.5m microarray data. We used PennCNV to call these deletions from the microarray data, which is also only detected from the proband, but not from the father and the two unaffected brothers. The dash lines in the figure of proband indicate the interval of the ERDS copy number variant call.

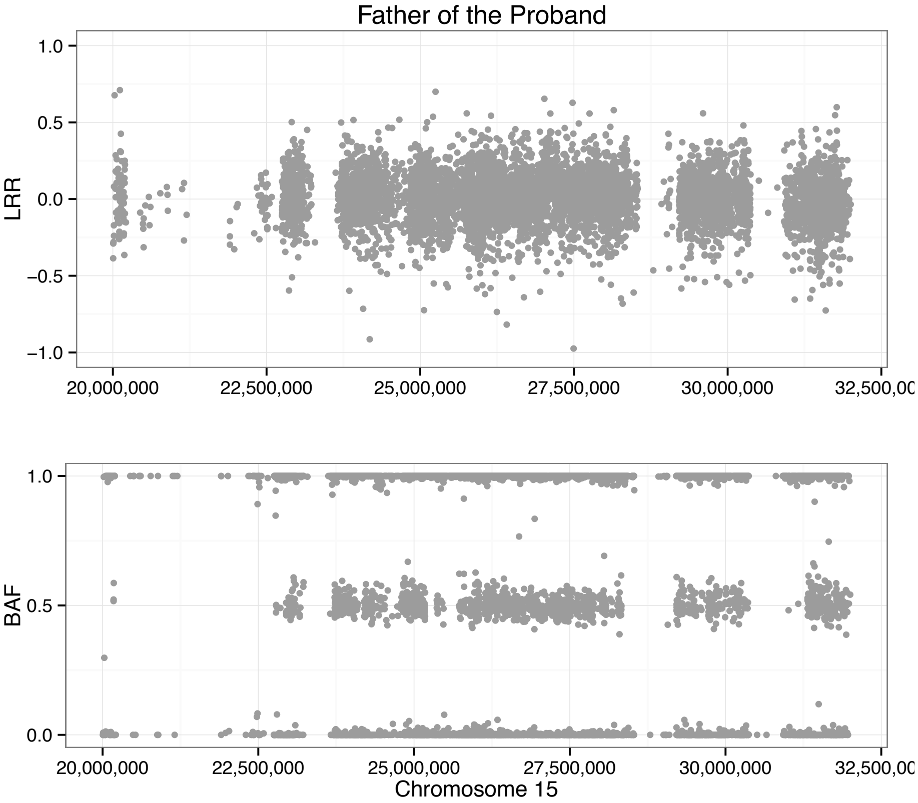

Fig. S5 Distributions of Log-R ratios (LRR) and B allele frequencies (BAF) in 15q11.2-15q13 of the microrarray data from the father of the proband (K10031-10231) with PWS. We used PennCNV to inspect this region from Illumina 2.5m microarray data.

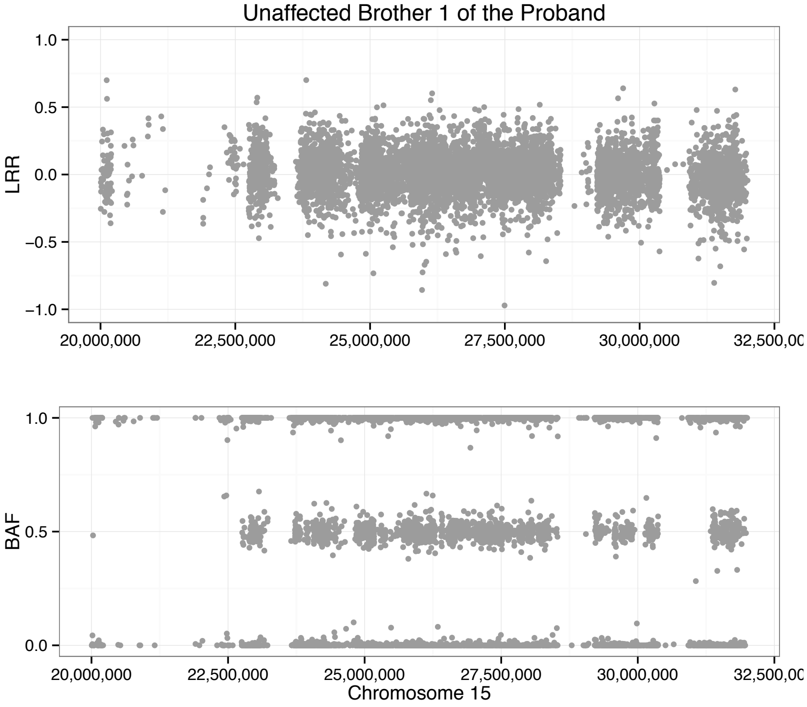

Fig. S6 Distributions of Log-R ratios (LRR) and B allele frequencies (BAF) in 15q11.2-15q13 of the microrarray data from the unaffected brother (K10031-10233) of the proband with PWS.

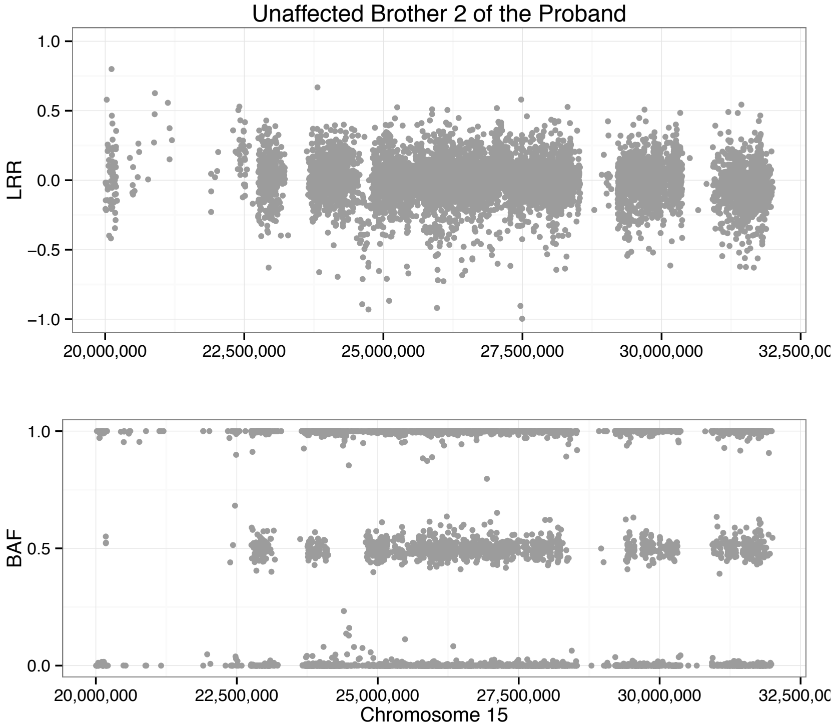

Fig. S7 Distributions of Log-R ratios (LRR) and B allele frequencies (BAF) in 15q11.2-15q13 of the microrarray data from the unaffected brother (K10031-10234) of the proband with PWS.

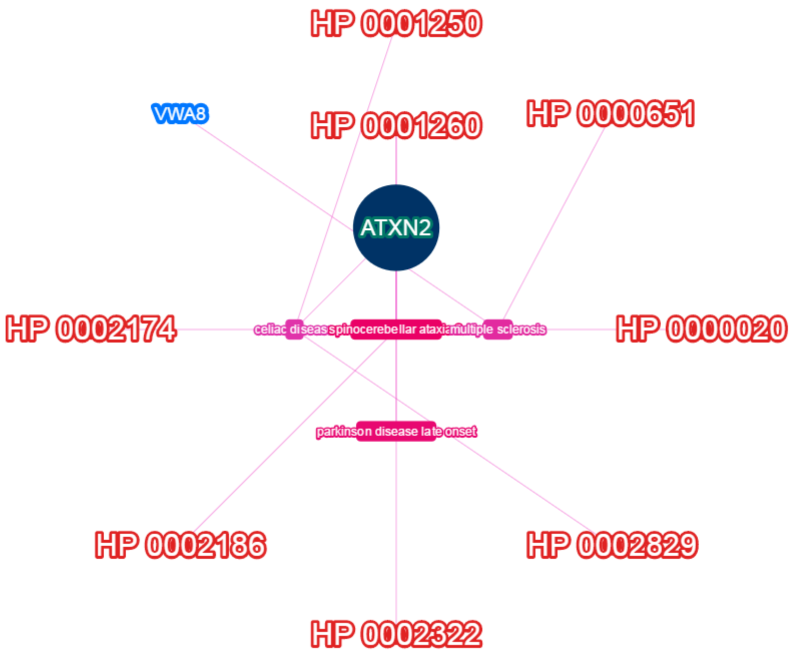

Fig. S8 Phenolyzer analysis of dysautonomia-like symptoms revealed the complexity of such diseases. Phenolyzer predicted four possible diagnoses and two seed genes for the individual (K10031-10133) with dysautonomia-like symptoms. HPO terms are colored in red, disease terms are colored in pink, seed genes are colored in blue.

Pharmacogenomic analyses for individual K10031-10133

Pharmacogenomic analyses were performed on individual K10031-10133 using the Omicia Opal platform, which is based on dosage-calculating algorithms from the PharmGKB database[1]. Pharmacogenetic variants influencing the metabolism of warfarin and simvastatin were found. These two medications were being routinely prescribed to K10031-10133. Pharmacogenomic analysis guidelines and algorithms were obtained from the International Warfarin Pharmarcogenomics Consortium (IWPC) and the Clinical Pharmacogenomics Implementation Consortium (CPIC) [2-4]. Dose calculation of warfarin was done based on the IWPC algorithm, which considers the individual's CKORC1 and CYP2C9 genotype, age, height, weight, race, history of enzyme inducer and amiodarone. The calculation suggests a warfarin dosage at 5.85 mg per day warfarin for 10031 (Table S8). For simvastatin, the CPIC algorithm suggests modestly-increased myopathy risk for people with the C allele at SLCO1B1 rs4149056 taking simvastatin doses of 40 mg daily (Table S8). Thus, a lower dosage of simvastatin at 20 mg per day is recommended instead.

The resulting calculation is very close to the previous prescriptions from the individual's cardiologist, although the pharmacogenomic recommendation for warfarin is slightly higher than the actual prescription (0.85 mg/day). This suggests that for this single individual, pharmacogenomics results confirm the appropriate dosages for these two medications. In fact, the FDA recommended doses for warfarin consist of a wide range, namely 2 to 10 mg per day. For simvastatin, the recommended FDA dose is 80 mg daily, which is much higher than the actual prescription dosages. In this case, dosage calculation purely relied on the individual's genotype

and other general information. One could include this information at least for a genotype-driven prescription.

Additional clinical information of individuals in the study:

K10031-10232:
Significant environmental risk was incurred when K10031-10232 was born at 36 weeks gestation through an emergency cesarean section (C-section) due to maternal failure to pass the nonstress test (NST). The pregnancy was significant for severe gestational diabetes and oligohydramnios, the latter serving as the original indication for the NST. His neonatal course was without complication, although some degree of neonatal hypotonia was noted. In regards to his family history, his paternal grandmother is reported to have anxiety disorder, and his father to have thrown vehement tantrums as a child. However, the tantrums subsided during his developmental maturation.

At K10031-10232's most recent clinical visit at 24 y.o., his height, weight, and BMI were 1.778 m, 79.83 kg, and 25.25 kg/m^2 respectively. He appeared happy and was performing well on daily doses of Abilify and Zoloft for treating his OCD, and subcutaneously injection of Somatropin (gonadotropin) 6 days per week for his PWS. Although there is no evidence we can find in our proband's medical record supporting the gonadotropin therapy, low baseline and GnRH-stimulated gonadotropin levels are a frequently reported finding in adolescents and adults with PWS. He has been using the continuous positive airway pressure (CPAP) machine during the past 6 months for his OSAS, and has been tolerating it well. His hyperphagia is continuously managed by parental education and structured, consistent parenting techniques. He is also exercising regularly by walking approximately six miles daily six days per week. Overall, from a PWS standpoint, he is functioning well and has transitioned successfully into a community setting, where he is employed at a laundry facility.

Test result -Autism Diagnostic Observation Schedule-Generic (ADOS)-Module 2
Communication Score: 4
Social Interaction Score: 4
Stereotyped Behaviors & Restricted Interests: 0
Other Abnormal Behaviors: 4
Overall Score: 12

K10031-10232's Language and Communication activities displayed some relatively complex language, but there were also grammatical errors, and intonation and volume varied across contexts. His conversation took place through role-playing, and some of his gestures were exaggerated, limited, or inappropriate to the given situation. For the reciprocal social interaction domain, he was able to make strong eye contact to initiate or terminate an activity with the staff, yet his facial expressions were usually out of context. He needed to be redirected on less important tasks by the examiner; when a task was broken into smaller steps and "farm animals" asked him to complete it, he engaged quickly and completed the task successfully. In the imagination domain, K10031-10232 displays spontaneity, creativity, and make-believe actions. K10031-10232 was markedly anxious during the assessment, which may have affected the reliability of the scores.

The Childhood Autism Rating Scale (CARS)
The parents of K10031-10232 yielded a score of 37.5, which falls in the high end of mildly-moderately autistic. The parents noted that their son's intellectual level is abnormal in that he is not as intelligent as a "normally developing child", but he may function better than the "normal

child” of the same age in one or more areas. The parents also rated his emotional response to specific situations as inappropriate and inhibited (score of 3 for Emotional Responses). They also noted intense and frequent abnormal body movements (3.5 score for Body Use).

K10031-10133:
A dual-chamber pacemaker (PM) was implanted when she was 21 to help manage her condition. However, despite the PM placement, she continued to experience syncopal events, with a frequency of one episode a week starting at age 23. In addition, she was recommended a full power wheelchair for the best quality of life to reduce injuries including concussions caused by frequent falls. This recent presentation has left K10031-10133 feeling overwhelmed and unsatisfied, resulting in the development of anxiety and depression diagnoses. She reported negative diagnostic test results included kidney ultrasound, chest X-ray, thyroid profile, urine vanillylmandelic acid (VMA) level, catecholamines panel (urine-free) and basic metabolic panel (BMP), and epinephrine and nor-epinephrine levels.

Her other remarkable medical history included a right hemisphere ischemic stroke at the age of 22. Causes for the stroke include the added risk of oral contraceptives (OCP) use for her irregular periods and a pre-existing patent foramen ovale (PFO). The stroke has led to residual left-side numbness, weakness and balance issues, as well as apraxia and dysarthria. Her other diagnoses include asthma, joint stiffness, hyperlipidemia, sleep walking, and dyspnea. See Table 2 and S6 for proband K10031-10133’s clinical phenotype list with HPO annotations. See supplemental note 6 for a full report of HPO analysis on proband K10031-10133. A video recording (HDV_0079) of K10031-10133 explaining her medical presentation is described in the supplemental videos section and can be provided on request to qualified investigators.

K10031-10138:
K10031-10133’s brother. He is put in a wheelchair to prevent the injuries from frequent syncopal events. He has bradycardia and tachycardia. He is also diagnosed with Tourette Syndrome; observable tics include tapping, abdominal crunches, vocal sniffing and grunting, and eyeblinking. And his ADD makes it difficult to focus in school. Moreover, he has OCD too; everything must be clean and “just right”, and needs a private bathroom.

K10031-10261:
K10031-10133’s another brother, has dark brown hair, pale skin, is overweight. He has asthma, seizures in response to Pertussis, dyslexia, migraines, arthritis, is very forgetful, has ADD but is not hyper, and has had two syncopic episodes. See HDV_0072 for a clinical presentation of K10031-10261.

K10031-10145:
The mother of the proband K10031-10133, has hemochromatosis and dysautonomia-like symptomes, which is claimed to have been inherited from her biological mother. Her dysautonomia-like symptoms started during her anorexia issues at age 19. She experiences dizziness frequently and has OCD (is always checking the curling irons to ensure they are turned off). She is also diagnosed with anxiety, depression, osteoporosis, dissociative identity disorder (DID) which makes her moody and resulted in a period of suicidal thoughts.

K10031-10143:
K10031-10133’s sister, is quite normal other than her separation anxiety. She does get dizzy but only passed out once.

K10031-10235:
K10031-10133's sister, has acid reflux, tremors, migraines, asthma, anxiety, and possibly bipolar disorder. The bipolar disorder is suspected due to family members' concerns for her mood swings, spending sprees, and irritability. See description of supplemental videos HDV_0080 and HDV_0081 at the end of document for further details.

K10031-10144:
K10031-10133's father, has migraines, arthritis, gastroesophageal reflux disease (GERD), and hiatal hernia. He has ADD, a history of suicidal attempt, and has two sisters with mental illnesses.

K10031-10233:
First cousin to K10031-10133 and brother to K10031-10232, is diagnosed with ADHD and depression. It is worth noting that K10031-10233's other brother, K10031-10234, is also diagnosed with ADD, as well as bipolar disorder and Tourette syndrome.

Supplemental video descriptions (Videos can be made available upon request to qualified investigators):

HDV_0079:
Video Summary: In this video, the proband K10031-10133 introduces her current medical conditions, including dysautonomia-like symptoms, bradycardia, tachycardia, heat intolerance, gastrophoresis, stroke, PFO, and glaucoma. She denies any psychological diagnosis on herself such as OCD and Tourette syndrome. Her parents (K10031-10145/10231) also discussed their family history of osteoporosis, Tourette's and bipolar disorder.

HDV_0072:
Video Summary: In this video, more relatives of the proband K10031-10133 were introduced, including her mother (K10031-10145), her maternal uncle (K10031-10231), her younger sisters (K10031-10235/10143), her younger brother (K10031-10261), and her cousin (K10031-10232). Medical history was collected from her younger brother (K10031-10261), who reports to have slight blood pressure issues, two fainting episodes and ADD, one of her younger sister (K10031-10235) and her cousin with Prader-Willi syndrome (PWS) (K10031-10232).

HDV_0073:
Video Summary: More interviews on other proband with PWS (K10031-10232) and his father (K10031-10231) were made in this video. His father (10231) explains the medications his son is taking, including growth hormone, Abilify, and Sertraline, and certain types of behaviors his son presents before taking his medications. Also, the mother of the female proband K10031-10133 explains more about her own medical diagnoses, including hemochromatosis and low blood pressure, as well as her children's issues with dysautonomia-like symptoms. Another maternal uncle (K10031-10262) was also interviewed in this video, who explains the negative medical and family histories of his own family. During the interview, his verbal consent of participation in this study was also collected.

Supplemental references:

1. Hewett M, Oliver DE, Rubin DL, Easton KL, Stuart JM, Altman RB, Klein TE. PharmGKB: the Pharmacogenetics Knowledge Base. Nucleic Acids Res. 2002; 30:163-165.

2. Johnson JA, Gong L, Whirl-Carrillo M, Gage BF, Scott SA, Stein CM, Anderson JL, Kimmel SE, Lee MTM, Pirmohamed M, et al. Clinical Pharmacogenetics Implementation Consortium Guidelines for CYP2C9 and VKORC1 Genotypes and Warfarin Dosing. Clin Pharmacol Ther. 2011;90:625-629.
3. Wilke RA, Ramsey LB, Johnson SG, Maxwell WD, McLeod HL, Voora D, Krauss RM, Roden DM, Feng Q, Cooper-DeHoff RM, et al. The Clinical Pharmacogenomics Implementation Consortium: CPIC Guideline for SLCO1B1 and Simvastatin-Induced Myopathy. Clin Pharmacol Ther. 2012; 92:112-117.
4. The International Warfarin Pharmacogenetics C: Estimation of the Warfarin Dose with Clinical and Pharmacogenetic Data. The New England journal of medicine. 2009;360:753-764.
