# Supplementary material for: Whole genome sequencing of one complex pedigree illustrates challenges with genomic medicine
Source: BMC Med Genomics. 2017 Feb 23;10:10. doi: 10.1186/s12920-017-0246-5 (PMC5322674; doi:10.1186/s12920-017-0246-5)
Supplement: Additional file 2: — Tute Genomics Report. This file contains nine Tute Genomics reports of all participants included in the study. (PDF 720 kb) [file 12920_2017_246_MOESM2_ESM.pdf]

TUTE GENOMICS

NextStep Dx PLUS report for K10031 10232 annotation

ORDERING PROVIDER

,

SPECIMEN

Collection Date:
Received Date:
Report Date:

PATIENT

Name:
Date of Birth:
Patient ID:
Gender:

Clinical Indication:

[Detailed clinical description.]

TEST RESULTS SUMMARY

Primary Result(s): [Findings related to clinical indication.]

Incidental/Secondary Result(s): [Incidental findings.]

PRIMARY/DIAGNOSTIC SEQUENCE VARIANT(S) DETECTED

Gene	Nucleotide Change	Protein Change	Zygosity	Tute Dx
HFE	c.845G>A	p.C282Y	het	Hereditary hemochromatosis|Hemochromatosis, juvenile, digenic
CFTR	c.4056G>C	p.Q1352H	het	Cystic fibrosis;Congenital bilateral absence of the vas deferens
C9	c.346C>T	p.R116X	het	Complement component 9 deficiency
BRIP1	c.139C>G	p.P47A	het	Breast cancer, early-onset
F5	c.1601A>G	p.Q534R	hom	Thrombophilia due to factor V Leiden
DPYD	c.85C>T	p.R29C	hom	Dihydropyrimidine dehydrogenase deficiency;not provided
PRSS1	c.86A>T	p.N29I	het	Hereditary pancreatitis;Hereditary pancreatitis
FUT2	c.461G>A	p.W154X	het	Norwalk virus infection, resistance to
PRSS1	c.47C>T	p.A16V	het	Hereditary pancreatitis
PRSS1	c.161A>G	p.N54S	het	Hereditary pancreatitis
FGFR4	c.1162G>A	p.G388R	hom	Cancer progression and tumor cell motility
ITGB3	c.176T>C	p.L59P	het	Thrombocytopenia, neonatal alloimmune|Posttransfusion purpura
TIRAP	c.539C>T	p.S180L	het	Malaria, resistance to|BACTEREMIA, RESISTANCE TO
HEXB	c.185T>C	p.L62S	hom	Sandhoff disease, infantile type
BANK1	c.182G>A	p.R61H	hom	Systemic lupus erythmatosus, association with
BBS2	c.209G>A	p.S70N	hom	Bardet-Biedl syndrome 2
ITGA2B	c.2621T>G	p.I874S	hom	Bak platelet-specific antigen
FCGR3B	c.302A>G	p.N101S	het	Neutrophil-specific antigens na1/na2
SLC24A5	c.331A>G	p.T111A	het	Skin/hair/eye pigmentation, variation in, 4
ART4	c.793G>A	p.D265N	het	DOMBROCK BLOOD GROUP

SECONDARY/INCIDENTAL SEQUENCE VARIANT(S) DETECTED

The American College of Medical Genetics and Genomics (ACMG) recently published a policy statement on clinical sequencing that emphasizes the possibility of recognizing and reporting incidental or secondary findings of medical value when conducting exome and genome sequencing. The ACMG recommends that laboratories performing clinical sequencing seek and report mutations of specified classes or types in the genes listed within the guidelines. This section of the report describes variants which fit the criteria outlined by ACMG for the reporting of incidental or secondary findings.

Gene	Nucleotide Change	Protein Change	Zygosity	Tute Dx
MSH2	c.1168C>T	p.L390F	het	Colorectal cancer, non-polyposis
MSH6	c.3143A>T	p.Q1048L	het	.
MUTYH	c.1276C>T	p.R426C	het	Adenomatous polyposis coli
SDHD	c.371A>G	p.Q124R	hom	.
CACNA1S	c.5399T>C	p.L1800S	het	.
FBN1	c.1415G>A	p.C472Y	hom	.
APOB	c.1853C>T	p.A618V	het	.
TP53	c.215C>G	p.P72R	het	.
BRCA2	c.7397T>C	p.V2466A	hom	Breast cancer
MUTYH	c.1014G>C	p.Q338H	het	.
MEN1	c.1636A>G	p.T546A	hom	.
BRCA1	c.2612C>T	p.P871L	het	.
DSG2	c.2318G>A	p.R773K	het	.
APC	c.5465T>A	p.V1822D	hom	.
PMS2	c.1621A>G	p.K541E	hom	Colorectal cancer, non-polyposis
COL3A1	c.4059T>G	p.H1353Q	hom	.
MYLK	c.1486C>G	p.L496V	hom	.
PCSK9	c.1420G>A	p.V474I	hom	.
TNNT2	c.758A>G	p.K253R	het	.
APOB	c.13013G>A	p.S4338N	het	.

INTERPRETATION OF PRIMARY FINDINGS

c.845G>A

The patient is heterozygous for a common missense polymorphism in the HFE gene which occurs at a frequency of 0.013% in the 1000 genomes project. OMIM reports 6 phenotypes associated with variants in the HFE gene including Hemochromatosis, [Transferrin serum level QTL2], susceptibility to Alzheimer disease, Microvascular complications of diabetes 7, susceptibility to Porphyria cutanea tarda, susceptibility to Porphyria variegata. This variant causes a nonsynonymous severe amino acid substitution (p.C282Y) which may alter protein function. ClinVar classifies this variant as

acid substitution (p.C282Y) which may alter protein function. ClinVar classifies this variant as pathogenic, other and non-pathogenic and reports an association with Hereditary hemochromatosis, Porphyria cutanea tarda (susceptibility to), Porphyria variegata (susceptibility to), Hemochromatosis (juvenile) (digenic), Alzheimer disease (susceptibility to), Transferrin serum level quantitative trait locus 2, Microvascular complications of diabetes 7, not specified. This variant is predicted to be deleterious by SIFT and deleterious by PolyPhen2.

c.4056G>C

The patient is heterozygous for a common missense polymorphism in the CFTR gene which occurs at a frequency of 0.0042% in the 1000 genomes project. OMIM reports 6 phenotypes associated with variants in the CFTR gene including Congenital bilateral absence of vas deferens, Cystic fibrosis, Sweat chloride elevation without CF, modifier of Bronchiectasis with or without elevated sweat chloride 1, Hypertrypsinemia (neonatal), Pancreatitis (idiopathic). This variant causes a nonsynonymous conservative amino acid substitution (p.Q1352H) which may alter protein function. ClinVar classifies this variant as pathogenic and reports an association with Cystic fibrosis;Congenital bilateral absence of the vas deferens. This variant is predicted to be deleterious by SIFT and deleterious by PolyPhen2.

c.346C>T

The patient is heterozygous for a common nonsense polymorphism in the C9 gene which occurs at a frequency of 0.003% in the 1000 genomes project. OMIM reports 2 phenotypes associated with variants in the C9 gene including C9 deficiency, suscepbitility to Macular degeneration (age-related) (15). This variant introduces a premature stop codon which may lead to early termination of protein transcription. ClinVar classifies this variant as pathogenic and reports an association with Complement component 9 deficiency. This variant is predicted to be tolerable by SIFT

c.139C>G

The patient is heterozygous for a rare missense mutation in the BRIP1 gene which doesn't occur in the 1000 genomes project. OMIM reports 2 phenotypes associated with variants in the BRIP1 gene including Breast cancer (early-onset), Fanconi anemia (complementation group J). This variant causes a nonsynonymous conservative amino acid substitution (p.P47A) which may alter protein function. ClinVar classifies this variant as pathogenic and other and reports an association with Breast cancer (early-onset), Neoplastic Syndromes (Hereditary). This variant is predicted to be deleterious by PolyPhen2.

c.1601A>G

The patient is homogeneous for a common missense polymorphism in the F5 gene which occurs at a frequency of 0.99% in the 1000 genomes project. OMIM reports 6 phenotypes associated with variants in the F5 gene including Factor V deficiency, Thrombophilia due to activated protein C resistance, Budd-Chiari syndrome, susceptibility to Pregnancy loss recurrent 1, susceptibility to Stroke (ischemic), susceptibility to Thrombophilia due to factor V Leiden. This variant causes a nonsynonymous conservative amino acid substitution (p.Q534R) which may alter protein function. ClinVar classifies this variant as pathogenic, untested and other and reports an association with Thrombophilia due to factor V Leiden, Ischemic stroke (susceptibility to), Budd-Chiari syndrome (susceptibility to), Recurrent abortion. This variant is predicted to be tolerable by SIFT

c.85C>T

The patient is homogeneous for a common missense polymorphism in the DPYD gene which occurs at a frequency of 0.74% in the 1000 genomes project. OMIM reports 2 phenotypes associated with variants in the DPYD gene including 5-fluorouracil toxicity, Dihydropyrimidine dehydrogenase deficiency. This variant causes a nonsynonymous severe amino acid substitution (p.R29C) which may alter protein function. ClinVar classifies this variant as pathogenic and reports an association with Dihydropyrimidine dehydrogenase deficiency;. This variant is predicted to be tolerable by SIFT and deleterious by PolyPhen2.

c.86A>T

The patient is heterozygous for a common missense polymorphism in the PRSS1 gene which

[illegible]
doesn't occur in the 1000 genomes project. OMIM reports 2 phenotypes associated with variants in the PRSS1 gene including Pancreatitis (hereditary), Trypsinogen deficiency. This variant causes a nonsynonymous moderate amino acid substitution (p.N29I) which may alter protein function. ClinVar classifies this variant as pathogenic and reports an association with Hereditary pancreatitis;Hereditary pancreatitis. This variant is predicted to be deleterious by SIFT and benign by PolyPhen2.

c.461G>A

The patient is heterozygous for a common nonsense polymorphism in the FUT2 gene which occurs at a frequency of 0.32% in the 1000 genomes project. OMIM reports 3 phenotypes associated with variants in the FUT2 gene including [Bombay phenotype], resistance to Norwalk virus infection, Vitamin B12 plasma level QTL1. This variant introduces a premature stop codon which may lead to early termination of protein transcription. ClinVar classifies this variant as non-pathogenic, pathogenic and other and reports an association with SECRETOR/NONSECRETOR POLYMORPHISM, Norwalk virus infection (resistance to), Vitamin b12 plasma level quantitative trait locus 1. This variant is predicted to be tolerable by SIFT

c.47C>T

The patient is heterozygous for a common missense polymorphism in the PRSS1 gene which doesn't occur in the 1000 genomes project. OMIM reports 2 phenotypes associated with variants in the PRSS1 gene including Pancreatitis (hereditary), Trypsinogen deficiency. This variant causes a nonsynonymous moderate amino acid substitution (p.A16V) which may alter protein function. ClinVar classifies this variant as pathogenic and reports an association with Hereditary pancreatitis. This variant is predicted to be tolerable by SIFT and benign by PolyPhen2.

c.161A>G

The patient is heterozygous for a common missense polymorphism in the PRSS1 gene which doesn't occur in the 1000 genomes project. OMIM reports 2 phenotypes associated with variants in the PRSS1 gene including Pancreatitis (hereditary), Trypsinogen deficiency. This variant causes a nonsynonymous conservative amino acid substitution (p.N54S) which may alter protein function. ClinVar classifies this variant as pathogenic and reports an association with Hereditary pancreatitis. This variant is predicted to be tolerable by SIFT and benign by PolyPhen2.

c.1162G>A

The patient is homogeneous for a common missense polymorphism in the FGFR4 gene which occurs at a frequency of 0.3% in the 1000 genomes project. OMIM reports that variants in the FGFR4 gene are associated with Cancer progression/metastasis. This variant causes a nonsynonymous moderate amino acid substitution (p.G388R) which may alter protein function. ClinVar classifies this variant as pathogenic and reports an association with Cancer progression and tumor cell motility. This variant is predicted to be deleterious by SIFT and deleterious by PolyPhen2.

c.176T>C

The patient is heterozygous for a common missense polymorphism in the ITGB3 gene which occurs at a frequency of 0.089% in the 1000 genomes project. OMIM reports 5 phenotypes associated with variants in the ITGB3 gene including Bleeding disorder (platelet-type) (16) (autosomal dominant), Glanzmann thrombasthenia, Purpura (posttransfusion), Thrombocytopenia (neonatal alloimmune), susceptibility to Myocardial infarction. This variant causes a nonsynonymous moderate amino acid substitution (p.L59P) which may alter protein function. ClinVar classifies this variant as non-pathogenic, pathogenic and other and reports an association with PL(A1)/(A2) ALLOANTIGEN POLYMORPHISM, Thrombocytopenia (neonatal alloimmune), Posttransfusion purpura, Myocardial infarction, Fracture (hip) (susceptibility to). This variant is predicted to be tolerable by SIFT and benign by PolyPhen2.

c.539C>T

The patient is heterozygous for a common missense polymorphism in the TIRAP gene which occurs at a frequency of 0.086% in the 1000 genomes project. OMIM reports 4 phenotypes
[illegible]

associated with variants in the TIRAP gene including protection against Bacteremia, protection against Malaria, protection against Pneumococcal disease (invasive), protection against Tuberculosis. This variant causes a nonsynonymous moderate amino acid substitution (p.S180L) which may alter protein function. ClinVar classifies this variant as other and pathogenic and reports an association with Invasive pneumococcal disease (protection against), Malaria (resistance to), Mycobacterium tuberculosis (protection against), BACTEREMIA (RESISTANCE TO). This variant is predicted to be possibly deleterious by PolyPhen2.

c.185T>C

The patient is homogeneous for a common missense polymorphism in the HEXB gene which occurs at a frequency of 0.98% in the 1000 genomes project. OMIM reports that variants in the HEXB gene are associated with Sandhoff disease (infantile) (juvenile) (and adult forms). This variant causes a nonsynonymous moderate amino acid substitution (p.L62S) which may alter protein function. ClinVar classifies this variant as pathogenic and reports an association with Sandhoff disease (infantile type). This variant is predicted to be tolerable by SIFT

c.182G>A

The patient is homogeneous for a common missense polymorphism in the BANK1 gene which occurs at a frequency of 0.22% in the 1000 genomes project. OMIM reports that variants in the BANK1 gene are associated with association with Systemic lupus erythematosus. This variant causes a nonsynonymous conservative amino acid substitution (p.R61H) which may alter protein function. ClinVar classifies this variant as pathogenic and reports an association with Systemic lupus erythmatosus (association with). This variant is predicted to be tolerable by SIFT and benign by PolyPhen2.

c.209G>A

The patient is homogeneous for a common missense polymorphism in the BBS2 gene which occurs at a frequency of 1.% in the 1000 genomes project. OMIM reports that variants in the BBS2 gene are associated with Bardet-Biedl syndrome 2. This variant causes a nonsynonymous conservative amino acid substitution (p.S70N) which may alter protein function. ClinVar classifies this variant as pathogenic and reports an association with Bardet-Biedl syndrome 2. This variant is predicted to be tolerable by SIFT

c.2621T>G

The patient is homogeneous for a common missense polymorphism in the ITGA2B gene which occurs at a frequency of 0.4% in the 1000 genomes project. OMIM reports 3 phenotypes associated with variants in the ITGA2B gene including Bleeding disorder (platelet-type) (16) (autosomal dominant), Glanzmann thrombasthenia, Thrombocytopenia (neonatal alloimmune) (BAK antigen related). This variant causes a nonsynonymous moderate amino acid substitution (p.I874S) which may alter protein function. ClinVar classifies this variant as pathogenic and reports an association with Bak platelet-specific antigen. This variant is predicted to be tolerable by SIFT and benign by PolyPhen2.

c.302A>G

The patient is heterozygous for a common missense polymorphism in the FCGR3B gene which occurs at a frequency of 0.53% in the 1000 genomes project. OMIM reports that variants in the FCGR3B gene are associated with Neutropenia (alloimmune neonatal). This variant causes a nonsynonymous conservative amino acid substitution (p.N101S) which may alter protein function. ClinVar classifies this variant as pathogenic and reports an association with Neutrophil-specific antigens na1/na2. This variant is predicted to be tolerable by SIFT

c.331A>G

The patient is heterozygous for a common missense polymorphism in the SLC24A5 gene which occurs at a frequency of 0.56% in the 1000 genomes project. OMIM reports 2 phenotypes associated with variants in the SLC24A5 gene including Albinism (oculocutaneous) (type VI), [Skin/hair/eye pigmentation 4 (fair)/dark skin]. This variant causes a nonsynonymous moderate amino acid substitution (p.T111A) which may alter protein function. ClinVar classifies this variant as

amino acid substitution (p.[illegible]) which may alter protein function. ClinVar classifies this variant as pathogenic and reports an association with Skin/hair/eye pigmentation (variation in) (4). This variant is predicted to be tolerable by SIFT and benign by PolyPhen2.

c.793G>A

The patient is heterozygous for a common missense polymorphism in the ART4 gene which occurs at a frequency of 0.29% in the 1000 genomes project. OMIM reports that variants in the ART4 gene are associated with [Blood group (Dombrock)]. This variant causes a nonsynonymous conservative amino acid substitution (p.D265N) which may alter protein function. ClinVar classifies this variant as pathogenic and reports an association with DOMBROCK BLOOD GROUP. This variant is predicted to be tolerable by SIFT and benign by PolyPhen2.

INTERPRETATION OF SECONDARY FINDINGS

---

c.1168C>T

The patient is heterozygous for a common missense polymorphism in the MSH2 gene which occurs at a frequency of 0.0028% in the 1000 genomes project. OMIM reports 3 phenotypes associated with variants in the MSH2 gene including Colorectal cancer (hereditary nonpolyposis) (type 1), Mismatch repair cancer syndrome, Muir-Torre syndrome. This variant causes a nonsynonymous conservative amino acid substitution (p.L390F) which may alter protein function. ClinVar classifies this variant as unknown, non-pathogenic and other and reports an association with , Lynch syndrome, not specified, Neoplastic Syndromes (Hereditary). This variant is predicted to be deleterious by SIFT and benign by PolyPhen2.

c.3143A>T

The patient is heterozygous for a rare mutation in the MSH6 gene which doesn't occur in the 1000 genomes project. OMIM reports 3 phenotypes associated with variants in the MSH6 gene including Colorectal cancer (hereditary nonpolyposis) (type 5), Endometrial cancer (familial), Mismatch repair cancer syndrome. This variant causes a nonsynonymous moderate amino acid substitution (p.Q1048L) which may alter protein function. This variant is predicted to be tolerable by SIFT and benign by PolyPhen2.

c.1276C>T

The patient is heterozygous for a rare missense mutation in the MUTYH gene which doesn't occur in the 1000 genomes project. OMIM reports 3 phenotypes associated with variants in the MUTYH gene including Adenomas (multiple colorectal), Colorectal adenomatous polyposis (autosomal recessive) (with pilomatricomas), Gastric cancer (somatic). This variant causes a nonsynonymous severe amino acid substitution (p.R426C) which may alter protein function. ClinVar classifies this variant as unknown and other and reports an association with , Neoplastic Syndromes (Hereditary), MYH-associated polyposis. Invitae classifies this variant as Variant of uncertain significance. This variant is predicted to be tolerable by SIFT and deleterious by PolyPhen2.

c.371A>G

The patient is homogeneous for a common missense polymorphism in the SDHD gene which occurs at a frequency of 0.95% in the 1000 genomes project. OMIM reports 6 phenotypes associated with variants in the SDHD gene including Carcinoid tumors (intestinal), Cowden syndrome 3, Merkel cell carcinoma (somatic), Paraganglioma and gastric stromal sarcoma, Paragangliomas 1 (with or without deafness), Pheochromocytoma. This variant causes a nonsynonymous conservative amino acid substitution (p.Q124R) which may alter protein function. This variant is predicted to be tolerable by SIFT

c.5399T>C

The patient is heterozygous for a common missense polymorphism in the CACNA1S gene which occurs at a frequency of 0.23% in the 1000 genomes project. OMIM reports 3 phenotypes associated with variants in the CACNA1S gene including Hypokalemic periodic paralysis (type 1),

associated with variants in the CACNA1S gene including Hypokalemic periodic paralysis (type 1), Malignant hyperthermia susceptibility 5, susceptibility to Thyrotoxic periodic paralysis 1. This variant causes a nonsynonymous moderate amino acid substitution (p.L1800S) which may alter protein function. This variant is predicted to be deleterious by SIFT and deleterious by PolyPhen2.

c.1415G>A

The patient is homogeneous for a common missense polymorphism in the FBN1 gene which occurs at a frequency of 1.% in the 1000 genomes project. OMIM reports 8 phenotypes associated with variants in the FBN1 gene including Acromicric dysplasia, Aortic aneurysm (ascending) (and dissection), Ectopia lentis (familial), Geleophysic dysplasia 2, MASS syndrome, Marfan syndrome, Stiff skin syndrome, Weill-Marchesani syndrome 2 (dominant). This variant causes a nonsynonymous severe amino acid substitution (p.C472Y) which may alter protein function. This variant is predicted to be tolerable by SIFT and benign by PolyPhen2.

c.1853C>T

The patient is heterozygous for a common missense polymorphism in the APOB gene which occurs at a frequency of 0.48% in the 1000 genomes project. OMIM reports 2 phenotypes associated with variants in the APOB gene including Hypercholesterolemia (due to ligand-defective apo B), Hypobetalipoproteinemia. This variant causes a nonsynonymous moderate amino acid substitution (p.A618V) which may alter protein function. ClinVar classifies this variant as probable-non-pathogenic and reports an association with not specified. This variant is predicted to be tolerable by SIFT and deleterious by PolyPhen2.

c.215C>G

The patient is heterozygous for a common missense polymorphism in the TP53 gene which occurs at a frequency of 0.54% in the 1000 genomes project. OMIM reports 11 phenotypes associated with variants in the TP53 gene including Adrenal cortical carcinoma, Breast cancer, Choroid plexus papilloma, Colorectal cancer, Hepatocellular carcinoma, Li-Fraumeni syndrome, Nasopharyngeal carcinoma, Osteosarcoma, Pancreatic cancer, Basal cell carcinoma 7, Glioma susceptibility 1. This variant causes a nonsynonymous moderate amino acid substitution (p.P72R) which may alter protein function. ClinVar classifies this variant as non-pathogenic and reports an association with CODON 72 POLYMORPHISM ( )(rs1042522), , not specified, Neoplastic Syndromes (Hereditary). This variant is predicted to be tolerable by SIFT and possibly deleterious by PolyPhen2.

c.7397T>C

The patient is homogeneous for a common missense polymorphism in the BRCA2 gene which occurs at a frequency of 0.98% in the 1000 genomes project. OMIM reports 9 phenotypes associated with variants in the BRCA2 gene including Fanconi anemia (complementation group D1), Pancreatic cancer, Prostate cancer, Wilms tumor, susceptibility to Breast cancer (male), Breast-ovarian cancer (familial) (2), Glioblastoma 3, Medulloblastoma, Pre-B-cell acute lymphoblastic leukemia. This variant causes a nonsynonymous moderate amino acid substitution (p.V2466A) which may alter protein function. ClinVar classifies this variant as untested, unknown and non-pathogenic and reports an association with Familial cancer of breast, Breast-ovarian cancer (familial 2), not specified. This variant is predicted to be tolerable by SIFT

c.1014G>C

The patient is heterozygous for a common missense polymorphism in the MUTYH gene which occurs at a frequency of 0.31% in the 1000 genomes project. OMIM reports 3 phenotypes associated with variants in the MUTYH gene including Adenomas (multiple colorectal), Colorectal adenomatous polyposis (autosomal recessive) (with pilomatricomas), Gastric cancer (somatic). This variant causes a nonsynonymous conservative amino acid substitution (p.Q338H) which may alter protein function. ClinVar classifies this variant as non-pathogenic and reports an association with , not specified, Neoplastic Syndromes (Hereditary). This variant is predicted to be tolerable by SIFT and possibly deleterious by PolyPhen2.

c.1636A>G

The patient is homogeneous for a common missense polymorphism in the MEN1 gene which

occurs at a frequency of 0.83% in the 1000 genomes project. OMIM reports 6 phenotypes associated with variants in the MEN1 gene including Adrenal adenoma (somatic), Angiofibroma (somatic), Carcinoid tumor of lung, Lipoma (somatic), Multiple endocrine neoplasia 1, Parathyroid adenoma (somatic). This variant causes a nonsynonymous moderate amino acid substitution (p.T546A) which may alter protein function. ClinVar classifies this variant as non-pathogenic and reports an association with , not specified. This variant is predicted to be tolerable by SIFT and benign by PolyPhen2.

c.2612C>T

The patient is heterozygous for a common missense polymorphism in the BRCA1 gene which occurs at a frequency of 0.54% in the 1000 genomes project. OMIM reports 2 phenotypes associated with variants in the BRCA1 gene including Breast-ovarian cancer (familial) (1), susceptibility to Pancreatic cancer 4. This variant causes a nonsynonymous moderate amino acid substitution (p.P871L) which may alter protein function. This variant is predicted to be tolerable by SIFT and benign by PolyPhen2.

c.2318G>A

The patient is heterozygous for a common missense polymorphism in the DSG2 gene which occurs at a frequency of 0.24% in the 1000 genomes project. OMIM reports 2 phenotypes associated with variants in the DSG2 gene including Arrhythmogenic right ventricular dysplasia 10, Cardiomyopathy (dilated) (1BB). This variant causes a nonsynonymous conservative amino acid substitution (p.R773K) which may alter protein function. ClinVar classifies this variant as non-pathogenic and reports an association with not specified. This variant is predicted to be tolerable by SIFT and benign by PolyPhen2.

c.5465T>A

The patient is homogeneous for a common missense polymorphism in the APC gene which occurs at a frequency of 0.87% in the 1000 genomes project. OMIM reports 8 phenotypes associated with variants in the APC gene including Adenoma (periampullary) (somatic), Adenomatous polyposis coli, Brain tumor-polyposis syndrome 2, Colorectal cancer (somatic), Desmoid disease (hereditary), Gardner syndrome, Gastric cancer (somatic), Hepatoblastoma (somatic). This variant causes a nonsynonymous severe amino acid substitution (p.V1822D) which may alter protein function. ClinVar classifies this variant as non-pathogenic and other and reports an association with Adenomatous polyposis coli, , not specified, Familial colorectal cancer, Neoplastic Syndromes (Hereditary). This variant is predicted to be benign by PolyPhen2.

c.1621A>G

The patient is homogeneous for a common missense polymorphism in the PMS2 gene which occurs at a frequency of 0.88% in the 1000 genomes project. OMIM reports 2 phenotypes associated with variants in the PMS2 gene including Colorectal cancer (hereditary nonpolyposis) (type 4), Mismatch repair cancer syndrome. This variant causes a nonsynonymous moderate amino acid substitution (p.K541E) which may alter protein function. ClinVar classifies this variant as non-pathogenic and reports an association with Lynch syndrome. This variant is predicted to be tolerable by SIFT and benign by PolyPhen2.

c.4059T>G

The patient is homogeneous for a common missense polymorphism in the COL3A1 gene which occurs at a frequency of 1.% in the 1000 genomes project. OMIM reports 2 phenotypes associated with variants in the COL3A1 gene including Ehlers-Danlos syndrome (type III), Ehlers-Danlos syndrome (type IV). This variant causes a nonsynonymous conservative amino acid substitution (p.H1353Q) which may alter protein function. This variant is predicted to be tolerable by SIFT and benign by PolyPhen2.

c.1486C>G

The patient is homogeneous for a common missense polymorphism in the MYLK gene which occurs at a frequency of 1.% in the 1000 genomes project. OMIM reports that variants in the MYLK [illegible]

gene are associated with Aortic aneurysm (familial thoracic 7). This variant causes a nonsynonymous conservative amino acid substitution (p.L496V) which may alter protein function. This variant is predicted to be tolerable by SIFT and benign by PolyPhen2.

c.1420G>A

The patient is homogeneous for a common missense polymorphism in the PCSK9 gene which occurs at a frequency of 0.87% in the 1000 genomes project. OMIM reports 2 phenotypes associated with variants in the PCSK9 gene including Hypercholesterolemia (familial) (3), Low density lipoprotein cholesterol level QTL 1. This variant causes a nonsynonymous conservative amino acid substitution (p.V474I) which may alter protein function. This variant is predicted to be tolerable by SIFT and benign by PolyPhen2.

c.758A>G

The patient is heterozygous for a common missense polymorphism in the TNNT2 gene which occurs at a frequency of 0.097% in the 1000 genomes project. OMIM reports 4 phenotypes associated with variants in the TNNT2 gene including Cardiomyopathy (dilated) (1D), Cardiomyopathy (familial hypertrophic) (2), Cardiomyopathy (familial restrictive) (3), Left ventricular noncompaction 6. This variant causes a nonsynonymous conservative amino acid substitution (p.K253R) which may alter protein function. ClinVar classifies this variant as non-pathogenic and reports an association with not specified. This variant is predicted to be tolerable by SIFT and benign by PolyPhen2.

c.13013G>A

The patient is heterozygous for a common missense polymorphism in the APOB gene which occurs at a frequency of 0.63% in the 1000 genomes project. OMIM reports 2 phenotypes associated with variants in the APOB gene including Hypercholesterolemia (due to ligand-defective apo B), Hypobetalipoproteinemia. This variant causes a nonsynonymous conservative amino acid substitution (p.S4338N) which may alter protein function. ClinVar classifies this variant as probable-non-pathogenic and reports an association with not specified. This variant is predicted to be tolerable by SIFT and benign by PolyPhen2.

RECOMMENDATIONS

Genetic counseling of the family is recommended to discuss the implications of this report. [Additional recommendations.]

METHODS AND LIMITATIONS

This report is a sample to be used solely for the purpose of evaluating functionality of the Tute Platform and should not be used in any clinical application.

Sequencing and Variant Detection

Whole exome sequencing was performed by solution-based hybridization which enriches for coding exons targeting DNA with specific baits. Sequencing was performed using the Illumina HiSeq 2500 to a minimum average exome depth of 90X. Sequencing reads were aligned to the reference genome (hg19) by BWA-MEM and variants were called using FreeBayes.

Variant Analysis and Report Generation

Reported variants were analyzed using the Tute platform to identify those variants which are reported by medical literature to be pathogenic. All non-synonymous variants of allele frequency <1% reported by the 1000 genomes project and annotated as ‘Pathogenic’ or ‘Probably Pathogenic’ by ClinVar are reported in the table ‘Primary/Diagnostic Sequence Variant(s) Detected’. Careful correlation of these variants with the clinical indications for the proband should be conducted by an experience medical geneticist to verify that reported findings are consistent with reported clinical phenotypes. Additionally, variants are included based on ACMG guidelines for the reporting of incidental findings in the table ‘Secondary/Incidental Sequence Variant(s) Detected’.

Validation of reported findings

Variants of interest should be confirmed by Sanger sequencing. The DNA immediately flanking each of these variants should be amplified genomic DNA followed by sequencing in both directions using fluorescent dideoxy sequencing.

Limitations

Absence of a primary diagnostic finding identified by this test does not exclude the possibility of a genetic basis for the clinical condition for this proband. Certain genetic abnormalities are not detectable by the technologies used to generate this report. Specifically, detection of abnormal variants depends on the presence of these sequence variants in the sequencing data. This exome sequence test is designed to evaluate single nucleotide variants within the human exome; however, this technology is only able to sequence 90-95% of the human reference to the requisite 10-fold coverage needed for reliable detection of heterozygous variants.

Additionally, certain types of genetic abnormalities are difficult to identify in sequencing data and have not been validated for clinical use including insertions, deletions, copy number alterations, long repetitive sequences, triplet repeat expansions, chromosomal rearrangements, polyploidy, repetitive regions including mono-, di- and tri-nucleotide repeats, GX rich regions, intronic variants outside the splice-site and epigenetic effects.

Variants believed to be benign based on medical literature, or with population frequencies greater than or equal to 1%, or resulting in synonymous amino acid changes, or occurring in 5’ or 3’ untranslated regions are generally not reported.

This report is a sample to be used solely for the purpose of evaluating functionality of the Tute Platform and should not be used in any clinical application.

Sources

REPORT SIGNATURES

Online Mendelian Inheritance in Man, OMIM (TM). Retrieved 0/26/2015 from http://www.ncbi.nlm.nih.gov/omim/
ClinVar. Retrieved 0/26/2015 from http://www.ncbi.nlm.nih.gov/clinvar/

TUTE GENOMICS

NextStep Dx PLUS report for K10031 10133 annotation

Apr 03, 2015

ORDERING PROVIDER

,

SPECIMEN

Collection Date:
Received Date:
Report Date:

PATIENT

Name:
Date of Birth:
Patient ID:
Gender:

Clinical Indication:

[Detailed clinical description.]

TEST RESULTS SUMMARY

Primary Result(s): [Findings related to clinical indication.]

Incidental/Secondary Result(s): [Incidental findings.]

PRIMARY/DIAGNOSTIC SEQUENCE VARIANT(S) DETECTED

Gene	Nucleotide Change	Protein Change	Zygosity	Tute Dx
HFE	c.845G>A	p.C282Y	het	Hereditary hemochromatosis|Hemochromatosis, juvenile, digenic
MKKS	c.724G>T	p.A242S	het	McKusick Kaufman syndrome
BRIP1	c.139C>G	p.P47A	het	Breast cancer, early-onset
F5	c.1601A>G	p.Q534R	hom	Thrombophilia due to factor V Leiden
TGM1	c.1552G>A	p.V518M	het	Autosomal recessive congenital ichthyosis 1
DPYD	c.85C>T	p.R29C	hom	Dihydropyrimidine dehydrogenase deficiency;not provided
PRSS1	c.86A>T	p.N29I	het	Hereditary pancreatitis;Hereditary pancreatitis
FUT2	c.461G>A	p.W154X	het	Norwalk virus infection, resistance to
PRSS1	c.47C>T	p.A16V	het	Hereditary pancreatitis
PRSS1	c.161A>G	p.N54S	het	Hereditary pancreatitis
ELAC2	c.1621G>A	p.A541T	het	Prostate cancer, hereditary, 2
OCA2	c.913C>T	p.R305W	het	Skin/hair/eye pigmentation, variation in, 1
PRF1	c.755A>G	p.N252S	het	Hemophagocytic lymphohistiocytosis, familial, 2|Malignant lymphoma, non-Hodgkin
KRT85	c.233G>A	p.R78H	het	Ectodermal dysplasia, 'pure' hair-nail type
FGFR4	c.1162G>A	p.G388R	het	Cancer progression and tumor cell motility
ITGB3	c.176T>C	p.L59P	hom	Thrombocytopenia, neonatal alloimmune|Posttransfusion purpura
CST3	c.73G>A	p.A25T	het	Age-related macular degeneration 11
HEXB	c.185T>C	p.L62S	hom	Sandhoff disease, infantile type
BBS2	c.209G>A	p.S70N	hom	Bardet-Biedl syndrome 2
KLK1	c.230G>A	p.R77H	het	Kallikrein, decreased urinary activity of

SECONDARY/INCIDENTAL SEQUENCE VARIANT(S) DETECTED

The American College of Medical Genetics and Genomics (ACMG) recently published a policy statement on clinical sequencing that emphasizes the possibility of recognizing and reporting incidental or secondary findings of medical value when conducting exome and genome sequencing. The ACMG recommends that laboratories performing clinical sequencing seek and report mutations of specified classes or types in the genes listed within the guidelines. This section of the report describes variants which fit the criteria outlined by ACMG for the reporting of incidental or secondary findings.

Gene	Nucleotide Change	Protein Change	Zygosity	Tute Dx
RYR1	c.7336G>T	p.G2446C	het	.
PMS2	c.59G>A	p.R20Q	het	Colorectal cancer, non-polyposis
SDHD	c.371A>G	p.Q124R	hom	.
FBN1	c.1415G>A	p.C472Y	hom	.
SCN5A	c.1673A>G	p.H558R	het	Progressive familial heart block type 1A
DSC2	c.2326A>G	p.I776V	het	.
APOB	c.1853C>T	p.A618V	het	.
TP53	c.215C>G	p.P72R	het	.
BRCA2	c.7397T>C	p.V2466A	hom	Breast cancer
MUTYH	c.1014G>C	p.Q338H	het	.
MEN1	c.1636A>G	p.T546A	hom	.
DSG2	c.2318G>A	p.R773K	het	.
APC	c.5465T>A	p.V1822D	het	.
PMS2	c.1621A>G	p.K541E	hom	Colorectal cancer, non-polyposis
COL3A1	c.4059T>G	p.H1353Q	hom	.
BRCA2	c.1114A>C	p.N372H	het	.
MYLK	c.1486C>G	p.L496V	hom	.
PCSK9	c.1420G>A	p.V474I	hom	.
APOB	c.13013G>A	p.S4338N	hom	.
PCSK9	c.2009G>A	p.G670E	hom	.

INTERPRETATION OF PRIMARY FINDINGS

c.845G>A

The patient is heterozygous for a common missense polymorphism in the HFE gene which occurs at a frequency of 0.013% in the 1000 genomes project. OMIM reports 6 phenotypes associated with

variants in the HFE gene including Hemochromatosis, [Transferrin serum level QTL2], susceptibility to Alzheimer disease, Microvascular complications of diabetes 7, susceptibility to Porphyria cutanea tarda, susceptibility to Porphyria variegata. This variant causes a nonsynonymous severe amino acid substitution (p.C282Y) which may alter protein function. ClinVar classifies this variant as pathogenic, other and non-pathogenic and reports an association with Hereditary hemochromatosis, Porphyria cutanea tarda (susceptibility to), Porphyria variegata (susceptibility to), Hemochromatosis (juvenile) (digenic), Alzheimer disease (susceptibility to), Transferrin serum level quantitative trait locus 2, Microvascular complications of diabetes 7, not specified. This variant is predicted to be deleterious by SIFT and deleterious by PolyPhen2.

c.724G>T

The patient is heterozygous for a rare missense mutation in the MKKS gene which occurs at a frequency of 0.0018% in the 1000 genomes project. OMIM reports 2 phenotypes associated with variants in the MKKS gene including Bardet-Biedl syndrome 6, McKusick-Kaufman syndrome. This variant causes a nonsynonymous moderate amino acid substitution (p.A242S) which may alter protein function. ClinVar classifies this variant as pathogenic and reports an association with McKusick Kaufman syndrome. This variant is predicted to be tolerable by SIFT and deleterious by PolyPhen2.

c.139C>G

The patient is heterozygous for a rare missense mutation in the BRIP1 gene which doesn't occur in the 1000 genomes project. OMIM reports 2 phenotypes associated with variants in the BRIP1 gene including Breast cancer (early-onset), Fanconi anemia (complementation group J). This variant causes a nonsynonymous conservative amino acid substitution (p.P47A) which may alter protein function. ClinVar classifies this variant as pathogenic and other and reports an association with Breast cancer (early-onset), Neoplastic Syndromes (Hereditary). This variant is predicted to be deleterious by PolyPhen2.

c.1601A>G

The patient is homogeneous for a common missense polymorphism in the F5 gene which occurs at a frequency of 0.99% in the 1000 genomes project. OMIM reports 6 phenotypes associated with variants in the F5 gene including Factor V deficiency, Thrombophilia due to activated protein C resistance, Budd-Chiari syndrome, susceptibility to Pregnancy loss recurrent 1, susceptibility to Stroke (ischemic), susceptibility to Thrombophilia due to factor V Leiden. This variant causes a nonsynonymous conservative amino acid substitution (p.Q534R) which may alter protein function. ClinVar classifies this variant as pathogenic, untested and other and reports an association with Thrombophilia due to factor V Leiden, Ischemic stroke (susceptibility to), Budd-Chiari syndrome (susceptibility to), Recurrent abortion. This variant is predicted to be tolerable by SIFT

c.1552G>A

The patient is heterozygous for a common missense polymorphism in the TGM1 gene which occurs at a frequency of 0.0036% in the 1000 genomes project. OMIM reports that variants in the TGM1 gene are associated with Ichthyosis (congenital) (autosomal recessive 1). This variant causes a nonsynonymous conservative amino acid substitution (p.V518M) which may alter protein function. ClinVar classifies this variant as pathogenic and reports an association with Autosomal recessive congenital ichthyosis 1. This variant is predicted to be tolerable by SIFT and deleterious by PolyPhen2.

c.85C>T

The patient is homogeneous for a common missense polymorphism in the DPYD gene which occurs at a frequency of 0.74% in the 1000 genomes project. OMIM reports 2 phenotypes associated with variants in the DPYD gene including 5-fluorouracil toxicity, Dihydropyrimidine dehydrogenase deficiency. This variant causes a nonsynonymous severe amino acid substitution (p.R29C) which may alter protein function. ClinVar classifies this variant as pathogenic and reports an association with Dihydropyrimidine dehydrogenase deficiency;. This variant is predicted to be tolerable by SIFT and deleterious by PolyPhen2.

[illegible]

c.86A>T

The patient is heterozygous for a common missense polymorphism in the PRSS1 gene which doesn't occur in the 1000 genomes project. OMIM reports 2 phenotypes associated with variants in the PRSS1 gene including Pancreatitis (hereditary), Trypsinogen deficiency. This variant causes a nonsynonymous moderate amino acid substitution (p.N29I) which may alter protein function. ClinVar classifies this variant as pathogenic and reports an association with Hereditary pancreatitis;Hereditary pancreatitis. This variant is predicted to be deleterious by SIFT and benign by PolyPhen2.

c.461G>A

The patient is heterozygous for a common nonsense polymorphism in the FUT2 gene which occurs at a frequency of 0.32% in the 1000 genomes project. OMIM reports 3 phenotypes associated with variants in the FUT2 gene including [Bombay phenotype], resistance to Norwalk virus infection, Vitamin B12 plasma level QTL1. This variant introduces a premature stop codon which may lead to early termination of protein transcription. ClinVar classifies this variant as non-pathogenic, pathogenic and other and reports an association with SECRETOR/NONSECRETOR POLYMORPHISM, Norwalk virus infection (resistance to), Vitamin b12 plasma level quantitative trait locus 1. This variant is predicted to be tolerable by SIFT

c.47C>T

The patient is heterozygous for a common missense polymorphism in the PRSS1 gene which doesn't occur in the 1000 genomes project. OMIM reports 2 phenotypes associated with variants in the PRSS1 gene including Pancreatitis (hereditary), Trypsinogen deficiency. This variant causes a nonsynonymous moderate amino acid substitution (p.A16V) which may alter protein function. ClinVar classifies this variant as pathogenic and reports an association with Hereditary pancreatitis. This variant is predicted to be tolerable by SIFT and benign by PolyPhen2.

c.161A>G

The patient is heterozygous for a common missense polymorphism in the PRSS1 gene which doesn't occur in the 1000 genomes project. OMIM reports 2 phenotypes associated with variants in the PRSS1 gene including Pancreatitis (hereditary), Trypsinogen deficiency. This variant causes a nonsynonymous conservative amino acid substitution (p.N54S) which may alter protein function. ClinVar classifies this variant as pathogenic and reports an association with Hereditary pancreatitis. This variant is predicted to be tolerable by SIFT and benign by PolyPhen2.

c.1621G>A

The patient is heterozygous for a common missense polymorphism in the ELAC2 gene which occurs at a frequency of 0.023% in the 1000 genomes project. OMIM reports 2 phenotypes associated with variants in the ELAC2 gene including Combined oxidative phosphorylation deficiency 17, susceptibility to Prostate cancer (hereditary) (2). This variant causes a nonsynonymous moderate amino acid substitution (p.A541T) which may alter protein function. ClinVar classifies this variant as pathogenic and reports an association with Prostate cancer (hereditary) (2). This variant is predicted to be tolerable by SIFT and possibly deleterious by PolyPhen2.

c.913C>T

The patient is heterozygous for a common missense polymorphism in the OCA2 gene which occurs at a frequency of 0.083% in the 1000 genomes project. OMIM reports 4 phenotypes associated with variants in the OCA2 gene including Albinism (brown oculocutaneous), Albinism (oculocutaneous) (type II), [Skin/hair/eye pigmentation 1 (blond)/brown hair], [Skin/hair/eye pigmentation 1 (blue)/nonblue eyes]. This variant causes a nonsynonymous moderate amino acid substitution (p.R305W) which may alter protein function. ClinVar classifies this variant as pathogenic and reports an association with Skin/hair/eye pigmentation (variation in) (1). This variant is predicted to be deleterious by SIFT and possibly deleterious by PolyPhen2.

c.755A>G

The patient is heterozygous for a common missense polymorphism in the PRF1 gene which occurs

The patient is heterozygous for a common missense polymorphism in the PRF1 gene which occurs at a frequency of 0.0076% in the 1000 genomes project. OMIM reports 2 phenotypes associated with variants in the PRF1 gene including Hemophagocytic lymphohistiocytosis (familial) (2), Lymphoma (non-Hodgkin). This variant causes a nonsynonymous conservative amino acid substitution (p.N252S) which may alter protein function. ClinVar classifies this variant as pathogenic and reports an association with Hemophagocytic lymphohistiocytosis (familial) (2), Malignant lymphoma (non-Hodgkin). This variant is predicted to be tolerable by SIFT and benign by PolyPhen2.

c.233G>A

The patient is heterozygous for a common missense polymorphism in the KRT85 gene which occurs at a frequency of 0.033% in the 1000 genomes project. OMIM reports that variants in the KRT85 gene are associated with Ectodermal dysplasia 4 (hair)/nail type. This variant causes a nonsynonymous conservative amino acid substitution (p.R78H) which may alter protein function. ClinVar classifies this variant as pathogenic and untested and reports an association with Ectodermal dysplasia ( )'pure' hair-nail type. This variant is predicted to be tolerable by SIFT and benign by PolyPhen2.

c.1162G>A

The patient is heterozygous for a common missense polymorphism in the FGFR4 gene which occurs at a frequency of 0.3% in the 1000 genomes project. OMIM reports that variants in the FGFR4 gene are associated with Cancer progression/metastasis. This variant causes a nonsynonymous moderate amino acid substitution (p.G388R) which may alter protein function. ClinVar classifies this variant as pathogenic and reports an association with Cancer progression and tumor cell motility. This variant is predicted to be deleterious by SIFT and deleterious by PolyPhen2.

c.176T>C

The patient is homogeneous for a common missense polymorphism in the ITGB3 gene which occurs at a frequency of 0.089% in the 1000 genomes project. OMIM reports 5 phenotypes associated with variants in the ITGB3 gene including Bleeding disorder (platelet-type) (16) (autosomal dominant), Glanzmann thrombasthenia, Purpura (posttransfusion), Thrombocytopenia (neonatal alloimmune), susceptibility to Myocardial infarction. This variant causes a nonsynonymous moderate amino acid substitution (p.L59P) which may alter protein function. ClinVar classifies this variant as non-pathogenic, pathogenic and other and reports an association with PL(A1)/(A2) ALLOANTIGEN POLYMORPHISM, Thrombocytopenia (neonatal alloimmune), Posttransfusion purpura, Myocardial infarction, Fracture (hip) (susceptibility to). This variant is predicted to be tolerable by SIFT and benign by PolyPhen2.

c.73G>A

The patient is heterozygous for a common missense polymorphism in the CST3 gene which occurs at a frequency of 0.21% in the 1000 genomes project. OMIM reports 2 phenotypes associated with variants in the CST3 gene including Cerebral amyloid angiopathy, Macular degeneration (age-related) (11). This variant causes a nonsynonymous moderate amino acid substitution (p.A25T) which may alter protein function. ClinVar classifies this variant as pathogenic and reports an association with Age-related macular degeneration 11. This variant is predicted to be tolerable by SIFT and benign by PolyPhen2.

c.185T>C

The patient is homogeneous for a common missense polymorphism in the HEXB gene which occurs at a frequency of 0.98% in the 1000 genomes project. OMIM reports that variants in the HEXB gene are associated with Sandhoff disease (infantile) (juvenile) (and adult forms). This variant causes a nonsynonymous moderate amino acid substitution (p.L62S) which may alter protein function. ClinVar classifies this variant as pathogenic and reports an association with Sandhoff disease (infantile type). This variant is predicted to be tolerable by SIFT

c.209G>A

The patient is homogeneous for a common missense polymorphism in the BBS2 gene which

[illegible]
occurs at a frequency of 1.% in the 1000 genomes project. OMIM reports that variants in the BBS2 gene are associated with Bardet-Biedl syndrome 2. This variant causes a nonsynonymous conservative amino acid substitution (p.S70N) which may alter protein function. ClinVar classifies this variant as pathogenic and reports an association with Bardet-Biedl syndrome 2. This variant is predicted to be tolerable by SIFT

c.230G>A

The patient is heterozygous for a common missense polymorphism in the KLK1 gene which occurs at a frequency of 0.042% in the 1000 genomes project. OMIM reports that variants in the KLK1 gene are associated with [decreased urinary activity of Kallikrein]. This variant causes a nonsynonymous conservative amino acid substitution (p.R77H) which may alter protein function. ClinVar classifies this variant as pathogenic and reports an association with Kallikrein (decreased urinary activity of). This variant is predicted to be deleterious by SIFT and possibly deleterious by PolyPhen2.

INTERPRETATION OF SECONDARY FINDINGS

c.7336G>T

The patient is heterozygous for a rare mutation in the RYR1 gene which doesn't occur in the 1000 genomes project. OMIM reports 5 phenotypes associated with variants in the RYR1 gene including Central core disease, King-Denborough syndrome, Minicore myopathy with external ophthalmoplegia, Neuromuscular disease (congenital) (with uniform type 1 fiber), Malignant hyperthermia susceptibility 1. This variant causes a nonsynonymous severe amino acid substitution (p.G2446C) which may alter protein function. This variant is predicted to be deleterious by SIFT and benign by PolyPhen2.

c.59G>A

The patient is heterozygous for a common missense polymorphism in the PMS2 gene which occurs at a frequency of 0.076% in the 1000 genomes project. OMIM reports 2 phenotypes associated with variants in the PMS2 gene including Colorectal cancer (hereditary nonpolyposis) (type 4), Mismatch repair cancer syndrome. This variant causes a nonsynonymous conservative amino acid substitution (p.R20Q) which may alter protein function. ClinVar classifies this variant as non-pathogenic and untested and reports an association with , Lynch syndrome, not specified, Neoplastic Syndromes (Hereditary). This variant is predicted to be tolerable by SIFT and possibly deleterious by PolyPhen2.

c.371A>G

The patient is homogeneous for a common missense polymorphism in the SDHD gene which occurs at a frequency of 0.95% in the 1000 genomes project. OMIM reports 6 phenotypes associated with variants in the SDHD gene including Carcinoid tumors (intestinal), Cowden syndrome 3, Merkel cell carcinoma (somatic), Paraganglioma and gastric stromal sarcoma, Paragangliomas 1 (with or without deafness), Pheochromocytoma. This variant causes a nonsynonymous conservative amino acid substitution (p.Q124R) which may alter protein function. This variant is predicted to be tolerable by SIFT

c.1415G>A

The patient is homogeneous for a common missense polymorphism in the FBN1 gene which occurs at a frequency of 1.% in the 1000 genomes project. OMIM reports 8 phenotypes associated with variants in the FBN1 gene including Acromicric dysplasia, Aortic aneurysm (ascending) (and dissection), Ectopia lentis (familial), Geleophysic dysplasia 2, MASS syndrome, Marfan syndrome, Stiff skin syndrome, Weill-Marchesani syndrome 2 (dominant). This variant causes a nonsynonymous severe amino acid substitution (p.C472Y) which may alter protein function. This variant is predicted to be tolerable by SIFT and benign by PolyPhen2.

c.1673A>G

The patient is heterozygous for a common missense polymorphism in the SCN5A gene which occurs at a frequency of 0.23% in the 1000 genomes project. OMIM reports 9 phenotypes associated with variants in the SCN5A gene including Atrial fibrillation (familial) (10), Brugada syndrome 1, Cardiomyopathy (dilated) (1E), Heart block (nonprogressive), Heart block (progressive) (type IA), Long QT syndrome-3, Sick sinus syndrome 1, Ventricular fibrillation (familial) (1), susceptibility to Sudden infant death syndrome. This variant causes a nonsynonymous conservative amino acid substitution (p.H558R) which may alter protein function. ClinVar classifies this variant as pathogenic and non-pathogenic and reports an association with Progressive familial heart block type 1A, not specified. This variant is predicted to be tolerable by SIFT and benign by PolyPhen2.

c.2326A>G

The patient is heterozygous for a common missense polymorphism in the DSC2 gene which occurs at a frequency of 0.2% in the 1000 genomes project. OMIM reports 2 phenotypes associated with variants in the DSC2 gene including Arrhythmogenic right ventricular dysplasia 11 with mild palmoplantar keratoderma and woolly hair, Arrhythmogenic right ventricular dysplasia 11. This variant causes a nonsynonymous conservative amino acid substitution (p.I776V) which may alter protein function. ClinVar classifies this variant as non-pathogenic and reports an association with not specified. This variant is predicted to be tolerable by SIFT and benign by PolyPhen2.

c.1853C>T

The patient is heterozygous for a common missense polymorphism in the APOB gene which occurs at a frequency of 0.48% in the 1000 genomes project. OMIM reports 2 phenotypes associated with variants in the APOB gene including Hypercholesterolemia (due to ligand-defective apo B), Hypobetalipoproteinemia. This variant causes a nonsynonymous moderate amino acid substitution (p.A618V) which may alter protein function. ClinVar classifies this variant as probable-non-pathogenic and reports an association with not specified. This variant is predicted to be tolerable by SIFT and deleterious by PolyPhen2.

c.215C>G

The patient is heterozygous for a common missense polymorphism in the TP53 gene which occurs at a frequency of 0.54% in the 1000 genomes project. OMIM reports 11 phenotypes associated with variants in the TP53 gene including Adrenal cortical carcinoma, Breast cancer, Choroid plexus papilloma, Colorectal cancer, Hepatocellular carcinoma, Li-Fraumeni syndrome, Nasopharyngeal carcinoma, Osteosarcoma, Pancreatic cancer, Basal cell carcinoma 7, Glioma susceptibility 1. This variant causes a nonsynonymous moderate amino acid substitution (p.P72R) which may alter protein function. ClinVar classifies this variant as non-pathogenic and reports an association with CODON 72 POLYMORPHISM ( )(rs1042522), , not specified, Neoplastic Syndromes (Hereditary). This variant is predicted to be tolerable by SIFT and possibly deleterious by PolyPhen2.

c.7397T>C

The patient is homogeneous for a common missense polymorphism in the BRCA2 gene which occurs at a frequency of 0.98% in the 1000 genomes project. OMIM reports 9 phenotypes associated with variants in the BRCA2 gene including Fanconi anemia (complementation group D1), Pancreatic cancer, Prostate cancer, Wilms tumor, susceptibility to Breast cancer (male), Breast-ovarian cancer (familial) (2), Glioblastoma 3, Medulloblastoma, Pre-B-cell acute lymphoblastic leukemia. This variant causes a nonsynonymous moderate amino acid substitution (p.V2466A) which may alter protein function. ClinVar classifies this variant as untested, unknown and non-pathogenic and reports an association with Familial cancer of breast, Breast-ovarian cancer (familial 2), not specified. This variant is predicted to be tolerable by SIFT

c.1014G>C

The patient is heterozygous for a common missense polymorphism in the MUTYH gene which occurs at a frequency of 0.31% in the 1000 genomes project. OMIM reports 3 phenotypes associated with variants in the MUTYH gene including Adenomas (multiple colorectal), Colorectal adenomatous polyposis (autosomal recessive) (with pilomatricomas), Gastric cancer (somatic). This variant causes a nonsynonymous conservative amino acid substitution (p.Q338H) which may

alter protein function. ClinVar classifies this variant as non-pathogenic and reports an association with , not specified, Neoplastic Syndromes (Hereditary). This variant is predicted to be tolerable by SIFT and possibly deleterious by PolyPhen2.

c.1636A>G

The patient is homogeneous for a common missense polymorphism in the MEN1 gene which occurs at a frequency of 0.83% in the 1000 genomes project. OMIM reports 6 phenotypes associated with variants in the MEN1 gene including Adrenal adenoma (somatic), Angiofibroma (somatic), Carcinoid tumor of lung, Lipoma (somatic), Multiple endocrine neoplasia 1, Parathyroid adenoma (somatic). This variant causes a nonsynonymous moderate amino acid substitution (p.T546A) which may alter protein function. ClinVar classifies this variant as non-pathogenic and reports an association with , not specified. This variant is predicted to be tolerable by SIFT and benign by PolyPhen2.

c.2318G>A

The patient is heterozygous for a common missense polymorphism in the DSG2 gene which occurs at a frequency of 0.24% in the 1000 genomes project. OMIM reports 2 phenotypes associated with variants in the DSG2 gene including Arrhythmogenic right ventricular dysplasia 10, Cardiomyopathy (dilated) (1BB). This variant causes a nonsynonymous conservative amino acid substitution (p.R773K) which may alter protein function. ClinVar classifies this variant as non-pathogenic and reports an association with not specified. This variant is predicted to be tolerable by SIFT and benign by PolyPhen2.

c.5465T>A

The patient is heterozygous for a common missense polymorphism in the APC gene which occurs at a frequency of 0.87% in the 1000 genomes project. OMIM reports 8 phenotypes associated with variants in the APC gene including Adenoma (periampullary) (somatic), Adenomatous polyposis coli, Brain tumor-polyposis syndrome 2, Colorectal cancer (somatic), Desmoid disease (hereditary), Gardner syndrome, Gastric cancer (somatic), Hepatoblastoma (somatic). This variant causes a nonsynonymous severe amino acid substitution (p.V1822D) which may alter protein function. ClinVar classifies this variant as non-pathogenic and other and reports an association with Adenomatous polyposis coli, , not specified, Familial colorectal cancer, Neoplastic Syndromes (Hereditary). This variant is predicted to be benign by PolyPhen2.

c.1621A>G

The patient is homogeneous for a common missense polymorphism in the PMS2 gene which occurs at a frequency of 0.88% in the 1000 genomes project. OMIM reports 2 phenotypes associated with variants in the PMS2 gene including Colorectal cancer (hereditary nonpolyposis) (type 4), Mismatch repair cancer syndrome. This variant causes a nonsynonymous moderate amino acid substitution (p.K541E) which may alter protein function. ClinVar classifies this variant as non-pathogenic and reports an association with Lynch syndrome. This variant is predicted to be tolerable by SIFT and benign by PolyPhen2.

c.4059T>G

The patient is homogeneous for a common missense polymorphism in the COL3A1 gene which occurs at a frequency of 1.% in the 1000 genomes project. OMIM reports 2 phenotypes associated with variants in the COL3A1 gene including Ehlers-Danlos syndrome (type III), Ehlers-Danlos syndrome (type IV). This variant causes a nonsynonymous conservative amino acid substitution (p.H1353Q) which may alter protein function. This variant is predicted to be tolerable by SIFT and benign by PolyPhen2.

c.1114A>C

The patient is heterozygous for a common missense polymorphism in the BRCA2 gene which occurs at a frequency of 0.25% in the 1000 genomes project. OMIM reports 9 phenotypes associated with variants in the BRCA2 gene including Fanconi anemia (complementation group D1), Pancreatic cancer, Prostate cancer, Wilms tumor, susceptibility to Breast cancer (male), Breast- [illegible]

ovarian cancer (familial) (2), Glioblastoma 3, Medulloblastoma, Pre-B-cell acute lymphoblastic leukemia. This variant causes a nonsynonymous moderate amino acid substitution (p.N372H) which may alter protein function. ClinVar classifies this variant as other, non-pathogenic and untested and reports an association with Breast-ovarian cancer (familial 2), , not specified, Neoplastic Syndromes (Hereditary). This variant is predicted to be tolerable by SIFT

c.1486C>G

The patient is homogeneous for a common missense polymorphism in the MYLK gene which occurs at a frequency of 1.% in the 1000 genomes project. OMIM reports that variants in the MYLK gene are associated with Aortic aneurysm (familial thoracic 7). This variant causes a nonsynonymous conservative amino acid substitution (p.L496V) which may alter protein function. This variant is predicted to be tolerable by SIFT and benign by PolyPhen2.

c.1420G>A

The patient is homogeneous for a common missense polymorphism in the PCSK9 gene which occurs at a frequency of 0.87% in the 1000 genomes project. OMIM reports 2 phenotypes associated with variants in the PCSK9 gene including Hypercholesterolemia (familial) (3), Low density lipoprotein cholesterol level QTL 1. This variant causes a nonsynonymous conservative amino acid substitution (p.V474I) which may alter protein function. This variant is predicted to be tolerable by SIFT and benign by PolyPhen2.

c.13013G>A

The patient is homogeneous for a common missense polymorphism in the APOB gene which occurs at a frequency of 0.63% in the 1000 genomes project. OMIM reports 2 phenotypes associated with variants in the APOB gene including Hypercholesterolemia (due to ligand-defective apo B), Hypobetalipoproteinemia. This variant causes a nonsynonymous conservative amino acid substitution (p.S4338N) which may alter protein function. ClinVar classifies this variant as probable-non-pathogenic and reports an association with not specified. This variant is predicted to be tolerable by SIFT and benign by PolyPhen2.

c.2009G>A

The patient is homogeneous for a common missense polymorphism in the PCSK9 gene which occurs at a frequency of 0.9% in the 1000 genomes project. OMIM reports 2 phenotypes associated with variants in the PCSK9 gene including Hypercholesterolemia (familial) (3), Low density lipoprotein cholesterol level QTL 1. This variant causes a nonsynonymous moderate amino acid substitution (p.G670E) which may alter protein function. ClinVar classifies this variant as non-pathogenic and reports an association with Familial hypercholesterolemia. This variant is predicted to be tolerable by SIFT and benign by PolyPhen2.

RECOMMENDATIONS

Genetic counseling of the family is recommended to discuss the implications of this report.
[Additional recommendations.]

METHODS AND LIMITATIONS

This report is a sample to be used solely for the purpose of evaluating functionality of the Tute Platform and should not be used in any clinical application.

Sequencing and Variant Detection

Whole exome sequencing was performed by solution-based hybridization which enriches for coding exons targeting DNA with specific baits. Sequencing was performed using the Illumina HiSeq 2500 to a minimum average exome depth of 90X. Sequencing reads were aligned to the reference genome (hg19) by BWA-MEM and variants were called using FreeBayes.

Variant Analysis and Report Generation

Reported variants were analyzed using the Tute platform to identify those variants which are reported by medical literature to be pathogenic. All non-synonymous variants of allele frequency <1% reported by the 1000 genomes project and annotated as 'Pathogenic' or 'Probably Pathogenic' by ClinVar are reported in the table 'Primary/Diagnostic Sequence Variant(s) Detected'. Careful correlation of these variants with the clinical indications for the proband should be conducted by an experience medical geneticist to verify that reported findings are consistent with reported clinical phenotypes. Additionally, variants are included based on ACMG guidelines for the reporting of incidental findings in the table 'Secondary/Incidental Sequence Variant(s) Detected'.

Validation of reported findings

Variants of interest should be confirmed by Sanger sequencing. The DNA immediately flanking each of these variants should be amplified genomic DNA followed by sequencing in both directions using fluorescent dideoxy sequencing.

Limitations

Absence of a primary diagnostic finding identified by this test does not exclude the possibility of a genetic basis for the clinical condition for this proband. Certain genetic abnormalities are not detectable by the technologies used to generate this report. Specifically, detection of abnormal variants depends on the presence of these sequence variants in the sequencing data. This exome sequence test is designed to evaluate single nucleotide variants within the human exome; however, this technology is only able to sequence 90-95% of the human reference to the requisite 10-fold coverage needed for reliable detection of heterozygous variants.

Additionally, certain types of genetic abnormalities are difficult to identify in sequencing data and have not been validated for clinical use including insertions, deletions, copy number alterations, long repetitive sequences, triplet repeat expansions, chromosomal rearrangements, polyploidy, repetitive regions including mono-, di- and tri-nucleotide repeats, GX rich regions, intronic variants outside the splice-site and epigenetic effects.

Variants believed to be benign based on medical literature, or with population frequencies greater than or equal to 1%, or resulting in synonymous amino acid changes, or occurring in 5' or 3' untranslated regions are generally not reported.

This report is a sample to be used solely for the purpose of evaluating functionality of the Tute Platform and should not be used in any clinical application.

Sources

REPORT SIGNATURES

Online Mendelian Inheritance in Man, OMIM (TM). Retrieved 0/26/2015 from http://www.ncbi.nlm.nih.gov/omim/
ClinVar. Retrieved 0/26/2015 from http://www.ncbi.nlm.nih.gov/clinvar/

NextStep Dx PLUS report for K10031 10145 annotation

ORDERING PROVIDER	SPECIMEN	PATIENT
,	Collection Date:	Name:
	Received Date:	Date of Birth:
	Report Date:	Patient ID:
		Gender:

Clinical Indication:

[Detailed clinical description.]

TEST RESULTS SUMMARY

Primary Result(s): [Findings related to clinical indication.]

Incidental/Secondary Result(s): [Incidental findings.]

PRIMARY/DIAGNOSTIC SEQUENCE VARIANT(S) DETECTED

Gene	Nucleotide Change	Protein Change	Zygosity	Tute Dx
HFE	c.845G>A	p.C282Y	hom	Hereditary hemochromatosis|Hemochromatosis, juvenile, digenic
CFTR	c.224G>A	p.R75Q	het	Hereditary pancreatitis;Cystic fibrosis
MKKS	c.724G>T	p.A242S	het	McKusick Kaufman syndrome
NTRK1	c.1810C>T	p.H604Y	het	Familial medullary thyroid carcinoma|Hereditary insensitivity to pain with anhidrosis
VPS13B	c.9406-1G>T	.	het	Cohen syndrome
BRIP1	c.139C>G	p.P47A	het	Breast cancer, early-onset
F5	c.1601A>G	p.Q534R	het	Thrombophilia due to factor V Leiden
DPYD	c.85C>T	p.R29C	hom	Dihydropyrimidine dehydrogenase deficiency;not provided
PRSS1	c.86A>T	p.N29I	het	Hereditary pancreatitis;Hereditary pancreatitis
FUT2	c.461G>A	p.W154X	hom	Norwalk virus infection, resistance to
SLC6A20	c.596C>T	p.T199M	het	Hyperglycinuria|Iminoglycinuria, digenic
PRSS1	c.47C>T	p.A16V	het	Hereditary pancreatitis
NTRK1	c.1838G>T	p.G613V	het	Familial medullary thyroid carcinoma|Hereditary insensitivity to pain with anhidrosis
PRSS1	c.161A>G	p.N54S	het	Hereditary pancreatitis
FGFR4	c.1162G>A	p.G388R	het	Cancer progression and tumor cell motility
ITGB3	c.176T>C	p.L59P	het	Thrombocytopenia, neonatal alloimmune|Posttransfusion purpura
TIRAP	c.539C>T	p.S180L	het	Malaria, resistance to|BACTEREMIA, RESISTANCE TO
CST3	c.73G>A	p.A25T	het	Age-related macular degeneration 11
HEXB	c.185T>C	p.L62S	hom	Sandhoff disease, infantile type
BBS2	c.209G>A	p.S70N	hom	Bardet-Biedl syndrome 2

SECONDARY/INCIDENTAL SEQUENCE VARIANT(S) DETECTED

The American College of Medical Genetics and Genomics (ACMG) recently published a policy statement on clinical sequencing that emphasizes the possibility of recognizing and reporting incidental or secondary findings of medical value when conducting exome and genome sequencing. The ACMG recommends that laboratories performing clinical sequencing seek and report mutations of specified classes or types in the genes listed within the guidelines. This section of the report describes variants which fit the criteria outlined by ACMG for the reporting of incidental or secondary findings.

Gene	Nucleotide Change	Protein Change	Zygosity	Tute Dx
MUTYH	c.1276C>T	p.R426C	het	Adenomatous polyposis coli
PMS2	c.59G>A	p.R20Q	het	Colorectal cancer, non-polyposis
BRCA1	c.1067A>G	p.Q356R	het	.
SDHD	c.371A>G	p.Q124R	hom	.
FBN1	c.1415G>A	p.C472Y	hom	.
BRCA2	c.7397T>C	p.V2466A	hom	Breast cancer
MUTYH	c.1014G>C	p.Q338H	het	.
MEN1	c.1636A>G	p.T546A	hom	.
DSG2	c.2318G>A	p.R773K	het	.
APC	c.5465T>A	p.V1822D	hom	.
PMS2	c.1621A>G	p.K541E	hom	Colorectal cancer, non-polyposis
COL3A1	c.4059T>G	p.H1353Q	hom	.
BRCA2	c.1114A>C	p.N372H	het	.
MYLK	c.1486C>G	p.L496V	hom	.
PCSK9	c.1420G>A	p.V474I	hom	.
APOB	c.13013G>A	p.S4338N	hom	.
PCSK9	c.2009G>A	p.G670E	hom	.
DSC2	c.2393G>A	p.R798Q	het	.
APOB	c.6937A>G	p.I2313V	hom	.
MYLK	c.439C>T	p.P147S	hom	.

INTERPRETATION OF PRIMARY FINDINGS

c.845G>A

The patient is homogeneous for a common missense polymorphism in the HFE gene which occurs at a frequency of 0.013% in the 1000 genomes project. OMIM reports 6 phenotypes associated with variants in the HFE gene including Hemochromatosis, [Transferrin serum level QTL2], susceptibility to Alzheimer disease, Microvascular complications of diabetes 7, susceptibility to Porphyria cutanea tarda, susceptibility to Porphyria variegata. This variant causes a nonsynonymous severe amino acid substitution (p.C282Y) which may alter protein function. ClinVar classifies this variant as

acid substitution (p.C282Y) which may alter protein function. ClinVar classifies this variant as pathogenic, other and non-pathogenic and reports an association with Hereditary hemochromatosis, Porphyria cutanea tarda (susceptibility to), Porphyria variegata (susceptibility to), Hemochromatosis (juvenile) (digenic), Alzheimer disease (susceptibility to), Transferrin serum level quantitative trait locus 2, Microvascular complications of diabetes 7, not specified. This variant is predicted to be deleterious by SIFT and deleterious by PolyPhen2.

c.224G>A

The patient is heterozygous for a common missense polymorphism in the CFTR gene which occurs at a frequency of 0.0064% in the 1000 genomes project. OMIM reports 6 phenotypes associated with variants in the CFTR gene including Congenital bilateral absence of vas deferens, Cystic fibrosis, Sweat chloride elevation without CF, modifier of Bronchiectasis with or without elevated sweat chloride 1, Hypertrypsinemia (neonatal), Pancreatitis (idiopathic). This variant causes a nonsynonymous conservative amino acid substitution (p.R75Q) which may alter protein function. ClinVar classifies this variant as probable-non-pathogenic, unknown, non-pathogenic and pathogenic and reports an association with Cystic fibrosis, , not specified, Hereditary pancreatitis;Cystic fibrosis. This variant is predicted to be deleterious by SIFT and deleterious by PolyPhen2.

c.724G>T

The patient is heterozygous for a rare missense mutation in the MKKS gene which occurs at a frequency of 0.0018% in the 1000 genomes project. OMIM reports 2 phenotypes associated with variants in the MKKS gene including Bardet-Biedl syndrome 6, McKusick-Kaufman syndrome. This variant causes a nonsynonymous moderate amino acid substitution (p.A242S) which may alter protein function. ClinVar classifies this variant as pathogenic and reports an association with McKusick Kaufman syndrome. This variant is predicted to be tolerable by SIFT and deleterious by PolyPhen2.

c.1810C>T

The patient is heterozygous for a common missense polymorphism in the NTRK1 gene which occurs at a frequency of 0.024% in the 1000 genomes project. OMIM reports 2 phenotypes associated with variants in the NTRK1 gene including Insensitivity to pain (congenital) (with anhidrosis), Medullary thyroid carcinoma (familial). This variant causes a nonsynonymous moderate amino acid substitution (p.H604Y) which may alter protein function. ClinVar classifies this variant as pathogenic and non-pathogenic and reports an association with Familial medullary thyroid carcinoma, Hereditary insensitivity to pain with anhidrosis, Hereditary insensitivity to pain with anhidrosis. This variant is predicted to be deleterious by SIFT and deleterious by PolyPhen2.

c.9406-1G>T

The patient is heterozygous for a common polymorphism in the VPS13B gene which doesn't occur in the 1000 genomes project. OMIM reports that variants in the VPS13B gene are associated with Cohen syndrome. ClinVar classifies this variant as probable-pathogenic and reports an association with Cohen syndrome.

c.139C>G

The patient is heterozygous for a rare missense mutation in the BRIP1 gene which doesn't occur in the 1000 genomes project. OMIM reports 2 phenotypes associated with variants in the BRIP1 gene including Breast cancer (early-onset), Fanconi anemia (complementation group J). This variant causes a nonsynonymous conservative amino acid substitution (p.P47A) which may alter protein function. ClinVar classifies this variant as pathogenic and other and reports an association with Breast cancer (early-onset), Neoplastic Syndromes (Hereditary). This variant is predicted to be deleterious by PolyPhen2.

c.1601A>G

The patient is heterozygous for a common missense polymorphism in the F5 gene which occurs at a frequency of 0.99% in the 1000 genomes project. OMIM reports 6 phenotypes associated with variants in the F5 gene including Factor V deficiency, Thrombophilia due to activated protein C resistance, Budd-Chiari syndrome, susceptibility to Pregnancy loss recurrent 1, susceptibility to

[illegible]
Stroke (ischemic), susceptibility to Thrombophilia due to factor V Leiden. This variant causes a nonsynonymous conservative amino acid substitution (p.Q534R) which may alter protein function. ClinVar classifies this variant as pathogenic, untested and other and reports an association with Thrombophilia due to factor V Leiden, Ischemic stroke (susceptibility to), Budd-Chiari syndrome (susceptibility to), Recurrent abortion. This variant is predicted to be tolerable by SIFT

c.85C>T
The patient is homogeneous for a common missense polymorphism in the DPYD gene which occurs at a frequency of 0.74% in the 1000 genomes project. OMIM reports 2 phenotypes associated with variants in the DPYD gene including 5-fluorouracil toxicity, Dihydropyrimidine dehydrogenase deficiency. This variant causes a nonsynonymous severe amino acid substitution (p.R29C) which may alter protein function. ClinVar classifies this variant as pathogenic and reports an association with Dihydropyrimidine dehydrogenase deficiency;. This variant is predicted to be tolerable by SIFT and deleterious by PolyPhen2.

c.86A>T
The patient is heterozygous for a common missense polymorphism in the PRSS1 gene which doesn't occur in the 1000 genomes project. OMIM reports 2 phenotypes associated with variants in the PRSS1 gene including Pancreatitis (hereditary), Trypsinogen deficiency. This variant causes a nonsynonymous moderate amino acid substitution (p.N29I) which may alter protein function. ClinVar classifies this variant as pathogenic and reports an association with Hereditary pancreatitis;Hereditary pancreatitis. This variant is predicted to be deleterious by SIFT and benign by PolyPhen2.

c.461G>A
The patient is homogeneous for a common nonsense polymorphism in the FUT2 gene which occurs at a frequency of 0.32% in the 1000 genomes project. OMIM reports 3 phenotypes associated with variants in the FUT2 gene including [Bombay phenotype], resistance to Norwalk virus infection, Vitamin B12 plasma level QTL1. This variant introduces a premature stop codon which may lead to early termination of protein transcription. ClinVar classifies this variant as non-pathogenic, pathogenic and other and reports an association with SECRETOR/NONSECRETOR POLYMORPHISM, Norwalk virus infection (resistance to), Vitamin b12 plasma level quantitative trait locus 1. This variant is predicted to be tolerable by SIFT

c.596C>T
The patient is heterozygous for a common missense polymorphism in the SLC6A20 gene which occurs at a frequency of 0.033% in the 1000 genomes project. OMIM reports 2 phenotypes associated with variants in the SLC6A20 gene including Hyperglycinuria, Iminoglycinuria (digenic). This variant causes a nonsynonymous moderate amino acid substitution (p.T199M) which may alter protein function. ClinVar classifies this variant as pathogenic and reports an association with Hyperglycinuria, Iminoglycinuria (digenic). This variant is predicted to be deleterious by SIFT and deleterious by PolyPhen2.

c.47C>T
The patient is heterozygous for a common missense polymorphism in the PRSS1 gene which doesn't occur in the 1000 genomes project. OMIM reports 2 phenotypes associated with variants in the PRSS1 gene including Pancreatitis (hereditary), Trypsinogen deficiency. This variant causes a nonsynonymous moderate amino acid substitution (p.A16V) which may alter protein function. ClinVar classifies this variant as pathogenic and reports an association with Hereditary pancreatitis. This variant is predicted to be tolerable by SIFT and benign by PolyPhen2.

c.1838G>T
The patient is heterozygous for a common missense polymorphism in the NTRK1 gene which occurs at a frequency of 0.023% in the 1000 genomes project. OMIM reports 2 phenotypes associated with variants in the NTRK1 gene including Insensitivity to pain (congenital) (with anhidrosis), Medullary thyroid carcinoma (familial). This variant causes a nonsynonymous moderate [illegible]

amino acid substitution (p.G613V) which may alter protein function. ClinVar classifies this variant as pathogenic and non-pathogenic and reports an association with Familial medullary thyroid carcinoma, Hereditary insensitivity to pain with anhidrosis, Hereditary insensitivity to pain with anhidrosis. This variant is predicted to be tolerable by SIFT and possibly deleterious by PolyPhen2.

c.161A>G

The patient is heterozygous for a common missense polymorphism in the PRSS1 gene which doesn't occur in the 1000 genomes project. OMIM reports 2 phenotypes associated with variants in the PRSS1 gene including Pancreatitis (hereditary), Trypsinogen deficiency. This variant causes a nonsynonymous conservative amino acid substitution (p.N54S) which may alter protein function. ClinVar classifies this variant as pathogenic and reports an association with Hereditary pancreatitis. This variant is predicted to be tolerable by SIFT and benign by PolyPhen2.

c.1162G>A

The patient is heterozygous for a common missense polymorphism in the FGFR4 gene which occurs at a frequency of 0.3% in the 1000 genomes project. OMIM reports that variants in the FGFR4 gene are associated with Cancer progression/metastasis. This variant causes a nonsynonymous moderate amino acid substitution (p.G388R) which may alter protein function. ClinVar classifies this variant as pathogenic and reports an association with Cancer progression and tumor cell motility. This variant is predicted to be deleterious by SIFT and deleterious by PolyPhen2.

c.176T>C

The patient is heterozygous for a common missense polymorphism in the ITGB3 gene which occurs at a frequency of 0.089% in the 1000 genomes project. OMIM reports 5 phenotypes associated with variants in the ITGB3 gene including Bleeding disorder (platelet-type) (16) (autosomal dominant), Glanzmann thrombasthenia, Purpura (posttransfusion), Thrombocytopenia (neonatal alloimmune), susceptibility to Myocardial infarction. This variant causes a nonsynonymous moderate amino acid substitution (p.L59P) which may alter protein function. ClinVar classifies this variant as non-pathogenic, pathogenic and other and reports an association with PL(A1)/(A2) ALLOANTIGEN POLYMORPHISM, Thrombocytopenia (neonatal alloimmune), Posttransfusion purpura, Myocardial infarction, Fracture (hip) (susceptibility to). This variant is predicted to be tolerable by SIFT and benign by PolyPhen2.

c.539C>T

The patient is heterozygous for a common missense polymorphism in the TIRAP gene which occurs at a frequency of 0.086% in the 1000 genomes project. OMIM reports 4 phenotypes associated with variants in the TIRAP gene including protection against Bacteremia, protection against Malaria, protection against Pneumococcal disease (invasive), protection against Tuberculosis. This variant causes a nonsynonymous moderate amino acid substitution (p.S180L) which may alter protein function. ClinVar classifies this variant as other and pathogenic and reports an association with Invasive pneumococcal disease (protection against), Malaria (resistance to), Mycobacterium tuberculosis (protection against), BACTEREMIA (RESISTANCE TO). This variant is predicted to be possibly deleterious by PolyPhen2.

c.73G>A

The patient is heterozygous for a common missense polymorphism in the CST3 gene which occurs at a frequency of 0.21% in the 1000 genomes project. OMIM reports 2 phenotypes associated with variants in the CST3 gene including Cerebral amyloid angiopathy, Macular degeneration (age-related) (11). This variant causes a nonsynonymous moderate amino acid substitution (p.A25T) which may alter protein function. ClinVar classifies this variant as pathogenic and reports an association with Age-related macular degeneration 11. This variant is predicted to be tolerable by SIFT and benign by PolyPhen2.

c.185T>C

The patient is homogeneous for a common missense polymorphism in the HEXB gene which occurs at a frequency of 0.98% in the 1000 genomes project. OMIM reports that variants in the HEXB gene are associated with Sandhoff disease (infantile) (juvenile) (and adult forms). This variant

HEXB gene are associated with Sandhoff disease (infantile) (juvenile) (and adult forms). This variant causes a nonsynonymous moderate amino acid substitution (p.L62S) which may alter protein function. ClinVar classifies this variant as pathogenic and reports an association with Sandhoff disease (infantile type). This variant is predicted to be tolerable by SIFT

c.209G>A

The patient is homogeneous for a common missense polymorphism in the BBS2 gene which occurs at a frequency of 1.% in the 1000 genomes project. OMIM reports that variants in the BBS2 gene are associated with Bardet-Biedl syndrome 2. This variant causes a nonsynonymous conservative amino acid substitution (p.S70N) which may alter protein function. ClinVar classifies this variant as pathogenic and reports an association with Bardet-Biedl syndrome 2. This variant is predicted to be tolerable by SIFT

INTERPRETATION OF SECONDARY FINDINGS

---

c.1276C>T

The patient is heterozygous for a rare missense mutation in the MUTYH gene which doesn't occur in the 1000 genomes project. OMIM reports 3 phenotypes associated with variants in the MUTYH gene including Adenomas (multiple colorectal), Colorectal adenomatous polyposis (autosomal recessive) (with pilomatricomas), Gastric cancer (somatic). This variant causes a nonsynonymous severe amino acid substitution (p.R426C) which may alter protein function. ClinVar classifies this variant as unknown and other and reports an association with , Neoplastic Syndromes (Hereditary), MYH-associated polyposis. Invitae classifies this variant as Variant of uncertain significance. This variant is predicted to be tolerable by SIFT and deleterious by PolyPhen2.

c.59G>A

The patient is heterozygous for a common missense polymorphism in the PMS2 gene which occurs at a frequency of 0.076% in the 1000 genomes project. OMIM reports 2 phenotypes associated with variants in the PMS2 gene including Colorectal cancer (hereditary nonpolyposis) (type 4), Mismatch repair cancer syndrome. This variant causes a nonsynonymous conservative amino acid substitution (p.R20Q) which may alter protein function. ClinVar classifies this variant as non-pathogenic and untested and reports an association with , Lynch syndrome, not specified, Neoplastic Syndromes (Hereditary). This variant is predicted to be tolerable by SIFT and possibly deleterious by PolyPhen2.

c.1067A>G

The patient is heterozygous for a common missense polymorphism in the BRCA1 gene which occurs at a frequency of 0.022% in the 1000 genomes project. OMIM reports 2 phenotypes associated with variants in the BRCA1 gene including Breast-ovarian cancer (familial) (1), susceptibility to Pancreatic cancer 4. This variant causes a nonsynonymous conservative amino acid substitution (p.Q356R) which may alter protein function. ClinVar classifies this variant as non-pathogenic, other and untested and reports an association with , BRCA1 and BRCA2 Hereditary Breast and Ovarian Cancer, Breast-ovarian cancer (familial 1), not specified, Neoplastic Syndromes (Hereditary). ARUP classifies this variant as 1 - Not pathogenic or of no clinical significance. Invitae classifies this variant as Benign. This variant is predicted to be deleterious by SIFT and deleterious by PolyPhen2.

c.371A>G

The patient is homogeneous for a common missense polymorphism in the SDHD gene which occurs at a frequency of 0.95% in the 1000 genomes project. OMIM reports 6 phenotypes associated with variants in the SDHD gene including Carcinoid tumors (intestinal), Cowden syndrome 3, Merkel cell carcinoma (somatic), Paraganglioma and gastric stromal sarcoma, Paragangliomas 1 (with or without deafness), Pheochromocytoma. This variant causes a nonsynonymous conservative amino acid substitution (p.Q124R) which may alter protein function. This variant is [illegible]

predicted to be tolerable by SIFT

c.1415G>A

The patient is homogeneous for a common missense polymorphism in the FBN1 gene which occurs at a frequency of 1.% in the 1000 genomes project. OMIM reports 8 phenotypes associated with variants in the FBN1 gene including Acromicric dysplasia, Aortic aneurysm (ascending) (and dissection), Ectopia lentis (familial), Geleophysic dysplasia 2, MASS syndrome, Marfan syndrome, Stiff skin syndrome, Weill-Marchesani syndrome 2 (dominant). This variant causes a nonsynonymous severe amino acid substitution (p.C472Y) which may alter protein function. This variant is predicted to be tolerable by SIFT and benign by PolyPhen2.

c.7397T>C

The patient is homogeneous for a common missense polymorphism in the BRCA2 gene which occurs at a frequency of 0.98% in the 1000 genomes project. OMIM reports 9 phenotypes associated with variants in the BRCA2 gene including Fanconi anemia (complementation group D1), Pancreatic cancer, Prostate cancer, Wilms tumor, susceptibility to Breast cancer (male), Breast-ovarian cancer (familial) (2), Glioblastoma 3, Medulloblastoma, Pre-B-cell acute lymphoblastic leukemia. This variant causes a nonsynonymous moderate amino acid substitution (p.V2466A) which may alter protein function. ClinVar classifies this variant as untested, unknown and non-pathogenic and reports an association with Familial cancer of breast, Breast-ovarian cancer (familial 2), not specified. This variant is predicted to be tolerable by SIFT

c.1014G>C

The patient is heterozygous for a common missense polymorphism in the MUTYH gene which occurs at a frequency of 0.31% in the 1000 genomes project. OMIM reports 3 phenotypes associated with variants in the MUTYH gene including Adenomas (multiple colorectal), Colorectal adenomatous polyposis (autosomal recessive) (with pilomatricomas), Gastric cancer (somatic). This variant causes a nonsynonymous conservative amino acid substitution (p.Q338H) which may alter protein function. ClinVar classifies this variant as non-pathogenic and reports an association with , not specified, Neoplastic Syndromes (Hereditary). This variant is predicted to be tolerable by SIFT and possibly deleterious by PolyPhen2.

c.1636A>G

The patient is homogeneous for a common missense polymorphism in the MEN1 gene which occurs at a frequency of 0.83% in the 1000 genomes project. OMIM reports 6 phenotypes associated with variants in the MEN1 gene including Adrenal adenoma (somatic), Angiofibroma (somatic), Carcinoid tumor of lung, Lipoma (somatic), Multiple endocrine neoplasia 1, Parathyroid adenoma (somatic). This variant causes a nonsynonymous moderate amino acid substitution (p.T546A) which may alter protein function. ClinVar classifies this variant as non-pathogenic and reports an association with , not specified. This variant is predicted to be tolerable by SIFT and benign by PolyPhen2.

c.2318G>A

The patient is heterozygous for a common missense polymorphism in the DSG2 gene which occurs at a frequency of 0.24% in the 1000 genomes project. OMIM reports 2 phenotypes associated with variants in the DSG2 gene including Arrhythmogenic right ventricular dysplasia 10, Cardiomyopathy (dilated) (1BB). This variant causes a nonsynonymous conservative amino acid substitution (p.R773K) which may alter protein function. ClinVar classifies this variant as non-pathogenic and reports an association with not specified. This variant is predicted to be tolerable by SIFT and benign by PolyPhen2.

c.5465T>A

The patient is homogeneous for a common missense polymorphism in the APC gene which occurs at a frequency of 0.87% in the 1000 genomes project. OMIM reports 8 phenotypes associated with variants in the APC gene including Adenoma (periampullary) (somatic), Adenomatous polyposis coli, Brain tumor-polyposis syndrome 2, Colorectal cancer (somatic), Desmoid disease (hereditary), Gardner syndrome, Gastric cancer (somatic), Hepatoblastoma (somatic). This variant causes a

Gardner syndrome, Gastric cancer (somatic), Hepatoblastoma (somatic). This variant causes a nonsynonymous severe amino acid substitution (p.V1822D) which may alter protein function. ClinVar classifies this variant as non-pathogenic and other and reports an association with Adenomatous polyposis coli, , not specified, Familial colorectal cancer, Neoplastic Syndromes (Hereditary). This variant is predicted to be benign by PolyPhen2.

c.1621A>G

The patient is homogeneous for a common missense polymorphism in the PMS2 gene which occurs at a frequency of 0.88% in the 1000 genomes project. OMIM reports 2 phenotypes associated with variants in the PMS2 gene including Colorectal cancer (hereditary nonpolyposis) (type 4), Mismatch repair cancer syndrome. This variant causes a nonsynonymous moderate amino acid substitution (p.K541E) which may alter protein function. ClinVar classifies this variant as non-pathogenic and reports an association with Lynch syndrome. This variant is predicted to be tolerable by SIFT and benign by PolyPhen2.

c.4059T>G

The patient is homogeneous for a common missense polymorphism in the COL3A1 gene which occurs at a frequency of 1.% in the 1000 genomes project. OMIM reports 2 phenotypes associated with variants in the COL3A1 gene including Ehlers-Danlos syndrome (type III), Ehlers-Danlos syndrome (type IV). This variant causes a nonsynonymous conservative amino acid substitution (p.H1353Q) which may alter protein function. This variant is predicted to be tolerable by SIFT and benign by PolyPhen2.

c.1114A>C

The patient is heterozygous for a common missense polymorphism in the BRCA2 gene which occurs at a frequency of 0.25% in the 1000 genomes project. OMIM reports 9 phenotypes associated with variants in the BRCA2 gene including Fanconi anemia (complementation group D1), Pancreatic cancer, Prostate cancer, Wilms tumor, susceptibility to Breast cancer (male), Breast-ovarian cancer (familial) (2), Glioblastoma 3, Medulloblastoma, Pre-B-cell acute lymphoblastic leukemia. This variant causes a nonsynonymous moderate amino acid substitution (p.N372H) which may alter protein function. ClinVar classifies this variant as other, non-pathogenic and untested and reports an association with Breast-ovarian cancer (familial 2), , not specified, Neoplastic Syndromes (Hereditary). This variant is predicted to be tolerable by SIFT

c.1486C>G

The patient is homogeneous for a common missense polymorphism in the MYLK gene which occurs at a frequency of 1.% in the 1000 genomes project. OMIM reports that variants in the MYLK gene are associated with Aortic aneurysm (familial thoracic 7). This variant causes a nonsynonymous conservative amino acid substitution (p.L496V) which may alter protein function. This variant is predicted to be tolerable by SIFT and benign by PolyPhen2.

c.1420G>A

The patient is homogeneous for a common missense polymorphism in the PCSK9 gene which occurs at a frequency of 0.87% in the 1000 genomes project. OMIM reports 2 phenotypes associated with variants in the PCSK9 gene including Hypercholesterolemia (familial) (3), Low density lipoprotein cholesterol level QTL 1. This variant causes a nonsynonymous conservative amino acid substitution (p.V474I) which may alter protein function. This variant is predicted to be tolerable by SIFT and benign by PolyPhen2.

c.13013G>A

The patient is homogeneous for a common missense polymorphism in the APOB gene which occurs at a frequency of 0.63% in the 1000 genomes project. OMIM reports 2 phenotypes associated with variants in the APOB gene including Hypercholesterolemia (due to ligand-defective apo B), Hypobetalipoproteinemia. This variant causes a nonsynonymous conservative amino acid substitution (p.S4338N) which may alter protein function. ClinVar classifies this variant as probable-non-pathogenic and reports an association with not specified. This variant is predicted to be tolerable by SIFT and benign by PolyPhen2.

[illegible]

c.2009G>A

The patient is homogeneous for a common missense polymorphism in the PCSK9 gene which occurs at a frequency of 0.9% in the 1000 genomes project. OMIM reports 2 phenotypes associated with variants in the PCSK9 gene including Hypercholesterolemia (familial) (3), Low density lipoprotein cholesterol level QTL 1. This variant causes a nonsynonymous moderate amino acid substitution (p.G670E) which may alter protein function. ClinVar classifies this variant as non-pathogenic and reports an association with Familial hypercholesterolemia. This variant is predicted to be tolerable by SIFT and benign by PolyPhen2.

c.2393G>A

The patient is heterozygous for a common missense polymorphism in the DSC2 gene which occurs at a frequency of 0.028% in the 1000 genomes project. OMIM reports 2 phenotypes associated with variants in the DSC2 gene including Arrhythmogenic right ventricular dysplasia 11 with mild palmoplantar keratoderma and woolly hair, Arrhythmogenic right ventricular dysplasia 11. This variant causes a nonsynonymous conservative amino acid substitution (p.R798Q) which may alter protein function. ClinVar classifies this variant as unknown and reports an association with not specified;Arrhythmogenic right ventricular cardiomyopathy, not specified. This variant is predicted to be tolerable by SIFT and benign by PolyPhen2.

c.6937A>G

The patient is homogeneous for a common missense polymorphism in the APOB gene which occurs at a frequency of 0.99% in the 1000 genomes project. OMIM reports 2 phenotypes associated with variants in the APOB gene including Hypercholesterolemia (due to ligand-defective apo B), Hypobetalipoproteinemia. This variant causes a nonsynonymous conservative amino acid substitution (p.I2313V) which may alter protein function. This variant is predicted to be tolerable by SIFT and benign by PolyPhen2.

c.439C>T

The patient is homogeneous for a common missense polymorphism in the MYLK gene which occurs at a frequency of 0.86% in the 1000 genomes project. OMIM reports that variants in the MYLK gene are associated with Aortic aneurysm (familial thoracic 7). This variant causes a nonsynonymous moderate amino acid substitution (p.P147S) which may alter protein function. This variant is predicted to be tolerable by SIFT and benign by PolyPhen2.

RECOMMENDATIONS

Genetic counseling of the family is recommended to discuss the implications of this report.
[Additional recommendations.]

METHODS AND LIMITATIONS

This report is a sample to be used solely for the purpose of evaluating functionality of the Tute Platform and should not be used in any clinical application.

Sequencing and Variant Detection

Whole exome sequencing was performed by solution-based hybridization which enriches for coding exons targeting DNA with specific baits. Sequencing was performed using the Illumina HiSeq 2500 to a minimum average exome depth of 90X. Sequencing reads were aligned to the reference genome (hg19) by BWA-MEM and variants were called using FreeBayes.

Variant Analysis and Report Generation

Reported variants were analyzed using the Tute platform to identify those variants which are reported by medical literature to be pathogenic. All non-synonymous variants of allele frequency <1% reported by the 1000 genomes project and annotated as ‘Pathogenic’ or ‘Probably Pathogenic’ by ClinVar are reported in the table ‘Primary/Diagnostic Sequence Variant(s) Detected’. Careful correlation of these variants with the clinical indications for the proband should be conducted by an experience medical geneticist to verify that reported findings are consistent with reported clinical phenotypes. Additionally, variants are included based on ACMG guidelines for the reporting of incidental findings in the table ‘Secondary/Incidental Sequence Variant(s) Detected’.

Validation of reported findings

Variants of interest should be confirmed by Sanger sequencing. The DNA immediately flanking each of these variants should be amplified genomic DNA followed by sequencing in both directions using fluorescent dideoxy sequencing.

Limitations

Absence of a primary diagnostic finding identified by this test does not exclude the possibility of a genetic basis for the clinical condition for this proband. Certain genetic abnormalities are not detectable by the technologies used to generate this report. Specifically, detection of abnormal variants depends on the presence of these sequence variants in the sequencing data. This exome sequence test is designed to evaluate single nucleotide variants within the human exome; however, this technology is only able to sequence 90-95% of the human reference to the requisite 10-fold coverage needed for reliable detection of heterozygous variants.

Additionally, certain types of genetic abnormalities are difficult to identify in sequencing data and have not been validated for clinical use including insertions, deletions, copy number alterations, long repetitive sequences, triplet repeat expansions, chromosomal rearrangements, polyploidy, repetitive regions including mono-, di- and tri-nucleotide repeats, GX rich regions, intronic variants outside the splice-site and epigenetic effects.

Variants believed to be benign based on medical literature, or with population frequencies greater than or equal to 1%, or resulting in synonymous amino acid changes, or occurring in 5’ or 3’ untranslated regions are generally not reported.

This report is a sample to be used solely for the purpose of evaluating functionality of the Tute Platform and should not be used in any clinical application.

Sources

REPORT SIGNATURES

Online Mendelian Inheritance in Man, OMIM (TM). Retrieved 0/26/2015 from http://www.ncbi.nlm.nih.gov/omim/
ClinVar. Retrieved 0/26/2015 from http://www.ncbi.nlm.nih.gov/clinvar/

TUTE GENOMICS

Whole Exome Sequencing (System Default) report for K10031 10138 annotation

ORDERING PROVIDER

,

SPECIMEN

Collection Date:
Received Date:
Report Date:

PATIENT

Name:
Date of Birth:
Patient ID:
Gender:

Clinical Indication:

[Detailed clinical description.]

TEST RESULTS SUMMARY

Primary Result(s): [Findings related to clinical indication.]

Incidental/Secondary Result(s): [Incidental findings.]

PRIMARY/DIAGNOSTIC SEQUENCE VARIANT(S) DETECTED

Gene	Nucleotide Change	Protein Change	Zygosity	Tute Dx
HFE	c.845G>A	p.C282Y	het	Hereditary hemochromatosis|Hemochromatosis, juvenile, digenic
LYST	c.2413delG	p.E805fs	het	Chédiak-Higashi syndrome
NTRK1	c.1810C>T	p.H604Y	het	Familial medullary thyroid carcinoma|Hereditary insensitivity to pain with anhidrosis
VPS13B	c.9406-1G>T	.	het	Cohen syndrome
BRIP1	c.139C>G	p.P47A	het	Breast cancer, early-onset
F5	c.1601A>G	p.Q534R	hom	Thrombophilia due to factor V Leiden
DPYD	c.85C>T	p.R29C	hom	Dihydropyrimidine dehydrogenase deficiency;not provided
DCLRE1C	c.97G>A	p.G33R	het	Severe combined immunodeficiency disease
PRSS1	c.86A>T	p.N29I	het	Hereditary pancreatitis;Hereditary pancreatitis
FUT2	c.461G>A	p.W154X	het	Norwalk virus infection, resistance to
NTRK1	c.1838G>T	p.G613V	het	Familial medullary thyroid carcinoma|Hereditary insensitivity to pain with anhidrosis
PRSS1	c.161A>G	p.N54S	het	Hereditary pancreatitis
ELAC2	c.1621G>A	p.A541T	het	Prostate cancer, hereditary, 2
OCA2	c.913C>T	p.R305W	het	Skin/hair/eye pigmentation, variation in, 1
CYP2B6	c.785A>G	p.K262R	het	Efavirenz, poor metabolism of
ITGB3	c.176T>C	p.L59P	hom	Thrombocytopenia, neonatal alloimmune|Posttransfusion purpura
TIRAP	c.539C>T	p.S180L	het	Malaria, resistance to|BACTEREMIA, RESISTANCE TO
CST3	c.73G>A	p.A25T	het	Age-related macular degeneration 11
HEXB	c.185T>C	p.L62S	hom	Sandhoff disease, infantile type
BBS2	c.209G>A	p.S70N	hom	Bardet-Biedl syndrome 2

SECONDARY/INCIDENTAL SEQUENCE VARIANT(S) DETECTED

The American College of Medical Genetics and Genomics (ACMG) recently published a policy statement on clinical sequencing that emphasizes the possibility of recognizing and reporting incidental or secondary findings of medical value when conducting exome and genome sequencing. The ACMG recommends that laboratories performing clinical sequencing seek and report mutations of specified classes or types in the genes listed within the guidelines. This section of the report describes variants which fit the criteria outlined by ACMG for the reporting of incidental or secondary findings.

Gene	Nucleotide Change	Protein Change	Zygosity	Tute Dx
RYR1	c.7336G>T	p.G2446C	het	.
PMS2	c.59G>A	p.R20Q	het	Colorectal cancer, non-polyposis
PMS2	c.1531A>G	p.T511A	het	.
SDHD	c.371A>G	p.Q124R	hom	.
FBN1	c.1415G>A	p.C472Y	hom	.
SCN5A	c.1673A>G	p.H558R	het	Progressive familial heart block type 1A
DSC2	c.2326A>G	p.I776V	het	.
TP53	c.215C>G	p.P72R	het	.
BRCA2	c.7397T>C	p.V2466A	hom	Breast cancer
MUTYH	c.1014G>C	p.Q338H	het	.
MEN1	c.1636A>G	p.T546A	hom	.
DSG2	c.2318G>A	p.R773K	hom	.
APC	c.5465T>A	p.V1822D	hom	.
PMS2	c.1621A>G	p.K541E	hom	Colorectal cancer, non-polyposis
COL3A1	c.4059T>G	p.H1353Q	hom	.
BRCA2	c.1114A>C	p.N372H	het	.
MYLK	c.1486C>G	p.L496V	hom	.
PCSK9	c.1420G>A	p.V474I	hom	.
APOB	c.13013G>A	p.S4338N	hom	.
PCSK9	c.2009G>A	p.G670E	hom	.

INTERPRETATION OF PRIMARY FINDINGS

c.845G>A

The patient is heterozygous for a common missense polymorphism in the HFE gene which occurs at a frequency of 0.013% in the 1000 genomes project. OMIM reports 6 phenotypes associated with

[illegible]
variants in the HFE gene including Hemochromatosis, [Transferrin serum level QTL2], susceptibility to Alzheimer disease, Microvascular complications of diabetes 7, susceptibility to Porphyria cutanea tarda, susceptibility to Porphyria variegata. This variant causes a nonsynonymous severe amino acid substitution (p.C282Y) which may alter protein function. ClinVar classifies this variant as pathogenic, other and non-pathogenic and reports an association with Hereditary hemochromatosis, Porphyria cutanea tarda (susceptibility to), Porphyria variegata (susceptibility to), Hemochromatosis (juvenile) (digenic), Alzheimer disease (susceptibility to), Transferrin serum level quantitative trait locus 2, Microvascular complications of diabetes 7, not specified. This variant is predicted to be deleterious by SIFT and deleterious by PolyPhen2.

c.2413delG

The patient is heterozygous for a rare mutation in the LYST gene which doesn't occur in the 1000 genomes project. OMIM reports that variants in the LYST gene are associated with Chediak-Higashi syndrome. This variant causes a frameshift which may significantly alter protein expression or function. ClinVar classifies this variant as pathogenic and reports an association with Chédiak-Higashi syndrome.

c.1810C>T

The patient is heterozygous for a common missense polymorphism in the NTRK1 gene which occurs at a frequency of 0.024% in the 1000 genomes project. OMIM reports 2 phenotypes associated with variants in the NTRK1 gene including Insensitivity to pain (congenital) (with anhidrosis), Medullary thyroid carcinoma (familial). This variant causes a nonsynonymous moderate amino acid substitution (p.H604Y) which may alter protein function. ClinVar classifies this variant as pathogenic and non-pathogenic and reports an association with Familial medullary thyroid carcinoma, Hereditary insensitivity to pain with anhidrosis, Hereditary insensitivity to pain with anhidrosis. This variant is predicted to be deleterious by SIFT and deleterious by PolyPhen2.

c.9406-1G>T

The patient is heterozygous for a common polymorphism in the VPS13B gene which doesn't occur in the 1000 genomes project. OMIM reports that variants in the VPS13B gene are associated with Cohen syndrome. ClinVar classifies this variant as probable-pathogenic and reports an association with Cohen syndrome.

c.139C>G

The patient is heterozygous for a rare missense mutation in the BRIP1 gene which doesn't occur in the 1000 genomes project. OMIM reports 2 phenotypes associated with variants in the BRIP1 gene including Breast cancer (early-onset), Fanconi anemia (complementation group J). This variant causes a nonsynonymous conservative amino acid substitution (p.P47A) which may alter protein function. ClinVar classifies this variant as pathogenic and other and reports an association with Breast cancer (early-onset), Neoplastic Syndromes (Hereditary). This variant is predicted to be deleterious by PolyPhen2.

c.1601A>G

The patient is homogeneous for a common missense polymorphism in the F5 gene which occurs at a frequency of 0.99% in the 1000 genomes project. OMIM reports 6 phenotypes associated with variants in the F5 gene including Factor V deficiency, Thrombophilia due to activated protein C resistance, Budd-Chiari syndrome, susceptibility to Pregnancy loss recurrent 1, susceptibility to Stroke (ischemic), susceptibility to Thrombophilia due to factor V Leiden. This variant causes a nonsynonymous conservative amino acid substitution (p.Q534R) which may alter protein function. ClinVar classifies this variant as pathogenic, untested and other and reports an association with Thrombophilia due to factor V Leiden, Ischemic stroke (susceptibility to), Budd-Chiari syndrome (susceptibility to), Recurrent abortion. This variant is predicted to be tolerable by SIFT

c.85C>T

The patient is homogeneous for a common missense polymorphism in the DPYD gene which occurs at a frequency of 0.74% in the 1000 genomes project. OMIM reports 2 phenotypes
[illegible]

associated with variants in the DPYD gene including 5-fluorouracil toxicity, Dihydropyrimidine dehydrogenase deficiency. This variant causes a nonsynonymous severe amino acid substitution (p.R29C) which may alter protein function. ClinVar classifies this variant as pathogenic and reports an association with Dihydropyrimidine dehydrogenase deficiency;. This variant is predicted to be tolerable by SIFT and deleterious by PolyPhen2.

c.97G>A

The patient is heterozygous for a common missense polymorphism in the DCLRE1C gene which occurs at a frequency of 0.0062% in the 1000 genomes project. OMIM reports 2 phenotypes associated with variants in the DCLRE1C gene including Omenn syndrome, Severe combined immunodeficiency (Athabascan type). This variant causes a nonsynonymous moderate amino acid substitution (p.G33R) which may alter protein function. ClinVar classifies this variant as probable-pathogenic and reports an association with Severe combined immunodeficiency disease. This variant is predicted to be deleterious by SIFT and deleterious by PolyPhen2.

c.86A>T

The patient is heterozygous for a common missense polymorphism in the PRSS1 gene which doesn't occur in the 1000 genomes project. OMIM reports 2 phenotypes associated with variants in the PRSS1 gene including Pancreatitis (hereditary), Trypsinogen deficiency. This variant causes a nonsynonymous moderate amino acid substitution (p.N29I) which may alter protein function. ClinVar classifies this variant as pathogenic and reports an association with Hereditary pancreatitis;Hereditary pancreatitis. This variant is predicted to be deleterious by SIFT and benign by PolyPhen2.

c.461G>A

The patient is heterozygous for a common nonsense polymorphism in the FUT2 gene which occurs at a frequency of 0.32% in the 1000 genomes project. OMIM reports 3 phenotypes associated with variants in the FUT2 gene including [Bombay phenotype], resistance to Norwalk virus infection, Vitamin B12 plasma level QTL1. This variant introduces a premature stop codon which may lead to early termination of protein transcription. ClinVar classifies this variant as non-pathogenic, pathogenic and other and reports an association with SECRETOR/NONSECRETOR POLYMORPHISM, Norwalk virus infection (resistance to), Vitamin b12 plasma level quantitative trait locus 1. This variant is predicted to be tolerable by SIFT

c.1838G>T

The patient is heterozygous for a common missense polymorphism in the NTRK1 gene which occurs at a frequency of 0.023% in the 1000 genomes project. OMIM reports 2 phenotypes associated with variants in the NTRK1 gene including Insensitivity to pain (congenital) (with anhidrosis), Medullary thyroid carcinoma (familial). This variant causes a nonsynonymous moderate amino acid substitution (p.G613V) which may alter protein function. ClinVar classifies this variant as pathogenic and non-pathogenic and reports an association with Familial medullary thyroid carcinoma, Hereditary insensitivity to pain with anhidrosis, Hereditary insensitivity to pain with anhidrosis. This variant is predicted to be tolerable by SIFT and possibly deleterious by PolyPhen2.

c.161A>G

The patient is heterozygous for a common missense polymorphism in the PRSS1 gene which doesn't occur in the 1000 genomes project. OMIM reports 2 phenotypes associated with variants in the PRSS1 gene including Pancreatitis (hereditary), Trypsinogen deficiency. This variant causes a nonsynonymous conservative amino acid substitution (p.N54S) which may alter protein function. ClinVar classifies this variant as pathogenic and reports an association with Hereditary pancreatitis. This variant is predicted to be tolerable by SIFT and benign by PolyPhen2.

c.1621G>A

The patient is heterozygous for a common missense polymorphism in the ELAC2 gene which occurs at a frequency of 0.023% in the 1000 genomes project. OMIM reports 2 phenotypes associated with variants in the ELAC2 gene including Combined oxidative phosphorylation deficiency 17, susceptibility to Prostate cancer (hereditary) (2). This variant causes a nonsynonymous

17), susceptibility to Prostate cancer (hereditary) (2). This variant causes a nonsynonymous moderate amino acid substitution (p.A541T) which may alter protein function. ClinVar classifies this variant as pathogenic and reports an association with Prostate cancer (hereditary) (2). This variant is predicted to be tolerable by SIFT and possibly deleterious by PolyPhen2.

c.913C>T

The patient is heterozygous for a common missense polymorphism in the OCA2 gene which occurs at a frequency of 0.083% in the 1000 genomes project. OMIM reports 4 phenotypes associated with variants in the OCA2 gene including Albinism (brown oculocutaneous), Albinism (oculocutaneous) (type II), [Skin/hair/eye pigmentation 1 (blond)/brown hair], [Skin/hair/eye pigmentation 1 (blue)/nonblue eyes]. This variant causes a nonsynonymous moderate amino acid substitution (p.R305W) which may alter protein function. ClinVar classifies this variant as pathogenic and reports an association with Skin/hair/eye pigmentation (variation in) (1). This variant is predicted to be deleterious by SIFT and possibly deleterious by PolyPhen2.

c.785A>G

The patient is heterozygous for a common missense polymorphism in the CYP2B6 gene which doesn't occur in the 1000 genomes project. OMIM reports 2 phenotypes associated with variants in the CYP2B6 gene including poor metabolism of Efavirenz, susceptibility to Efavirenz central nervous system toxicity. This variant causes a nonsynonymous conservative amino acid substitution (p.K262R) which may alter protein function. ClinVar classifies this variant as pathogenic and reports an association with Efavirenz (poor metabolism of). This variant is predicted to be tolerable by SIFT and benign by PolyPhen2.

c.176T>C

The patient is homogeneous for a common missense polymorphism in the ITGB3 gene which occurs at a frequency of 0.089% in the 1000 genomes project. OMIM reports 5 phenotypes associated with variants in the ITGB3 gene including Bleeding disorder (platelet-type) (16) (autosomal dominant), Glanzmann thrombasthenia, Purpura (posttransfusion), Thrombocytopenia (neonatal alloimmune), susceptibility to Myocardial infarction. This variant causes a nonsynonymous moderate amino acid substitution (p.L59P) which may alter protein function. ClinVar classifies this variant as non-pathogenic, pathogenic and other and reports an association with PL(A1)/(A2) ALLOANTIGEN POLYMORPHISM, Thrombocytopenia (neonatal alloimmune), Posttransfusion purpura, Myocardial infarction, Fracture (hip) (susceptibility to). This variant is predicted to be tolerable by SIFT and benign by PolyPhen2.

c.539C>T

The patient is heterozygous for a common missense polymorphism in the TIRAP gene which occurs at a frequency of 0.086% in the 1000 genomes project. OMIM reports 4 phenotypes associated with variants in the TIRAP gene including protection against Bacteremia, protection against Malaria, protection against Pneumococcal disease (invasive), protection against Tuberculosis. This variant causes a nonsynonymous moderate amino acid substitution (p.S180L) which may alter protein function. ClinVar classifies this variant as other and pathogenic and reports an association with Invasive pneumococcal disease (protection against), Malaria (resistance to), Mycobacterium tuberculosis (protection against), BACTEREMIA (RESISTANCE TO). This variant is predicted to be possibly deleterious by PolyPhen2.

c.73G>A

The patient is heterozygous for a common missense polymorphism in the CST3 gene which occurs at a frequency of 0.21% in the 1000 genomes project. OMIM reports 2 phenotypes associated with variants in the CST3 gene including Cerebral amyloid angiopathy, Macular degeneration (age-related) (11). This variant causes a nonsynonymous moderate amino acid substitution (p.A25T) which may alter protein function. ClinVar classifies this variant as pathogenic and reports an association with Age-related macular degeneration 11. This variant is predicted to be tolerable by SIFT and benign by PolyPhen2.

c.185T>C

The patient is homogeneous for a common missense polymorphism in the HEXB gene which occurs at a frequency of 0.98% in the 1000 genomes project. OMIM reports that variants in the HEXB gene are associated with Sandhoff disease (infantile) (juvenile) (and adult forms). This variant causes a nonsynonymous moderate amino acid substitution (p.L62S) which may alter protein function. ClinVar classifies this variant as pathogenic and reports an association with Sandhoff disease (infantile type). This variant is predicted to be tolerable by SIFT

c.209G>A

The patient is homogeneous for a common missense polymorphism in the BBS2 gene which occurs at a frequency of 1.% in the 1000 genomes project. OMIM reports that variants in the BBS2 gene are associated with Bardet-Biedl syndrome 2. This variant causes a nonsynonymous conservative amino acid substitution (p.S70N) which may alter protein function. ClinVar classifies this variant as pathogenic and reports an association with Bardet-Biedl syndrome 2. This variant is predicted to be tolerable by SIFT

INTERPRETATION OF SECONDARY FINDINGS

---

c.7336G>T

The patient is heterozygous for a rare mutation in the RYR1 gene which doesn't occur in the 1000 genomes project. OMIM reports 5 phenotypes associated with variants in the RYR1 gene including Central core disease, King-Denborough syndrome, Minicore myopathy with external ophthalmoplegia, Neuromuscular disease (congenital) (with uniform type 1 fiber), Malignant hyperthermia susceptibility 1. This variant causes a nonsynonymous severe amino acid substitution (p.G2446C) which may alter protein function. This variant is predicted to be deleterious by SIFT and benign by PolyPhen2.

c.59G>A

The patient is heterozygous for a common missense polymorphism in the PMS2 gene which occurs at a frequency of 0.076% in the 1000 genomes project. OMIM reports 2 phenotypes associated with variants in the PMS2 gene including Colorectal cancer (hereditary nonpolyposis) (type 4), Mismatch repair cancer syndrome. This variant causes a nonsynonymous conservative amino acid substitution (p.R20Q) which may alter protein function. ClinVar classifies this variant as non-pathogenic and untested and reports an association with , Lynch syndrome, not specified, Neoplastic Syndromes (Hereditary). This variant is predicted to be tolerable by SIFT and possibly deleterious by PolyPhen2.

c.1531A>G

The patient is heterozygous for a common missense polymorphism in the PMS2 gene which occurs at a frequency of 0.011% in the 1000 genomes project. OMIM reports 2 phenotypes associated with variants in the PMS2 gene including Colorectal cancer (hereditary nonpolyposis) (type 4), Mismatch repair cancer syndrome. This variant causes a nonsynonymous moderate amino acid substitution (p.T511A) which may alter protein function. ClinVar classifies this variant as non-pathogenic and untested and reports an association with , Lynch syndrome, not specified, Neoplastic Syndromes (Hereditary). Invitae classifies this variant as Benign. This variant is predicted to be tolerable by SIFT and benign by PolyPhen2.

c.371A>G

The patient is homogeneous for a common missense polymorphism in the SDHD gene which occurs at a frequency of 0.95% in the 1000 genomes project. OMIM reports 6 phenotypes associated with variants in the SDHD gene including Carcinoid tumors (intestinal), Cowden syndrome 3, Merkel cell carcinoma (somatic), Paraganglioma and gastric stromal sarcoma, Paragangliomas 1 (with or without deafness), Pheochromocytoma. This variant causes a nonsynonymous conservative amino acid substitution (p.Q124R) which may alter protein function. This variant is predicted to be tolerable by SIFT

predicted to be tolerable by SIFT

c.1415G>A

The patient is homogeneous for a common missense polymorphism in the FBN1 gene which occurs at a frequency of 1.% in the 1000 genomes project. OMIM reports 8 phenotypes associated with variants in the FBN1 gene including Acromicric dysplasia, Aortic aneurysm (ascending) (and dissection), Ectopia lentis (familial), Geleophysic dysplasia 2, MASS syndrome, Marfan syndrome, Stiff skin syndrome, Weill-Marchesani syndrome 2 (dominant). This variant causes a nonsynonymous severe amino acid substitution (p.C472Y) which may alter protein function. This variant is predicted to be tolerable by SIFT and benign by PolyPhen2.

c.1673A>G

The patient is heterozygous for a common missense polymorphism in the SCN5A gene which occurs at a frequency of 0.23% in the 1000 genomes project. OMIM reports 9 phenotypes associated with variants in the SCN5A gene including Atrial fibrillation (familial) (10), Brugada syndrome 1, Cardiomyopathy (dilated) (1E), Heart block (nonprogressive), Heart block (progressive) (type IA), Long QT syndrome-3, Sick sinus syndrome 1, Ventricular fibrillation (familial) (1), susceptibility to Sudden infant death syndrome. This variant causes a nonsynonymous conservative amino acid substitution (p.H558R) which may alter protein function. ClinVar classifies this variant as pathogenic and non-pathogenic and reports an association with Progressive familial heart block type 1A, not specified. This variant is predicted to be tolerable by SIFT and benign by PolyPhen2.

c.2326A>G

The patient is heterozygous for a common missense polymorphism in the DSC2 gene which occurs at a frequency of 0.2% in the 1000 genomes project. OMIM reports 2 phenotypes associated with variants in the DSC2 gene including Arrhythmogenic right ventricular dysplasia 11 with mild palmoplantar keratoderma and woolly hair, Arrhythmogenic right ventricular dysplasia 11. This variant causes a nonsynonymous conservative amino acid substitution (p.I776V) which may alter protein function. ClinVar classifies this variant as non-pathogenic and reports an association with not specified. This variant is predicted to be tolerable by SIFT and benign by PolyPhen2.

c.215C>G

The patient is heterozygous for a common missense polymorphism in the TP53 gene which occurs at a frequency of 0.54% in the 1000 genomes project. OMIM reports 11 phenotypes associated with variants in the TP53 gene including Adrenal cortical carcinoma, Breast cancer, Choroid plexus papilloma, Colorectal cancer, Hepatocellular carcinoma, Li-Fraumeni syndrome, Nasopharyngeal carcinoma, Osteosarcoma, Pancreatic cancer, Basal cell carcinoma 7, Glioma susceptibility 1. This variant causes a nonsynonymous moderate amino acid substitution (p.P72R) which may alter protein function. ClinVar classifies this variant as non-pathogenic and reports an association with CODON 72 POLYMORPHISM ( )(rs1042522), , not specified, Neoplastic Syndromes (Hereditary). This variant is predicted to be tolerable by SIFT and possibly deleterious by PolyPhen2.

c.7397T>C

The patient is homogeneous for a common missense polymorphism in the BRCA2 gene which occurs at a frequency of 0.98% in the 1000 genomes project. OMIM reports 9 phenotypes associated with variants in the BRCA2 gene including Fanconi anemia (complementation group D1), Pancreatic cancer, Prostate cancer, Wilms tumor, susceptibility to Breast cancer (male), Breast-ovarian cancer (familial) (2), Glioblastoma 3, Medulloblastoma, Pre-B-cell acute lymphoblastic leukemia. This variant causes a nonsynonymous moderate amino acid substitution (p.V2466A) which may alter protein function. ClinVar classifies this variant as untested, unknown and non-pathogenic and reports an association with Familial cancer of breast, Breast-ovarian cancer (familial 2), not specified. This variant is predicted to be tolerable by SIFT

c.1014G>C

The patient is heterozygous for a common missense polymorphism in the MUTYH gene which occurs at a frequency of 0.31% in the 1000 genomes project. OMIM reports 3 phenotypes associated with variants in the MUTYH gene including Adenomas (multiple colorectal), Colorectal

[illegible]
adenomatous polyposis (autosomal recessive) (with pilomatricomas), Gastric cancer (somatic). This variant causes a nonsynonymous conservative amino acid substitution (p.Q338H) which may alter protein function. ClinVar classifies this variant as non-pathogenic and reports an association with , not specified, Neoplastic Syndromes (Hereditary). This variant is predicted to be tolerable by SIFT and possibly deleterious by PolyPhen2.

c.1636A>G

The patient is homogeneous for a common missense polymorphism in the MEN1 gene which occurs at a frequency of 0.83% in the 1000 genomes project. OMIM reports 6 phenotypes associated with variants in the MEN1 gene including Adrenal adenoma (somatic), Angiofibroma (somatic), Carcinoid tumor of lung, Lipoma (somatic), Multiple endocrine neoplasia 1, Parathyroid adenoma (somatic). This variant causes a nonsynonymous moderate amino acid substitution (p.T546A) which may alter protein function. ClinVar classifies this variant as non-pathogenic and reports an association with , not specified. This variant is predicted to be tolerable by SIFT and benign by PolyPhen2.

c.2318G>A

The patient is homogeneous for a common missense polymorphism in the DSG2 gene which occurs at a frequency of 0.24% in the 1000 genomes project. OMIM reports 2 phenotypes associated with variants in the DSG2 gene including Arrhythmogenic right ventricular dysplasia 10, Cardiomyopathy (dilated) (1BB). This variant causes a nonsynonymous conservative amino acid substitution (p.R773K) which may alter protein function. ClinVar classifies this variant as non-pathogenic and reports an association with not specified. This variant is predicted to be tolerable by SIFT and benign by PolyPhen2.

c.5465T>A

The patient is homogeneous for a common missense polymorphism in the APC gene which occurs at a frequency of 0.87% in the 1000 genomes project. OMIM reports 8 phenotypes associated with variants in the APC gene including Adenoma (periampullary) (somatic), Adenomatous polyposis coli, Brain tumor-polyposis syndrome 2, Colorectal cancer (somatic), Desmoid disease (hereditary), Gardner syndrome, Gastric cancer (somatic), Hepatoblastoma (somatic). This variant causes a nonsynonymous severe amino acid substitution (p.V1822D) which may alter protein function. ClinVar classifies this variant as non-pathogenic and other and reports an association with Adenomatous polyposis coli, , not specified, Familial colorectal cancer, Neoplastic Syndromes (Hereditary). This variant is predicted to be benign by PolyPhen2.

c.1621A>G

The patient is homogeneous for a common missense polymorphism in the PMS2 gene which occurs at a frequency of 0.88% in the 1000 genomes project. OMIM reports 2 phenotypes associated with variants in the PMS2 gene including Colorectal cancer (hereditary nonpolyposis) (type 4), Mismatch repair cancer syndrome. This variant causes a nonsynonymous moderate amino acid substitution (p.K541E) which may alter protein function. ClinVar classifies this variant as non-pathogenic and reports an association with Lynch syndrome. This variant is predicted to be tolerable by SIFT and benign by PolyPhen2.

c.4059T>G

The patient is homogeneous for a common missense polymorphism in the COL3A1 gene which occurs at a frequency of 1.% in the 1000 genomes project. OMIM reports 2 phenotypes associated with variants in the COL3A1 gene including Ehlers-Danlos syndrome (type III), Ehlers-Danlos syndrome (type IV). This variant causes a nonsynonymous conservative amino acid substitution (p.H1353Q) which may alter protein function. This variant is predicted to be tolerable by SIFT and benign by PolyPhen2.

c.1114A>C

The patient is heterozygous for a common missense polymorphism in the BRCA2 gene which occurs at a frequency of 0.25% in the 1000 genomes project. OMIM reports 9 phenotypes
[illegible]

associated with variants in the BRCA2 gene including Fanconi anemia (complementation group D1), Pancreatic cancer, Prostate cancer, Wilms tumor, susceptibility to Breast cancer (male), Breast-ovarian cancer (familial) (2), Glioblastoma 3, Medulloblastoma, Pre-B-cell acute lymphoblastic leukemia. This variant causes a nonsynonymous moderate amino acid substitution (p.N372H) which may alter protein function. ClinVar classifies this variant as other, non-pathogenic and untested and reports an association with Breast-ovarian cancer (familial 2), , not specified, Neoplastic Syndromes (Hereditary). This variant is predicted to be tolerable by SIFT

c.1486C>G

The patient is homogeneous for a common missense polymorphism in the MYLK gene which occurs at a frequency of 1.% in the 1000 genomes project. OMIM reports that variants in the MYLK gene are associated with Aortic aneurysm (familial thoracic 7). This variant causes a nonsynonymous conservative amino acid substitution (p.L496V) which may alter protein function. This variant is predicted to be tolerable by SIFT and benign by PolyPhen2.

c.1420G>A

The patient is homogeneous for a common missense polymorphism in the PCSK9 gene which occurs at a frequency of 0.87% in the 1000 genomes project. OMIM reports 2 phenotypes associated with variants in the PCSK9 gene including Hypercholesterolemia (familial) (3), Low density lipoprotein cholesterol level QTL 1. This variant causes a nonsynonymous conservative amino acid substitution (p.V474I) which may alter protein function. This variant is predicted to be tolerable by SIFT and benign by PolyPhen2.

c.13013G>A

The patient is homogeneous for a common missense polymorphism in the APOB gene which occurs at a frequency of 0.63% in the 1000 genomes project. OMIM reports 2 phenotypes associated with variants in the APOB gene including Hypercholesterolemia (due to ligand-defective apo B), Hypobetalipoproteinemia. This variant causes a nonsynonymous conservative amino acid substitution (p.S4338N) which may alter protein function. ClinVar classifies this variant as probable-non-pathogenic and reports an association with not specified. This variant is predicted to be tolerable by SIFT and benign by PolyPhen2.

c.2009G>A

The patient is homogeneous for a common missense polymorphism in the PCSK9 gene which occurs at a frequency of 0.9% in the 1000 genomes project. OMIM reports 2 phenotypes associated with variants in the PCSK9 gene including Hypercholesterolemia (familial) (3), Low density lipoprotein cholesterol level QTL 1. This variant causes a nonsynonymous moderate amino acid substitution (p.G670E) which may alter protein function. ClinVar classifies this variant as non-pathogenic and reports an association with Familial hypercholesterolemia. This variant is predicted to be tolerable by SIFT and benign by PolyPhen2.

RECOMMENDATIONS

Genetic counseling of the family is recommended to discuss the implications of this report.
[Additional recommendations.]

METHODS AND LIMITATIONS

This report is a sample to be used solely for the purpose of evaluating functionality of the Tute Platform and should not be used in any clinical application.

Sequencing and Variant Detection

Whole exome sequencing was performed by solution-based hybridization which enriches for coding exons targeting DNA with specific baits. Sequencing was performed using the Illumina HiSeq 2500 to a minimum average exome depth of 90X. Sequencing reads were aligned to the reference genome (hg19) by BWA-MEM and variants were called using FreeBayes.

Variant Analysis and Report Generation

Reported variants were analyzed using the Tute platform to identify those variants which are reported by medical literature to be pathogenic. All non-synonymous variants of allele frequency <1% reported by the 1000 genomes project and annotated as 'Pathogenic' or 'Probably Pathogenic' by ClinVar are reported in the table 'Primary/Diagnostic Sequence Variant(s) Detected'. Careful correlation of these variants with the clinical indications for the proband should be conducted by an experience medical geneticist to verify that reported findings are consistent with reported clinical phenotypes. Additionally, variants are included based on ACMG guidelines for the reporting of incidental findings in the table 'Secondary/Incidental Sequence Variant(s) Detected'.

Validation of reported findings

Variants of interest should be confirmed by Sanger sequencing. The DNA immediately flanking each of these variants should be amplified genomic DNA followed by sequencing in both directions using fluorescent dideoxy sequencing.

Limitations

Absence of a primary diagnostic finding identified by this test does not exclude the possibility of a genetic basis for the clinical condition for this proband. Certain genetic abnormalities are not detectable by the technologies used to generate this report. Specifically, detection of abnormal variants depends on the presence of these sequence variants in the sequencing data. This exome sequence test is designed to evaluate single nucleotide variants within the human exome; however, this technology is only able to sequence 90-95% of the human reference to the requisite 10-fold coverage needed for reliable detection of heterozygous variants.

Additionally, certain types of genetic abnormalities are difficult to identify in sequencing data and have not been validated for clinical use including insertions, deletions, copy number alterations, long repetitive sequences, triplet repeat expansions, chromosomal rearrangements, polyploidy, repetitive regions including mono-, di- and tri-nucleotide repeats, GX rich regions, intronic variants outside the splice-site and epigenetic effects.

Variants believed to be benign based on medical literature, or with population frequencies greater than or equal to 1%, or resulting in synonymous amino acid changes, or occurring in 5' or 3' untranslated regions are generally not reported.

This report is a sample to be used solely for the purpose of evaluating functionality of the Tute Platform and should not be used in any clinical application.

Sources

REPORT SIGNATURES

Online Mendelian Inheritance in Man, OMIM (TM). Retrieved 0/26/2015 from http://www.ncbi.nlm.nih.gov/omim/
ClinVar. Retrieved 0/26/2015 from http://www.ncbi.nlm.nih.gov/clinvar/

TUTE GENOMICS

NextStep Dx PLUS report for K10031 10143 annotation

ORDERING PROVIDER

,

SPECIMEN

Collection Date:
Received Date:
Report Date:

PATIENT

Name:
Date of Birth:
Patient ID:
Gender:

Clinical Indication:

[Detailed clinical description.]

TEST RESULTS SUMMARY

Primary Result(s): [Findings related to clinical indication.]

Incidental/Secondary Result(s): [Incidental findings.]

PRIMARY/DIAGNOSTIC SEQUENCE VARIANT(S) DETECTED

Gene	Nucleotide Change	Protein Change	Zygosity	Tute Dx
HFE	c.845G>A	p.C282Y	het	Hereditary hemochromatosis|Hemochromatosis, juvenile, digenic
MKKS	c.724G>T	p.A242S	het	McKusick Kaufman syndrome
F5	c.1601A>G	p.Q534R	het	Thrombophilia due to factor V Leiden
TGM1	c.1552G>A	p.V518M	het	Autosomal recessive congenital ichthyosis 1
DPYD	c.85C>T	p.R29C	hom	Dihydropyrimidine dehydrogenase deficiency;not provided
DCLRE1C	c.97G>A	p.G33R	het	Severe combined immunodeficiency disease
PRSS1	c.86A>T	p.N29I	het	Hereditary pancreatitis;Hereditary pancreatitis
FUT2	c.461G>A	p.W154X	het	Norwalk virus infection, resistance to
PRSS1	c.47C>T	p.A16V	het	Hereditary pancreatitis
PRSS1	c.161A>G	p.N54S	het	Hereditary pancreatitis
OCA2	c.913C>T	p.R305W	het	Skin/hair/eye pigmentation, variation in, 1
TIRAP	c.539C>T	p.S180L	het	Malaria, resistance to|BACTEREMIA, RESISTANCE TO
CST3	c.73G>A	p.A25T	het	Age-related macular degeneration 11
HEXB	c.185T>C	p.L62S	hom	Sandhoff disease, infantile type
BBS2	c.209G>A	p.S70N	hom	Bardet-Biedl syndrome 2
KLK1	c.230G>A	p.R77H	het	Kallikrein, decreased urinary activity of
ITGA2B	c.2621T>G	p.I874S	hom	Bak platelet-specific antigen
FCGR3B	c.302A>G	p.N101S	hom	Neutrophil-specific antigens na1/na2
ART4	c.793G>A	p.D265N	hom	DOMBROCK BLOOD GROUP
HPD	c.97A>G	p.T33A	hom	4-Alpha-hydroxyphenylpyruvate hydroxylase deficiency

SECONDARY/INCIDENTAL SEQUENCE VARIANT(S) DETECTED

The American College of Medical Genetics and Genomics (ACMG) recently published a policy statement on clinical sequencing that emphasizes the possibility of recognizing and reporting incidental or secondary findings of medical value when conducting exome and genome sequencing. The ACMG recommends that laboratories performing clinical sequencing seek and report mutations of specified classes or types in the genes listed within the guidelines. This section of the report describes variants which fit the criteria outlined by ACMG for the reporting of incidental or secondary findings.

Gene	Nucleotide Change	Protein Change	Zygosity	Tute Dx
BRCA1	c.2477C>A	p.T826K	het	Breast cancer
MUTYH	c.1276C>T	p.R426C	het	Adenomatous polyposis coli
MUTYH	c.64G>A	p.V22M	het	.
PMS2	c.1531A>G	p.T511A	het	.
BRCA1	c.1067A>G	p.Q356R	hom	.
SDHD	c.371A>G	p.Q124R	hom	.
FBN1	c.1415G>A	p.C472Y	hom	.
DSC2	c.2326A>G	p.I776V	het	.
BRCA2	c.7397T>C	p.V2466A	hom	Breast cancer
MUTYH	c.1014G>C	p.Q338H	het	.
MEN1	c.1636A>G	p.T546A	hom	.
DSG2	c.2318G>A	p.R773K	het	.
APC	c.5465T>A	p.V1822D	het	.
PMS2	c.1621A>G	p.K541E	hom	Colorectal cancer, non-polyposis
COL3A1	c.4059T>G	p.H1353Q	hom	.
BRCA2	c.1114A>C	p.N372H	het	.
MYLK	c.1486C>G	p.L496V	hom	.
PCSK9	c.1420G>A	p.V474I	hom	.
APOB	c.13013G>A	p.S4338N	hom	.
PCSK9	c.2009G>A	p.G670E	hom	.

INTERPRETATION OF PRIMARY FINDINGS

c.845G>A

The patient is heterozygous for a common missense polymorphism in the HFE gene which occurs at a frequency of 0.013% in the 1000 genomes project. OMIM reports 6 phenotypes associated with variants in the HFE gene including Hemochromatosis, [Transferrin serum level QTL2], susceptibility to Alzheimer disease, Microvascular complications of diabetes 7, susceptibility to Porphyria cutanea tarda, susceptibility to Porphyria variegata. This variant causes a nonsynonymous severe amino acid substitution (p.C282Y) which may alter protein function. ClinVar classifies this variant as pathogenic, other and non-pathogenic and reports an association with Hereditary

hemochromatosis, Porphyria cutanea tarda (susceptibility to), Porphyria variegata (susceptibility to), Hemochromatosis (juvenile) (digenic), Alzheimer disease (susceptibility to), Transferrin serum level quantitative trait locus 2, Microvascular complications of diabetes 7, not specified. This variant is predicted to be deleterious by SIFT and deleterious by PolyPhen2.

c.724G>T

The patient is heterozygous for a rare missense mutation in the MKKS gene which occurs at a frequency of 0.0018% in the 1000 genomes project. OMIM reports 2 phenotypes associated with variants in the MKKS gene including Bardet-Biedl syndrome 6, McKusick-Kaufman syndrome. This variant causes a nonsynonymous moderate amino acid substitution (p.A242S) which may alter protein function. ClinVar classifies this variant as pathogenic and reports an association with McKusick Kaufman syndrome. This variant is predicted to be tolerable by SIFT and deleterious by PolyPhen2.

c.1601A>G

The patient is heterozygous for a common missense polymorphism in the F5 gene which occurs at a frequency of 0.99% in the 1000 genomes project. OMIM reports 6 phenotypes associated with variants in the F5 gene including Factor V deficiency, Thrombophilia due to activated protein C resistance, Budd-Chiari syndrome, susceptibility to Pregnancy loss recurrent 1, susceptibility to Stroke (ischemic), susceptibility to Thrombophilia due to factor V Leiden. This variant causes a nonsynonymous conservative amino acid substitution (p.Q534R) which may alter protein function. ClinVar classifies this variant as pathogenic, untested and other and reports an association with Thrombophilia due to factor V Leiden, Ischemic stroke (susceptibility to), Budd-Chiari syndrome (susceptibility to), Recurrent abortion. This variant is predicted to be tolerable by SIFT

c.1552G>A

The patient is heterozygous for a common missense polymorphism in the TGM1 gene which occurs at a frequency of 0.0036% in the 1000 genomes project. OMIM reports that variants in the TGM1 gene are associated with Ichthyosis (congenital) (autosomal recessive 1). This variant causes a nonsynonymous conservative amino acid substitution (p.V518M) which may alter protein function. ClinVar classifies this variant as pathogenic and reports an association with Autosomal recessive congenital ichthyosis 1. This variant is predicted to be tolerable by SIFT and deleterious by PolyPhen2.

c.85C>T

The patient is homogeneous for a common missense polymorphism in the DPYD gene which occurs at a frequency of 0.74% in the 1000 genomes project. OMIM reports 2 phenotypes associated with variants in the DPYD gene including 5-fluorouracil toxicity, Dihydropyrimidine dehydrogenase deficiency. This variant causes a nonsynonymous severe amino acid substitution (p.R29C) which may alter protein function. ClinVar classifies this variant as pathogenic and reports an association with Dihydropyrimidine dehydrogenase deficiency;. This variant is predicted to be tolerable by SIFT and deleterious by PolyPhen2.

c.97G>A

The patient is heterozygous for a common missense polymorphism in the DCLRE1C gene which occurs at a frequency of 0.0062% in the 1000 genomes project. OMIM reports 2 phenotypes associated with variants in the DCLRE1C gene including Omenn syndrome, Severe combined immunodeficiency (Athabascan type). This variant causes a nonsynonymous moderate amino acid substitution (p.G33R) which may alter protein function. ClinVar classifies this variant as probable-pathogenic and reports an association with Severe combined immunodeficiency disease. This variant is predicted to be deleterious by SIFT and deleterious by PolyPhen2.

c.86A>T

The patient is heterozygous for a common missense polymorphism in the PRSS1 gene which doesn't occur in the 1000 genomes project. OMIM reports 2 phenotypes associated with variants in the PRSS1 gene including Pancreatitis (hereditary), Trypsinogen deficiency. This variant causes a nonsynonymous moderate amino acid substitution (p.N29I) which may alter protein function. ClinVar

nonsynonymous moderate amino acid substitution (p.N29I) which may alter protein function. ClinVar classifies this variant as pathogenic and reports an association with Hereditary pancreatitis;Hereditary pancreatitis. This variant is predicted to be deleterious by SIFT and benign by PolyPhen2.

c.461G>A

The patient is heterozygous for a common nonsense polymorphism in the FUT2 gene which occurs at a frequency of 0.32% in the 1000 genomes project. OMIM reports 3 phenotypes associated with variants in the FUT2 gene including [Bombay phenotype], resistance to Norwalk virus infection, Vitamin B12 plasma level QTL1. This variant introduces a premature stop codon which may lead to early termination of protein transcription. ClinVar classifies this variant as non-pathogenic, pathogenic and other and reports an association with SECRETOR/NONSECRETOR POLYMORPHISM, Norwalk virus infection (resistance to), Vitamin b12 plasma level quantitative trait locus 1. This variant is predicted to be tolerable by SIFT

c.47C>T

The patient is heterozygous for a common missense polymorphism in the PRSS1 gene which doesn't occur in the 1000 genomes project. OMIM reports 2 phenotypes associated with variants in the PRSS1 gene including Pancreatitis (hereditary), Trypsinogen deficiency. This variant causes a nonsynonymous moderate amino acid substitution (p.A16V) which may alter protein function. ClinVar classifies this variant as pathogenic and reports an association with Hereditary pancreatitis. This variant is predicted to be tolerable by SIFT and benign by PolyPhen2.

c.161A>G

The patient is heterozygous for a common missense polymorphism in the PRSS1 gene which doesn't occur in the 1000 genomes project. OMIM reports 2 phenotypes associated with variants in the PRSS1 gene including Pancreatitis (hereditary), Trypsinogen deficiency. This variant causes a nonsynonymous conservative amino acid substitution (p.N54S) which may alter protein function. ClinVar classifies this variant as pathogenic and reports an association with Hereditary pancreatitis. This variant is predicted to be tolerable by SIFT and benign by PolyPhen2.

c.913C>T

The patient is heterozygous for a common missense polymorphism in the OCA2 gene which occurs at a frequency of 0.083% in the 1000 genomes project. OMIM reports 4 phenotypes associated with variants in the OCA2 gene including Albinism (brown oculocutaneous), Albinism (oculocutaneous) (type II), [Skin/hair/eye pigmentation 1 (blond)/brown hair], [Skin/hair/eye pigmentation 1 (blue)/nonblue eyes]. This variant causes a nonsynonymous moderate amino acid substitution (p.R305W) which may alter protein function. ClinVar classifies this variant as pathogenic and reports an association with Skin/hair/eye pigmentation (variation in) (1). This variant is predicted to be deleterious by SIFT and possibly deleterious by PolyPhen2.

c.539C>T

The patient is heterozygous for a common missense polymorphism in the TIRAP gene which occurs at a frequency of 0.086% in the 1000 genomes project. OMIM reports 4 phenotypes associated with variants in the TIRAP gene including protection against Bacteremia, protection against Malaria, protection against Pneumococcal disease (invasive), protection against Tuberculosis. This variant causes a nonsynonymous moderate amino acid substitution (p.S180L) which may alter protein function. ClinVar classifies this variant as other and pathogenic and reports an association with Invasive pneumococcal disease (protection against), Malaria (resistance to), Mycobacterium tuberculosis (protection against), BACTEREMIA (RESISTANCE TO). This variant is predicted to be possibly deleterious by PolyPhen2.

c.73G>A

The patient is heterozygous for a common missense polymorphism in the CST3 gene which occurs at a frequency of 0.21% in the 1000 genomes project. OMIM reports 2 phenotypes associated with variants in the CST3 gene including Cerebral amyloid angiopathy, Macular degeneration (age-related) (11). This variant causes a nonsynonymous moderate amino acid substitution (p.A25T) which may

(11). This variant causes a nonsynonymous moderate amino acid substitution (p.[illegible]) which may alter protein function. ClinVar classifies this variant as pathogenic and reports an association with Age-related macular degeneration 11. This variant is predicted to be tolerable by SIFT and benign by PolyPhen2.

c.185T>C

The patient is homogeneous for a common missense polymorphism in the HEXB gene which occurs at a frequency of 0.98% in the 1000 genomes project. OMIM reports that variants in the HEXB gene are associated with Sandhoff disease (infantile) (juvenile) (and adult forms). This variant causes a nonsynonymous moderate amino acid substitution (p.L62S) which may alter protein function. ClinVar classifies this variant as pathogenic and reports an association with Sandhoff disease (infantile type). This variant is predicted to be tolerable by SIFT

c.209G>A

The patient is homogeneous for a common missense polymorphism in the BBS2 gene which occurs at a frequency of 1.% in the 1000 genomes project. OMIM reports that variants in the BBS2 gene are associated with Bardet-Biedl syndrome 2. This variant causes a nonsynonymous conservative amino acid substitution (p.S70N) which may alter protein function. ClinVar classifies this variant as pathogenic and reports an association with Bardet-Biedl syndrome 2. This variant is predicted to be tolerable by SIFT

c.230G>A

The patient is heterozygous for a common missense polymorphism in the KLK1 gene which occurs at a frequency of 0.042% in the 1000 genomes project. OMIM reports that variants in the KLK1 gene are associated with [decreased urinary activity of Kallikrein]. This variant causes a nonsynonymous conservative amino acid substitution (p.R77H) which may alter protein function. ClinVar classifies this variant as pathogenic and reports an association with Kallikrein (decreased urinary activity of). This variant is predicted to be deleterious by SIFT and possibly deleterious by PolyPhen2.

c.2621T>G

The patient is homogeneous for a common missense polymorphism in the ITGA2B gene which occurs at a frequency of 0.4% in the 1000 genomes project. OMIM reports 3 phenotypes associated with variants in the ITGA2B gene including Bleeding disorder (platelet-type) (16) (autosomal dominant), Glanzmann thrombasthenia, Thrombocytopenia (neonatal alloimmune) (BAK antigen related). This variant causes a nonsynonymous moderate amino acid substitution (p.I874S) which may alter protein function. ClinVar classifies this variant as pathogenic and reports an association with Bak platelet-specific antigen. This variant is predicted to be tolerable by SIFT and benign by PolyPhen2.

c.302A>G

The patient is homogeneous for a common missense polymorphism in the FCGR3B gene which occurs at a frequency of 0.53% in the 1000 genomes project. OMIM reports that variants in the FCGR3B gene are associated with Neutropenia (alloimmune neonatal). This variant causes a nonsynonymous conservative amino acid substitution (p.N101S) which may alter protein function. ClinVar classifies this variant as pathogenic and reports an association with Neutrophil-specific antigens na1/na2. This variant is predicted to be tolerable by SIFT

c.793G>A

The patient is homogeneous for a common missense polymorphism in the ART4 gene which occurs at a frequency of 0.29% in the 1000 genomes project. OMIM reports that variants in the ART4 gene are associated with [Blood group (Dombrock)]. This variant causes a nonsynonymous conservative amino acid substitution (p.D265N) which may alter protein function. ClinVar classifies this variant as pathogenic and reports an association with DOMBROCK BLOOD GROUP. This variant is predicted to be tolerable by SIFT and benign by PolyPhen2.

c.97A>G

The patient is homogeneous for a common missense polymorphism in the HPD gene which occurs at a frequency of 0.88% in the 1000 genomes project. OMIM reports 2 phenotypes associated with

at a frequency of 0.88% in the 1000 genomes project. OMIM reports 2 phenotypes associated with variants in the HPD gene including Hawkinsinuria, Tyrosinemia (type III). This variant causes a nonsynonymous moderate amino acid substitution (p.T33A) which may alter protein function. ClinVar classifies this variant as pathogenic and reports an association with 4-Alpha-hydroxyphenylpyruvate hydroxylase deficiency. This variant is predicted to be tolerable by SIFT

INTERPRETATION OF SECONDARY FINDINGS

---

c.2477C>A

The patient is heterozygous for a rare missense mutation in the BRCA1 gene which occurs at a frequency of 0.0002% in the 1000 genomes project. OMIM reports 2 phenotypes associated with variants in the BRCA1 gene including Breast-ovarian cancer (familial) (1), susceptibility to Pancreatic cancer 4. This variant causes a nonsynonymous moderate amino acid substitution (p.T826K) which may alter protein function. ClinVar classifies this variant as non-pathogenic, unknown and other and reports an association with BRCA1 and BRCA2 Hereditary Breast and Ovarian Cancer, Familial cancer of breast, Breast-ovarian cancer (familial 1). ARUP classifies this variant as 1 - Not pathogenic or of no clinical significance. Invitae classifies this variant as Benign. This variant is predicted to be deleterious by SIFT and deleterious by PolyPhen2.

c.1276C>T

The patient is heterozygous for a rare missense mutation in the MUTYH gene which doesn't occur in the 1000 genomes project. OMIM reports 3 phenotypes associated with variants in the MUTYH gene including Adenomas (multiple colorectal), Colorectal adenomatous polyposis (autosomal recessive) (with pilomatricomas), Gastric cancer (somatic). This variant causes a nonsynonymous severe amino acid substitution (p.R426C) which may alter protein function. ClinVar classifies this variant as unknown and other and reports an association with , Neoplastic Syndromes (Hereditary), MYH-associated polyposis. Invitae classifies this variant as Variant of uncertain significance. This variant is predicted to be tolerable by SIFT and deleterious by PolyPhen2.

c.64G>A

The patient is heterozygous for a common missense polymorphism in the MUTYH gene which occurs at a frequency of 0.02% in the 1000 genomes project. OMIM reports 3 phenotypes associated with variants in the MUTYH gene including Adenomas (multiple colorectal), Colorectal adenomatous polyposis (autosomal recessive) (with pilomatricomas), Gastric cancer (somatic). This variant causes a nonsynonymous conservative amino acid substitution (p.V22M) which may alter protein function. ClinVar classifies this variant as non-pathogenic and reports an association with , not specified, MYH-associated polyposis, Neoplastic Syndromes (Hereditary). Invitae classifies this variant as Benign. This variant is predicted to be deleterious by SIFT and possibly deleterious by PolyPhen2.

c.1531A>G

The patient is heterozygous for a common missense polymorphism in the PMS2 gene which occurs at a frequency of 0.011% in the 1000 genomes project. OMIM reports 2 phenotypes associated with variants in the PMS2 gene including Colorectal cancer (hereditary nonpolyposis) (type 4), Mismatch repair cancer syndrome. This variant causes a nonsynonymous moderate amino acid substitution (p.T511A) which may alter protein function. ClinVar classifies this variant as non-pathogenic and untested and reports an association with , Lynch syndrome, not specified, Neoplastic Syndromes (Hereditary). Invitae classifies this variant as Benign. This variant is predicted to be tolerable by SIFT and benign by PolyPhen2.

c.1067A>G

The patient is homogeneous for a common missense polymorphism in the BRCA1 gene which occurs at a frequency of 0.022% in the 1000 genomes project. OMIM reports 2 phenotypes associated with variants in the BRCA1 gene including Breast-ovarian cancer (familial) (1),

susceptibility to Pancreatic cancer 4. This variant causes a nonsynonymous conservative amino acid substitution (p.Q356R) which may alter protein function. ClinVar classifies this variant as non-pathogenic, other and untested and reports an association with , BRCA1 and BRCA2 Hereditary Breast and Ovarian Cancer, Breast-ovarian cancer (familial 1), not specified, Neoplastic Syndromes (Hereditary). ARUP classifies this variant as 1 - Not pathogenic or of no clinical significance. Invitae classifies this variant as Benign. This variant is predicted to be deleterious by SIFT and deleterious by PolyPhen2.

c.371A>G

The patient is homogeneous for a common missense polymorphism in the SDHD gene which occurs at a frequency of 0.95% in the 1000 genomes project. OMIM reports 6 phenotypes associated with variants in the SDHD gene including Carcinoid tumors (intestinal), Cowden syndrome 3, Merkel cell carcinoma (somatic), Paraganglioma and gastric stromal sarcoma, Paragangliomas 1 (with or without deafness), Pheochromocytoma. This variant causes a nonsynonymous conservative amino acid substitution (p.Q124R) which may alter protein function. This variant is predicted to be tolerable by SIFT

c.1415G>A

The patient is homogeneous for a common missense polymorphism in the FBN1 gene which occurs at a frequency of 1.% in the 1000 genomes project. OMIM reports 8 phenotypes associated with variants in the FBN1 gene including Acromicric dysplasia, Aortic aneurysm (ascending) (and dissection), Ectopia lentis (familial), Geleophysic dysplasia 2, MASS syndrome, Marfan syndrome, Stiff skin syndrome, Weill-Marchesani syndrome 2 (dominant). This variant causes a nonsynonymous severe amino acid substitution (p.C472Y) which may alter protein function. This variant is predicted to be tolerable by SIFT and benign by PolyPhen2.

c.2326A>G

The patient is heterozygous for a common missense polymorphism in the DSC2 gene which occurs at a frequency of 0.2% in the 1000 genomes project. OMIM reports 2 phenotypes associated with variants in the DSC2 gene including Arrhythmogenic right ventricular dysplasia 11 with mild palmoplantar keratoderma and woolly hair, Arrhythmogenic right ventricular dysplasia 11. This variant causes a nonsynonymous conservative amino acid substitution (p.I776V) which may alter protein function. ClinVar classifies this variant as non-pathogenic and reports an association with not specified. This variant is predicted to be tolerable by SIFT and benign by PolyPhen2.

c.7397T>C

The patient is homogeneous for a common missense polymorphism in the BRCA2 gene which occurs at a frequency of 0.98% in the 1000 genomes project. OMIM reports 9 phenotypes associated with variants in the BRCA2 gene including Fanconi anemia (complementation group D1), Pancreatic cancer, Prostate cancer, Wilms tumor, susceptibility to Breast cancer (male), Breast-ovarian cancer (familial) (2), Glioblastoma 3, Medulloblastoma, Pre-B-cell acute lymphoblastic leukemia. This variant causes a nonsynonymous moderate amino acid substitution (p.V2466A) which may alter protein function. ClinVar classifies this variant as untested, unknown and non-pathogenic and reports an association with Familial cancer of breast, Breast-ovarian cancer (familial 2), not specified. This variant is predicted to be tolerable by SIFT

c.1014G>C

The patient is heterozygous for a common missense polymorphism in the MUTYH gene which occurs at a frequency of 0.31% in the 1000 genomes project. OMIM reports 3 phenotypes associated with variants in the MUTYH gene including Adenomas (multiple colorectal), Colorectal adenomatous polyposis (autosomal recessive) (with pilomatricomas), Gastric cancer (somatic). This variant causes a nonsynonymous conservative amino acid substitution (p.Q338H) which may alter protein function. ClinVar classifies this variant as non-pathogenic and reports an association with , not specified, Neoplastic Syndromes (Hereditary). This variant is predicted to be tolerable by SIFT and possibly deleterious by PolyPhen2.

c.1636A>G

c.[illegible]

The patient is homogeneous for a common missense polymorphism in the MEN1 gene which occurs at a frequency of 0.83% in the 1000 genomes project. OMIM reports 6 phenotypes associated with variants in the MEN1 gene including Adrenal adenoma (somatic), Angiofibroma (somatic), Carcinoid tumor of lung, Lipoma (somatic), Multiple endocrine neoplasia 1, Parathyroid adenoma (somatic). This variant causes a nonsynonymous moderate amino acid substitution (p.T546A) which may alter protein function. ClinVar classifies this variant as non-pathogenic and reports an association with , not specified. This variant is predicted to be tolerable by SIFT and benign by PolyPhen2.

c.2318G>A

The patient is heterozygous for a common missense polymorphism in the DSG2 gene which occurs at a frequency of 0.24% in the 1000 genomes project. OMIM reports 2 phenotypes associated with variants in the DSG2 gene including Arrhythmogenic right ventricular dysplasia 10, Cardiomyopathy (dilated) (1BB). This variant causes a nonsynonymous conservative amino acid substitution (p.R773K) which may alter protein function. ClinVar classifies this variant as non-pathogenic and reports an association with not specified. This variant is predicted to be tolerable by SIFT and benign by PolyPhen2.

c.5465T>A

The patient is heterozygous for a common missense polymorphism in the APC gene which occurs at a frequency of 0.87% in the 1000 genomes project. OMIM reports 8 phenotypes associated with variants in the APC gene including Adenoma (periampullary) (somatic), Adenomatous polyposis coli, Brain tumor-polyposis syndrome 2, Colorectal cancer (somatic), Desmoid disease (hereditary), Gardner syndrome, Gastric cancer (somatic), Hepatoblastoma (somatic). This variant causes a nonsynonymous severe amino acid substitution (p.V1822D) which may alter protein function. ClinVar classifies this variant as non-pathogenic and other and reports an association with Adenomatous polyposis coli, , not specified, Familial colorectal cancer, Neoplastic Syndromes (Hereditary). This variant is predicted to be benign by PolyPhen2.

c.1621A>G

The patient is homogeneous for a common missense polymorphism in the PMS2 gene which occurs at a frequency of 0.88% in the 1000 genomes project. OMIM reports 2 phenotypes associated with variants in the PMS2 gene including Colorectal cancer (hereditary nonpolyposis) (type 4), Mismatch repair cancer syndrome. This variant causes a nonsynonymous moderate amino acid substitution (p.K541E) which may alter protein function. ClinVar classifies this variant as non-pathogenic and reports an association with Lynch syndrome. This variant is predicted to be tolerable by SIFT and benign by PolyPhen2.

c.4059T>G

The patient is homogeneous for a common missense polymorphism in the COL3A1 gene which occurs at a frequency of 1.% in the 1000 genomes project. OMIM reports 2 phenotypes associated with variants in the COL3A1 gene including Ehlers-Danlos syndrome (type III), Ehlers-Danlos syndrome (type IV). This variant causes a nonsynonymous conservative amino acid substitution (p.H1353Q) which may alter protein function. This variant is predicted to be tolerable by SIFT and benign by PolyPhen2.

c.1114A>C

The patient is heterozygous for a common missense polymorphism in the BRCA2 gene which occurs at a frequency of 0.25% in the 1000 genomes project. OMIM reports 9 phenotypes associated with variants in the BRCA2 gene including Fanconi anemia (complementation group D1), Pancreatic cancer, Prostate cancer, Wilms tumor, susceptibility to Breast cancer (male), Breast-ovarian cancer (familial) (2), Glioblastoma 3, Medulloblastoma, Pre-B-cell acute lymphoblastic leukemia. This variant causes a nonsynonymous moderate amino acid substitution (p.N372H) which may alter protein function. ClinVar classifies this variant as other, non-pathogenic and untested and reports an association with Breast-ovarian cancer (familial 2), , not specified, Neoplastic Syndromes (Hereditary). This variant is predicted to be tolerable by SIFT

(Hereditary). This variant is predicted to be tolerable by SIFT

c.1486C>G

The patient is homogeneous for a common missense polymorphism in the MYLK gene which occurs at a frequency of 1.% in the 1000 genomes project. OMIM reports that variants in the MYLK gene are associated with Aortic aneurysm (familial thoracic 7). This variant causes a nonsynonymous conservative amino acid substitution (p.L496V) which may alter protein function. This variant is predicted to be tolerable by SIFT and benign by PolyPhen2.

c.1420G>A

The patient is homogeneous for a common missense polymorphism in the PCSK9 gene which occurs at a frequency of 0.87% in the 1000 genomes project. OMIM reports 2 phenotypes associated with variants in the PCSK9 gene including Hypercholesterolemia (familial) (3), Low density lipoprotein cholesterol level QTL 1. This variant causes a nonsynonymous conservative amino acid substitution (p.V474I) which may alter protein function. This variant is predicted to be tolerable by SIFT and benign by PolyPhen2.

c.13013G>A

The patient is homogeneous for a common missense polymorphism in the APOB gene which occurs at a frequency of 0.63% in the 1000 genomes project. OMIM reports 2 phenotypes associated with variants in the APOB gene including Hypercholesterolemia (due to ligand-defective apo B), Hypobetalipoproteinemia. This variant causes a nonsynonymous conservative amino acid substitution (p.S4338N) which may alter protein function. ClinVar classifies this variant as probable-non-pathogenic and reports an association with not specified. This variant is predicted to be tolerable by SIFT and benign by PolyPhen2.

c.2009G>A

The patient is homogeneous for a common missense polymorphism in the PCSK9 gene which occurs at a frequency of 0.9% in the 1000 genomes project. OMIM reports 2 phenotypes associated with variants in the PCSK9 gene including Hypercholesterolemia (familial) (3), Low density lipoprotein cholesterol level QTL 1. This variant causes a nonsynonymous moderate amino acid substitution (p.G670E) which may alter protein function. ClinVar classifies this variant as non-pathogenic and reports an association with Familial hypercholesterolemia. This variant is predicted to be tolerable by SIFT and benign by PolyPhen2.

RECOMMENDATIONS

Genetic counseling of the family is recommended to discuss the implications of this report. [Additional recommendations.]

METHODS AND LIMITATIONS

This report is a sample to be used solely for the purpose of evaluating functionality of the Tute Platform and should not be used in any clinical application.

Sequencing and Variant Detection

Whole exome sequencing was performed by solution-based hybridization which enriches for coding exons targeting DNA with specific baits. Sequencing was performed using the Illumina HiSeq 2500 to a minimum average exome depth of 90X. Sequencing reads were aligned to the reference genome (hg19) by BWA-MEM and variants were called using FreeBayes.

Variant Analysis and Report Generation

Reported variants were analyzed using the Tute platform to identify those variants which are reported by medical literature to be pathogenic. All non-synonymous variants of allele frequency <1% reported by the 1000 genomes project and annotated as 'Pathogenic' or 'Probably Pathogenic' by ClinVar are reported in the table 'Primary/Diagnostic Sequence Variant(s) Detected'. Careful correlation of these variants with the clinical indications for the proband should be conducted by an experience medical geneticist to verify that reported findings are consistent with reported clinical phenotypes. Additionally, variants are included based on ACMG guidelines for the reporting of incidental findings in the table 'Secondary/Incidental Sequence Variant(s) Detected'.

Validation of reported findings

Variants of interest should be confirmed by Sanger sequencing. The DNA immediately flanking each of these variants should be amplified genomic DNA followed by sequencing in both directions using fluorescent dideoxy sequencing.

Limitations

Absence of a primary diagnostic finding identified by this test does not exclude the possibility of a genetic basis for the clinical condition for this proband. Certain genetic abnormalities are not detectable by the technologies used to generate this report. Specifically, detection of abnormal variants depends on the presence of these sequence variants in the sequencing data. This exome sequence test is designed to evaluate single nucleotide variants within the human exome; however, this technology is only able to sequence 90-95% of the human reference to the requisite 10-fold coverage needed for reliable detection of heterozygous variants.

Additionally, certain types of genetic abnormalities are difficult to identify in sequencing data and have not been validated for clinical use including insertions, deletions, copy number alterations, long repetitive sequences, triplet repeat expansions, chromosomal rearrangements, polyploidy, repetitive regions including mono-, di- and tri-nucleotide repeats, GX rich regions, intronic variants outside the splice-site and epigenetic effects.

Variants believed to be benign based on medical literature, or with population frequencies greater than or equal to 1%, or resulting in synonymous amino acid changes, or occurring in 5' or 3' untranslated regions are generally not reported.

This report is a sample to be used solely for the purpose of evaluating functionality of the Tute Platform and should not be used in any clinical application.

Sources

REPORT SIGNATURES

Online Mendelian Inheritance in Man, OMIM (TM). Retrieved 0/26/2015 from http://www.ncbi.nlm.nih.gov/omim/
ClinVar. Retrieved 0/26/2015 from http://www.ncbi.nlm.nih.gov/clinvar/

TUTE GENOMICS

NextStep Dx PLUS report for K10031 10144 annotation

ORDERING PROVIDER

,

SPECIMEN

Collection Date:
Received Date:
Report Date:

PATIENT

Name:
Date of Birth:
Patient ID:
Gender:

Clinical Indication:

[Detailed clinical description.]

TEST RESULTS SUMMARY

Primary Result(s): [Findings related to clinical indication.]

Incidental/Secondary Result(s): [Incidental findings.]

PRIMARY/DIAGNOSTIC SEQUENCE VARIANT(S) DETECTED

Gene	Nucleotide Change	Protein Change	Zygosity	Tute Dx
LYST	c.2413delG	p.E805fs	het	Chédiak-Higashi syndrome
VPS13B	c.9406-1G>T	.	het	Cohen syndrome
F5	c.1601A>G	p.Q534R	hom	Thrombophilia due to factor V Leiden
TGM1	c.1552G>A	p.V518M	het	Autosomal recessive congenital ichthyosis 1
DPYD	c.85C>T	p.R29C	hom	Dihydropyrimidine dehydrogenase deficiency;not provided
DCLRE1C	c.97G>A	p.G33R	het	Severe combined immunodeficiency disease
PRSS1	c.86A>T	p.N29I	het	Hereditary pancreatitis;Hereditary pancreatitis
ELAC2	c.1621G>A	p.A541T	het	Prostate cancer, hereditary, 2
OCA2	c.913C>T	p.R305W	het	Skin/hair/eye pigmentation, variation in, 1
PRF1	c.755A>G	p.N252S	het	Hemophagocytic lymphohistiocytosis, familial, 2|Malignant lymphoma, non-Hodgkin
KRT85	c.233G>A	p.R78H	het	Ectodermal dysplasia, 'pure' hair-nail type
CYP2B6	c.785A>G	p.K262R	het	Efavirenz, poor metabolism of
ITGB3	c.176T>C	p.L59P	het	Thrombocytopenia, neonatal alloimmune|Posttransfusion purpura
CST3	c.73G>A	p.A25T	hom	Age-related macular degeneration 11
HEXB	c.185T>C	p.L62S	hom	Sandhoff disease, infantile type
BANK1	c.182G>A	p.R61H	het	Systemic lupus erythmatosus, association with
BBS2	c.209G>A	p.S70N	hom	Bardet-Biedl syndrome 2
KLK1	c.230G>A	p.R77H	het	Kallikrein, decreased urinary activity of
NAT1	c.445G>A	p.V149I	het	NAT1*17 ALLELE
ITGA2B	c.2621T>G	p.I874S	hom	Bak platelet-specific antigen

SECONDARY/INCIDENTAL SEQUENCE VARIANT(S) DETECTED

The American College of Medical Genetics and Genomics (ACMG) recently published a policy statement on clinical sequencing that emphasizes the possibility of recognizing and reporting incidental or secondary findings of medical value when conducting exome and genome sequencing. The ACMG recommends that laboratories performing clinical sequencing seek and report mutations of specified classes or types in the genes listed within the guidelines. This section of the report describes variants which fit the criteria outlined by ACMG for the reporting of incidental or secondary findings.

Gene	Nucleotide Change	Protein Change	Zygosity	Tute Dx
RYR1	c.7336G>T	p.G2446C	het	.
BRCA1	c.2477C>A	p.T826K	het	Breast cancer
MUTYH	c.64G>A	p.V22M	het	.
PMS2	c.1531A>G	p.T511A	het	.
BRCA1	c.1067A>G	p.Q356R	het	.
SDHD	c.371A>G	p.Q124R	hom	.
FBN1	c.1415G>A	p.C472Y	hom	.
SCN5A	c.1673A>G	p.H558R	het	Progressive familial heart block type 1A
DSC2	c.2326A>G	p.I776V	het	.
APOB	c.1853C>T	p.A618V	het	.
TP53	c.215C>G	p.P72R	het	.
BRCA2	c.7397T>C	p.V2466A	hom	Breast cancer
MUTYH	c.1014G>C	p.Q338H	het	.
MEN1	c.1636A>G	p.T546A	hom	.
DSG2	c.2318G>A	p.R773K	het	.
APC	c.5465T>A	p.V1822D	het	.
PMS2	c.1621A>G	p.K541E	hom	Colorectal cancer, non-polyposis
COL3A1	c.4059T>G	p.H1353Q	hom	.
BRCA2	c.1114A>C	p.N372H	het	.
MYLK	c.1486C>G	p.L496V	hom	.

INTERPRETATION OF PRIMARY FINDINGS

c.2413delG

The patient is heterozygous for a rare mutation in the LYST gene which doesn't occur in the 1000 genomes project. OMIM reports that variants in the LYST gene are associated with Chediak-Higashi syndrome. This variant causes a frameshift which may significantly alter protein expression or function. [illegible]

function. ClinVar classifies this variant as pathogenic and reports an association with Chediak-Higashi syndrome.

c.9406-1G>T

The patient is heterozygous for a common polymorphism in the VPS13B gene which doesn't occur in the 1000 genomes project. OMIM reports that variants in the VPS13B gene are associated with Cohen syndrome. ClinVar classifies this variant as probable-pathogenic and reports an association with Cohen syndrome.

c.1601A>G

The patient is homogeneous for a common missense polymorphism in the F5 gene which occurs at a frequency of 0.99% in the 1000 genomes project. OMIM reports 6 phenotypes associated with variants in the F5 gene including Factor V deficiency, Thrombophilia due to activated protein C resistance, Budd-Chiari syndrome, susceptibility to Pregnancy loss recurrent 1, susceptibility to Stroke (ischemic), susceptibility to Thrombophilia due to factor V Leiden. This variant causes a nonsynonymous conservative amino acid substitution (p.Q534R) which may alter protein function. ClinVar classifies this variant as pathogenic, untested and other and reports an association with Thrombophilia due to factor V Leiden, Ischemic stroke (susceptibility to), Budd-Chiari syndrome (susceptibility to), Recurrent abortion. This variant is predicted to be tolerable by SIFT

c.1552G>A

The patient is heterozygous for a common missense polymorphism in the TGM1 gene which occurs at a frequency of 0.0036% in the 1000 genomes project. OMIM reports that variants in the TGM1 gene are associated with Ichthyosis (congenital) (autosomal recessive 1). This variant causes a nonsynonymous conservative amino acid substitution (p.V518M) which may alter protein function. ClinVar classifies this variant as pathogenic and reports an association with Autosomal recessive congenital ichthyosis 1. This variant is predicted to be tolerable by SIFT and deleterious by PolyPhen2.

c.85C>T

The patient is homogeneous for a common missense polymorphism in the DPYD gene which occurs at a frequency of 0.74% in the 1000 genomes project. OMIM reports 2 phenotypes associated with variants in the DPYD gene including 5-fluorouracil toxicity, Dihydropyrimidine dehydrogenase deficiency. This variant causes a nonsynonymous severe amino acid substitution (p.R29C) which may alter protein function. ClinVar classifies this variant as pathogenic and reports an association with Dihydropyrimidine dehydrogenase deficiency;. This variant is predicted to be tolerable by SIFT and deleterious by PolyPhen2.

c.97G>A

The patient is heterozygous for a common missense polymorphism in the DCLRE1C gene which occurs at a frequency of 0.0062% in the 1000 genomes project. OMIM reports 2 phenotypes associated with variants in the DCLRE1C gene including Omenn syndrome, Severe combined immunodeficiency (Athabascan type). This variant causes a nonsynonymous moderate amino acid substitution (p.G33R) which may alter protein function. ClinVar classifies this variant as probable-pathogenic and reports an association with Severe combined immunodeficiency disease. This variant is predicted to be deleterious by SIFT and deleterious by PolyPhen2.

c.86A>T

The patient is heterozygous for a common missense polymorphism in the PRSS1 gene which doesn't occur in the 1000 genomes project. OMIM reports 2 phenotypes associated with variants in the PRSS1 gene including Pancreatitis (hereditary), Trypsinogen deficiency. This variant causes a nonsynonymous moderate amino acid substitution (p.N29I) which may alter protein function. ClinVar classifies this variant as pathogenic and reports an association with Hereditary pancreatitis;Hereditary pancreatitis. This variant is predicted to be deleterious by SIFT and benign by PolyPhen2.

c.1621G>A

[illegible]

The patient is heterozygous for a common missense polymorphism in the ELAC2 gene which occurs at a frequency of 0.023% in the 1000 genomes project. OMIM reports 2 phenotypes associated with variants in the ELAC2 gene including Combined oxidative phosphorylation deficiency 17, susceptibility to Prostate cancer (hereditary) (2). This variant causes a nonsynonymous moderate amino acid substitution (p.A541T) which may alter protein function. ClinVar classifies this variant as pathogenic and reports an association with Prostate cancer (hereditary) (2). This variant is predicted to be tolerable by SIFT and possibly deleterious by PolyPhen2.

c.913C>T

The patient is heterozygous for a common missense polymorphism in the OCA2 gene which occurs at a frequency of 0.083% in the 1000 genomes project. OMIM reports 4 phenotypes associated with variants in the OCA2 gene including Albinism (brown oculocutaneous), Albinism (oculocutaneous) (type II), [Skin/hair/eye pigmentation 1 (blond)/brown hair], [Skin/hair/eye pigmentation 1 (blue)/nonblue eyes]. This variant causes a nonsynonymous moderate amino acid substitution (p.R305W) which may alter protein function. ClinVar classifies this variant as pathogenic and reports an association with Skin/hair/eye pigmentation (variation in) (1). This variant is predicted to be deleterious by SIFT and possibly deleterious by PolyPhen2.

c.755A>G

The patient is heterozygous for a common missense polymorphism in the PRF1 gene which occurs at a frequency of 0.0076% in the 1000 genomes project. OMIM reports 2 phenotypes associated with variants in the PRF1 gene including Hemophagocytic lymphohistiocytosis (familial) (2), Lymphoma (non-Hodgkin). This variant causes a nonsynonymous conservative amino acid substitution (p.N252S) which may alter protein function. ClinVar classifies this variant as pathogenic and reports an association with Hemophagocytic lymphohistiocytosis (familial) (2), Malignant lymphoma (non-Hodgkin). This variant is predicted to be tolerable by SIFT and benign by PolyPhen2.

c.233G>A

The patient is heterozygous for a common missense polymorphism in the KRT85 gene which occurs at a frequency of 0.033% in the 1000 genomes project. OMIM reports that variants in the KRT85 gene are associated with Ectodermal dysplasia 4 (hair)/nail type. This variant causes a nonsynonymous conservative amino acid substitution (p.R78H) which may alter protein function. ClinVar classifies this variant as pathogenic and untested and reports an association with Ectodermal dysplasia ( )'pure' hair-nail type. This variant is predicted to be tolerable by SIFT and benign by PolyPhen2.

c.785A>G

The patient is heterozygous for a common missense polymorphism in the CYP2B6 gene which doesn't occur in the 1000 genomes project. OMIM reports 2 phenotypes associated with variants in the CYP2B6 gene including poor metabolism of Efavirenz, susceptibility to Efavirenz central nervous system toxicity. This variant causes a nonsynonymous conservative amino acid substitution (p.K262R) which may alter protein function. ClinVar classifies this variant as pathogenic and reports an association with Efavirenz (poor metabolism of). This variant is predicted to be tolerable by SIFT and benign by PolyPhen2.

c.176T>C

The patient is heterozygous for a common missense polymorphism in the ITGB3 gene which occurs at a frequency of 0.089% in the 1000 genomes project. OMIM reports 5 phenotypes associated with variants in the ITGB3 gene including Bleeding disorder (platelet-type) (16) (autosomal dominant), Glanzmann thrombasthenia, Purpura (posttransfusion), Thrombocytopenia (neonatal alloimmune), susceptibility to Myocardial infarction. This variant causes a nonsynonymous moderate amino acid substitution (p.L59P) which may alter protein function. ClinVar classifies this variant as non-pathogenic, pathogenic and other and reports an association with PL(A1)/(A2) ALLOANTIGEN POLYMORPHISM, Thrombocytopenia (neonatal alloimmune), Posttransfusion purpura, Myocardial infarction, Fracture (hip) (susceptibility to). This variant is predicted to be

tolerable by SIFT and benign by PolyPhen2.

c.73G>A
The patient is homogeneous for a common missense polymorphism in the CST3 gene which occurs at a frequency of 0.21% in the 1000 genomes project. OMIM reports 2 phenotypes associated with variants in the CST3 gene including Cerebral amyloid angiopathy, Macular degeneration (age-related) (11). This variant causes a nonsynonymous moderate amino acid substitution (p.A25T) which may alter protein function. ClinVar classifies this variant as pathogenic and reports an association with Age-related macular degeneration 11. This variant is predicted to be tolerable by SIFT and benign by PolyPhen2.

c.185T>C
The patient is homogeneous for a common missense polymorphism in the HEXB gene which occurs at a frequency of 0.98% in the 1000 genomes project. OMIM reports that variants in the HEXB gene are associated with Sandhoff disease (infantile) (juvenile) (and adult forms). This variant causes a nonsynonymous moderate amino acid substitution (p.L62S) which may alter protein function. ClinVar classifies this variant as pathogenic and reports an association with Sandhoff disease (infantile type). This variant is predicted to be tolerable by SIFT

c.182G>A
The patient is heterozygous for a common missense polymorphism in the BANK1 gene which occurs at a frequency of 0.22% in the 1000 genomes project. OMIM reports that variants in the BANK1 gene are associated with association with Systemic lupus erythematosus. This variant causes a nonsynonymous conservative amino acid substitution (p.R61H) which may alter protein function. ClinVar classifies this variant as pathogenic and reports an association with Systemic lupus erythmatosus (association with). This variant is predicted to be tolerable by SIFT and benign by PolyPhen2.

c.209G>A
The patient is homogeneous for a common missense polymorphism in the BBS2 gene which occurs at a frequency of 1.% in the 1000 genomes project. OMIM reports that variants in the BBS2 gene are associated with Bardet-Biedl syndrome 2. This variant causes a nonsynonymous conservative amino acid substitution (p.S70N) which may alter protein function. ClinVar classifies this variant as pathogenic and reports an association with Bardet-Biedl syndrome 2. This variant is predicted to be tolerable by SIFT

c.230G>A
The patient is heterozygous for a common missense polymorphism in the KLK1 gene which occurs at a frequency of 0.042% in the 1000 genomes project. OMIM reports that variants in the KLK1 gene are associated with [decreased urinary activity of Kallikrein]. This variant causes a nonsynonymous conservative amino acid substitution (p.R77H) which may alter protein function. ClinVar classifies this variant as pathogenic and reports an association with Kallikrein (decreased urinary activity of). This variant is predicted to be deleterious by SIFT and possibly deleterious by PolyPhen2.

c.445G>A
The patient is heterozygous for a common missense polymorphism in the NAT1 gene which occurs at a frequency of 0.017% in the 1000 genomes project. OMIM reports that variants in the NAT1 gene are associated with Orthostatic intolerance. This variant causes a nonsynonymous conservative amino acid substitution (p.V149I) which may alter protein function. ClinVar classifies this variant as pathogenic and reports an association with NAT1*17 ALLELE. This variant is predicted to be tolerable by SIFT and benign by PolyPhen2.

c.2621T>G
The patient is homogeneous for a common missense polymorphism in the ITGA2B gene which occurs at a frequency of 0.4% in the 1000 genomes project. OMIM reports 3 phenotypes associated with variants in the ITGA2B gene including Bleeding disorder (platelet-type) (16) (autosomal dominant), Glanzmann thrombasthenia, Thrombocytopenia (neonatal alloimmune) (BAK

[illegible] antigen related). This variant causes a nonsynonymous moderate amino acid substitution (p.I874S) which may alter protein function. ClinVar classifies this variant as pathogenic and reports an association with Bak platelet-specific antigen. This variant is predicted to be tolerable by SIFT and benign by PolyPhen2.

INTERPRETATION OF SECONDARY FINDINGS

---

c.7336G>T

The patient is heterozygous for a rare mutation in the RYR1 gene which doesn't occur in the 1000 genomes project. OMIM reports 5 phenotypes associated with variants in the RYR1 gene including Central core disease, King-Denborough syndrome, Minicore myopathy with external ophthalmoplegia, Neuromuscular disease (congenital) (with uniform type 1 fiber), Malignant hyperthermia susceptibility 1. This variant causes a nonsynonymous severe amino acid substitution (p.G2446C) which may alter protein function. This variant is predicted to be deleterious by SIFT and benign by PolyPhen2.

c.2477C>A

The patient is heterozygous for a rare missense mutation in the BRCA1 gene which occurs at a frequency of 0.0002% in the 1000 genomes project. OMIM reports 2 phenotypes associated with variants in the BRCA1 gene including Breast-ovarian cancer (familial) (1), susceptibility to Pancreatic cancer 4. This variant causes a nonsynonymous moderate amino acid substitution (p.T826K) which may alter protein function. ClinVar classifies this variant as non-pathogenic, unknown and other and reports an association with BRCA1 and BRCA2 Hereditary Breast and Ovarian Cancer, Familial cancer of breast, Breast-ovarian cancer (familial 1). ARUP classifies this variant as 1 - Not pathogenic or of no clinical significance. Invitae classifies this variant as Benign. This variant is predicted to be deleterious by SIFT and deleterious by PolyPhen2.

c.64G>A

The patient is heterozygous for a common missense polymorphism in the MUTYH gene which occurs at a frequency of 0.02% in the 1000 genomes project. OMIM reports 3 phenotypes associated with variants in the MUTYH gene including Adenomas (multiple colorectal), Colorectal adenomatous polyposis (autosomal recessive) (with pilomatricomas), Gastric cancer (somatic). This variant causes a nonsynonymous conservative amino acid substitution (p.V22M) which may alter protein function. ClinVar classifies this variant as non-pathogenic and reports an association with , not specified, MYH-associated polyposis, Neoplastic Syndromes (Hereditary). Invitae classifies this variant as Benign. This variant is predicted to be deleterious by SIFT and possibly deleterious by PolyPhen2.

c.1531A>G

The patient is heterozygous for a common missense polymorphism in the PMS2 gene which occurs at a frequency of 0.011% in the 1000 genomes project. OMIM reports 2 phenotypes associated with variants in the PMS2 gene including Colorectal cancer (hereditary nonpolyposis) (type 4), Mismatch repair cancer syndrome. This variant causes a nonsynonymous moderate amino acid substitution (p.T511A) which may alter protein function. ClinVar classifies this variant as non-pathogenic and untested and reports an association with , Lynch syndrome, not specified, Neoplastic Syndromes (Hereditary). Invitae classifies this variant as Benign. This variant is predicted to be tolerable by SIFT and benign by PolyPhen2.

c.1067A>G

The patient is heterozygous for a common missense polymorphism in the BRCA1 gene which occurs at a frequency of 0.022% in the 1000 genomes project. OMIM reports 2 phenotypes associated with variants in the BRCA1 gene including Breast-ovarian cancer (familial) (1), susceptibility to Pancreatic cancer 4. This variant causes a nonsynonymous conservative amino [illegible]

acid substitution (p.Q356R) which may alter protein function. ClinVar classifies this variant as non-pathogenic, other and untested and reports an association with , BRCA1 and BRCA2 Hereditary Breast and Ovarian Cancer, Breast-ovarian cancer (familial 1), not specified, Neoplastic Syndromes (Hereditary). ARUP classifies this variant as 1 - Not pathogenic or of no clinical significance. Invitae classifies this variant as Benign. This variant is predicted to be deleterious by SIFT and deleterious by PolyPhen2.

c.371A>G

The patient is homogeneous for a common missense polymorphism in the SDHD gene which occurs at a frequency of 0.95% in the 1000 genomes project. OMIM reports 6 phenotypes associated with variants in the SDHD gene including Carcinoid tumors (intestinal), Cowden syndrome 3, Merkel cell carcinoma (somatic), Paraganglioma and gastric stromal sarcoma, Paragangliomas 1 (with or without deafness), Pheochromocytoma. This variant causes a nonsynonymous conservative amino acid substitution (p.Q124R) which may alter protein function. This variant is predicted to be tolerable by SIFT

c.1415G>A

The patient is homogeneous for a common missense polymorphism in the FBN1 gene which occurs at a frequency of 1.% in the 1000 genomes project. OMIM reports 8 phenotypes associated with variants in the FBN1 gene including Acromicric dysplasia, Aortic aneurysm (ascending) (and dissection), Ectopia lentis (familial), Geleophysic dysplasia 2, MASS syndrome, Marfan syndrome, Stiff skin syndrome, Weill-Marchesani syndrome 2 (dominant). This variant causes a nonsynonymous severe amino acid substitution (p.C472Y) which may alter protein function. This variant is predicted to be tolerable by SIFT and benign by PolyPhen2.

c.1673A>G

The patient is heterozygous for a common missense polymorphism in the SCN5A gene which occurs at a frequency of 0.23% in the 1000 genomes project. OMIM reports 9 phenotypes associated with variants in the SCN5A gene including Atrial fibrillation (familial) (10), Brugada syndrome 1, Cardiomyopathy (dilated) (1E), Heart block (nonprogressive), Heart block (progressive) (type IA), Long QT syndrome-3, Sick sinus syndrome 1, Ventricular fibrillation (familial) (1), susceptibility to Sudden infant death syndrome. This variant causes a nonsynonymous conservative amino acid substitution (p.H558R) which may alter protein function. ClinVar classifies this variant as pathogenic and non-pathogenic and reports an association with Progressive familial heart block type 1A, not specified. This variant is predicted to be tolerable by SIFT and benign by PolyPhen2.

c.2326A>G

The patient is heterozygous for a common missense polymorphism in the DSC2 gene which occurs at a frequency of 0.2% in the 1000 genomes project. OMIM reports 2 phenotypes associated with variants in the DSC2 gene including Arrhythmogenic right ventricular dysplasia 11 with mild palmoplantar keratoderma and woolly hair, Arrhythmogenic right ventricular dysplasia 11. This variant causes a nonsynonymous conservative amino acid substitution (p.I776V) which may alter protein function. ClinVar classifies this variant as non-pathogenic and reports an association with not specified. This variant is predicted to be tolerable by SIFT and benign by PolyPhen2.

c.1853C>T

The patient is heterozygous for a common missense polymorphism in the APOB gene which occurs at a frequency of 0.48% in the 1000 genomes project. OMIM reports 2 phenotypes associated with variants in the APOB gene including Hypercholesterolemia (due to ligand-defective apo B), Hypobetalipoproteinemia. This variant causes a nonsynonymous moderate amino acid substitution (p.A618V) which may alter protein function. ClinVar classifies this variant as probable-non-pathogenic and reports an association with not specified. This variant is predicted to be tolerable by SIFT and deleterious by PolyPhen2.

c.215C>G

The patient is heterozygous for a common missense polymorphism in the TP53 gene which occurs at a frequency of 0.54% in the 1000 genomes project. OMIM reports 11 phenotypes associated with

[illegible]
variants in the TP53 gene including Adrenal cortical carcinoma, Breast cancer, Choroid plexus papilloma, Colorectal cancer, Hepatocellular carcinoma, Li-Fraumeni syndrome, Nasopharyngeal carcinoma, Osteosarcoma, Pancreatic cancer, Basal cell carcinoma 7, Glioma susceptibility 1. This variant causes a nonsynonymous moderate amino acid substitution (p.P72R) which may alter protein function. ClinVar classifies this variant as non-pathogenic and reports an association with CODON 72 POLYMORPHISM ( )(rs1042522), , not specified, Neoplastic Syndromes (Hereditary). This variant is predicted to be tolerable by SIFT and possibly deleterious by PolyPhen2.

c.7397T>C

The patient is homogeneous for a common missense polymorphism in the BRCA2 gene which occurs at a frequency of 0.98% in the 1000 genomes project. OMIM reports 9 phenotypes associated with variants in the BRCA2 gene including Fanconi anemia (complementation group D1), Pancreatic cancer, Prostate cancer, Wilms tumor, susceptibility to Breast cancer (male), Breast-ovarian cancer (familial) (2), Glioblastoma 3, Medulloblastoma, Pre-B-cell acute lymphoblastic leukemia. This variant causes a nonsynonymous moderate amino acid substitution (p.V2466A) which may alter protein function. ClinVar classifies this variant as untested, unknown and non-pathogenic and reports an association with Familial cancer of breast, Breast-ovarian cancer (familial 2), not specified. This variant is predicted to be tolerable by SIFT

c.1014G>C

The patient is heterozygous for a common missense polymorphism in the MUTYH gene which occurs at a frequency of 0.31% in the 1000 genomes project. OMIM reports 3 phenotypes associated with variants in the MUTYH gene including Adenomas (multiple colorectal), Colorectal adenomatous polyposis (autosomal recessive) (with pilomatricomas), Gastric cancer (somatic). This variant causes a nonsynonymous conservative amino acid substitution (p.Q338H) which may alter protein function. ClinVar classifies this variant as non-pathogenic and reports an association with , not specified, Neoplastic Syndromes (Hereditary). This variant is predicted to be tolerable by SIFT and possibly deleterious by PolyPhen2.

c.1636A>G

The patient is homogeneous for a common missense polymorphism in the MEN1 gene which occurs at a frequency of 0.83% in the 1000 genomes project. OMIM reports 6 phenotypes associated with variants in the MEN1 gene including Adrenal adenoma (somatic), Angiofibroma (somatic), Carcinoid tumor of lung, Lipoma (somatic), Multiple endocrine neoplasia 1, Parathyroid adenoma (somatic). This variant causes a nonsynonymous moderate amino acid substitution (p.T546A) which may alter protein function. ClinVar classifies this variant as non-pathogenic and reports an association with , not specified. This variant is predicted to be tolerable by SIFT and benign by PolyPhen2.

c.2318G>A

The patient is heterozygous for a common missense polymorphism in the DSG2 gene which occurs at a frequency of 0.24% in the 1000 genomes project. OMIM reports 2 phenotypes associated with variants in the DSG2 gene including Arrhythmogenic right ventricular dysplasia 10, Cardiomyopathy (dilated) (1BB). This variant causes a nonsynonymous conservative amino acid substitution (p.R773K) which may alter protein function. ClinVar classifies this variant as non-pathogenic and reports an association with not specified. This variant is predicted to be tolerable by SIFT and benign by PolyPhen2.

c.5465T>A

The patient is heterozygous for a common missense polymorphism in the APC gene which occurs at a frequency of 0.87% in the 1000 genomes project. OMIM reports 8 phenotypes associated with variants in the APC gene including Adenoma (periampullary) (somatic), Adenomatous polyposis coli, Brain tumor-polyposis syndrome 2, Colorectal cancer (somatic), Desmoid disease (hereditary), Gardner syndrome, Gastric cancer (somatic), Hepatoblastoma (somatic). This variant causes a nonsynonymous severe amino acid substitution (p.V1822D) which may alter protein function. ClinVar classifies this variant as non-pathogenic and other and reports an association with Adenomatous

classifies this variant as non-pathogenic and other and reports an association with Adenomatous polyposis coli, , not specified, Familial colorectal cancer, Neoplastic Syndromes (Hereditary). This variant is predicted to be benign by PolyPhen2.

c.1621A>G

The patient is homogeneous for a common missense polymorphism in the PMS2 gene which occurs at a frequency of 0.88% in the 1000 genomes project. OMIM reports 2 phenotypes associated with variants in the PMS2 gene including Colorectal cancer (hereditary nonpolyposis) (type 4), Mismatch repair cancer syndrome. This variant causes a nonsynonymous moderate amino acid substitution (p.K541E) which may alter protein function. ClinVar classifies this variant as non-pathogenic and reports an association with Lynch syndrome. This variant is predicted to be tolerable by SIFT and benign by PolyPhen2.

c.4059T>G

The patient is homogeneous for a common missense polymorphism in the COL3A1 gene which occurs at a frequency of 1.% in the 1000 genomes project. OMIM reports 2 phenotypes associated with variants in the COL3A1 gene including Ehlers-Danlos syndrome (type III), Ehlers-Danlos syndrome (type IV). This variant causes a nonsynonymous conservative amino acid substitution (p.H1353Q) which may alter protein function. This variant is predicted to be tolerable by SIFT and benign by PolyPhen2.

c.1114A>C

The patient is heterozygous for a common missense polymorphism in the BRCA2 gene which occurs at a frequency of 0.25% in the 1000 genomes project. OMIM reports 9 phenotypes associated with variants in the BRCA2 gene including Fanconi anemia (complementation group D1), Pancreatic cancer, Prostate cancer, Wilms tumor, susceptibility to Breast cancer (male), Breast-ovarian cancer (familial) (2), Glioblastoma 3, Medulloblastoma, Pre-B-cell acute lymphoblastic leukemia. This variant causes a nonsynonymous moderate amino acid substitution (p.N372H) which may alter protein function. ClinVar classifies this variant as other, non-pathogenic and untested and reports an association with Breast-ovarian cancer (familial 2), , not specified, Neoplastic Syndromes (Hereditary). This variant is predicted to be tolerable by SIFT

c.1486C>G

The patient is homogeneous for a common missense polymorphism in the MYLK gene which occurs at a frequency of 1.% in the 1000 genomes project. OMIM reports that variants in the MYLK gene are associated with Aortic aneurysm (familial thoracic 7). This variant causes a nonsynonymous conservative amino acid substitution (p.L496V) which may alter protein function. This variant is predicted to be tolerable by SIFT and benign by PolyPhen2.

RECOMMENDATIONS

Genetic counseling of the family is recommended to discuss the implications of this report.
[Additional recommendations.]

METHODS AND LIMITATIONS

This report is a sample to be used solely for the purpose of evaluating functionality of the Tute Platform and should not be used in any clinical application.

Sequencing and Variant Detection

Whole exome sequencing was performed by solution-based hybridization which enriches for coding exons targeting DNA with specific baits. Sequencing was performed using the Illumina HiSeq 2500 to a minimum average exome depth of 90X. Sequencing reads were aligned to the reference genome (hg19) by BWA-MEM and variants were called using FreeBayes.

Variant Analysis and Report Generation

Reported variants were analyzed using the Tute platform to identify those variants which are reported by medical literature to be pathogenic. All non-synonymous variants of allele frequency <1% reported by the 1000 genomes project and annotated as 'Pathogenic' or 'Probably Pathogenic' by ClinVar are reported in the table 'Primary/Diagnostic Sequence Variant(s) Detected'. Careful correlation of these variants with the clinical indications for the proband should be conducted by an experience medical geneticist to verify that reported findings are consistent with reported clinical phenotypes. Additionally, variants are included based on ACMG guidelines for the reporting of incidental findings in the table 'Secondary/Incidental Sequence Variant(s) Detected'.

Validation of reported findings

Variants of interest should be confirmed by Sanger sequencing. The DNA immediately flanking each of these variants should be amplified genomic DNA followed by sequencing in both directions using fluorescent dideoxy sequencing.

Limitations

Absence of a primary diagnostic finding identified by this test does not exclude the possibility of a genetic basis for the clinical condition for this proband. Certain genetic abnormalities are not detectable by the technologies used to generate this report. Specifically, detection of abnormal variants depends on the presence of these sequence variants in the sequencing data. This exome sequence test is designed to evaluate single nucleotide variants within the human exome; however, this technology is only able to sequence 90-95% of the human reference to the requisite 10-fold coverage needed for reliable detection of heterozygous variants.

Additionally, certain types of genetic abnormalities are difficult to identify in sequencing data and have not been validated for clinical use including insertions, deletions, copy number alterations, long repetitive sequences, triplet repeat expansions, chromosomal rearrangements, polyploidy, repetitive regions including mono-, di- and tri-nucleotide repeats, GX rich regions, intronic variants outside the splice-site and epigenetic effects.

Variants believed to be benign based on medical literature, or with population frequencies greater than or equal to 1%, or resulting in synonymous amino acid changes, or occurring in 5' or 3' untranslated regions are generally not reported.

This report is a sample to be used solely for the purpose of evaluating functionality of the Tute Platform and should not be used in any clinical application.

Sources

REPORT SIGNATURES

Online Mendelian Inheritance in Man, OMIM (TM). Retrieved 0/26/2015 from http://www.ncbi.nlm.nih.gov/omim/
ClinVar. Retrieved 0/26/2015 from http://www.ncbi.nlm.nih.gov/clinvar/

TUTE GENOMICS

NextStep Dx PLUS report for K10031 10231 annotation

ORDERING PROVIDER	SPECIMEN	PATIENT
,	Collection Date:	Name:
	Received Date:	Date of Birth:
	Report Date:	Patient ID:
		Gender:

Clinical Indication:

[Detailed clinical description.]

TEST RESULTS SUMMARY

Primary Result(s): [Findings related to clinical indication.]

Incidental/Secondary Result(s): [Incidental findings.]

PRIMARY/DIAGNOSTIC SEQUENCE VARIANT(S) DETECTED

Gene	Nucleotide Change	Protein Change	Zygosity	Tute Dx
HFE	c.845G>A	p.C282Y	hom	Hereditary hemochromatosis|Hemochromatosis, juvenile, digenic
CTH	c.200C>T	p.T67I	het	Cystathioninuria
CFTR	c.224G>A	p.R75Q	het	Hereditary pancreatitis;Cystic fibrosis
NTRK1	c.1810C>T	p.H604Y	het	Familial medullary thyroid carcinoma|Hereditary insensitivity to pain with anhidrosis
BRIP1	c.139C>G	p.P47A	het	Breast cancer, early-onset
F5	c.1601A>G	p.Q534R	het	Thrombophilia due to factor V Leiden
DPYD	c.85C>T	p.R29C	hom	Dihydropyrimidine dehydrogenase deficiency;not provided
PRSS1	c.86A>T	p.N29I	het	Hereditary pancreatitis;Hereditary pancreatitis
FUT2	c.461G>A	p.W154X	hom	Norwalk virus infection, resistance to
CYP2D6	c.506-1G>A	.	het	Debrisoquine, poor metabolism of
PRSS1	c.47C>T	p.A16V	het	Hereditary pancreatitis
NTRK1	c.1838G>T	p.G613V	het	Familial medullary thyroid carcinoma|Hereditary insensitivity to pain with anhidrosis
PRSS1	c.161A>G	p.N54S	het	Hereditary pancreatitis
FGFR4	c.1162G>A	p.G388R	het	Cancer progression and tumor cell motility
CYP2B6	c.785A>G	p.K262R	het	Efavirenz, poor metabolism of
ITGB3	c.176T>C	p.L59P	het	Thrombocytopenia, neonatal alloimmune|Posttransfusion purpura
TIRAP	c.539C>T	p.S180L	het	Malaria, resistance to|BACTEREMIA, RESISTANCE TO
CST3	c.73G>A	p.A25T	het	Age-related macular degeneration 11
HEXB	c.185T>C	p.L62S	hom	Sandhoff disease, infantile type
BANK1	c.182G>A	p.R61H	het	Systemic lupus erythmatosus, association with

SECONDARY/INCIDENTAL SEQUENCE VARIANT(S) DETECTED

The American College of Medical Genetics and Genomics (ACMG) recently published a policy statement on clinical sequencing that emphasizes the possibility of recognizing and reporting incidental or secondary findings of medical value when conducting exome and genome sequencing. The ACMG recommends that laboratories performing clinical sequencing seek and report mutations of specified classes or types in the genes listed within the guidelines. This section of the report describes variants which fit the criteria outlined by ACMG for the reporting of incidental or secondary findings.

Gene	Nucleotide Change	Protein Change	Zygosity	Tute Dx
MUTYH	c.1276C>T	p.R426C	het	Adenomatous polyposis coli
SDHD	c.371A>G	p.Q124R	hom	.
CACNA1S	c.5399T>C	p.L1800S	het	.
FBN1	c.1415G>A	p.C472Y	hom	.
BRCA2	c.7397T>C	p.V2466A	hom	Breast cancer
MUTYH	c.1014G>C	p.Q338H	het	.
MEN1	c.1636A>G	p.T546A	hom	.
BRCA1	c.2612C>T	p.P871L	het	.
DSG2	c.2318G>A	p.R773K	het	.
APC	c.5465T>A	p.V1822D	hom	.
PMS2	c.1621A>G	p.K541E	hom	Colorectal cancer, non-polyposis
COL3A1	c.4059T>G	p.H1353Q	hom	.
BRCA1	c.2077G>A	p.D693N	het	.
BRCA2	c.1114A>C	p.N372H	het	.
MYLK	c.1486C>G	p.L496V	hom	.
PCSK9	c.1420G>A	p.V474I	hom	.
APOB	c.13013G>A	p.S4338N	hom	.
PCSK9	c.2009G>A	p.G670E	hom	.
DSC2	c.2393G>A	p.R798Q	het	.
APOB	c.6937A>G	p.I2313V	hom	.

INTERPRETATION OF PRIMARY FINDINGS

c.845G>A

The patient is homogeneous for a common missense polymorphism in the HFE gene which occurs at a frequency of 0.013% in the 1000 genomes project. OMIM reports 6 phenotypes associated with variants in the HFE gene including Hemochromatosis, [Transferrin serum level QTL2], susceptibility to Alzheimer disease, Microvascular complications of diabetes 7, susceptibility to Porphyria cutanea tarda, susceptibility to Porphyria variegata. This variant causes a nonsynonymous severe amino acid substitution (p.C282Y) which may alter protein function. ClinVar classifies this variant as pathogenic, other and non-pathogenic and reports an association with Hereditary

hemochromatosis, Porphyria cutanea tarda (susceptibility to), Porphyria variegata (susceptibility to), Hemochromatosis (juvenile) (digenic), Alzheimer disease (susceptibility to), Transferrin serum level quantitative trait locus 2, Microvascular complications of diabetes 7, not specified. This variant is predicted to be deleterious by SIFT and deleterious by PolyPhen2.

c.200C>T

The patient is heterozygous for a common missense polymorphism in the CTH gene which occurs at a frequency of 0.0026% in the 1000 genomes project. OMIM reports 2 phenotypes associated with variants in the CTH gene including Cystathioninuria, Homocysteine (total plasma) (elevated). This variant causes a nonsynonymous moderate amino acid substitution (p.T67I) which may alter protein function. ClinVar classifies this variant as pathogenic and reports an association with Cystathioninuria. This variant is predicted to be deleterious by SIFT and deleterious by PolyPhen2.

c.224G>A

The patient is heterozygous for a common missense polymorphism in the CFTR gene which occurs at a frequency of 0.0064% in the 1000 genomes project. OMIM reports 6 phenotypes associated with variants in the CFTR gene including Congenital bilateral absence of vas deferens, Cystic fibrosis, Sweat chloride elevation without CF, modifier of Bronchiectasis with or without elevated sweat chloride 1, Hypertrypsinemia (neonatal), Pancreatitis (idiopathic). This variant causes a nonsynonymous conservative amino acid substitution (p.R75Q) which may alter protein function. ClinVar classifies this variant as probable-non-pathogenic, unknown, non-pathogenic and pathogenic and reports an association with Cystic fibrosis, , not specified, Hereditary pancreatitis;Cystic fibrosis. This variant is predicted to be deleterious by SIFT and deleterious by PolyPhen2.

c.1810C>T

The patient is heterozygous for a common missense polymorphism in the NTRK1 gene which occurs at a frequency of 0.024% in the 1000 genomes project. OMIM reports 2 phenotypes associated with variants in the NTRK1 gene including Insensitivity to pain (congenital) (with anhidrosis), Medullary thyroid carcinoma (familial). This variant causes a nonsynonymous moderate amino acid substitution (p.H604Y) which may alter protein function. ClinVar classifies this variant as pathogenic and non-pathogenic and reports an association with Familial medullary thyroid carcinoma, Hereditary insensitivity to pain with anhidrosis, Hereditary insensitivity to pain with anhidrosis. This variant is predicted to be deleterious by SIFT and deleterious by PolyPhen2.

c.139C>G

The patient is heterozygous for a rare missense mutation in the BRIP1 gene which doesn't occur in the 1000 genomes project. OMIM reports 2 phenotypes associated with variants in the BRIP1 gene including Breast cancer (early-onset), Fanconi anemia (complementation group J). This variant causes a nonsynonymous conservative amino acid substitution (p.P47A) which may alter protein function. ClinVar classifies this variant as pathogenic and other and reports an association with Breast cancer (early-onset), Neoplastic Syndromes (Hereditary). This variant is predicted to be deleterious by PolyPhen2.

c.1601A>G

The patient is heterozygous for a common missense polymorphism in the F5 gene which occurs at a frequency of 0.99% in the 1000 genomes project. OMIM reports 6 phenotypes associated with variants in the F5 gene including Factor V deficiency, Thrombophilia due to activated protein C resistance, Budd-Chiari syndrome, susceptibility to Pregnancy loss recurrent 1, susceptibility to Stroke (ischemic), susceptibility to Thrombophilia due to factor V Leiden. This variant causes a nonsynonymous conservative amino acid substitution (p.Q534R) which may alter protein function. ClinVar classifies this variant as pathogenic, untested and other and reports an association with Thrombophilia due to factor V Leiden, Ischemic stroke (susceptibility to), Budd-Chiari syndrome (susceptibility to), Recurrent abortion. This variant is predicted to be tolerable by SIFT

c.85C>T

The patient is homogeneous for a common missense polymorphism in the DPYD gene which occurs at a frequency of 0.74% in the 1000 genomes project. OMIM reports [illegible] phenotypes

occurs at a frequency of 0.74% in the 1000 genomes project. OMIM reports 2 phenotypes associated with variants in the DPYD gene including 5-fluorouracil toxicity, Dihydropyrimidine dehydrogenase deficiency. This variant causes a nonsynonymous severe amino acid substitution (p.R29C) which may alter protein function. ClinVar classifies this variant as pathogenic and reports an association with Dihydropyrimidine dehydrogenase deficiency;. This variant is predicted to be tolerable by SIFT and deleterious by PolyPhen2.

c.86A>T

The patient is heterozygous for a common missense polymorphism in the PRSS1 gene which doesn't occur in the 1000 genomes project. OMIM reports 2 phenotypes associated with variants in the PRSS1 gene including Pancreatitis (hereditary), Trypsinogen deficiency. This variant causes a nonsynonymous moderate amino acid substitution (p.N29I) which may alter protein function. ClinVar classifies this variant as pathogenic and reports an association with Hereditary pancreatitis;Hereditary pancreatitis. This variant is predicted to be deleterious by SIFT and benign by PolyPhen2.

c.461G>A

The patient is homogeneous for a common nonsense polymorphism in the FUT2 gene which occurs at a frequency of 0.32% in the 1000 genomes project. OMIM reports 3 phenotypes associated with variants in the FUT2 gene including [Bombay phenotype], resistance to Norwalk virus infection, Vitamin B12 plasma level QTL1. This variant introduces a premature stop codon which may lead to early termination of protein transcription. ClinVar classifies this variant as non-pathogenic, pathogenic and other and reports an association with SECRETOR/NONSECRETOR POLYMORPHISM, Norwalk virus infection (resistance to), Vitamin b12 plasma level quantitative trait locus 1. This variant is predicted to be tolerable by SIFT

c.506-1G>A

The patient is heterozygous for a common polymorphism in the CYP2D6 gene which occurs at a frequency of 0.093% in the 1000 genomes project. OMIM reports 2 phenotypes associated with variants in the CYP2D6 gene including Codeine sensitivity, Debrisoquine sensitivity. ClinVar classifies this variant as pathogenic and reports an association with Debrisoquine (poor metabolism of).

c.47C>T

The patient is heterozygous for a common missense polymorphism in the PRSS1 gene which doesn't occur in the 1000 genomes project. OMIM reports 2 phenotypes associated with variants in the PRSS1 gene including Pancreatitis (hereditary), Trypsinogen deficiency. This variant causes a nonsynonymous moderate amino acid substitution (p.A16V) which may alter protein function. ClinVar classifies this variant as pathogenic and reports an association with Hereditary pancreatitis. This variant is predicted to be tolerable by SIFT and benign by PolyPhen2.

c.1838G>T

The patient is heterozygous for a common missense polymorphism in the NTRK1 gene which occurs at a frequency of 0.023% in the 1000 genomes project. OMIM reports 2 phenotypes associated with variants in the NTRK1 gene including Insensitivity to pain (congenital) (with anhidrosis), Medullary thyroid carcinoma (familial). This variant causes a nonsynonymous moderate amino acid substitution (p.G613V) which may alter protein function. ClinVar classifies this variant as pathogenic and non-pathogenic and reports an association with Familial medullary thyroid carcinoma, Hereditary insensitivity to pain with anhidrosis, Hereditary insensitivity to pain with anhidrosis. This variant is predicted to be tolerable by SIFT and possibly deleterious by PolyPhen2.

c.161A>G

The patient is heterozygous for a common missense polymorphism in the PRSS1 gene which doesn't occur in the 1000 genomes project. OMIM reports 2 phenotypes associated with variants in the PRSS1 gene including Pancreatitis (hereditary), Trypsinogen deficiency. This variant causes a nonsynonymous conservative amino acid substitution (p.N54S) which may alter protein function. ClinVar classifies this variant as pathogenic and reports an association with Hereditary pancreatitis. This variant is predicted to be tolerable by SIFT and benign by PolyPhen2.

This variant is predicted to be tolerable by SIFT and benign by PolyPhen2.

c.1162G>A

The patient is heterozygous for a common missense polymorphism in the FGFR4 gene which occurs at a frequency of 0.3% in the 1000 genomes project. OMIM reports that variants in the FGFR4 gene are associated with Cancer progression/metastasis. This variant causes a nonsynonymous moderate amino acid substitution (p.G388R) which may alter protein function. ClinVar classifies this variant as pathogenic and reports an association with Cancer progression and tumor cell motility. This variant is predicted to be deleterious by SIFT and deleterious by PolyPhen2.

c.785A>G

The patient is heterozygous for a common missense polymorphism in the CYP2B6 gene which doesn't occur in the 1000 genomes project. OMIM reports 2 phenotypes associated with variants in the CYP2B6 gene including poor metabolism of Efavirenz, susceptibility to Efavirenz central nervous system toxicity. This variant causes a nonsynonymous conservative amino acid substitution (p.K262R) which may alter protein function. ClinVar classifies this variant as pathogenic and reports an association with Efavirenz (poor metabolism of). This variant is predicted to be tolerable by SIFT and benign by PolyPhen2.

c.176T>C

The patient is heterozygous for a common missense polymorphism in the ITGB3 gene which occurs at a frequency of 0.089% in the 1000 genomes project. OMIM reports 5 phenotypes associated with variants in the ITGB3 gene including Bleeding disorder (platelet-type) (16) (autosomal dominant), Glanzmann thrombasthenia, Purpura (posttransfusion), Thrombocytopenia (neonatal alloimmune), susceptibility to Myocardial infarction. This variant causes a nonsynonymous moderate amino acid substitution (p.L59P) which may alter protein function. ClinVar classifies this variant as non-pathogenic, pathogenic and other and reports an association with PL(A1)/(A2) ALLOANTIGEN POLYMORPHISM, Thrombocytopenia (neonatal alloimmune), Posttransfusion purpura, Myocardial infarction, Fracture (hip) (susceptibility to). This variant is predicted to be tolerable by SIFT and benign by PolyPhen2.

c.539C>T

The patient is heterozygous for a common missense polymorphism in the TIRAP gene which occurs at a frequency of 0.086% in the 1000 genomes project. OMIM reports 4 phenotypes associated with variants in the TIRAP gene including protection against Bacteremia, protection against Malaria, protection against Pneumococcal disease (invasive), protection against Tuberculosis. This variant causes a nonsynonymous moderate amino acid substitution (p.S180L) which may alter protein function. ClinVar classifies this variant as other and pathogenic and reports an association with Invasive pneumococcal disease (protection against), Malaria (resistance to), Mycobacterium tuberculosis (protection against), BACTEREMIA (RESISTANCE TO). This variant is predicted to be possibly deleterious by PolyPhen2.

c.73G>A

The patient is heterozygous for a common missense polymorphism in the CST3 gene which occurs at a frequency of 0.21% in the 1000 genomes project. OMIM reports 2 phenotypes associated with variants in the CST3 gene including Cerebral amyloid angiopathy, Macular degeneration (age-related) (11). This variant causes a nonsynonymous moderate amino acid substitution (p.A25T) which may alter protein function. ClinVar classifies this variant as pathogenic and reports an association with Age-related macular degeneration 11. This variant is predicted to be tolerable by SIFT and benign by PolyPhen2.

c.185T>C

The patient is homogeneous for a common missense polymorphism in the HEXB gene which occurs at a frequency of 0.98% in the 1000 genomes project. OMIM reports that variants in the HEXB gene are associated with Sandhoff disease (infantile) (juvenile) (and adult forms). This variant causes a nonsynonymous moderate amino acid substitution (p.L62S) which may alter protein function. ClinVar classifies this variant as pathogenic and reports an association with Sandhoff

disease (infantile type). This variant is predicted to be tolerable by SIFT

c.182G>A
The patient is heterozygous for a common missense polymorphism in the BANK1 gene which occurs at a frequency of 0.22% in the 1000 genomes project. OMIM reports that variants in the BANK1 gene are associated with association with Systemic lupus erythematosus. This variant causes a nonsynonymous conservative amino acid substitution (p.R61H) which may alter protein function. ClinVar classifies this variant as pathogenic and reports an association with Systemic lupus erythmatosus (association with). This variant is predicted to be tolerable by SIFT and benign by PolyPhen2.

INTERPRETATION OF SECONDARY FINDINGS

---

c.1276C>T
The patient is heterozygous for a rare missense mutation in the MUTYH gene which doesn't occur in the 1000 genomes project. OMIM reports 3 phenotypes associated with variants in the MUTYH gene including Adenomas (multiple colorectal), Colorectal adenomatous polyposis (autosomal recessive) (with pilomatricomas), Gastric cancer (somatic). This variant causes a nonsynonymous severe amino acid substitution (p.R426C) which may alter protein function. ClinVar classifies this variant as unknown and other and reports an association with , Neoplastic Syndromes (Hereditary), MYH-associated polyposis. Invitae classifies this variant as Variant of uncertain significance. This variant is predicted to be tolerable by SIFT and deleterious by PolyPhen2.

c.371A>G
The patient is homogeneous for a common missense polymorphism in the SDHD gene which occurs at a frequency of 0.95% in the 1000 genomes project. OMIM reports 6 phenotypes associated with variants in the SDHD gene including Carcinoid tumors (intestinal), Cowden syndrome 3, Merkel cell carcinoma (somatic), Paraganglioma and gastric stromal sarcoma, Paragangliomas 1 (with or without deafness), Pheochromocytoma. This variant causes a nonsynonymous conservative amino acid substitution (p.Q124R) which may alter protein function. This variant is predicted to be tolerable by SIFT

c.5399T>C
The patient is heterozygous for a common missense polymorphism in the CACNA1S gene which occurs at a frequency of 0.23% in the 1000 genomes project. OMIM reports 3 phenotypes associated with variants in the CACNA1S gene including Hypokalemic periodic paralysis (type 1), Malignant hyperthermia susceptibility 5, susceptibility to Thyrotoxic periodic paralysis 1. This variant causes a nonsynonymous moderate amino acid substitution (p.L1800S) which may alter protein function. This variant is predicted to be deleterious by SIFT and deleterious by PolyPhen2.

c.1415G>A
The patient is homogeneous for a common missense polymorphism in the FBN1 gene which occurs at a frequency of 1.% in the 1000 genomes project. OMIM reports 8 phenotypes associated with variants in the FBN1 gene including Acromicric dysplasia, Aortic aneurysm (ascending) (and dissection), Ectopia lentis (familial), Geleophysic dysplasia 2, MASS syndrome, Marfan syndrome, Stiff skin syndrome, Weill-Marchesani syndrome 2 (dominant). This variant causes a nonsynonymous severe amino acid substitution (p.C472Y) which may alter protein function. This variant is predicted to be tolerable by SIFT and benign by PolyPhen2.

c.7397T>C
The patient is homogeneous for a common missense polymorphism in the BRCA2 gene which occurs at a frequency of 0.98% in the 1000 genomes project. OMIM reports 9 phenotypes associated with variants in the BRCA2 gene including Fanconi anemia (complementation group D1), Pancreatic cancer, Prostate cancer, Wilms tumor, susceptibility to Breast cancer (male), Breast- [illegible]

ovarian cancer (familial) (2), Glioblastoma 3, Medulloblastoma, Pre-B-cell acute lymphoblastic leukemia. This variant causes a nonsynonymous moderate amino acid substitution (p.V2466A) which may alter protein function. ClinVar classifies this variant as untested, unknown and non-pathogenic and reports an association with Familial cancer of breast, Breast-ovarian cancer (familial 2), not specified. This variant is predicted to be tolerable by SIFT

c.1014G>C

The patient is heterozygous for a common missense polymorphism in the MUTYH gene which occurs at a frequency of 0.31% in the 1000 genomes project. OMIM reports 3 phenotypes associated with variants in the MUTYH gene including Adenomas (multiple colorectal), Colorectal adenomatous polyposis (autosomal recessive) (with pilomatricomas), Gastric cancer (somatic). This variant causes a nonsynonymous conservative amino acid substitution (p.Q338H) which may alter protein function. ClinVar classifies this variant as non-pathogenic and reports an association with , not specified, Neoplastic Syndromes (Hereditary). This variant is predicted to be tolerable by SIFT and possibly deleterious by PolyPhen2.

c.1636A>G

The patient is homogeneous for a common missense polymorphism in the MEN1 gene which occurs at a frequency of 0.83% in the 1000 genomes project. OMIM reports 6 phenotypes associated with variants in the MEN1 gene including Adrenal adenoma (somatic), Angiofibroma (somatic), Carcinoid tumor of lung, Lipoma (somatic), Multiple endocrine neoplasia 1, Parathyroid adenoma (somatic). This variant causes a nonsynonymous moderate amino acid substitution (p.T546A) which may alter protein function. ClinVar classifies this variant as non-pathogenic and reports an association with , not specified. This variant is predicted to be tolerable by SIFT and benign by PolyPhen2.

c.2612C>T

The patient is heterozygous for a common missense polymorphism in the BRCA1 gene which occurs at a frequency of 0.54% in the 1000 genomes project. OMIM reports 2 phenotypes associated with variants in the BRCA1 gene including Breast-ovarian cancer (familial) (1), susceptibility to Pancreatic cancer 4. This variant causes a nonsynonymous moderate amino acid substitution (p.P871L) which may alter protein function. This variant is predicted to be tolerable by SIFT and benign by PolyPhen2.

c.2318G>A

The patient is heterozygous for a common missense polymorphism in the DSG2 gene which occurs at a frequency of 0.24% in the 1000 genomes project. OMIM reports 2 phenotypes associated with variants in the DSG2 gene including Arrhythmogenic right ventricular dysplasia 10, Cardiomyopathy (dilated) (1BB). This variant causes a nonsynonymous conservative amino acid substitution (p.R773K) which may alter protein function. ClinVar classifies this variant as non-pathogenic and reports an association with not specified. This variant is predicted to be tolerable by SIFT and benign by PolyPhen2.

c.5465T>A

The patient is homogeneous for a common missense polymorphism in the APC gene which occurs at a frequency of 0.87% in the 1000 genomes project. OMIM reports 8 phenotypes associated with variants in the APC gene including Adenoma (periampullary) (somatic), Adenomatous polyposis coli, Brain tumor-polyposis syndrome 2, Colorectal cancer (somatic), Desmoid disease (hereditary), Gardner syndrome, Gastric cancer (somatic), Hepatoblastoma (somatic). This variant causes a nonsynonymous severe amino acid substitution (p.V1822D) which may alter protein function. ClinVar classifies this variant as non-pathogenic and other and reports an association with Adenomatous polyposis coli, , not specified, Familial colorectal cancer, Neoplastic Syndromes (Hereditary). This variant is predicted to be benign by PolyPhen2.

c.1621A>G

The patient is homogeneous for a common missense polymorphism in the PMS2 gene which occurs at a frequency of 0.88% in the 1000 genomes project. OMIM reports 2 phenotypes

occurs at a frequency of 0.88% in the 1000 genomes project. OMIM reports 2 phenotypes associated with variants in the PMS2 gene including Colorectal cancer (hereditary nonpolyposis) (type 4), Mismatch repair cancer syndrome. This variant causes a nonsynonymous moderate amino acid substitution (p.K541E) which may alter protein function. ClinVar classifies this variant as non-pathogenic and reports an association with Lynch syndrome. This variant is predicted to be tolerable by SIFT and benign by PolyPhen2.

c.4059T>G

The patient is homogeneous for a common missense polymorphism in the COL3A1 gene which occurs at a frequency of 1.% in the 1000 genomes project. OMIM reports 2 phenotypes associated with variants in the COL3A1 gene including Ehlers-Danlos syndrome (type III), Ehlers-Danlos syndrome (type IV). This variant causes a nonsynonymous conservative amino acid substitution (p.H1353Q) which may alter protein function. This variant is predicted to be tolerable by SIFT and benign by PolyPhen2.

c.2077G>A

The patient is heterozygous for a common missense polymorphism in the BRCA1 gene which occurs at a frequency of 0.034% in the 1000 genomes project. OMIM reports 2 phenotypes associated with variants in the BRCA1 gene including Breast-ovarian cancer (familial) (1), susceptibility to Pancreatic cancer 4. This variant causes a nonsynonymous conservative amino acid substitution (p.D693N) which may alter protein function. ClinVar classifies this variant as non-pathogenic and reports an association with Breast-ovarian cancer (familial 1);, BRCA1 and BRCA2 Hereditary Breast and Ovarian Cancer, Breast-ovarian cancer (familial 1), not specified, Neoplastic Syndromes (Hereditary). ARUP classifies this variant as 1 - Not pathogenic or of no clinical significance. Invitae classifies this variant as Benign. This variant is predicted to be deleterious by SIFT and benign by PolyPhen2.

c.1114A>C

The patient is heterozygous for a common missense polymorphism in the BRCA2 gene which occurs at a frequency of 0.25% in the 1000 genomes project. OMIM reports 9 phenotypes associated with variants in the BRCA2 gene including Fanconi anemia (complementation group D1), Pancreatic cancer, Prostate cancer, Wilms tumor, susceptibility to Breast cancer (male), Breast-ovarian cancer (familial) (2), Glioblastoma 3, Medulloblastoma, Pre-B-cell acute lymphoblastic leukemia. This variant causes a nonsynonymous moderate amino acid substitution (p.N372H) which may alter protein function. ClinVar classifies this variant as other, non-pathogenic and untested and reports an association with Breast-ovarian cancer (familial 2), , not specified, Neoplastic Syndromes (Hereditary). This variant is predicted to be tolerable by SIFT

c.1486C>G

The patient is homogeneous for a common missense polymorphism in the MYLK gene which occurs at a frequency of 1.% in the 1000 genomes project. OMIM reports that variants in the MYLK gene are associated with Aortic aneurysm (familial thoracic 7). This variant causes a nonsynonymous conservative amino acid substitution (p.L496V) which may alter protein function. This variant is predicted to be tolerable by SIFT and benign by PolyPhen2.

c.1420G>A

The patient is homogeneous for a common missense polymorphism in the PCSK9 gene which occurs at a frequency of 0.87% in the 1000 genomes project. OMIM reports 2 phenotypes associated with variants in the PCSK9 gene including Hypercholesterolemia (familial) (3), Low density lipoprotein cholesterol level QTL 1. This variant causes a nonsynonymous conservative amino acid substitution (p.V474I) which may alter protein function. This variant is predicted to be tolerable by SIFT and benign by PolyPhen2.

c.13013G>A

The patient is homogeneous for a common missense polymorphism in the APOB gene which occurs at a frequency of 0.63% in the 1000 genomes project. OMIM reports 2 phenotypes associated with variants in the APOB gene including Hypercholesterolemia (due to ligand-defective

[illegible]
apo B), Hypobetalipoproteinemia. This variant causes a nonsynonymous conservative amino acid substitution (p.S4338N) which may alter protein function. ClinVar classifies this variant as probable-non-pathogenic and reports an association with not specified. This variant is predicted to be tolerable by SIFT and benign by PolyPhen2.

c.2009G>A

The patient is homogeneous for a common missense polymorphism in the PCSK9 gene which occurs at a frequency of 0.9% in the 1000 genomes project. OMIM reports 2 phenotypes associated with variants in the PCSK9 gene including Hypercholesterolemia (familial) (3), Low density lipoprotein cholesterol level QTL 1. This variant causes a nonsynonymous moderate amino acid substitution (p.G670E) which may alter protein function. ClinVar classifies this variant as non-pathogenic and reports an association with Familial hypercholesterolemia. This variant is predicted to be tolerable by SIFT and benign by PolyPhen2.

c.2393G>A

The patient is heterozygous for a common missense polymorphism in the DSC2 gene which occurs at a frequency of 0.028% in the 1000 genomes project. OMIM reports 2 phenotypes associated with variants in the DSC2 gene including Arrhythmogenic right ventricular dysplasia 11 with mild palmoplantar keratoderma and woolly hair, Arrhythmogenic right ventricular dysplasia 11. This variant causes a nonsynonymous conservative amino acid substitution (p.R798Q) which may alter protein function. ClinVar classifies this variant as unknown and reports an association with not specified;Arrhythmogenic right ventricular cardiomyopathy, not specified. This variant is predicted to be tolerable by SIFT and benign by PolyPhen2.

c.6937A>G

The patient is homogeneous for a common missense polymorphism in the APOB gene which occurs at a frequency of 0.99% in the 1000 genomes project. OMIM reports 2 phenotypes associated with variants in the APOB gene including Hypercholesterolemia (due to ligand-defective apo B), Hypobetalipoproteinemia. This variant causes a nonsynonymous conservative amino acid substitution (p.I2313V) which may alter protein function. This variant is predicted to be tolerable by SIFT and benign by PolyPhen2.

RECOMMENDATIONS

Genetic counseling of the family is recommended to discuss the implications of this report.
[Additional recommendations.]

METHODS AND LIMITATIONS

This report is a sample to be used solely for the purpose of evaluating functionality of the Tute Platform and should not be used in any clinical application.

Sequencing and Variant Detection

Whole exome sequencing was performed by solution-based hybridization which enriches for coding exons targeting DNA with specific baits. Sequencing was performed using the Illumina HiSeq 2500 to a minimum average exome depth of 90X. Sequencing reads were aligned to the reference genome (hg19) by BWA-MEM and variants were called using FreeBayes.

Variant Analysis and Report Generation

Reported variants were analyzed using the Tute platform to identify those variants which are reported by medical literature to be pathogenic. All non-synonymous variants of allele frequency <1% reported by the 1000 genomes project and annotated as 'Pathogenic' or 'Probably Pathogenic' by ClinVar are reported in the table 'Primary/Diagnostic Sequence Variant(s) Detected'. Careful correlation of these variants with the clinical indications for the proband should be conducted by an experience medical geneticist to verify that reported findings are consistent with reported clinical phenotypes. Additionally, variants are included based on ACMG guidelines for the reporting of incidental findings in the table 'Secondary/Incidental Sequence Variant(s) Detected'.

Validation of reported findings

Variants of interest should be confirmed by Sanger sequencing. The DNA immediately flanking each of these variants should be amplified genomic DNA followed by sequencing in both directions using fluorescent dideoxy sequencing.

Limitations

Absence of a primary diagnostic finding identified by this test does not exclude the possibility of a genetic basis for the clinical condition for this proband. Certain genetic abnormalities are not detectable by the technologies used to generate this report. Specifically, detection of abnormal variants depends on the presence of these sequence variants in the sequencing data. This exome sequence test is designed to evaluate single nucleotide variants within the human exome; however, this technology is only able to sequence 90-95% of the human reference to the requisite 10-fold coverage needed for reliable detection of heterozygous variants.

Additionally, certain types of genetic abnormalities are difficult to identify in sequencing data and have not been validated for clinical use including insertions, deletions, copy number alterations, long repetitive sequences, triplet repeat expansions, chromosomal rearrangements, polyploidy, repetitive regions including mono-, di- and tri-nucleotide repeats, GX rich regions, intronic variants outside the splice-site and epigenetic effects.

Variants believed to be benign based on medical literature, or with population frequencies greater than or equal to 1%, or resulting in synonymous amino acid changes, or occurring in 5' or 3' untranslated regions are generally not reported.

This report is a sample to be used solely for the purpose of evaluating functionality of the Tute Platform and should not be used in any clinical application.

Sources

REPORT SIGNATURES

Online Mendelian Inheritance in Man, OMIM (TM). Retrieved 0/26/2015 from http://www.ncbi.nlm.nih.gov/omim/
ClinVar. Retrieved 0/26/2015 from http://www.ncbi.nlm.nih.gov/clinvar/

NextStep Dx PLUS report for K10031 10233 annotation

ORDERING PROVIDER

,

SPECIMEN

Collection Date:
Received Date:
Report Date:

PATIENT

Name:
Date of Birth:
Patient ID:
Gender:

Clinical Indication:

[Detailed clinical description.]

TEST RESULTS SUMMARY

Primary Result(s): [Findings related to clinical indication.]

Incidental/Secondary Result(s): [Incidental findings.]

PRIMARY/DIAGNOSTIC SEQUENCE VARIANT(S) DETECTED

Gene	Nucleotide Change	Protein Change	Zygosity	Tute Dx
HFE	c.845G>A	p.C282Y	het	Hereditary hemochromatosis|Hemochromatosis, juvenile, digenic
CFTR	c.224G>A	p.R75Q	het	Hereditary pancreatitis;Cystic fibrosis
GNPTAB	c.569A>G	p.D190G	het	Mucopolysaccharidosis, MPS-III-A
F5	c.1601A>G	p.Q534R	het	Thrombophilia due to factor V Leiden
DPYD	c.85C>T	p.R29C	hom	Dihydropyrimidine dehydrogenase deficiency;not provided
SPINK1	c.194+2T>C	.	het	Hereditary pancreatitis
PRSS1	c.86A>T	p.N29I	het	Hereditary pancreatitis;Hereditary pancreatitis
FUT2	c.461G>A	p.W154X	het	Norwalk virus infection, resistance to
CFTR	c.1666A>G	p.I556V	het	Cystic fibrosis|Nonclassic cystic fibrosis
SCN5A	c.3578G>A	p.R1193Q	het	Brugada syndrome 1
FGFR4	c.1162G>A	p.G388R	het	Cancer progression and tumor cell motility
ITGB3	c.176T>C	p.L59P	het	Thrombocytopenia, neonatal alloimmune|Posttransfusion purpura
TIRAP	c.539C>T	p.S180L	het	Malaria, resistance to|BACTEREMIA, RESISTANCE TO
HEXB	c.185T>C	p.L62S	hom	Sandhoff disease, infantile type
BBS2	c.209G>A	p.S70N	hom	Bardet-Biedl syndrome 2
ITGA2B	c.2621T>G	p.I874S	hom	Bak platelet-specific antigen
IL4R	c.1462T>C	p.S488P	het	Atopy, resistance to
SLC24A5	c.331A>G	p.T111A	het	Skin/hair/eye pigmentation, variation in, 4
HPD	c.97A>G	p.T33A	hom	4-Alpha-hydroxyphenylpyruvate hydroxylase deficiency
CYP4V2	c.64C>G	p.L22V	het	Bietti crystalline corneoretinal dystrophy

SECONDARY/INCIDENTAL SEQUENCE VARIANT(S) DETECTED

The American College of Medical Genetics and Genomics (ACMG) recently published a policy statement on clinical sequencing that emphasizes the possibility of recognizing and reporting incidental or secondary findings of medical value when conducting exome and genome sequencing. The ACMG recommends that laboratories performing clinical sequencing seek and report mutations of specified classes or types in the genes listed within the guidelines. This section of the report describes variants which fit the criteria outlined by ACMG for the reporting of incidental or secondary findings.

Gene	Nucleotide Change	Protein Change	Zygosity	Tute Dx
MSH2	c.1168C>T	p.L390F	het	Colorectal cancer, non-polyposis
KCNQ1	c.1927G>A	p.G643S	het	.
MSH6	c.3143A>T	p.Q1048L	het	.
SCN5A	c.3578G>A	p.R1193Q	het	Brugada syndrome 1
SDHD	c.371A>G	p.Q124R	hom	.
FBN1	c.1415G>A	p.C472Y	hom	.
DSC2	c.2326A>G	p.I776V	het	.
APOB	c.1853C>T	p.A618V	het	.
TP53	c.215C>G	p.P72R	het	.
BRCA2	c.7397T>C	p.V2466A	hom	Breast cancer
MUTYH	c.1014G>C	p.Q338H	het	.
MEN1	c.1636A>G	p.T546A	hom	.
BRCA1	c.2612C>T	p.P871L	het	.
DSG2	c.2318G>A	p.R773K	hom	.
APC	c.5465T>A	p.V1822D	hom	.
PMS2	c.1621A>G	p.K541E	hom	Colorectal cancer, non-polyposis
COL3A1	c.4059T>G	p.H1353Q	hom	.
BRCA2	c.1114A>C	p.N372H	hom	.
MYLK	c.1486C>G	p.L496V	hom	.
PCSK9	c.1420G>A	p.V474I	hom	.

INTERPRETATION OF PRIMARY FINDINGS

c.845G>A

The patient is heterozygous for a common missense polymorphism in the HFE gene which occurs at a frequency of 0.013% in the 1000 genomes project. OMIM reports 6 phenotypes associated with variants in the HFE gene including Hemochromatosis, [Transferrin serum level QTL2], susceptibility to

[illegible]
Alzheimer disease, Microvascular complications of diabetes 7, susceptibility to Porphyria cutanea tarda, susceptibility to Porphyria variegata. This variant causes a nonsynonymous severe amino acid substitution (p.C282Y) which may alter protein function. ClinVar classifies this variant as pathogenic, other and non-pathogenic and reports an association with Hereditary hemochromatosis, Porphyria cutanea tarda (susceptibility to), Porphyria variegata (susceptibility to), Hemochromatosis (juvenile) (digenic), Alzheimer disease (susceptibility to), Transferrin serum level quantitative trait locus 2, Microvascular complications of diabetes 7, not specified. This variant is predicted to be deleterious by SIFT and deleterious by PolyPhen2.

c.224G>A

The patient is heterozygous for a common missense polymorphism in the CFTR gene which occurs at a frequency of 0.0064% in the 1000 genomes project. OMIM reports 6 phenotypes associated with variants in the CFTR gene including Congenital bilateral absence of vas deferens, Cystic fibrosis, Sweat chloride elevation without CF, modifier of Bronchiectasis with or without elevated sweat chloride 1, Hypertrypsinemia (neonatal), Pancreatitis (idiopathic). This variant causes a nonsynonymous conservative amino acid substitution (p.R75Q) which may alter protein function. ClinVar classifies this variant as probable-non-pathogenic, unknown, non-pathogenic and pathogenic and reports an association with Cystic fibrosis, , not specified, Hereditary pancreatitis;Cystic fibrosis. This variant is predicted to be deleterious by SIFT and deleterious by PolyPhen2.

c.569A>G

The patient is heterozygous for a common missense polymorphism in the GNPTAB gene which occurs at a frequency of 0.0002% in the 1000 genomes project. OMIM reports 2 phenotypes associated with variants in the GNPTAB gene including Mucolipidosis II alpha/beta, Mucolipidosis III alpha/beta. This variant causes a nonsynonymous moderate amino acid substitution (p.D190G) which may alter protein function. ClinVar classifies this variant as pathogenic and reports an association with Mucopolysaccharidosis (MPS-III-A). This variant is predicted to be deleterious by SIFT and benign by PolyPhen2.

c.1601A>G

The patient is heterozygous for a common missense polymorphism in the F5 gene which occurs at a frequency of 0.99% in the 1000 genomes project. OMIM reports 6 phenotypes associated with variants in the F5 gene including Factor V deficiency, Thrombophilia due to activated protein C resistance, Budd-Chiari syndrome, susceptibility to Pregnancy loss recurrent 1, susceptibility to Stroke (ischemic), susceptibility to Thrombophilia due to factor V Leiden. This variant causes a nonsynonymous conservative amino acid substitution (p.Q534R) which may alter protein function. ClinVar classifies this variant as pathogenic, untested and other and reports an association with Thrombophilia due to factor V Leiden, Ischemic stroke (susceptibility to), Budd-Chiari syndrome (susceptibility to), Recurrent abortion. This variant is predicted to be tolerable by SIFT

c.85C>T

The patient is homogeneous for a common missense polymorphism in the DPYD gene which occurs at a frequency of 0.74% in the 1000 genomes project. OMIM reports 2 phenotypes associated with variants in the DPYD gene including 5-fluorouracil toxicity, Dihydropyrimidine dehydrogenase deficiency. This variant causes a nonsynonymous severe amino acid substitution (p.R29C) which may alter protein function. ClinVar classifies this variant as pathogenic and reports an association with Dihydropyrimidine dehydrogenase deficiency;. This variant is predicted to be tolerable by SIFT and deleterious by PolyPhen2.

c.194+2T>C

The patient is heterozygous for a rare mutation in the SPINK1 gene which occurs at a frequency of 0.0008% in the 1000 genomes project. OMIM reports 3 phenotypes associated with variants in the SPINK1 gene including Pancreatitis (hereditary), Tropical calcific pancreatitis, susceptibility to Fibrocalculous pancreatic diabetes. ClinVar classifies this variant as pathogenic and reports an association with Hereditary pancreatitis.

c.86A>T

The patient is heterozygous for a common missense polymorphism in the PRSS1 gene which doesn't occur in the 1000 genomes project. OMIM reports 2 phenotypes associated with variants in the PRSS1 gene including Pancreatitis (hereditary), Trypsinogen deficiency. This variant causes a nonsynonymous moderate amino acid substitution (p.N29I) which may alter protein function. ClinVar classifies this variant as pathogenic and reports an association with Hereditary pancreatitis;Hereditary pancreatitis. This variant is predicted to be deleterious by SIFT and benign by PolyPhen2.

c.461G>A

The patient is heterozygous for a common nonsense polymorphism in the FUT2 gene which occurs at a frequency of 0.32% in the 1000 genomes project. OMIM reports 3 phenotypes associated with variants in the FUT2 gene including [Bombay phenotype], resistance to Norwalk virus infection, Vitamin B12 plasma level QTL1. This variant introduces a premature stop codon which may lead to early termination of protein transcription. ClinVar classifies this variant as non-pathogenic, pathogenic and other and reports an association with SECRETOR/NONSECRETOR POLYMORPHISM, Norwalk virus infection (resistance to), Vitamin b12 plasma level quantitative trait locus 1. This variant is predicted to be tolerable by SIFT

c.1666A>G

The patient is heterozygous for a common missense polymorphism in the CFTR gene which occurs at a frequency of 0.011% in the 1000 genomes project. OMIM reports 6 phenotypes associated with variants in the CFTR gene including Congenital bilateral absence of vas deferens, Cystic fibrosis, Sweat chloride elevation without CF, modifier of Bronchiectasis with or without elevated sweat chloride 1, Hypertrypsinemia (neonatal), Pancreatitis (idiopathic). This variant causes a nonsynonymous conservative amino acid substitution (p.I556V) which may alter protein function. ClinVar classifies this variant as pathogenic and probable-pathogenic and reports an association with Cystic fibrosis, Nonclassic cystic fibrosis. This variant is predicted to be tolerable by SIFT and benign by PolyPhen2.

c.3578G>A

The patient is heterozygous for a common missense polymorphism in the SCN5A gene which occurs at a frequency of 0.012% in the 1000 genomes project. OMIM reports 9 phenotypes associated with variants in the SCN5A gene including Atrial fibrillation (familial) (10), Brugada syndrome 1, Cardiomyopathy (dilated) (1E), Heart block (nonprogressive), Heart block (progressive) (type IA), Long QT syndrome-3, Sick sinus syndrome 1, Ventricular fibrillation (familial) (1), susceptibility to Sudden infant death syndrome. This variant causes a nonsynonymous conservative amino acid substitution (p.R1193Q) which may alter protein function. ClinVar classifies this variant as pathogenic and other and reports an association with Brugada syndrome 1, Long QT syndrome 3 (acquired) (susceptibility to). This variant is predicted to be tolerable by SIFT and possibly deleterious by PolyPhen2.

c.1162G>A

The patient is heterozygous for a common missense polymorphism in the FGFR4 gene which occurs at a frequency of 0.3% in the 1000 genomes project. OMIM reports that variants in the FGFR4 gene are associated with Cancer progression/metastasis. This variant causes a nonsynonymous moderate amino acid substitution (p.G388R) which may alter protein function. ClinVar classifies this variant as pathogenic and reports an association with Cancer progression and tumor cell motility. This variant is predicted to be deleterious by SIFT and deleterious by PolyPhen2.

c.176T>C

The patient is heterozygous for a common missense polymorphism in the ITGB3 gene which occurs at a frequency of 0.089% in the 1000 genomes project. OMIM reports 5 phenotypes associated with variants in the ITGB3 gene including Bleeding disorder (platelet-type) (16) (autosomal dominant), Glanzmann thrombasthenia, Purpura (posttransfusion), Thrombocytopenia (neonatal alloimmune), susceptibility to Myocardial infarction. This variant causes a nonsynonymous

moderate amino acid substitution (p.L59P) which may alter protein function. ClinVar classifies this variant as non-pathogenic, pathogenic and other and reports an association with PL(A1)/(A2) ALLOANTIGEN POLYMORPHISM, Thrombocytopenia (neonatal alloimmune), Posttransfusion purpura, Myocardial infarction, Fracture (hip) (susceptibility to). This variant is predicted to be tolerable by SIFT and benign by PolyPhen2.

c.539C>T

The patient is heterozygous for a common missense polymorphism in the TIRAP gene which occurs at a frequency of 0.086% in the 1000 genomes project. OMIM reports 4 phenotypes associated with variants in the TIRAP gene including protection against Bacteremia, protection against Malaria, protection against Pneumococcal disease (invasive), protection against Tuberculosis. This variant causes a nonsynonymous moderate amino acid substitution (p.S180L) which may alter protein function. ClinVar classifies this variant as other and pathogenic and reports an association with Invasive pneumococcal disease (protection against), Malaria (resistance to), Mycobacterium tuberculosis (protection against), BACTEREMIA (RESISTANCE TO). This variant is predicted to be possibly deleterious by PolyPhen2.

c.185T>C

The patient is homogeneous for a common missense polymorphism in the HEXB gene which occurs at a frequency of 0.98% in the 1000 genomes project. OMIM reports that variants in the HEXB gene are associated with Sandhoff disease (infantile) (juvenile) (and adult forms). This variant causes a nonsynonymous moderate amino acid substitution (p.L62S) which may alter protein function. ClinVar classifies this variant as pathogenic and reports an association with Sandhoff disease (infantile type). This variant is predicted to be tolerable by SIFT

c.209G>A

The patient is homogeneous for a common missense polymorphism in the BBS2 gene which occurs at a frequency of 1.% in the 1000 genomes project. OMIM reports that variants in the BBS2 gene are associated with Bardet-Biedl syndrome 2. This variant causes a nonsynonymous conservative amino acid substitution (p.S70N) which may alter protein function. ClinVar classifies this variant as pathogenic and reports an association with Bardet-Biedl syndrome 2. This variant is predicted to be tolerable by SIFT

c.2621T>G

The patient is homogeneous for a common missense polymorphism in the ITGA2B gene which occurs at a frequency of 0.4% in the 1000 genomes project. OMIM reports 3 phenotypes associated with variants in the ITGA2B gene including Bleeding disorder (platelet-type) (16) (autosomal dominant), Glanzmann thrombasthenia, Thrombocytopenia (neonatal alloimmune) (BAK antigen related). This variant causes a nonsynonymous moderate amino acid substitution (p.I874S) which may alter protein function. ClinVar classifies this variant as pathogenic and reports an association with Bak platelet-specific antigen. This variant is predicted to be tolerable by SIFT and benign by PolyPhen2.

c.1462T>C

The patient is heterozygous for a common missense polymorphism in the IL4R gene which occurs at a frequency of 0.2% in the 1000 genomes project. OMIM reports 2 phenotypes associated with variants in the IL4R gene including slow progression to AIDS, susceptibility to Atopy. This variant causes a nonsynonymous moderate amino acid substitution (p.S488P) which may alter protein function. ClinVar classifies this variant as pathogenic and other and reports an association with Atopy (resistance to), Asthma (susceptibility to). This variant is predicted to be tolerable by SIFT and possibly deleterious by PolyPhen2.

c.331A>G

The patient is heterozygous for a common missense polymorphism in the SLC24A5 gene which occurs at a frequency of 0.56% in the 1000 genomes project. OMIM reports 2 phenotypes associated with variants in the SLC24A5 gene including Albinism (oculocutaneous) (type VI), [illegible]

[Skin/hair/eye pigmentation 4 (fair)/dark skin]. This variant causes a nonsynonymous moderate amino acid substitution (p.T111A) which may alter protein function. ClinVar classifies this variant as pathogenic and reports an association with Skin/hair/eye pigmentation (variation in) (4). This variant is predicted to be tolerable by SIFT and benign by PolyPhen2.

c.97A>G

The patient is homogeneous for a common missense polymorphism in the HPD gene which occurs at a frequency of 0.88% in the 1000 genomes project. OMIM reports 2 phenotypes associated with variants in the HPD gene including Hawkinsinuria, Tyrosinemia (type III). This variant causes a nonsynonymous moderate amino acid substitution (p.T33A) which may alter protein function. ClinVar classifies this variant as pathogenic and reports an association with 4-Alpha-hydroxyphenylpyruvate hydroxylase deficiency. This variant is predicted to be tolerable by SIFT

c.64C>G

The patient is heterozygous for a common missense polymorphism in the CYP4V2 gene which occurs at a frequency of 0.41% in the 1000 genomes project. OMIM reports that variants in the CYP4V2 gene are associated with Bietti crystalline corneoretinal dystrophy. This variant causes a nonsynonymous conservative amino acid substitution (p.L22V) which may alter protein function. ClinVar classifies this variant as pathogenic, non-pathogenic and probable-non-pathogenic and reports an association with Bietti crystalline corneoretinal dystrophy, not specified. This variant is predicted to be tolerable by SIFT and benign by PolyPhen2.

INTERPRETATION OF SECONDARY FINDINGS

c.1168C>T

The patient is heterozygous for a common missense polymorphism in the MSH2 gene which occurs at a frequency of 0.0028% in the 1000 genomes project. OMIM reports 3 phenotypes associated with variants in the MSH2 gene including Colorectal cancer (hereditary nonpolyposis) (type 1), Mismatch repair cancer syndrome, Muir-Torre syndrome. This variant causes a nonsynonymous conservative amino acid substitution (p.L390F) which may alter protein function. ClinVar classifies this variant as unknown, non-pathogenic and other and reports an association with , Lynch syndrome, not specified, Neoplastic Syndromes (Hereditary). This variant is predicted to be deleterious by SIFT and benign by PolyPhen2.

c.1927G>A

The patient is heterozygous for a common missense polymorphism in the KCNQ1 gene which occurs at a frequency of 0.017% in the 1000 genomes project. OMIM reports 5 phenotypes associated with variants in the KCNQ1 gene including Atrial fibrillation (familial) (3), Jervell and Lange-Nielsen syndrome, Long QT syndrome 1, Short QT syndrome 2, susceptibility to Long QT syndrome 1 (acquired). This variant causes a nonsynonymous moderate amino acid substitution (p.G643S) which may alter protein function. ClinVar classifies this variant as probable-non-pathogenic and non-pathogenic and reports an association with Cardiac arrhythmia. This variant is predicted to be tolerable by SIFT and benign by PolyPhen2.

c.3143A>T

The patient is heterozygous for a rare mutation in the MSH6 gene which doesn't occur in the 1000 genomes project. OMIM reports 3 phenotypes associated with variants in the MSH6 gene including Colorectal cancer (hereditary nonpolyposis) (type 5), Endometrial cancer (familial), Mismatch repair cancer syndrome. This variant causes a nonsynonymous moderate amino acid substitution (p.Q1048L) which may alter protein function. This variant is predicted to be tolerable by SIFT and benign by PolyPhen2.

c.3578G>A

The patient is heterozygous for a common missense polymorphism in the SCN5A gene which [illegible]

occurs at a frequency of 0.012% in the 1000 genomes project. OMIM reports 9 phenotypes associated with variants in the SCN5A gene including Atrial fibrillation (familial) (10), Brugada syndrome 1, Cardiomyopathy (dilated) (1E), Heart block (nonprogressive), Heart block (progressive) (type IA), Long QT syndrome-3, Sick sinus syndrome 1, Ventricular fibrillation (familial) (1), susceptibility to Sudden infant death syndrome. This variant causes a nonsynonymous conservative amino acid substitution (p.R1193Q) which may alter protein function. ClinVar classifies this variant as pathogenic and other and reports an association with Brugada syndrome 1, Long QT syndrome 3 (acquired) (susceptibility to). This variant is predicted to be tolerable by SIFT and possibly deleterious by PolyPhen2.

c.371A>G

The patient is homogeneous for a common missense polymorphism in the SDHD gene which occurs at a frequency of 0.95% in the 1000 genomes project. OMIM reports 6 phenotypes associated with variants in the SDHD gene including Carcinoid tumors (intestinal), Cowden syndrome 3, Merkel cell carcinoma (somatic), Paraganglioma and gastric stromal sarcoma, Paragangliomas 1 (with or without deafness), Pheochromocytoma. This variant causes a nonsynonymous conservative amino acid substitution (p.Q124R) which may alter protein function. This variant is predicted to be tolerable by SIFT

c.1415G>A

The patient is homogeneous for a common missense polymorphism in the FBN1 gene which occurs at a frequency of 1.% in the 1000 genomes project. OMIM reports 8 phenotypes associated with variants in the FBN1 gene including Acromicric dysplasia, Aortic aneurysm (ascending) (and dissection), Ectopia lentis (familial), Geleophysic dysplasia 2, MASS syndrome, Marfan syndrome, Stiff skin syndrome, Weill-Marchesani syndrome 2 (dominant). This variant causes a nonsynonymous severe amino acid substitution (p.C472Y) which may alter protein function. This variant is predicted to be tolerable by SIFT and benign by PolyPhen2.

c.2326A>G

The patient is heterozygous for a common missense polymorphism in the DSC2 gene which occurs at a frequency of 0.2% in the 1000 genomes project. OMIM reports 2 phenotypes associated with variants in the DSC2 gene including Arrhythmogenic right ventricular dysplasia 11 with mild palmoplantar keratoderma and woolly hair, Arrhythmogenic right ventricular dysplasia 11. This variant causes a nonsynonymous conservative amino acid substitution (p.I776V) which may alter protein function. ClinVar classifies this variant as non-pathogenic and reports an association with not specified. This variant is predicted to be tolerable by SIFT and benign by PolyPhen2.

c.1853C>T

The patient is heterozygous for a common missense polymorphism in the APOB gene which occurs at a frequency of 0.48% in the 1000 genomes project. OMIM reports 2 phenotypes associated with variants in the APOB gene including Hypercholesterolemia (due to ligand-defective apo B), Hypobetalipoproteinemia. This variant causes a nonsynonymous moderate amino acid substitution (p.A618V) which may alter protein function. ClinVar classifies this variant as probable-non-pathogenic and reports an association with not specified. This variant is predicted to be tolerable by SIFT and deleterious by PolyPhen2.

c.215C>G

The patient is heterozygous for a common missense polymorphism in the TP53 gene which occurs at a frequency of 0.54% in the 1000 genomes project. OMIM reports 11 phenotypes associated with variants in the TP53 gene including Adrenal cortical carcinoma, Breast cancer, Choroid plexus papilloma, Colorectal cancer, Hepatocellular carcinoma, Li-Fraumeni syndrome, Nasopharyngeal carcinoma, Osteosarcoma, Pancreatic cancer, Basal cell carcinoma 7, Glioma susceptibility 1. This variant causes a nonsynonymous moderate amino acid substitution (p.P72R) which may alter protein function. ClinVar classifies this variant as non-pathogenic and reports an association with CODON 72 POLYMORPHISM ( )(rs1042522), , not specified, Neoplastic Syndromes (Hereditary). This variant is predicted to be tolerable by SIFT and possibly deleterious by PolyPhen2.

c.7397T>C

The patient is homogeneous for a common missense polymorphism in the BRCA2 gene which occurs at a frequency of 0.98% in the 1000 genomes project. OMIM reports 9 phenotypes associated with variants in the BRCA2 gene including Fanconi anemia (complementation group D1), Pancreatic cancer, Prostate cancer, Wilms tumor, susceptibility to Breast cancer (male), Breast-ovarian cancer (familial) (2), Glioblastoma 3, Medulloblastoma, Pre-B-cell acute lymphoblastic leukemia. This variant causes a nonsynonymous moderate amino acid substitution (p.V2466A) which may alter protein function. ClinVar classifies this variant as untested, unknown and non-pathogenic and reports an association with Familial cancer of breast, Breast-ovarian cancer (familial 2), not specified. This variant is predicted to be tolerable by SIFT

c.1014G>C

The patient is heterozygous for a common missense polymorphism in the MUTYH gene which occurs at a frequency of 0.31% in the 1000 genomes project. OMIM reports 3 phenotypes associated with variants in the MUTYH gene including Adenomas (multiple colorectal), Colorectal adenomatous polyposis (autosomal recessive) (with pilomatricomas), Gastric cancer (somatic). This variant causes a nonsynonymous conservative amino acid substitution (p.Q338H) which may alter protein function. ClinVar classifies this variant as non-pathogenic and reports an association with , not specified, Neoplastic Syndromes (Hereditary). This variant is predicted to be tolerable by SIFT and possibly deleterious by PolyPhen2.

c.1636A>G

The patient is homogeneous for a common missense polymorphism in the MEN1 gene which occurs at a frequency of 0.83% in the 1000 genomes project. OMIM reports 6 phenotypes associated with variants in the MEN1 gene including Adrenal adenoma (somatic), Angiofibroma (somatic), Carcinoid tumor of lung, Lipoma (somatic), Multiple endocrine neoplasia 1, Parathyroid adenoma (somatic). This variant causes a nonsynonymous moderate amino acid substitution (p.T546A) which may alter protein function. ClinVar classifies this variant as non-pathogenic and reports an association with , not specified. This variant is predicted to be tolerable by SIFT and benign by PolyPhen2.

c.2612C>T

The patient is heterozygous for a common missense polymorphism in the BRCA1 gene which occurs at a frequency of 0.54% in the 1000 genomes project. OMIM reports 2 phenotypes associated with variants in the BRCA1 gene including Breast-ovarian cancer (familial) (1), susceptibility to Pancreatic cancer 4. This variant causes a nonsynonymous moderate amino acid substitution (p.P871L) which may alter protein function. This variant is predicted to be tolerable by SIFT and benign by PolyPhen2.

c.2318G>A

The patient is homogeneous for a common missense polymorphism in the DSG2 gene which occurs at a frequency of 0.24% in the 1000 genomes project. OMIM reports 2 phenotypes associated with variants in the DSG2 gene including Arrhythmogenic right ventricular dysplasia 10, Cardiomyopathy (dilated) (1BB). This variant causes a nonsynonymous conservative amino acid substitution (p.R773K) which may alter protein function. ClinVar classifies this variant as non-pathogenic and reports an association with not specified. This variant is predicted to be tolerable by SIFT and benign by PolyPhen2.

c.5465T>A

The patient is homogeneous for a common missense polymorphism in the APC gene which occurs at a frequency of 0.87% in the 1000 genomes project. OMIM reports 8 phenotypes associated with variants in the APC gene including Adenoma (periampullary) (somatic), Adenomatous polyposis coli, Brain tumor-polyposis syndrome 2, Colorectal cancer (somatic), Desmoid disease (hereditary), Gardner syndrome, Gastric cancer (somatic), Hepatoblastoma (somatic). This variant causes a nonsynonymous severe amino acid substitution (p.V1822D) which may alter protein function. ClinVar classifies this variant as non-pathogenic and other and reports an association with Adenomatous

classifies this variant as non-pathogenic and other and reports an association with Adenomatous polyposis coli, , not specified, Familial colorectal cancer, Neoplastic Syndromes (Hereditary). This variant is predicted to be benign by PolyPhen2.

c.1621A>G

The patient is homogeneous for a common missense polymorphism in the PMS2 gene which occurs at a frequency of 0.88% in the 1000 genomes project. OMIM reports 2 phenotypes associated with variants in the PMS2 gene including Colorectal cancer (hereditary nonpolyposis) (type 4), Mismatch repair cancer syndrome. This variant causes a nonsynonymous moderate amino acid substitution (p.K541E) which may alter protein function. ClinVar classifies this variant as non-pathogenic and reports an association with Lynch syndrome. This variant is predicted to be tolerable by SIFT and benign by PolyPhen2.

c.4059T>G

The patient is homogeneous for a common missense polymorphism in the COL3A1 gene which occurs at a frequency of 1.% in the 1000 genomes project. OMIM reports 2 phenotypes associated with variants in the COL3A1 gene including Ehlers-Danlos syndrome (type III), Ehlers-Danlos syndrome (type IV). This variant causes a nonsynonymous conservative amino acid substitution (p.H1353Q) which may alter protein function. This variant is predicted to be tolerable by SIFT and benign by PolyPhen2.

c.1114A>C

The patient is homogeneous for a common missense polymorphism in the BRCA2 gene which occurs at a frequency of 0.25% in the 1000 genomes project. OMIM reports 9 phenotypes associated with variants in the BRCA2 gene including Fanconi anemia (complementation group D1), Pancreatic cancer, Prostate cancer, Wilms tumor, susceptibility to Breast cancer (male), Breast-ovarian cancer (familial) (2), Glioblastoma 3, Medulloblastoma, Pre-B-cell acute lymphoblastic leukemia. This variant causes a nonsynonymous moderate amino acid substitution (p.N372H) which may alter protein function. ClinVar classifies this variant as other, non-pathogenic and untested and reports an association with Breast-ovarian cancer (familial 2), , not specified, Neoplastic Syndromes (Hereditary). This variant is predicted to be tolerable by SIFT

c.1486C>G

The patient is homogeneous for a common missense polymorphism in the MYLK gene which occurs at a frequency of 1.% in the 1000 genomes project. OMIM reports that variants in the MYLK gene are associated with Aortic aneurysm (familial thoracic 7). This variant causes a nonsynonymous conservative amino acid substitution (p.L496V) which may alter protein function. This variant is predicted to be tolerable by SIFT and benign by PolyPhen2.

c.1420G>A

The patient is homogeneous for a common missense polymorphism in the PCSK9 gene which occurs at a frequency of 0.87% in the 1000 genomes project. OMIM reports 2 phenotypes associated with variants in the PCSK9 gene including Hypercholesterolemia (familial) (3), Low density lipoprotein cholesterol level QTL 1. This variant causes a nonsynonymous conservative amino acid substitution (p.V474I) which may alter protein function. This variant is predicted to be tolerable by SIFT and benign by PolyPhen2.

RECOMMENDATIONS

Genetic counseling of the family is recommended to discuss the implications of this report. [Additional recommendations.]

METHODS AND LIMITATIONS

This report is a sample to be used solely for the purpose of evaluating functionality of the Tute Platform and should not be used in any clinical application.

Sequencing and Variant Detection

Whole exome sequencing was performed by solution-based hybridization which enriches for coding exons targeting DNA with specific baits. Sequencing was performed using the Illumina HiSeq 2500 to a minimum average exome depth of 90X. Sequencing reads were aligned to the reference genome (hg19) by BWA-MEM and variants were called using FreeBayes.

Variant Analysis and Report Generation

Reported variants were analyzed using the Tute platform to identify those variants which are reported by medical literature to be pathogenic. All non-synonymous variants of allele frequency <1% reported by the 1000 genomes project and annotated as 'Pathogenic' or 'Probably Pathogenic' by ClinVar are reported in the table 'Primary/Diagnostic Sequence Variant(s) Detected'. Careful correlation of these variants with the clinical indications for the proband should be conducted by an experience medical geneticist to verify that reported findings are consistent with reported clinical phenotypes. Additionally, variants are included based on ACMG guidelines for the reporting of incidental findings in the table 'Secondary/Incidental Sequence Variant(s) Detected'.

Validation of reported findings

Variants of interest should be confirmed by Sanger sequencing. The DNA immediately flanking each of these variants should be amplified genomic DNA followed by sequencing in both directions using fluorescent dideoxy sequencing.

Limitations

Absence of a primary diagnostic finding identified by this test does not exclude the possibility of a genetic basis for the clinical condition for this proband. Certain genetic abnormalities are not detectable by the technologies used to generate this report. Specifically, detection of abnormal variants depends on the presence of these sequence variants in the sequencing data. This exome sequence test is designed to evaluate single nucleotide variants within the human exome; however, this technology is only able to sequence 90-95% of the human reference to the requisite 10-fold coverage needed for reliable detection of heterozygous variants.

Additionally, certain types of genetic abnormalities are difficult to identify in sequencing data and have not been validated for clinical use including insertions, deletions, copy number alterations, long repetitive sequences, triplet repeat expansions, chromosomal rearrangements, polyploidy, repetitive regions including mono-, di- and tri-nucleotide repeats, GX rich regions, intronic variants outside the splice-site and epigenetic effects.

Variants believed to be benign based on medical literature, or with population frequencies greater than or equal to 1%, or resulting in synonymous amino acid changes, or occurring in 5' or 3' untranslated regions are generally not reported.

This report is a sample to be used solely for the purpose of evaluating functionality of the Tute Platform and should not be used in any clinical application.

Sources

REPORT SIGNATURES

Online Mendelian Inheritance in Man, OMIM (TM). Retrieved 0/26/2015 from http://www.ncbi.nlm.nih.gov/omim/
ClinVar. Retrieved 0/26/2015 from http://www.ncbi.nlm.nih.gov/clinvar/

NextStep Dx PLUS report for K10031 10235 annotation

ORDERING PROVIDER	SPECIMEN	PATIENT
,	Collection Date:	Name:
	Received Date:	Date of Birth:
	Report Date:	Patient ID:
		Gender:

Clinical Indication:

[Detailed clinical description.]

TEST RESULTS SUMMARY

Primary Result(s): [Findings related to clinical indication.]

Incidental/Secondary Result(s): [Incidental findings.]

PRIMARY/DIAGNOSTIC SEQUENCE VARIANT(S) DETECTED

Gene	Nucleotide Change	Protein Change	Zygosity	Tute Dx
HFE	c.845G>A	p.C282Y	het	Hereditary hemochromatosis|Hemochromatosis, juvenile, digenic
LYST	c.2413delG	p.E805fs	het	Chédiak-Higashi syndrome
BRIP1	c.139C>G	p.P47A	het	Breast cancer, early-onset
F5	c.1601A>G	p.Q534R	het	Thrombophilia due to factor V Leiden
TGM1	c.1552G>A	p.V518M	het	Autosomal recessive congenital ichthyosis 1
DPYD	c.85C>T	p.R29C	hom	Dihydropyrimidine dehydrogenase deficiency;not provided
PRSS1	c.86A>T	p.N29I	het	Hereditary pancreatitis;Hereditary pancreatitis
FUT2	c.461G>A	p.W154X	het	Norwalk virus infection, resistance to
SLC6A20	c.596C>T	p.T199M	het	Hyperglycinuria|Iminoglycinuria, digenic
PRSS1	c.47C>T	p.A16V	het	Hereditary pancreatitis
PRSS1	c.161A>G	p.N54S	het	Hereditary pancreatitis
ELAC2	c.1621G>A	p.A541T	het	Prostate cancer, hereditary, 2
OCA2	c.913C>T	p.R305W	het	Skin/hair/eye pigmentation, variation in, 1
PRF1	c.755A>G	p.N252S	het	Hemophagocytic lymphohistiocytosis, familial, 2|Malignant lymphoma, non-Hodgkin
CYP2B6	c.785A>G	p.K262R	het	Efavirenz, poor metabolism of
ITGB3	c.176T>C	p.L59P	hom	Thrombocytopenia, neonatal alloimmune|Posttransfusion purpura
CST3	c.73G>A	p.A25T	het	Age-related macular degeneration 11
HEXB	c.185T>C	p.L62S	hom	Sandhoff disease, infantile type
BBS2	c.209G>A	p.S70N	hom	Bardet-Biedl syndrome 2
BTD	c.1177C>T	p.P393S	het	Biotinidase deficiency

SECONDARY/INCIDENTAL SEQUENCE VARIANT(S) DETECTED

The American College of Medical Genetics and Genomics (ACMG) recently published a policy statement on clinical sequencing that emphasizes the possibility of recognizing and reporting incidental or secondary findings of medical value when conducting exome and genome sequencing. The ACMG recommends that laboratories performing clinical sequencing seek and report mutations of specified classes or types in the genes listed within the guidelines. This section of the report describes variants which fit the criteria outlined by ACMG for the reporting of incidental or secondary findings.

Gene	Nucleotide Change	Protein Change	Zygosity	Tute Dx
RYR1	c.7336G>T	p.G2446C	het	.
MUTYH	c.1276C>T	p.R426C	het	Adenomatous polyposis coli
PMS2	c.59G>A	p.R20Q	het	Colorectal cancer, non-polyposis
MUTYH	c.64G>A	p.V22M	het	.
SDHD	c.371A>G	p.Q124R	hom	.
FBN1	c.1415G>A	p.C472Y	hom	.
TP53	c.215C>G	p.P72R	het	.
BRCA2	c.7397T>C	p.V2466A	hom	Breast cancer
MUTYH	c.1014G>C	p.Q338H	het	.
MEN1	c.1636A>G	p.T546A	hom	.
DSG2	c.2318G>A	p.R773K	het	.
APC	c.5465T>A	p.V1822D	het	.
PMS2	c.1621A>G	p.K541E	hom	Colorectal cancer, non-polyposis
COL3A1	c.4059T>G	p.H1353Q	hom	.
BRCA2	c.1114A>C	p.N372H	het	.
MYLK	c.1486C>G	p.L496V	hom	.
PCSK9	c.1420G>A	p.V474I	hom	.
APOB	c.13013G>A	p.S4338N	hom	.
PCSK9	c.2009G>A	p.G670E	hom	.
APOB	c.6937A>G	p.I2313V	hom	.

INTERPRETATION OF PRIMARY FINDINGS

c.845G>A

The patient is heterozygous for a common missense polymorphism in the HFE gene which occurs at a frequency of 0.013% in the 1000 genomes project. OMIM reports 6 phenotypes associated with variants in the HFE gene including Hemochromatosis, [Transferrin serum level QTL2], susceptibility to Alzheimer disease, Microvascular complications of diabetes 7, susceptibility to Porphyria cutanea tarda, susceptibility to Porphyria variegata. This variant causes a nonsynonymous severe amino acid substitution (p.C282Y) which may alter protein function. ClinVar classifies this variant as

acid substitution (p.C282[illegible]) which may alter protein function. ClinVar classifies this variant as pathogenic, other and non-pathogenic and reports an association with Hereditary hemochromatosis, Porphyria cutanea tarda (susceptibility to), Porphyria variegata (susceptibility to), Hemochromatosis (juvenile) (digenic), Alzheimer disease (susceptibility to), Transferrin serum level quantitative trait locus 2, Microvascular complications of diabetes 7, not specified. This variant is predicted to be deleterious by SIFT and deleterious by PolyPhen2.

c.2413delG

The patient is heterozygous for a rare mutation in the LYST gene which doesn't occur in the 1000 genomes project. OMIM reports that variants in the LYST gene are associated with Chediak-Higashi syndrome. This variant causes a frameshift which may significantly alter protein expression or function. ClinVar classifies this variant as pathogenic and reports an association with Chédiak-Higashi syndrome.

c.139C>G

The patient is heterozygous for a rare missense mutation in the BRIP1 gene which doesn't occur in the 1000 genomes project. OMIM reports 2 phenotypes associated with variants in the BRIP1 gene including Breast cancer (early-onset), Fanconi anemia (complementation group J). This variant causes a nonsynonymous conservative amino acid substitution (p.P47A) which may alter protein function. ClinVar classifies this variant as pathogenic and other and reports an association with Breast cancer (early-onset), Neoplastic Syndromes (Hereditary). This variant is predicted to be deleterious by PolyPhen2.

c.1601A>G

The patient is heterozygous for a common missense polymorphism in the F5 gene which occurs at a frequency of 0.99% in the 1000 genomes project. OMIM reports 6 phenotypes associated with variants in the F5 gene including Factor V deficiency, Thrombophilia due to activated protein C resistance, Budd-Chiari syndrome, susceptibility to Pregnancy loss recurrent 1, susceptibility to Stroke (ischemic), susceptibility to Thrombophilia due to factor V Leiden. This variant causes a nonsynonymous conservative amino acid substitution (p.Q534R) which may alter protein function. ClinVar classifies this variant as pathogenic, untested and other and reports an association with Thrombophilia due to factor V Leiden, Ischemic stroke (susceptibility to), Budd-Chiari syndrome (susceptibility to), Recurrent abortion. This variant is predicted to be tolerable by SIFT

c.1552G>A

The patient is heterozygous for a common missense polymorphism in the TGM1 gene which occurs at a frequency of 0.0036% in the 1000 genomes project. OMIM reports that variants in the TGM1 gene are associated with Ichthyosis (congenital) (autosomal recessive 1). This variant causes a nonsynonymous conservative amino acid substitution (p.V518M) which may alter protein function. ClinVar classifies this variant as pathogenic and reports an association with Autosomal recessive congenital ichthyosis 1. This variant is predicted to be tolerable by SIFT and deleterious by PolyPhen2.

c.85C>T

The patient is homogeneous for a common missense polymorphism in the DPYD gene which occurs at a frequency of 0.74% in the 1000 genomes project. OMIM reports 2 phenotypes associated with variants in the DPYD gene including 5-fluorouracil toxicity, Dihydropyrimidine dehydrogenase deficiency. This variant causes a nonsynonymous severe amino acid substitution (p.R29C) which may alter protein function. ClinVar classifies this variant as pathogenic and reports an association with Dihydropyrimidine dehydrogenase deficiency;. This variant is predicted to be tolerable by SIFT and deleterious by PolyPhen2.

c.86A>T

The patient is heterozygous for a common missense polymorphism in the PRSS1 gene which doesn't occur in the 1000 genomes project. OMIM reports 2 phenotypes associated with variants in the PRSS1 gene including Pancreatitis (hereditary), Trypsinogen deficiency. This variant causes a nonsynonymous moderate amino acid substitution (p.N29I) which may alter protein function. ClinVar

[illegible]
classifies this variant as pathogenic and reports an association with Hereditary pancreatitis;Hereditary pancreatitis. This variant is predicted to be deleterious by SIFT and benign by PolyPhen2.

c.461G>A

The patient is heterozygous for a common nonsense polymorphism in the FUT2 gene which occurs at a frequency of 0.32% in the 1000 genomes project. OMIM reports 3 phenotypes associated with variants in the FUT2 gene including [Bombay phenotype], resistance to Norwalk virus infection, Vitamin B12 plasma level QTL1. This variant introduces a premature stop codon which may lead to early termination of protein transcription. ClinVar classifies this variant as non-pathogenic, pathogenic and other and reports an association with SECRETOR/NONSECRETOR POLYMORPHISM, Norwalk virus infection (resistance to), Vitamin b12 plasma level quantitative trait locus 1. This variant is predicted to be tolerable by SIFT

c.596C>T

The patient is heterozygous for a common missense polymorphism in the SLC6A20 gene which occurs at a frequency of 0.033% in the 1000 genomes project. OMIM reports 2 phenotypes associated with variants in the SLC6A20 gene including Hyperglycinuria, Iminoglycinuria (digenic). This variant causes a nonsynonymous moderate amino acid substitution (p.T199M) which may alter protein function. ClinVar classifies this variant as pathogenic and reports an association with Hyperglycinuria, Iminoglycinuria (digenic). This variant is predicted to be deleterious by SIFT and deleterious by PolyPhen2.

c.47C>T

The patient is heterozygous for a common missense polymorphism in the PRSS1 gene which doesn't occur in the 1000 genomes project. OMIM reports 2 phenotypes associated with variants in the PRSS1 gene including Pancreatitis (hereditary), Trypsinogen deficiency. This variant causes a nonsynonymous moderate amino acid substitution (p.A16V) which may alter protein function. ClinVar classifies this variant as pathogenic and reports an association with Hereditary pancreatitis. This variant is predicted to be tolerable by SIFT and benign by PolyPhen2.

c.161A>G

The patient is heterozygous for a common missense polymorphism in the PRSS1 gene which doesn't occur in the 1000 genomes project. OMIM reports 2 phenotypes associated with variants in the PRSS1 gene including Pancreatitis (hereditary), Trypsinogen deficiency. This variant causes a nonsynonymous conservative amino acid substitution (p.N54S) which may alter protein function. ClinVar classifies this variant as pathogenic and reports an association with Hereditary pancreatitis. This variant is predicted to be tolerable by SIFT and benign by PolyPhen2.

c.1621G>A

The patient is heterozygous for a common missense polymorphism in the ELAC2 gene which occurs at a frequency of 0.023% in the 1000 genomes project. OMIM reports 2 phenotypes associated with variants in the ELAC2 gene including Combined oxidative phosphorylation deficiency 17, susceptibility to Prostate cancer (hereditary) (2). This variant causes a nonsynonymous moderate amino acid substitution (p.A541T) which may alter protein function. ClinVar classifies this variant as pathogenic and reports an association with Prostate cancer (hereditary) (2). This variant is predicted to be tolerable by SIFT and possibly deleterious by PolyPhen2.

c.913C>T

The patient is heterozygous for a common missense polymorphism in the OCA2 gene which occurs at a frequency of 0.083% in the 1000 genomes project. OMIM reports 4 phenotypes associated with variants in the OCA2 gene including Albinism (brown oculocutaneous), Albinism (oculocutaneous) (type II), [Skin/hair/eye pigmentation 1 (blond)/brown hair], [Skin/hair/eye pigmentation 1 (blue)/nonblue eyes]. This variant causes a nonsynonymous moderate amino acid substitution (p.R305W) which may alter protein function. ClinVar classifies this variant as pathogenic and reports an association with Skin/hair/eye pigmentation (variation in) (1). This variant is predicted to be
[illegible]

deleterious by SIFT and possibly deleterious by PolyPhen2.

c.755A>G

The patient is heterozygous for a common missense polymorphism in the PRF1 gene which occurs at a frequency of 0.0076% in the 1000 genomes project. OMIM reports 2 phenotypes associated with variants in the PRF1 gene including Hemophagocytic lymphohistiocytosis (familial) (2), Lymphoma (non-Hodgkin). This variant causes a nonsynonymous conservative amino acid substitution (p.N252S) which may alter protein function. ClinVar classifies this variant as pathogenic and reports an association with Hemophagocytic lymphohistiocytosis (familial) (2), Malignant lymphoma (non-Hodgkin). This variant is predicted to be tolerable by SIFT and benign by PolyPhen2.

c.785A>G

The patient is heterozygous for a common missense polymorphism in the CYP2B6 gene which doesn't occur in the 1000 genomes project. OMIM reports 2 phenotypes associated with variants in the CYP2B6 gene including poor metabolism of Efavirenz, susceptibility to Efavirenz central nervous system toxicity. This variant causes a nonsynonymous conservative amino acid substitution (p.K262R) which may alter protein function. ClinVar classifies this variant as pathogenic and reports an association with Efavirenz (poor metabolism of). This variant is predicted to be tolerable by SIFT and benign by PolyPhen2.

c.176T>C

The patient is homogeneous for a common missense polymorphism in the ITGB3 gene which occurs at a frequency of 0.089% in the 1000 genomes project. OMIM reports 5 phenotypes associated with variants in the ITGB3 gene including Bleeding disorder (platelet-type) (16) (autosomal dominant), Glanzmann thrombasthenia, Purpura (posttransfusion), Thrombocytopenia (neonatal alloimmune), susceptibility to Myocardial infarction. This variant causes a nonsynonymous moderate amino acid substitution (p.L59P) which may alter protein function. ClinVar classifies this variant as non-pathogenic, pathogenic and other and reports an association with PL(A1)/(A2) ALLOANTIGEN POLYMORPHISM, Thrombocytopenia (neonatal alloimmune), Posttransfusion purpura, Myocardial infarction, Fracture (hip) (susceptibility to). This variant is predicted to be tolerable by SIFT and benign by PolyPhen2.

c.73G>A

The patient is heterozygous for a common missense polymorphism in the CST3 gene which occurs at a frequency of 0.21% in the 1000 genomes project. OMIM reports 2 phenotypes associated with variants in the CST3 gene including Cerebral amyloid angiopathy, Macular degeneration (age-related) (11). This variant causes a nonsynonymous moderate amino acid substitution (p.A25T) which may alter protein function. ClinVar classifies this variant as pathogenic and reports an association with Age-related macular degeneration 11. This variant is predicted to be tolerable by SIFT and benign by PolyPhen2.

c.185T>C

The patient is homogeneous for a common missense polymorphism in the HEXB gene which occurs at a frequency of 0.98% in the 1000 genomes project. OMIM reports that variants in the HEXB gene are associated with Sandhoff disease (infantile) (juvenile) (and adult forms). This variant causes a nonsynonymous moderate amino acid substitution (p.L62S) which may alter protein function. ClinVar classifies this variant as pathogenic and reports an association with Sandhoff disease (infantile type). This variant is predicted to be tolerable by SIFT

c.209G>A

The patient is homogeneous for a common missense polymorphism in the BBS2 gene which occurs at a frequency of 1.% in the 1000 genomes project. OMIM reports that variants in the BBS2 gene are associated with Bardet-Biedl syndrome 2. This variant causes a nonsynonymous conservative amino acid substitution (p.S70N) which may alter protein function. ClinVar classifies this variant as pathogenic and reports an association with Bardet-Biedl syndrome 2. This variant is predicted to be tolerable by SIFT

predicted to be tolerable by SIFT

c.1177C>T

The patient is heterozygous for a common missense polymorphism in the BTD gene which occurs at a frequency of 0.0068% in the 1000 genomes project. OMIM reports that variants in the BTD gene are associated with Biotinidase deficiency. This variant causes a nonsynonymous moderate amino acid substitution (p.P393S) which may alter protein function. ClinVar classifies this variant as non-pathogenic and pathogenic and reports an association with Biotinidase deficiency, Biotinidase deficiency. ARUP classifies this variant as Pathogenic. This variant is predicted to be tolerable by SIFT and benign by PolyPhen2.

INTERPRETATION OF SECONDARY FINDINGS

---

c.7336G>T

The patient is heterozygous for a rare mutation in the RYR1 gene which doesn't occur in the 1000 genomes project. OMIM reports 5 phenotypes associated with variants in the RYR1 gene including Central core disease, King-Denborough syndrome, Minicore myopathy with external ophthalmoplegia, Neuromuscular disease (congenital) (with uniform type 1 fiber), Malignant hyperthermia susceptibility 1. This variant causes a nonsynonymous severe amino acid substitution (p.G2446C) which may alter protein function. This variant is predicted to be deleterious by SIFT and benign by PolyPhen2.

c.1276C>T

The patient is heterozygous for a rare missense mutation in the MUTYH gene which doesn't occur in the 1000 genomes project. OMIM reports 3 phenotypes associated with variants in the MUTYH gene including Adenomas (multiple colorectal), Colorectal adenomatous polyposis (autosomal recessive) (with pilomatricomas), Gastric cancer (somatic). This variant causes a nonsynonymous severe amino acid substitution (p.R426C) which may alter protein function. ClinVar classifies this variant as unknown and other and reports an association with , Neoplastic Syndromes (Hereditary), MYH-associated polyposis. Invitae classifies this variant as Variant of uncertain significance. This variant is predicted to be tolerable by SIFT and deleterious by PolyPhen2.

c.59G>A

The patient is heterozygous for a common missense polymorphism in the PMS2 gene which occurs at a frequency of 0.076% in the 1000 genomes project. OMIM reports 2 phenotypes associated with variants in the PMS2 gene including Colorectal cancer (hereditary nonpolyposis) (type 4), Mismatch repair cancer syndrome. This variant causes a nonsynonymous conservative amino acid substitution (p.R20Q) which may alter protein function. ClinVar classifies this variant as non-pathogenic and untested and reports an association with , Lynch syndrome, not specified, Neoplastic Syndromes (Hereditary). This variant is predicted to be tolerable by SIFT and possibly deleterious by PolyPhen2.

c.64G>A

The patient is heterozygous for a common missense polymorphism in the MUTYH gene which occurs at a frequency of 0.02% in the 1000 genomes project. OMIM reports 3 phenotypes associated with variants in the MUTYH gene including Adenomas (multiple colorectal), Colorectal adenomatous polyposis (autosomal recessive) (with pilomatricomas), Gastric cancer (somatic). This variant causes a nonsynonymous conservative amino acid substitution (p.V22M) which may alter protein function. ClinVar classifies this variant as non-pathogenic and reports an association with , not specified, MYH-associated polyposis, Neoplastic Syndromes (Hereditary). Invitae classifies this variant as Benign. This variant is predicted to be deleterious by SIFT and possibly deleterious by PolyPhen2.

c.371A>G

The patient is homogeneous for a common missense polymorphism in the SDHD gene which

The patient is homogeneous for a common missense polymorphism in the SDHD gene which occurs at a frequency of 0.95% in the 1000 genomes project. OMIM reports 6 phenotypes associated with variants in the SDHD gene including Carcinoid tumors (intestinal), Cowden syndrome 3, Merkel cell carcinoma (somatic), Paraganglioma and gastric stromal sarcoma, Paragangliomas 1 (with or without deafness), Pheochromocytoma. This variant causes a nonsynonymous conservative amino acid substitution (p.Q124R) which may alter protein function. This variant is predicted to be tolerable by SIFT

c.1415G>A

The patient is homogeneous for a common missense polymorphism in the FBN1 gene which occurs at a frequency of 1.% in the 1000 genomes project. OMIM reports 8 phenotypes associated with variants in the FBN1 gene including Acromicric dysplasia, Aortic aneurysm (ascending) (and dissection), Ectopia lentis (familial), Geleophysic dysplasia 2, MASS syndrome, Marfan syndrome, Stiff skin syndrome, Weill-Marchesani syndrome 2 (dominant). This variant causes a nonsynonymous severe amino acid substitution (p.C472Y) which may alter protein function. This variant is predicted to be tolerable by SIFT and benign by PolyPhen2.

c.215C>G

The patient is heterozygous for a common missense polymorphism in the TP53 gene which occurs at a frequency of 0.54% in the 1000 genomes project. OMIM reports 11 phenotypes associated with variants in the TP53 gene including Adrenal cortical carcinoma, Breast cancer, Choroid plexus papilloma, Colorectal cancer, Hepatocellular carcinoma, Li-Fraumeni syndrome, Nasopharyngeal carcinoma, Osteosarcoma, Pancreatic cancer, Basal cell carcinoma 7, Glioma susceptibility 1. This variant causes a nonsynonymous moderate amino acid substitution (p.P72R) which may alter protein function. ClinVar classifies this variant as non-pathogenic and reports an association with CODON 72 POLYMORPHISM ( )(rs1042522), , not specified, Neoplastic Syndromes (Hereditary). This variant is predicted to be tolerable by SIFT and possibly deleterious by PolyPhen2.

c.7397T>C

The patient is homogeneous for a common missense polymorphism in the BRCA2 gene which occurs at a frequency of 0.98% in the 1000 genomes project. OMIM reports 9 phenotypes associated with variants in the BRCA2 gene including Fanconi anemia (complementation group D1), Pancreatic cancer, Prostate cancer, Wilms tumor, susceptibility to Breast cancer (male), Breast-ovarian cancer (familial) (2), Glioblastoma 3, Medulloblastoma, Pre-B-cell acute lymphoblastic leukemia. This variant causes a nonsynonymous moderate amino acid substitution (p.V2466A) which may alter protein function. ClinVar classifies this variant as untested, unknown and non-pathogenic and reports an association with Familial cancer of breast, Breast-ovarian cancer (familial 2), not specified. This variant is predicted to be tolerable by SIFT

c.1014G>C

The patient is heterozygous for a common missense polymorphism in the MUTYH gene which occurs at a frequency of 0.31% in the 1000 genomes project. OMIM reports 3 phenotypes associated with variants in the MUTYH gene including Adenomas (multiple colorectal), Colorectal adenomatous polyposis (autosomal recessive) (with pilomatricomas), Gastric cancer (somatic). This variant causes a nonsynonymous conservative amino acid substitution (p.Q338H) which may alter protein function. ClinVar classifies this variant as non-pathogenic and reports an association with , not specified, Neoplastic Syndromes (Hereditary). This variant is predicted to be tolerable by SIFT and possibly deleterious by PolyPhen2.

c.1636A>G

The patient is homogeneous for a common missense polymorphism in the MEN1 gene which occurs at a frequency of 0.83% in the 1000 genomes project. OMIM reports 6 phenotypes associated with variants in the MEN1 gene including Adrenal adenoma (somatic), Angiofibroma (somatic), Carcinoid tumor of lung, Lipoma (somatic), Multiple endocrine neoplasia 1, Parathyroid adenoma (somatic). This variant causes a nonsynonymous moderate amino acid substitution (p.T546A) which may alter protein function. ClinVar classifies this variant as non-pathogenic and reports an association with , not specified. This variant is predicted to be tolerable by SIFT and

reports an association with , not specified. This variant is predicted to be tolerable by SIFT and benign by PolyPhen2.

c.2318G>A

The patient is heterozygous for a common missense polymorphism in the DSG2 gene which occurs at a frequency of 0.24% in the 1000 genomes project. OMIM reports 2 phenotypes associated with variants in the DSG2 gene including Arrhythmogenic right ventricular dysplasia 10, Cardiomyopathy (dilated) (1BB). This variant causes a nonsynonymous conservative amino acid substitution (p.R773K) which may alter protein function. ClinVar classifies this variant as non-pathogenic and reports an association with not specified. This variant is predicted to be tolerable by SIFT and benign by PolyPhen2.

c.5465T>A

The patient is heterozygous for a common missense polymorphism in the APC gene which occurs at a frequency of 0.87% in the 1000 genomes project. OMIM reports 8 phenotypes associated with variants in the APC gene including Adenoma (periampullary) (somatic), Adenomatous polyposis coli, Brain tumor-polyposis syndrome 2, Colorectal cancer (somatic), Desmoid disease (hereditary), Gardner syndrome, Gastric cancer (somatic), Hepatoblastoma (somatic). This variant causes a nonsynonymous severe amino acid substitution (p.V1822D) which may alter protein function. ClinVar classifies this variant as non-pathogenic and other and reports an association with Adenomatous polyposis coli, , not specified, Familial colorectal cancer, Neoplastic Syndromes (Hereditary). This variant is predicted to be benign by PolyPhen2.

c.1621A>G

The patient is homogeneous for a common missense polymorphism in the PMS2 gene which occurs at a frequency of 0.88% in the 1000 genomes project. OMIM reports 2 phenotypes associated with variants in the PMS2 gene including Colorectal cancer (hereditary nonpolyposis) (type 4), Mismatch repair cancer syndrome. This variant causes a nonsynonymous moderate amino acid substitution (p.K541E) which may alter protein function. ClinVar classifies this variant as non-pathogenic and reports an association with Lynch syndrome. This variant is predicted to be tolerable by SIFT and benign by PolyPhen2.

c.4059T>G

The patient is homogeneous for a common missense polymorphism in the COL3A1 gene which occurs at a frequency of 1.% in the 1000 genomes project. OMIM reports 2 phenotypes associated with variants in the COL3A1 gene including Ehlers-Danlos syndrome (type III), Ehlers-Danlos syndrome (type IV). This variant causes a nonsynonymous conservative amino acid substitution (p.H1353Q) which may alter protein function. This variant is predicted to be tolerable by SIFT and benign by PolyPhen2.

c.1114A>C

The patient is heterozygous for a common missense polymorphism in the BRCA2 gene which occurs at a frequency of 0.25% in the 1000 genomes project. OMIM reports 9 phenotypes associated with variants in the BRCA2 gene including Fanconi anemia (complementation group D1), Pancreatic cancer, Prostate cancer, Wilms tumor, susceptibility to Breast cancer (male), Breast-ovarian cancer (familial) (2), Glioblastoma 3, Medulloblastoma, Pre-B-cell acute lymphoblastic leukemia. This variant causes a nonsynonymous moderate amino acid substitution (p.N372H) which may alter protein function. ClinVar classifies this variant as other, non-pathogenic and untested and reports an association with Breast-ovarian cancer (familial 2), , not specified, Neoplastic Syndromes (Hereditary). This variant is predicted to be tolerable by SIFT

c.1486C>G

The patient is homogeneous for a common missense polymorphism in the MYLK gene which occurs at a frequency of 1.% in the 1000 genomes project. OMIM reports that variants in the MYLK gene are associated with Aortic aneurysm (familial thoracic 7). This variant causes a nonsynonymous conservative amino acid substitution (p.L496V) which may alter protein function. This variant is predicted to be tolerable by SIFT and benign by PolyPhen2.

[illegible]

c.1420G>A

The patient is homogeneous for a common missense polymorphism in the PCSK9 gene which occurs at a frequency of 0.87% in the 1000 genomes project. OMIM reports 2 phenotypes associated with variants in the PCSK9 gene including Hypercholesterolemia (familial) (3), Low density lipoprotein cholesterol level QTL 1. This variant causes a nonsynonymous conservative amino acid substitution (p.V474I) which may alter protein function. This variant is predicted to be tolerable by SIFT and benign by PolyPhen2.

c.13013G>A

The patient is homogeneous for a common missense polymorphism in the APOB gene which occurs at a frequency of 0.63% in the 1000 genomes project. OMIM reports 2 phenotypes associated with variants in the APOB gene including Hypercholesterolemia (due to ligand-defective apo B), Hypobetalipoproteinemia. This variant causes a nonsynonymous conservative amino acid substitution (p.S4338N) which may alter protein function. ClinVar classifies this variant as probable-non-pathogenic and reports an association with not specified. This variant is predicted to be tolerable by SIFT and benign by PolyPhen2.

c.2009G>A

The patient is homogeneous for a common missense polymorphism in the PCSK9 gene which occurs at a frequency of 0.9% in the 1000 genomes project. OMIM reports 2 phenotypes associated with variants in the PCSK9 gene including Hypercholesterolemia (familial) (3), Low density lipoprotein cholesterol level QTL 1. This variant causes a nonsynonymous moderate amino acid substitution (p.G670E) which may alter protein function. ClinVar classifies this variant as non-pathogenic and reports an association with Familial hypercholesterolemia. This variant is predicted to be tolerable by SIFT and benign by PolyPhen2.

c.6937A>G

The patient is homogeneous for a common missense polymorphism in the APOB gene which occurs at a frequency of 0.99% in the 1000 genomes project. OMIM reports 2 phenotypes associated with variants in the APOB gene including Hypercholesterolemia (due to ligand-defective apo B), Hypobetalipoproteinemia. This variant causes a nonsynonymous conservative amino acid substitution (p.I2313V) which may alter protein function. This variant is predicted to be tolerable by SIFT and benign by PolyPhen2.

RECOMMENDATIONS

Genetic counseling of the family is recommended to discuss the implications of this report.
[Additional recommendations.]

METHODS AND LIMITATIONS

This report is a sample to be used solely for the purpose of evaluating functionality of the Tute Platform and should not be used in any clinical application.

Sequencing and Variant Detection

Whole exome sequencing was performed by solution-based hybridization which enriches for coding exons targeting DNA with specific baits. Sequencing was performed using the Illumina HiSeq 2500 to a minimum average exome depth of 90X. Sequencing reads were aligned to the reference genome (hg19) by BWA-MEM and variants were called using FreeBayes.

Variant Analysis and Report Generation

Reported variants were analyzed using the Tute platform to identify those variants which are reported by medical literature to be pathogenic. All non-synonymous variants of allele frequency <1% reported by the 1000 genomes project and annotated as 'Pathogenic' or 'Probably Pathogenic' by ClinVar are reported in the table 'Primary/Diagnostic Sequence Variant(s) Detected'. Careful correlation of these variants with the clinical indications for the proband should be conducted by an experience medical geneticist to verify that reported findings are consistent with reported clinical phenotypes. Additionally, variants are included based on ACMG guidelines for the reporting of incidental findings in the table 'Secondary/Incidental Sequence Variant(s) Detected'.

Validation of reported findings

Variants of interest should be confirmed by Sanger sequencing. The DNA immediately flanking each of these variants should be amplified genomic DNA followed by sequencing in both directions using fluorescent dideoxy sequencing.

Limitations

Absence of a primary diagnostic finding identified by this test does not exclude the possibility of a genetic basis for the clinical condition for this proband. Certain genetic abnormalities are not detectable by the technologies used to generate this report. Specifically, detection of abnormal variants depends on the presence of these sequence variants in the sequencing data. This exome sequence test is designed to evaluate single nucleotide variants within the human exome; however, this technology is only able to sequence 90-95% of the human reference to the requisite 10-fold coverage needed for reliable detection of heterozygous variants.

Additionally, certain types of genetic abnormalities are difficult to identify in sequencing data and have not been validated for clinical use including insertions, deletions, copy number alterations, long repetitive sequences, triplet repeat expansions, chromosomal rearrangements, polyploidy, repetitive regions including mono-, di- and tri-nucleotide repeats, GX rich regions, intronic variants outside the splice-site and epigenetic effects.

Variants believed to be benign based on medical literature, or with population frequencies greater than or equal to 1%, or resulting in synonymous amino acid changes, or occurring in 5' or 3' untranslated regions are generally not reported.

This report is a sample to be used solely for the purpose of evaluating functionality of the Tute Platform and should not be used in any clinical application.

Sources

REPORT SIGNATURES

Online Mendelian Inheritance in Man, OMIM (TM). Retrieved 0/26/2015 from http://www.ncbi.nlm.nih.gov/omim/
ClinVar. Retrieved 0/26/2015 from http://www.ncbi.nlm.nih.gov/clinvar/
