# Supplementary material for: Whole genome sequencing of one complex pedigree illustrates challenges with genomic medicine
Source: BMC Med Genomics. 2017 Feb 23;10:10. doi: 10.1186/s12920-017-0246-5 (PMC5322674; doi:10.1186/s12920-017-0246-5)
Supplement: Additional file 3: — Phenomizer Diagnosis Report. This file contains three Phenomizer diagnosis reports of individual K10031-10232, K10031-10133, and K10031-10145. (PDF 429 kb) [file 12920_2017_246_MOESM3_ESM.pdf]

1 Patient data

Name, Firstname: K10031-10232

Date of birth:

Gender: Male ◉ Female ○

2 Query

Reference:
Köhler S, Schulz MH, Krawitz P, Bauer S, Dölken S, Ott CE, Mundlos C, Horn D, Mundlos S, Robinson PN
Clinical Diagnostics in Human Genetics with Semantic Similarity Searches in Ontologies
The American Journal of Human Genetics 85, pp. 457-464, Oktober 2009.

Query Terms:

- Caesarian section (HP:0011410)
- Narrow forehead (HP:0000341)
- Low frustration tolerance (HP:0000744)
- Poor eye contact (HP:0000817)
- Delayed speech and language development (HP:0000750)
- Hyperesthesia (HP:0100963)
- Anxiety (HP:0000739)
- Irritability (HP:0000737)
- Short attention span (HP:0000736)
- Growth hormone deficiency (HP:0000824)
- Almond-shaped palpebral fissure (HP:0007874)
- Obstructive sleep apnea (HP:0002870)
- Excessive daytime sleepiness (HP:0002189)
- Infantile muscular hypotonia (HP:0008947)
- Cryptorchidism (HP:0000028)
- Lack of peer relationships (HP:0002332)
- Lack of insight (HP:0000757)
- Hair-pulling (HP:0012167)
- Maternal diabetes (HP:0009800)
- Skin-picking (HP:0012166)
- Poor fine motor coordination (HP:0007010)
- Hypersomnia (HP:0100786)
- Impaired social reciprocity (HP:0012760)
- Depression (HP:0000716)
- Aggressive behavior (HP:0000718)
- Somnolence (HP:0001262)
- Polyphagia (HP:0002591)
- Scoliosis (HP:0002650)
- Dysarthria (HP:0001260)
- Pain insensitivity (HP:0007021)
- Nail-biting (HP:0012170)
- Intellectual disability, mild (HP:0001256)
- Impaired ability to form peer relationships (HP:0000728)
- Downslanted palpebral fissures (HP:0000494)
- Inflexible adherence to routines or rituals (HP:0000732)

Inheritance:	none
Similarity measure:	Resnik (not symmetric)

3 Results

p-Value	*Score*	*Disease entry*	*Known Genes*
0.0001	3.2383	#176270 PRADER-WILLI SYNDROME; PWS;;PRADER-LABHART-WILLI SYNDROMEPRADER-WILLI SYNDROME CHROMOSOME REGION, INCLUDED; PWCR, INCLUDED;;PRADER-WILLI-LIKE SYNDROME ASSOCIATED WITH CHROMOSOME 6, INCLUDED (OMIM:176270)	SNRPN, NDN
0.0001	2.9703	#613406 CHROMOSOME 15Q24 DELETION SYNDROMECHROMOSOME 15Q24 DUPLICATION SYNDROME, INCLUDED (OMIM:613406)	
0.0001	2.7799	#606232 CHROMOSOME 22Q13.3 DELETION SYNDROME;;TELOMERIC 22Q13 MONOSOMY SYNDROME;;PHELAN-MCDERMID SYNDROME (OMIM:606232)	SHANK3
0.0001	2.7473	#300260 LUBS X-LINKED MENTAL RETARDATION SYNDROME; MRXSL;;MECP2 DUPLICATION SYNDROME;;MENTAL RETARDATION, X-LINKED, SYNDROMIC, LUBS TYPE;;MENTAL RETARDATION, X-LINKED, WITH RECURRENT RESPIRATORY INFECTIONS (OMIM:300260)	MECP2
0.0001	2.6822	#610253 KLEEFSTRA SYNDROME;;CHROMOSOME 9Q34.3 DELETION SYNDROME;;9Q- SYNDROME;;9Q SUBTELOMERIC DELETION SYNDROME (OMIM:610253)	EHMT1
0.0001	2.6294	#610883 POTOCKI-LUPSKI SYNDROME; PTLS;;CHROMOSOME 17P11.2 DUPLICATION SYNDROME (OMIM:610883)	
0.0001	2.5905	NORRIE DISEASE (OMIM:310600)	NDP
0.0001	2.5823	FRAGILE X MENTAL RETARDATION SYNDROME (OMIM:300624)	FMR1

Reference:
Köhler S, Schulz MH, Krawitz P, Bauer S, Dölken S, Ott CE, Mundlos C, Horn D, Mundlos S, Robinson PN
Clinical Diagnostics in Human Genetics with Semantic Similarity Searches in Ontologies
The American Journal of Human Genetics 85, pp. 457-464, Oktober 2009.

p-Value	*Score*	*Disease entry*	*Known Genes*
0.0001	2.5222	#261600 PHENYLKETONURIA; PKU;;PHENYLALANINE HYDROXYLASE DEFICIENCY;;PAH DEFICIENCY;;OLIGOPHRENIA PHENYLPYRUVICA;;FOLLING DISEASE-HYPERPHENYLALANINEMIA, NON-PKU MILD, INCLUDED;;HPA, NON-PKU MILD, INCLUDED;;PHENYLKETONURIA, MATERNAL, INCLUDED (OMIM:261600)	PAH
0.0001	2.5131	#300352 CEREBRAL CREATINE DEFICIENCY SYNDROME 1; CCDS1;;CREATINE DEFICIENCY SYNDROME, X-LINKED;;CREATINE TRANSPORTER DEFECT;;MENTAL RETARDATION, X-LINKED, WITH SEIZURES, SHORT STATURE, AND MIDFACEHYPOPLASIA;;MENTAL RETARDATION, X-LINKED, WITH CREATINE TRANSPORT DEFICIENCY (OMIM:300352)	SLC6A8

4 Further analysis

(Shown is a list of features that are special to the corresponding OMIM entry and not shared by another OMIM entry from the result list.)

OMIM entry	*Features*
#176270 PRADER-WILLI SYNDROME; PWS;;PRADER-LABHART-WILLI SYNDROMEPRADER-WILLI SYNDROME CHROMOSOME REGION, INCLUDED; PWCR, INCLUDED;;PRADER-WILLI-LIKE SYNDROME ASSOCIATED WITH CHROMOSOME 6, INCLUDED (OMIM:176270)	
#613406 CHROMOSOME 15Q24 DELETION SYNDROMECHROMOSOME 15Q24 DUPLICATION SYNDROME, INCLUDED (OMIM:613406)	
#606232 CHROMOSOME 22Q13.3 DELETION SYNDROME;;TELOMERIC 22Q13 MONOSOMY SYNDROME;;PHELAN-MCDERMID SYNDROME (OMIM:606232)	
#300260 LUBS X-LINKED MENTAL RETARDATION SYNDROME; MRXSL;;MECP2 DUPLICATION SYNDROME;;MENTAL RETARDATION, X-LINKED, SYNDROMIC, LUBS TYPE;;MENTAL RETARDATION, X-LINKED, WITH RECURRENT RESPIRATORY INFECTIONS (OMIM:300260)	
#610253 KLEEFSTRA SYNDROME;;CHROMOSOME 9Q34.3 DELETION SYNDROME;;9Q- SYNDROME;;9Q SUBTELOMERIC DELETION SYNDROME (OMIM:610253)	
#610883 POTOCKI-LUPSKI SYNDROME; PTLS;;CHROMOSOME 17P11.2 DUPLICATION SYNDROME (OMIM:610883)	

Reference:
Köhler S, Schulz MH, Krawitz P, Bauer S, Dölken S, Ott CE, Mundlos C, Horn D, Mundlos S, Robinson PN
Clinical Diagnostics in Human Genetics with Semantic Similarity Searches in Ontologies
The American Journal of Human Genetics 85, pp. 457-464, Oktober 2009.

OMIM entry	*Features*
NORRIE DISEASE (OMIM:310600)	
FRAGILE X MENTAL RETARDATION SYNDROME (OMIM:300624)	
#261600 PHENYLKETONURIA; PKU;;PHENYLALANINE HYDROXYLASE DEFICIENCY;;PAH DEFICIENCY;;OLIGOPHRENIA PHENYLPYRUVICA;;FOLLING DISEASEHYPERPHENYLALANINEMIA, NON-PKU MILD, INCLUDED;;HPA, NON-PKU MILD, INCLUDED;;PHENYLKETONURIA, MATERNAL, INCLUDED (OMIM:261600)	
#300352 CEREBRAL CREATINE DEFICIENCY SYNDROME 1; CCDS1;;CREATINE DEFICIENCY SYNDROME, X-LINKED;;CREATINE TRANSPORTER DEFECT;;MENTAL RETARDATION, X-LINKED, WITH SEIZURES, SHORT STATURE, AND MIDFACEHYPOPLASIA;;MENTAL RETARDATION, X-LINKED, WITH CREATINE TRANSPORT DEFICIENCY (OMIM:300352)	
	**Abnormality of head or neck:**<br>- Myopathic facies (HP:0002058)<br>- Mask-like facies (HP:0000298)<br>**Abnormality of the abdomen:**<br>- Ileus (HP:0002595)<br>- Aganglionic megacolon (HP:0002251)<br>**Abnormality of the ear:**<br>- Underfolded superior helices (HP:0008583)<br>**Abnormality of the eye:**<br>- Exotropia (HP:0000577)<br>**Abnormality of the integument:**<br>- Cutis laxa (HP:0000973)<br>**Abnormality of the nervous system:**<br>- Poor hand-eye coordination (HP:0007057)<br>- Aganglionic megacolon (HP:0002251)<br>- Dystonia (HP:0001332)

Reference:
Köhler S, Schulz MH, Krawitz P, Bauer S, Dölken S, Ott CE, Mundlos C, Horn D, Mundlos S, Robinson PN
Clinical Diagnostics in Human Genetics with Semantic Similarity Searches in Ontologies
The American Journal of Human Genetics 85, pp. 457-464, Oktober 2009.

1 Patient data

Name, Firstname: K10031-10133

Date of birth:

Gender: Male ○ Female ◉

2 Query

Reference:

Köhler S, Schulz MH, Krawitz P, Bauer S, Dölken S, Ott CE, Mundlos C, Horn D, Mundlos S, Robinson PN
Clinical Diagnostics in Human Genetics with Semantic Similarity Searches in Ontologies
The American Journal of Human Genetics 85, pp. 457-464, Oktober 2009.

Query Terms:

- Arthritis (HP:0001369)
- Paresthesia (HP:0003401)
- Myopia (HP:0000545)
- Anxiety (HP:0000739)
- Ischemic stroke (HP:0002140)
- Migraine (HP:0002076)
- Heat intolerance (HP:0002046)
- Resting tremor (HP:0002322)
- Urinary retention (HP:0000016)
- Apraxia (HP:0002186)
- Nausea (HP:0002018)
- Urinary incontinence (HP:0000020)
- Bradycardia (HP:0001662)
- Auditory hallucinations (HP:0008765)
- Tachycardia (HP:0001649)
- Patent foramen ovale (HP:0001655)
- Lymphadenopathy (HP:0002716)
- Asthma (HP:0002099)
- Seizures (HP:0001250)
- Depression (HP:0000716)
- Diplopia (HP:0000651)
- Gastroparesis (HP:0002578)
- Postural tremor (HP:0002174)
- Arthralgia (HP:0002829)
- Dysarthria (HP:0001260)
- Acrocyanosis (HP:0001063)
- Muscle weakness (HP:0001324)
- Frequent falls (HP:0002359)
- Joint stiffness (HP:0001387)
- Syncope (HP:0001279)
- Dyspnea (HP:0002094)
- Fatigue (HP:0012378)
- Visual hallucinations (HP:0002367)
- Astigmatism (HP:0000483)

Inheritance:	none
Similarity measure:	Resnik (not symmetric)

3 Results

p-Value	*Score*	*Disease entry*	*Known Genes*
0.0018	2.6115	TEMPORAL ARTERITIS (OMIM:187360)	
0.0018	2.5548	#176000 PORPHYRIA, ACUTE INTERMITTENT;;AIP;;PORPHYRIA, SWEDISH TYPE;;PORPHOBILINOGEN DEAMINASE DEFICIENCY;;PBGD DEFICIENCY;;UROPORPHYRINOGEN SYNTHASE DEFICIENCY;;UPS DEFICIENCYPORPHYRIA, ACUTE INTERMITTENT, NONERYTHROID VARIANT, INCLUDED;;PORPHYRIA, CHESTER TYPE, INCLUDED; PORC, INCLUDED (OMIM:176000)	HMBS
0.0018	2.2435	LEGIONELLOSIS (ORPHANET:549)	
0.0018	2.1483	#300623 FRAGILE X TREMOR/ATAXIA SYNDROME; FXTAS (OMIM:300623)	FMR1
0.0018	2.1410	TYPHOID (ORPHANET:99745)	
0.0018	2.1299	HEMORRHAGIC FEVER - RENAL SYNDROME (ORPHANET:340)	
0.0018	2.0991	#121300 COPROPORPHYRIA, HEREDITARY; HCP;;COPROPORPHYRINOGEN OXIDASE DEFICIENCY;;CPOX DEFICIENCY;;CPO DEFICIENCY;;CPX DEFICIENCYHARDEROPORPHYRIA, INCLUDED (OMIM:121300)	CPOX
0.0018	2.0177	SCRUB TYPHUS (ORPHANET:83317)	
0.0018	1.8736	LYME DISEASE (ORPHANET:91546)	
0.0018	1.8399	RABIES (ORPHANET:770)	

4 Further analysis

(Shown is a list of features that are special to the corresponding OMIM entry and not shared by another OMIM entry from the result list.)

Reference:
Köhler S, Schulz MH, Krawitz P, Bauer S, Dölken S, Ott CE, Mundlos C, Horn D, Mundlos S, Robinson PN
Clinical Diagnostics in Human Genetics with Semantic Similarity Searches in Ontologies
The American Journal of Human Genetics 85, pp. 457-464, Oktober 2009.

OMIM entry	*Features*
TEMPORAL ARTERITIS (OMIM:187360)	
#176000 PORPHYRIA, ACUTE INTERMITTENT;;AIP;;PORPHYRIA, SWEDISH TYPE;;PORPHOBILINOGEN DEAMINASE DEFICIENCY;;PBGD DEFICIENCY;;UROPORPHYRINOGEN SYNTHASE DEFICIENCY;;UPS DEFICIENCYPORPHYRIA, ACUTE INTERMITTENT, NONERYTHROID VARIANT, INCLUDED;;PORPHYRIA, CHESTER TYPE, INCLUDED; PORC, INCLUDED (OMIM:176000)	
LEGIONELLOSIS (ORPHANET:549)	
#300623 FRAGILE X TREMOR/ATAXIA SYNDROME; FXTAS (OMIM:300623)	
TYPHOID (ORPHANET:99745)	
HEMORRHAGIC FEVER - RENAL SYNDROME (ORPHANET:340)	
#121300 COPROPORPHYRIA, HEREDITARY; HCP;;COPROPORPHYRINOGEN OXIDASE DEFICIENCY;;CPOX DEFICIENCY;;CPO DEFICIENCY;;CPX DEFICIENCYHARDEROPORPHYRIA, INCLUDED (OMIM:121300)	
RABIES (ORPHANET:770)	**Abnormality of head or neck:**<br>- Excessive salivation (HP:0003781)<br>**Abnormality of the nervous system:**<br>- Cerebral palsy (HP:0100021)<br>- Attention deficit hyperactivity disorder (HP:0007018)<br>**Abnormality of the respiratory system:**<br>- Vocal cord paresis (HP:0001604)
SCRUB TYPHUS (ORPHANET:83317)	
LYME DISEASE (ORPHANET:91546)	

Reference:
Köhler S, Schulz MH, Krawitz P, Bauer S, Dölken S, Ott CE, Mundlos C, Horn D, Mundlos S, Robinson PN
Clinical Diagnostics in Human Genetics with Semantic Similarity Searches in Ontologies
The American Journal of Human Genetics 85, pp. 457-464, Oktober 2009.

1 Patient data

Name, Firstname: K10031-10145

Date of birth:

Gender: Male ○ Female ◉

2 Query

Query Terms:

- Hypotension (HP:0002615)
- Abnormality of iron homeostasis (HP:0011031)
- Osteoporosis (HP:0000939)
- Mood swings (HP:0000720)
- Anxiety (HP:0000739)
- Syncope (HP:0001279)
- Obsessive-compulsive behavior (HP:0000722)
- Heat intolerance (HP:0002046)

Inheritance:	none
Similarity measure:	Resnik (not symmetric)

3 Results

p-Value	*Score*	*Disease entry*	*Known Genes*
0.0048	2.8899	AROMATIC L-AMINO ACID DECARBOXYLASE DEFICIENCY (OMIM:608643)	DDC
0.0048	2.8226	#615830 PIGMENTED NODULAR ADRENOCORTICAL DISEASE, PRIMARY, 4; PPNAD4;;CUSHING SYNDROME, ADRENAL, DUE TO PPNAD4;;CHROMOSOME 19P13 DUPLICATION SYNDROMEACTH-INDEPENDENT ADRENAL CUSHING SYNDROME, SOMATIC, INCLUDED (OMIM:615830)	PRKACA

Reference:
Köhler S, Schulz MH, Krawitz P, Bauer S, Dölken S, Ott CE, Mundlos C, Horn D, Mundlos S, Robinson PN
Clinical Diagnostics in Human Genetics with Semantic Similarity Searches in Ontologies
The American Journal of Human Genetics 85, pp. 457-464, Oktober 2009.

p-Value	*Score*	*Disease entry*	*Known Genes*
0.0048	2.3584	#615962 GLUCOCORTICOID RESISTANCE, GENERALIZED;;GLUCOCORTICOID RECEPTOR DEFICIENCY;;GCCR DEFICIENCY;;GCR DEFICIENCY;;GRL DEFICIENCY;;CORTISOL RESISTANCE FROM GLUCOCORTICOID RECEPTOR DEFECT (OMIM:615962)	NR3C1
0.0048	2.2984	NIPAH VIRUS DISEASE (ORPHANET:99825)	
0.0048	2.2960	NEPHROGENIC SYNDROME OF INAPPROPRIATE ANTIDIURESIS (OMIM:300539)	AVPR2
0.0048	1.4841	#118700 CHOREA, BENIGN HEREDITARY; BHC;;BCH;;HEREDITARY PROGRESSIVE CHOREA WITHOUT DEMENTIA (OMIM:118700)	NKX2-1
0.0048	1.4841	PANIC DISORDER 1 (OMIM:167870)	
0.0048	1.2550	#164230 OBSESSIVE-COMPULSIVE DISORDER; OCD (OMIM:164230)	
0.0070	3.5068	#223900 NEUROPATHY, HEREDITARY SENSORY AND AUTONOMIC, TYPE III; HSAN3;;HSAN III;;DYSAUTONOMIA, FAMILIAL; DYS; FD;;RILEY-DAY SYNDROME (OMIM:223900)	IKBKAP
0.0070	3.1603	#606159 NEURODEGENERATION WITH BRAIN IRON ACCUMULATION 3; NBIA3;;NEUROFERRITINOPATHY;;BASAL GANGLIA DISEASE, ADULT-ONSET (OMIM:606159)	FTL

4 Further analysis

(Shown is a list of features that are special to the corresponding OMIM entry and not shared by another OMIM entry from the result list.)

OMIM entry	*Features*
AROMATIC L-AMINO ACID DECARBOXYLASE DEFICIENCY (OMIM:608643)	
#615830 PIGMENTED NODULAR ADRENOCORTICAL DISEASE, PRIMARY, 4; PPNAD4;;CUSHING SYNDROME, ADRENAL, DUE TO PPNAD4;;CHROMOSOME 19P13 DUPLICATION SYNDROMEACTH-INDEPENDENT ADRENAL CUSHING SYNDROME, SOMATIC, INCLUDED (OMIM:615830)	

Reference:
Köhler S, Schulz MH, Krawitz P, Bauer S, Dölken S, Ott CE, Mundlos C, Horn D, Mundlos S, Robinson PN
Clinical Diagnostics in Human Genetics with Semantic Similarity Searches in Ontologies
The American Journal of Human Genetics 85, pp. 457-464, Oktober 2009.

OMIM entry	*Features*
#615962 GLUCOCORTICOID RESISTANCE, GENERALIZED;;GLUCOCORTICOID RECEPTOR DEFICIENCY;;GCCR DEFICIENCY;;GCR DEFICIENCY;;GRL DEFICIENCY;;CORTISOL RESISTANCE FROM GLUCOCORTICOID RECEPTOR DEFECT (OMIM:615962)	
NIPAH VIRUS DISEASE (ORPHANET:99825)	
NEPHROGENIC SYNDROME OF INAPPROPRIATE ANTIDIURESIS (OMIM:300539)	
#118700 CHOREA, BENIGN HEREDITARY; BHC;;BCH;;HEREDITARY PROGRESSIVE CHOREA WITHOUT DEMENTIA (OMIM:118700)	
PANIC DISORDER 1 (OMIM:167870)	
#164230 OBSESSIVE-COMPULSIVE DISORDER; OCD (OMIM:164230)	
#223900 NEUROPATHY, HEREDITARY SENSORY AND AUTONOMIC, TYPE III; HSAN3;;HSAN III;;DYSAUTONOMIA, FAMILIAL; DYS; FD;;RILEY-DAY SYNDROME (OMIM:223900)	
#606159 NEURODEGENERATION WITH BRAIN IRON ACCUMULATION 3; NBIA3;;NEUROFERRITINOPATHY;;BASAL GANGLIA DISEASE, ADULT-ONSET (OMIM:606159)	**Abnormality of head or neck:**<br>- Blepharospasm (HP:0000643)<br>**Abnormality of metabolism/homeostasis:**<br>- Decreased serum ferritin (HP:0012343)<br>**Abnormality of the abdomen:**<br>- Dysphagia (HP:0002015)<br>**Abnormality of the eye:**<br>- Abnormality of eye movement (HP:0000496)<br>**Abnormality of the musculature:**<br>- Spasticity (HP:0001257)<br>**Abnormality of the nervous system:**<br>- Bradykinesia (HP:0002067)<br>- Developmental regression (HP:0002376)<br>- Parkinsonism (HP:0001300)<br>- Neurodegeneration (HP:0002180)<br>- Cavitation of the basal ganglia (HP:0007007)<br>- Rigidity (HP:0002063)<br>- Mutism (HP:0002300)<br>- Ataxia (HP:0001251)<br>- Dysphagia (HP:0002015)<br>- Blepharospasm (HP:0000643)<br>- Spasticity (HP:0001257)<br>- Writer's cramp (HP:0002356)<br>- Disinhibition (HP:0000734)<br>- Anarthria (HP:0002425)<br>- Laryngeal dystonia (HP:0012049)<br>- Dementia (HP:0000726)<br>**Abnormality of the voice:**<br>- Laryngeal dystonia (HP:0012049)

Reference:
Köhler S, Schulz MH, Krawitz P, Bauer S, Dölken S, Ott CE, Mundlos C, Horn D, Mundlos S, Robinson PN
Clinical Diagnostics in Human Genetics with Semantic Similarity Searches in Ontologies
The American Journal of Human Genetics 85, pp. 457-464, Oktober 2009.

Reference:
Köhler S, Schulz MH, Krawitz P, Bauer S, Dölken S, Ott CE, Mundlos C, Horn D, Mundlos S, Robinson PN
Clinical Diagnostics in Human Genetics with Semantic Similarity Searches in Ontologies
The American Journal of Human Genetics 85, pp. 457-464, Oktober 2009.
